# Supplementary material for: Enantioselective cyanation via radical-mediated C–C single bond cleavage for synthesis of chiral dinitriles
Source: Nat Commun. 2019 Nov 26;10:5373. doi: 10.1038/s41467-019-13369-x (PMC6879615; doi:10.1038/s41467-019-13369-x)
Supplement: Supplementary file 2 — Supplementary Information [file 41467_2019_13369_MOESM2_ESM.pdf]

Enantioselective cyanation via radical-mediated C−C single bond cleavage for synthesis of chiral dinitriles

Wang *et al.*

Supplementary Figures

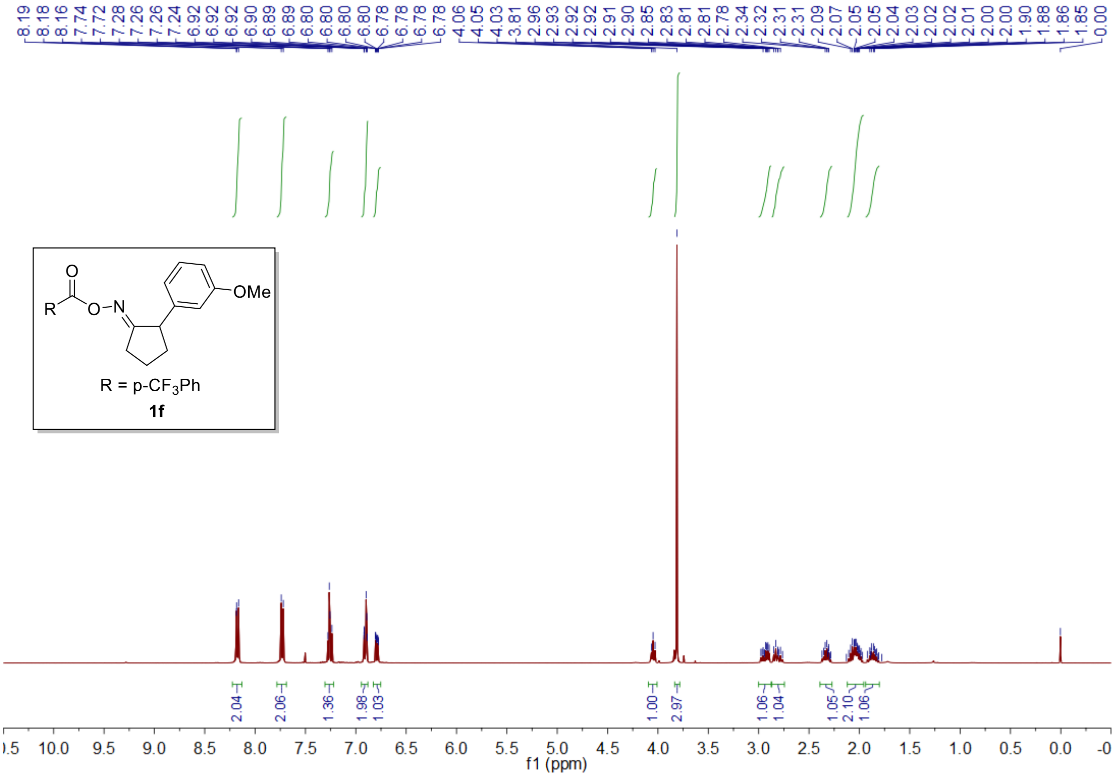

Supplementary Figure 1. ^{1}H NMR of **1f**

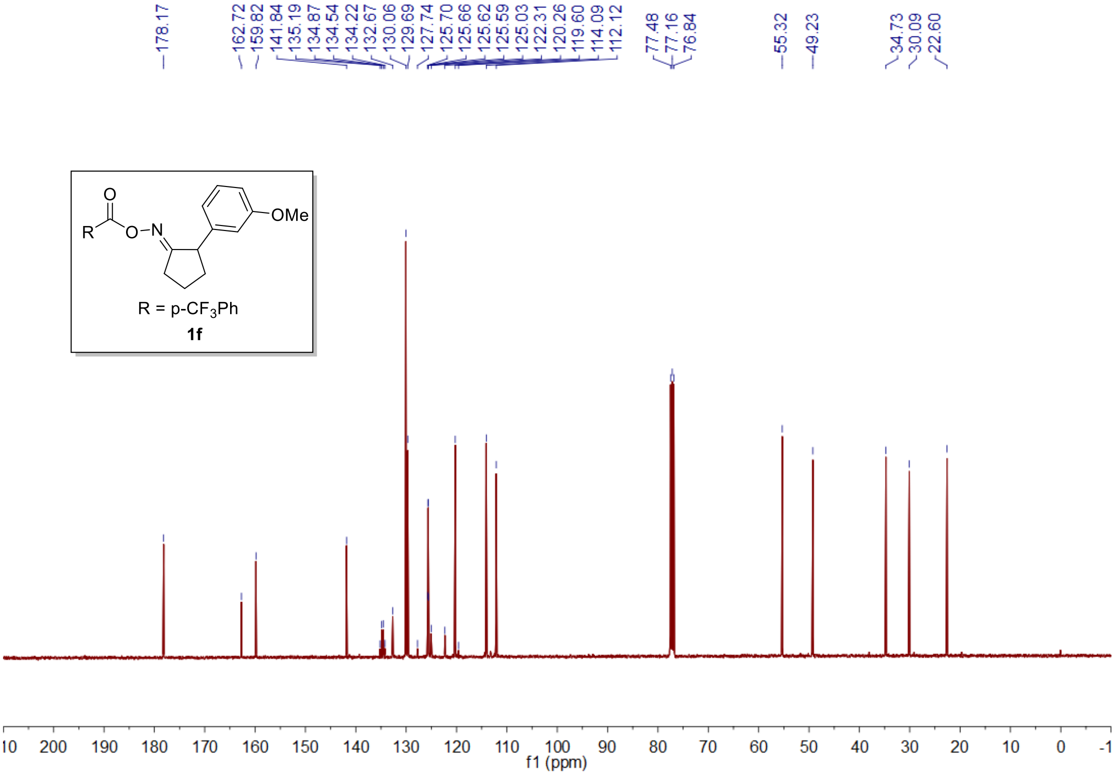

Supplementary Figure 2. ^{13}C NMR of **1f**

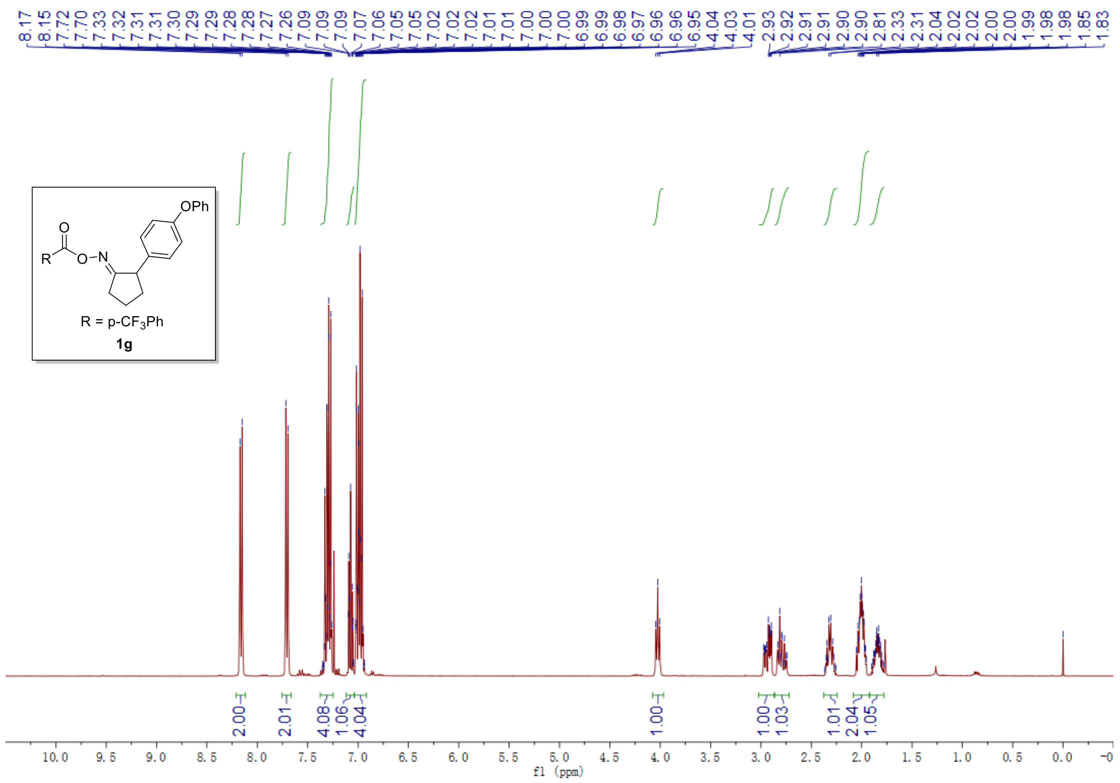

Supplementary Figure 3. ^{1}H NMR of **1g**

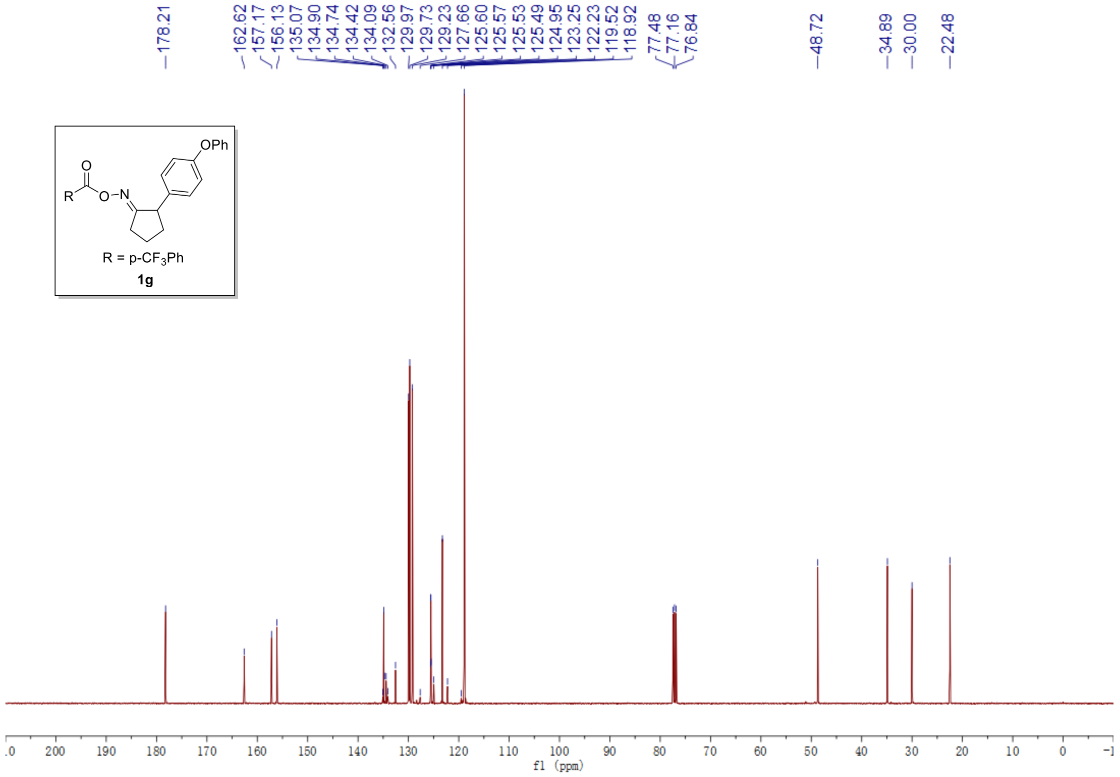

Supplementary Figure 4. ^{13}C NMR of **1g**

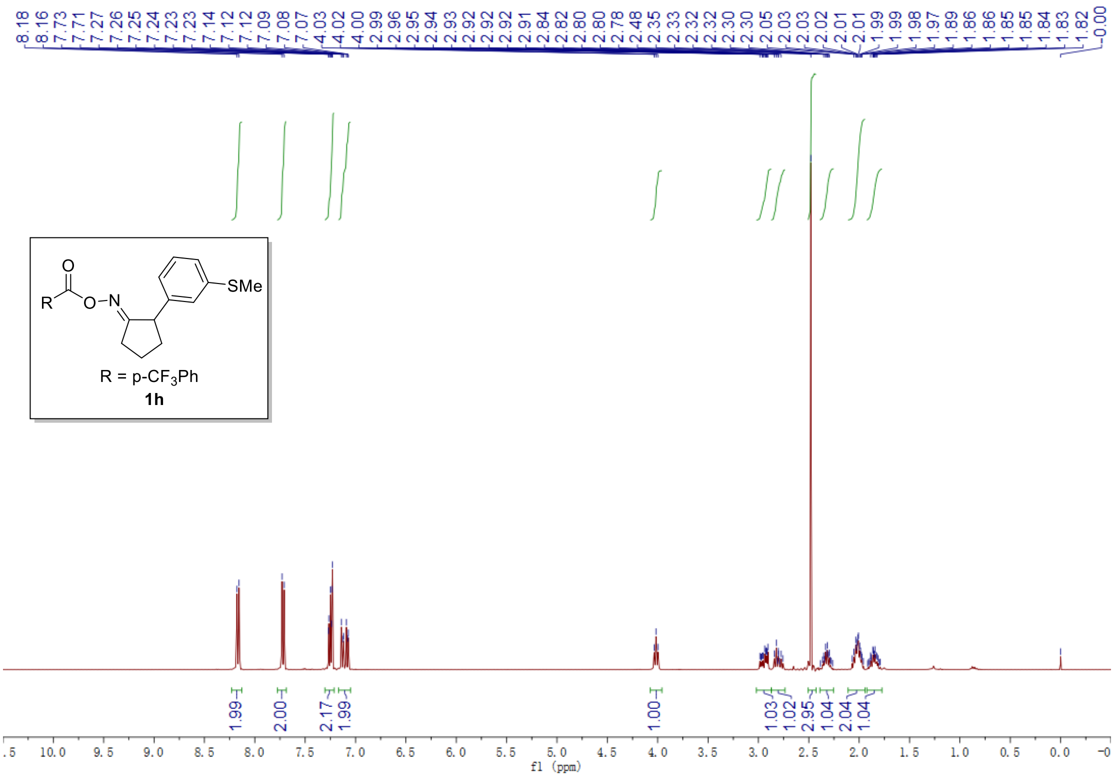

Supplementary Figure 5. ^{1}H NMR of **1h**

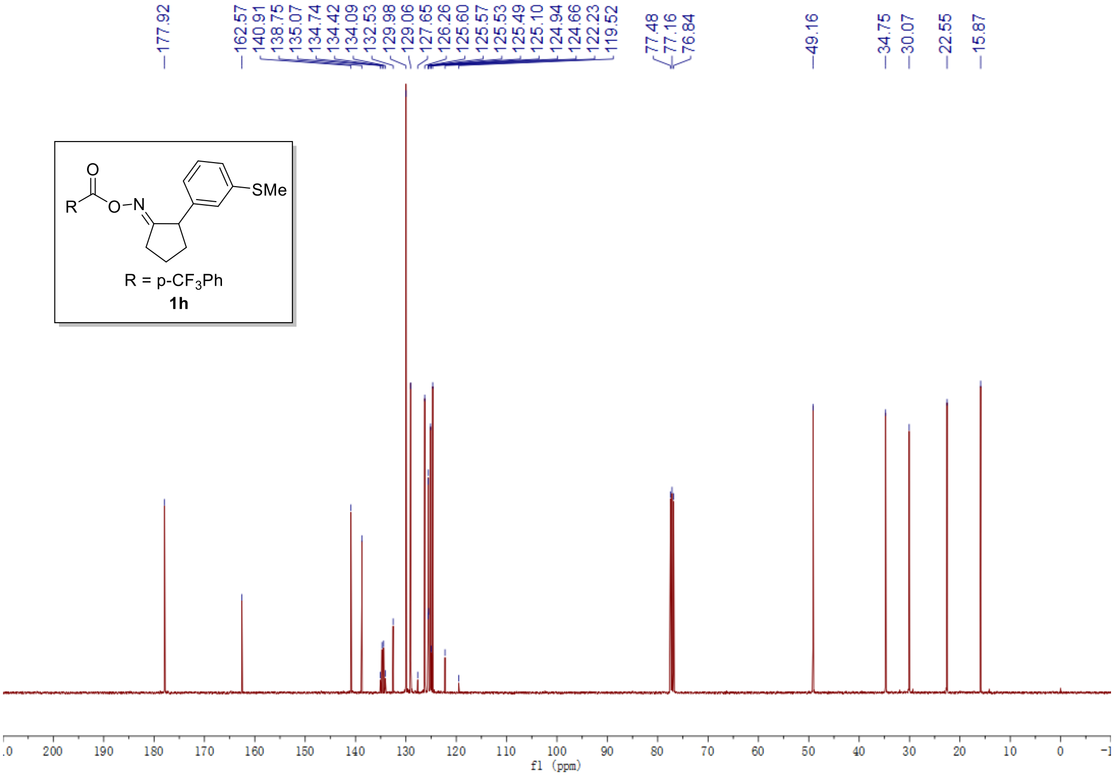

Supplementary Figure 6. ^{13}C NMR of **1h**

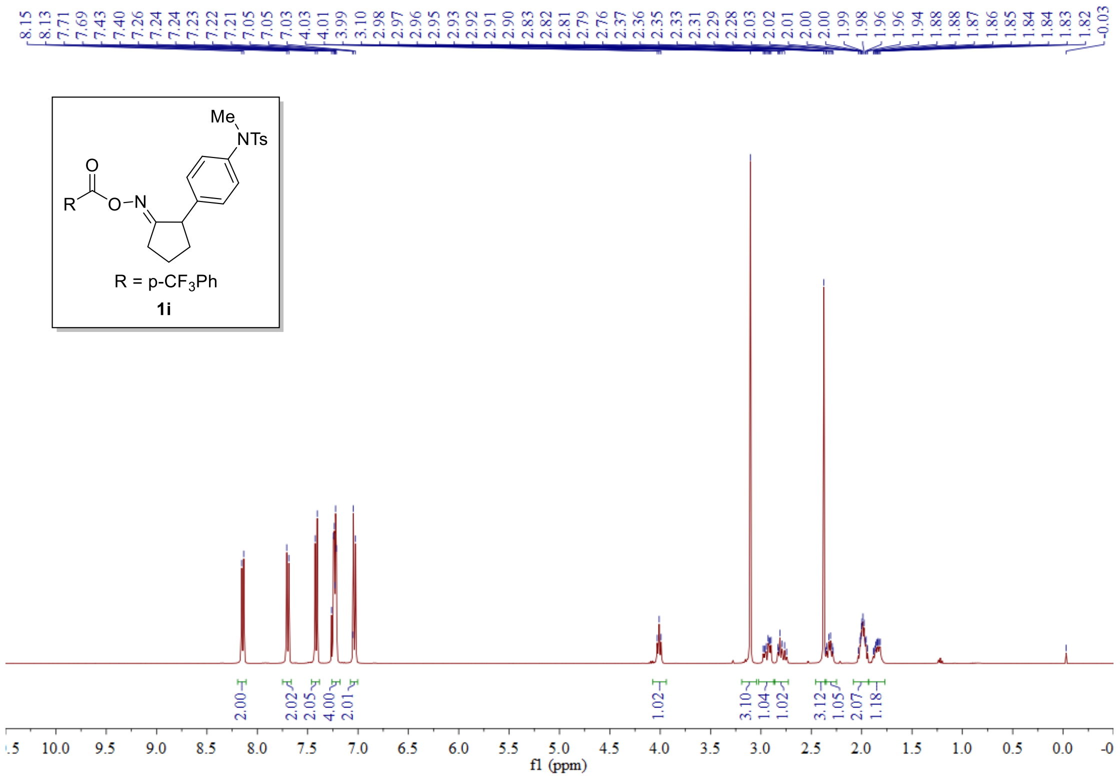

Supplementary Figure 7. ^{1}H NMR of **1i**

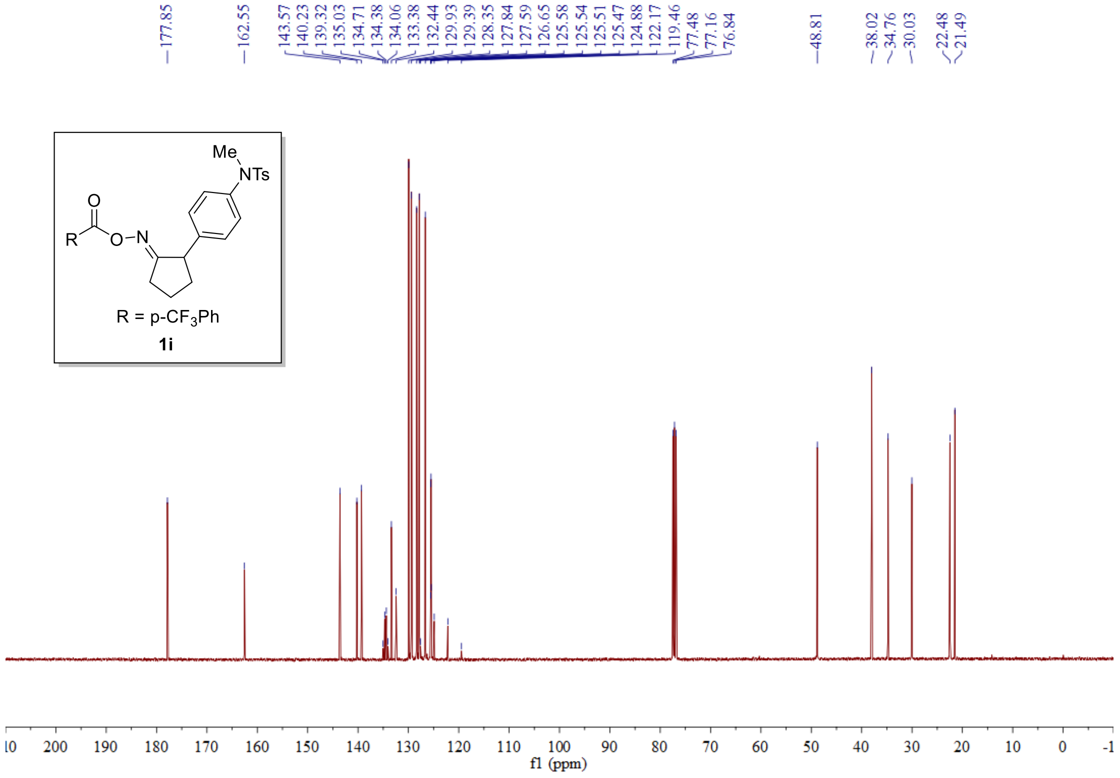

Supplementary Figure 8. ^{13}C NMR of **1i**

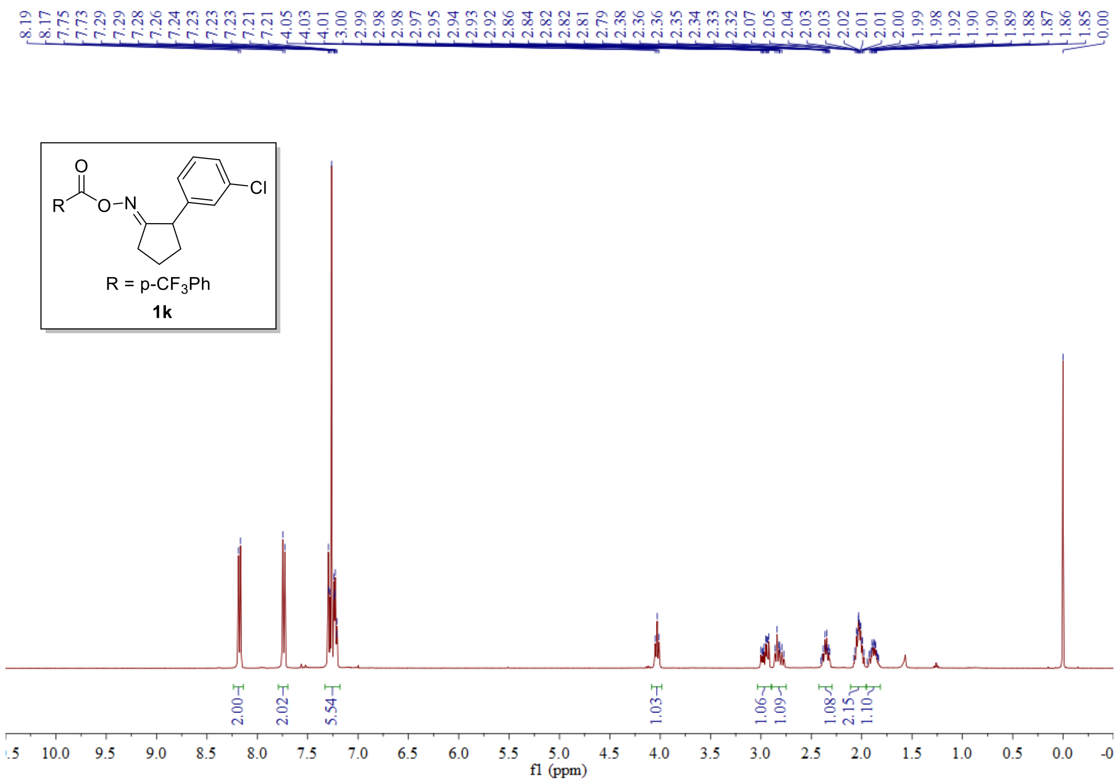

Supplementary Figure 9. ^{1}H NMR of **1k**

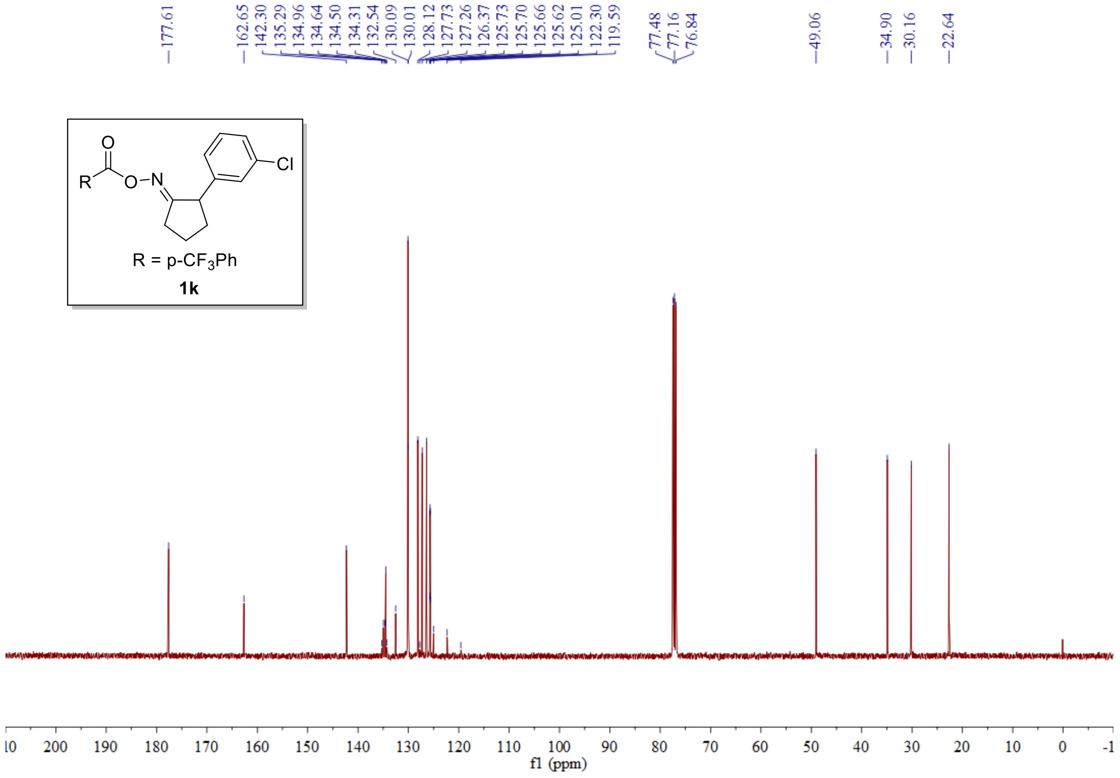

Supplementary Figure 10. ^{13}C NMR of **1k**

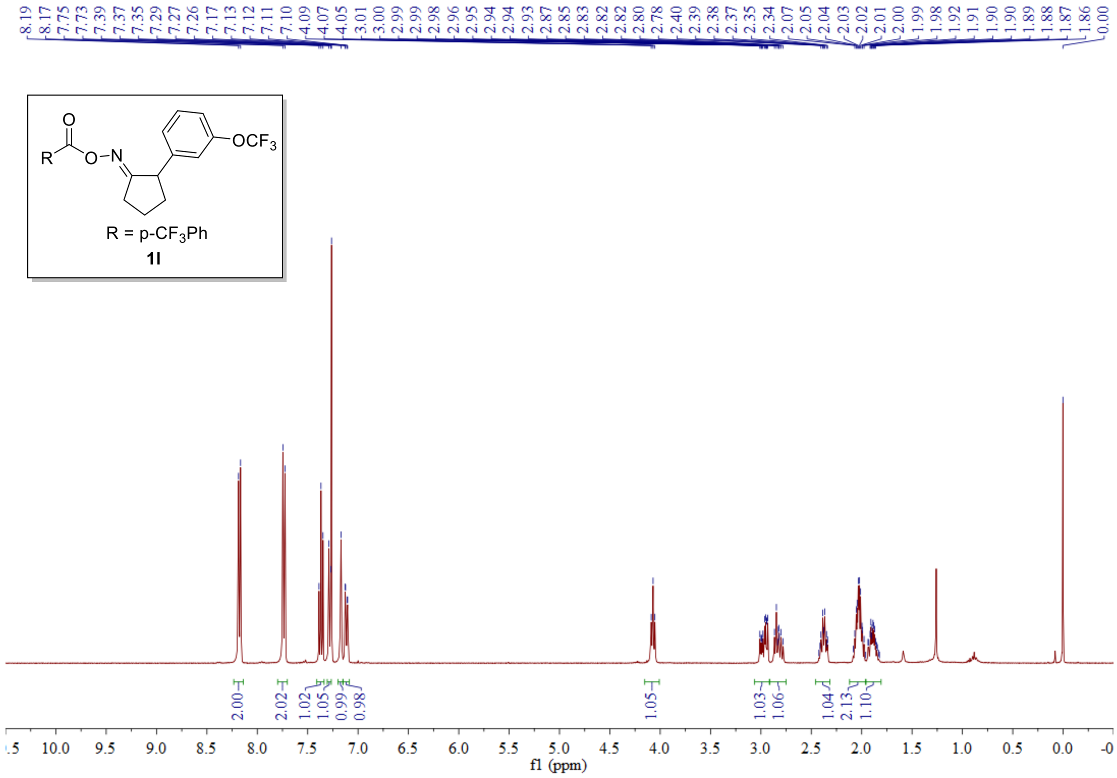

Supplementary Figure 11. ^{1}H NMR of **1l**

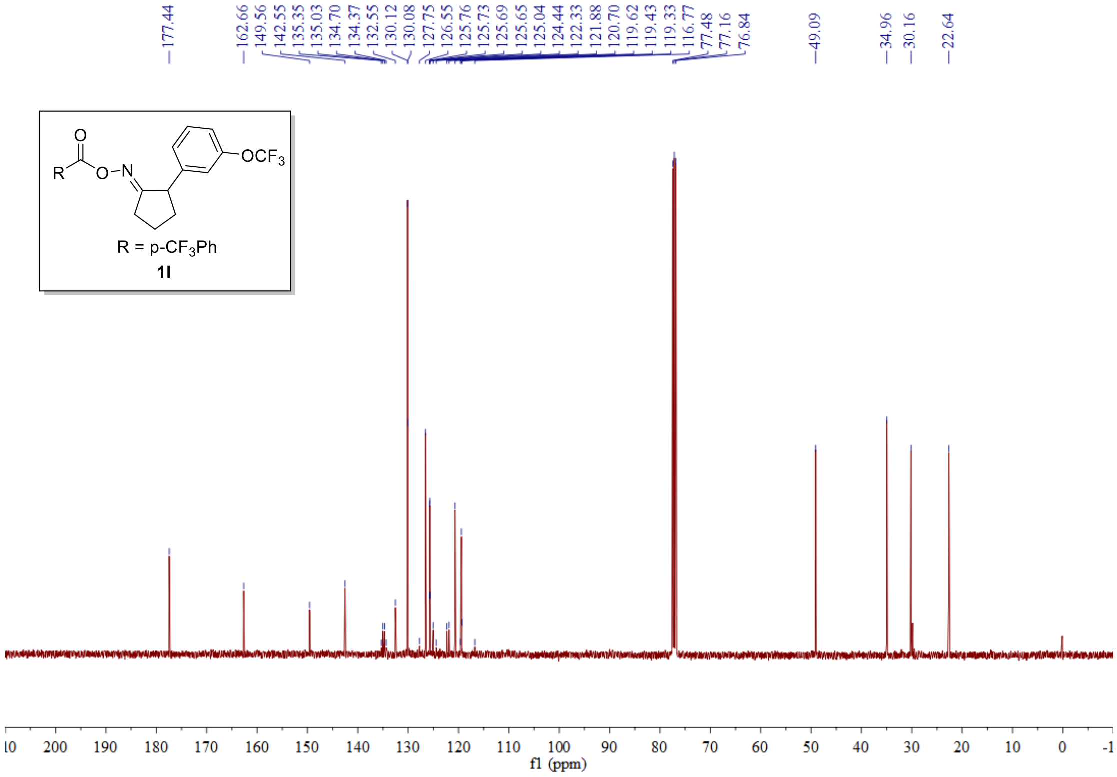

Supplementary Figure 12. ^{13}C NMR of **1l**

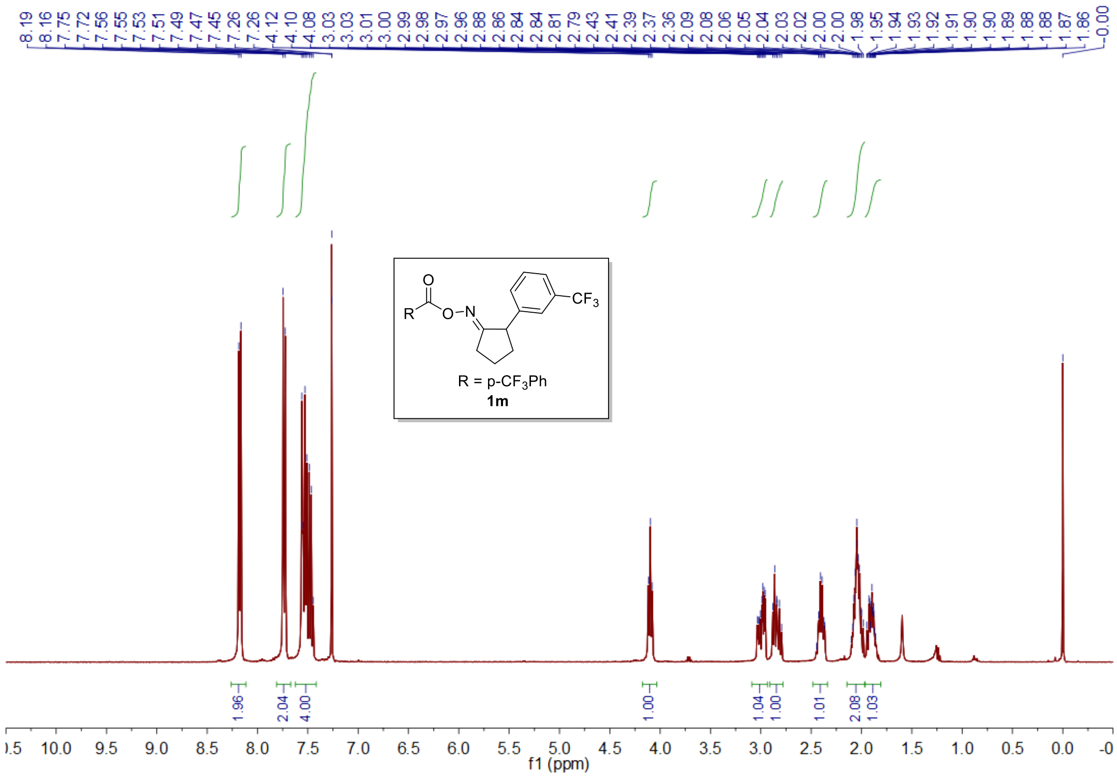

Supplementary Figure 13. ^{1}H NMR of **1m**

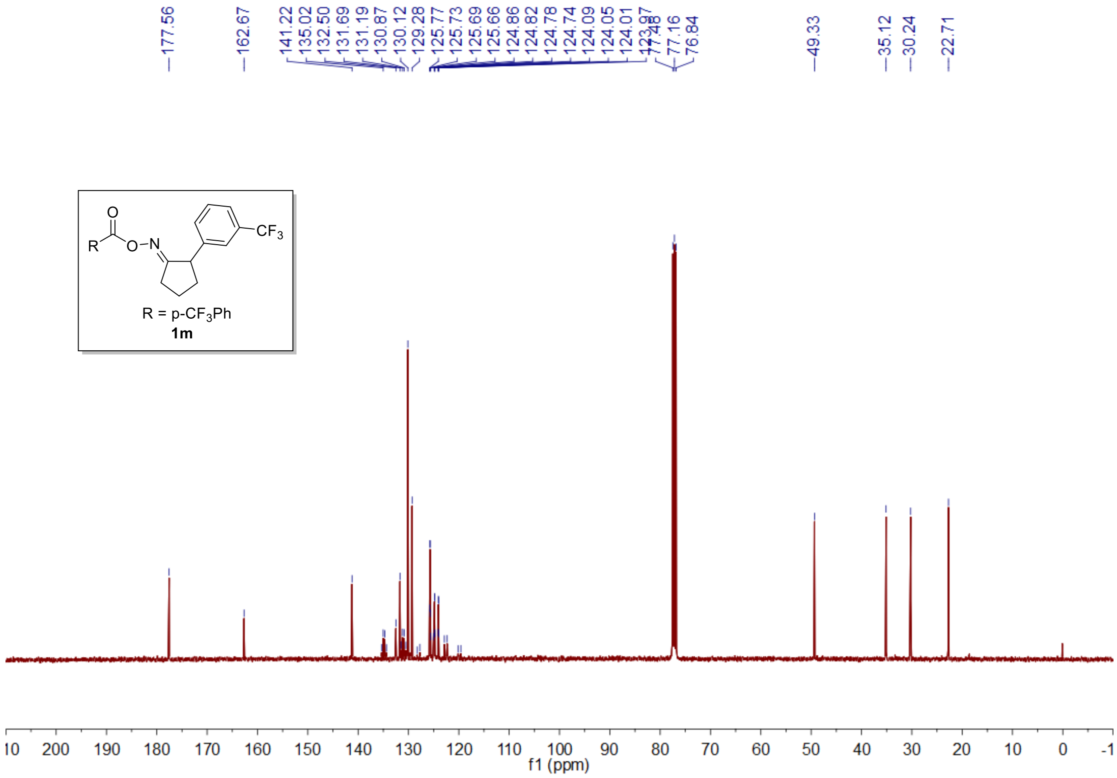

Supplementary Figure 14. ^{13}C NMR of **1m**

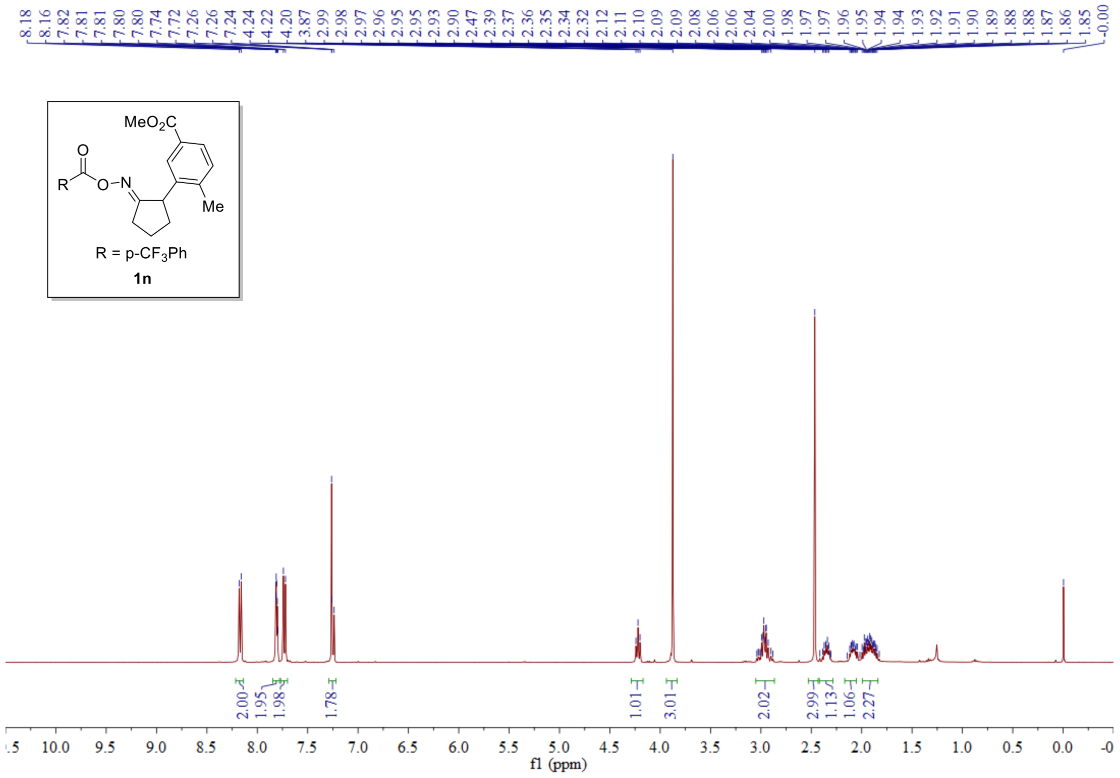

Supplementary Figure 15. ^{1}H NMR of **1n**

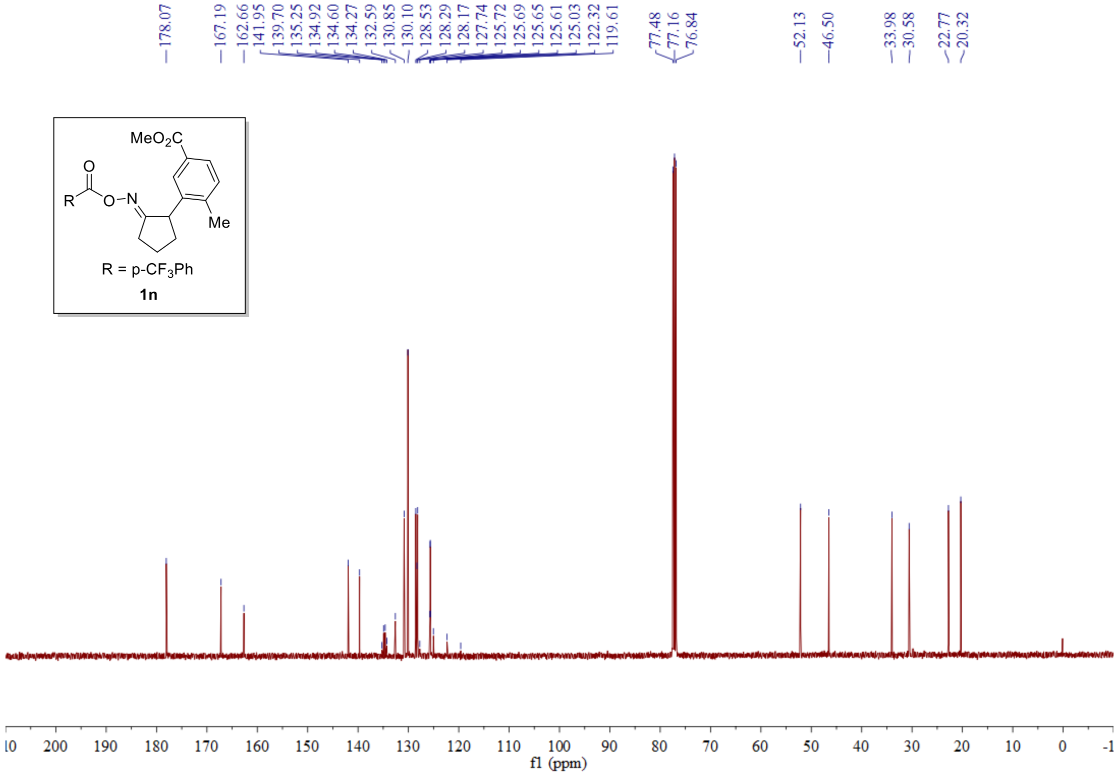

Supplementary Figure 16. ^{13}C NMR of **1n**

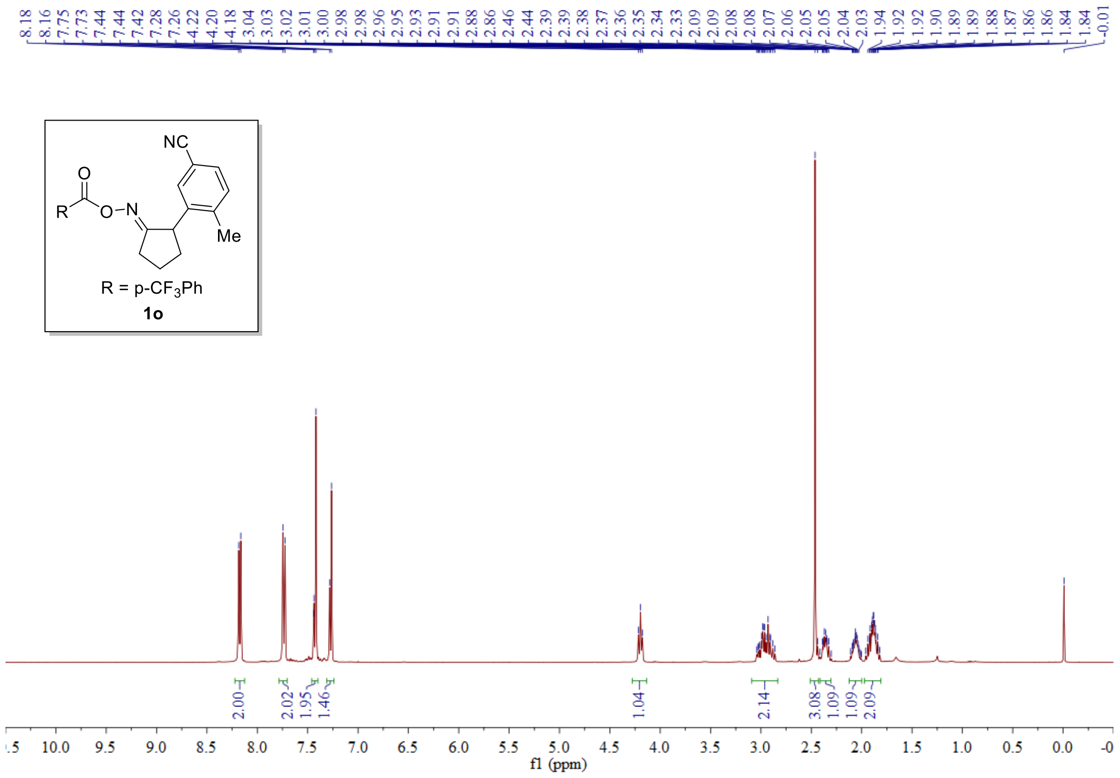

Supplementary Figure 17. ^{1}H NMR of **1o**

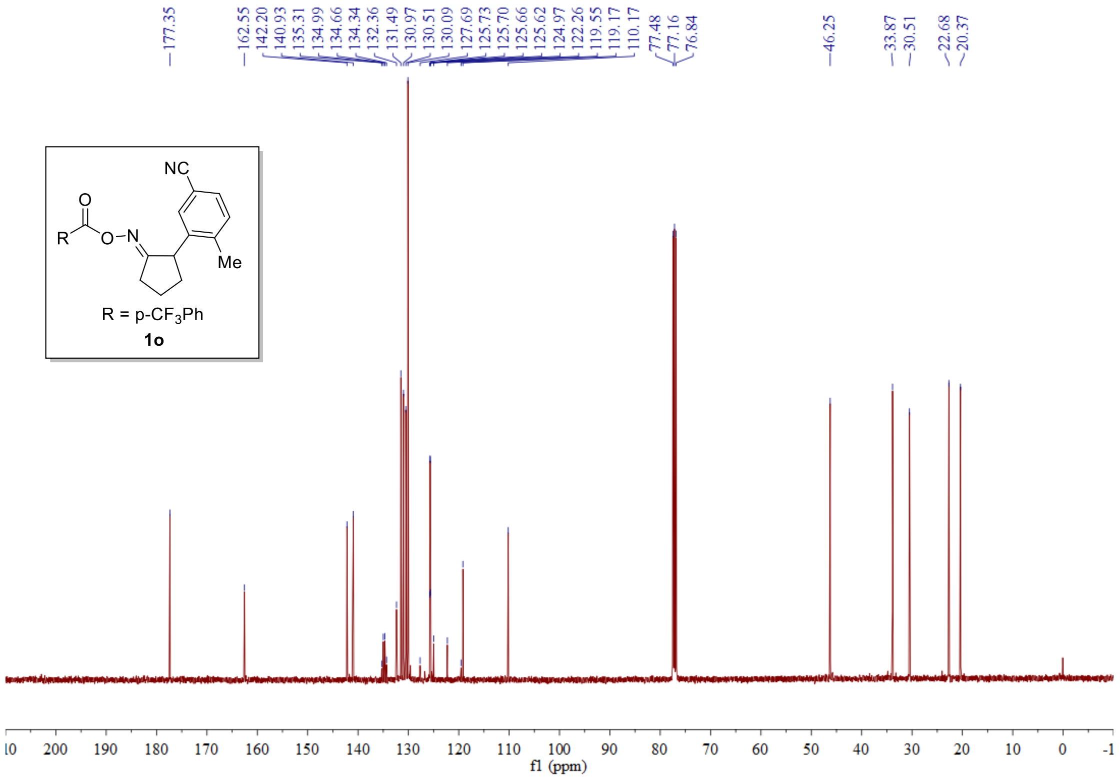

Supplementary Figure 18. ^{13}C NMR of **1o**

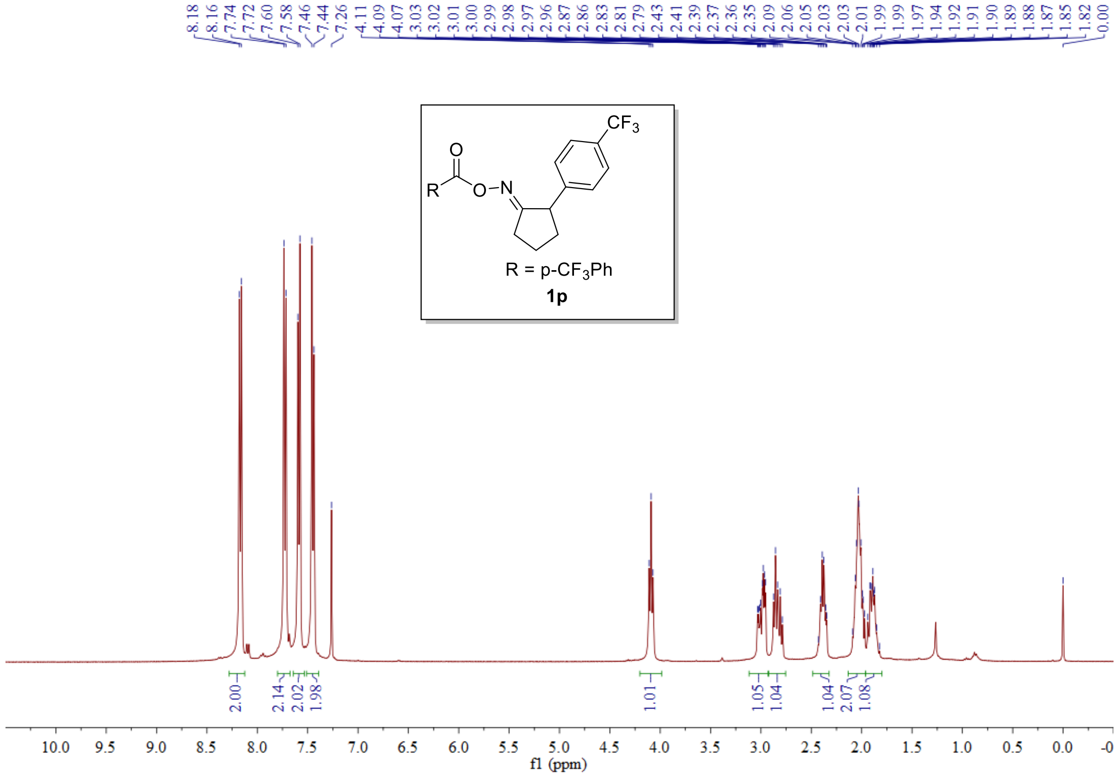

Supplementary Figure 19. ^{1}H NMR of **1p**

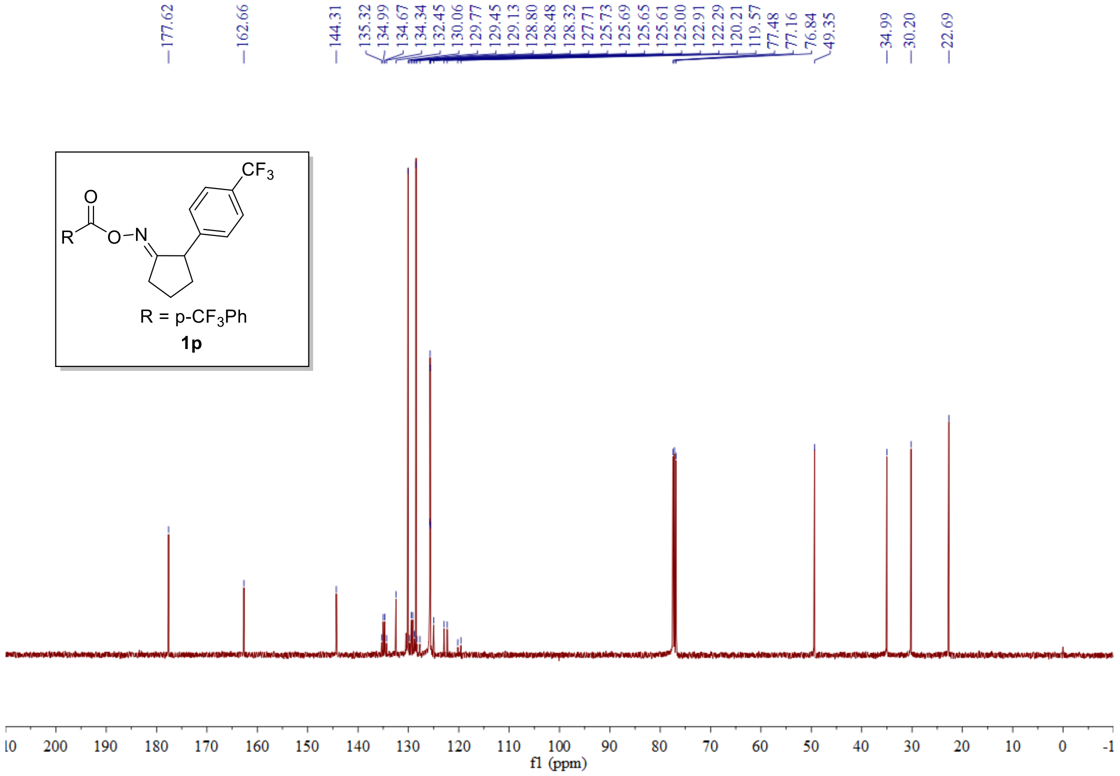

Supplementary Figure 20. ^{13}C NMR of **1p**

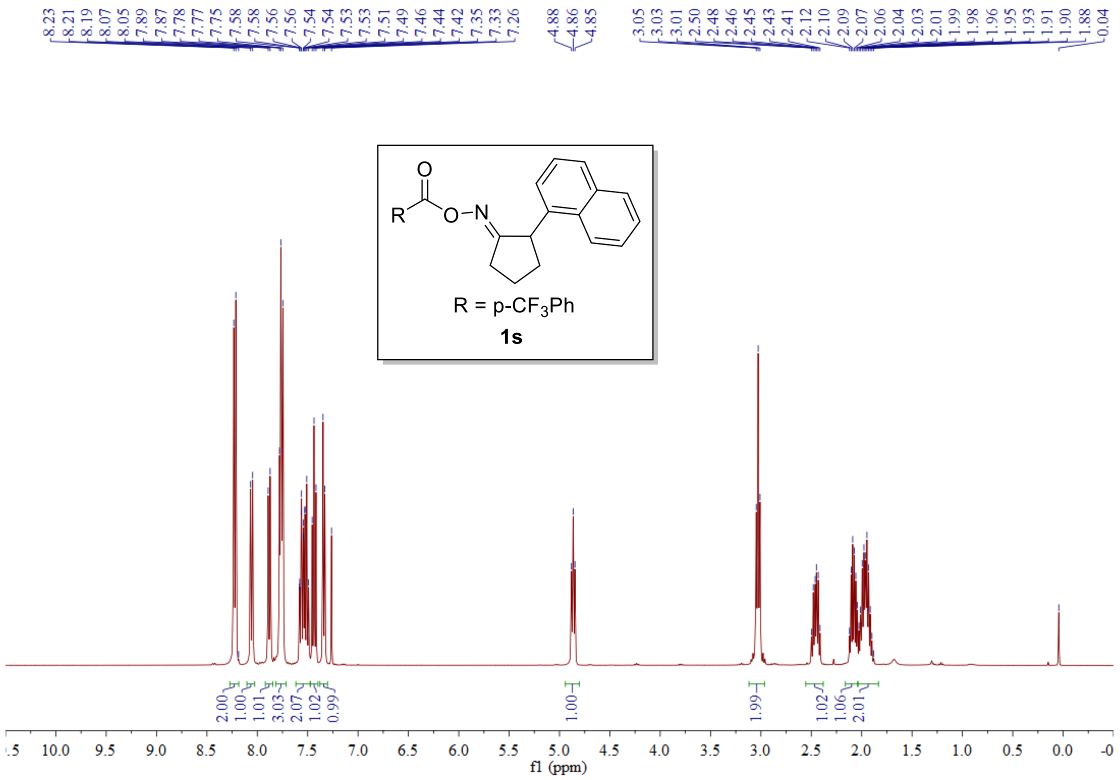

Supplementary Figure 21. ^{1}H NMR of **1s**

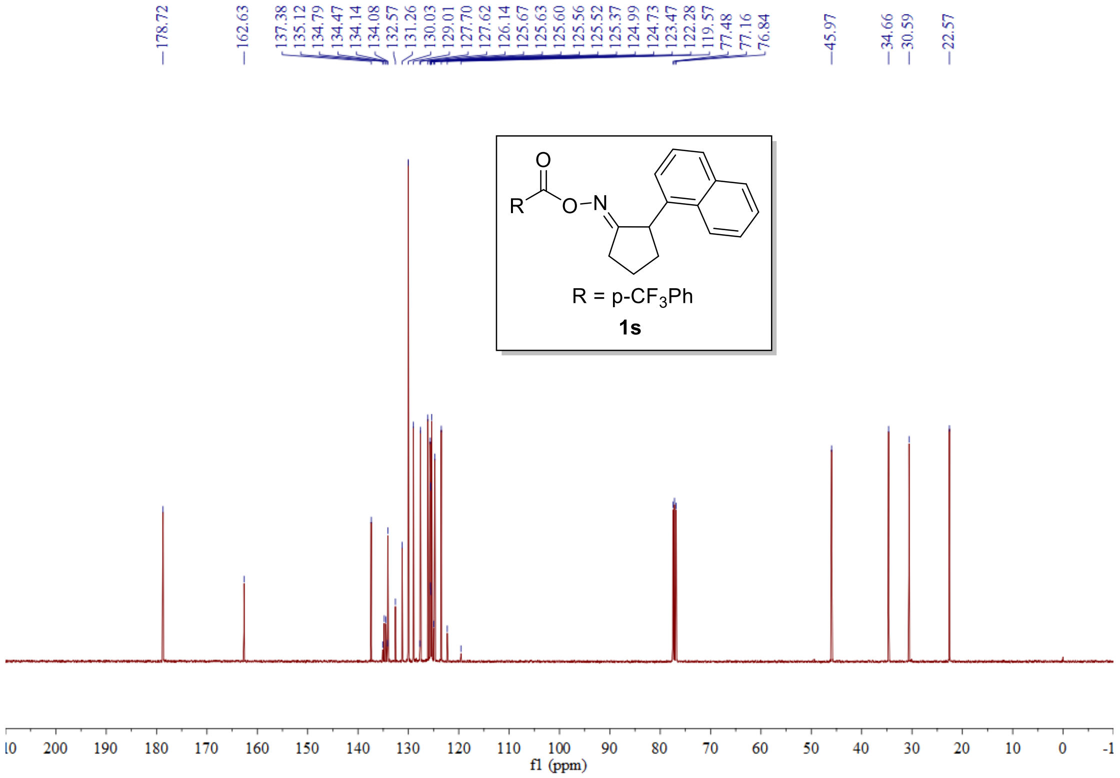

Supplementary Figure 22. ^{13}C NMR of **1s**

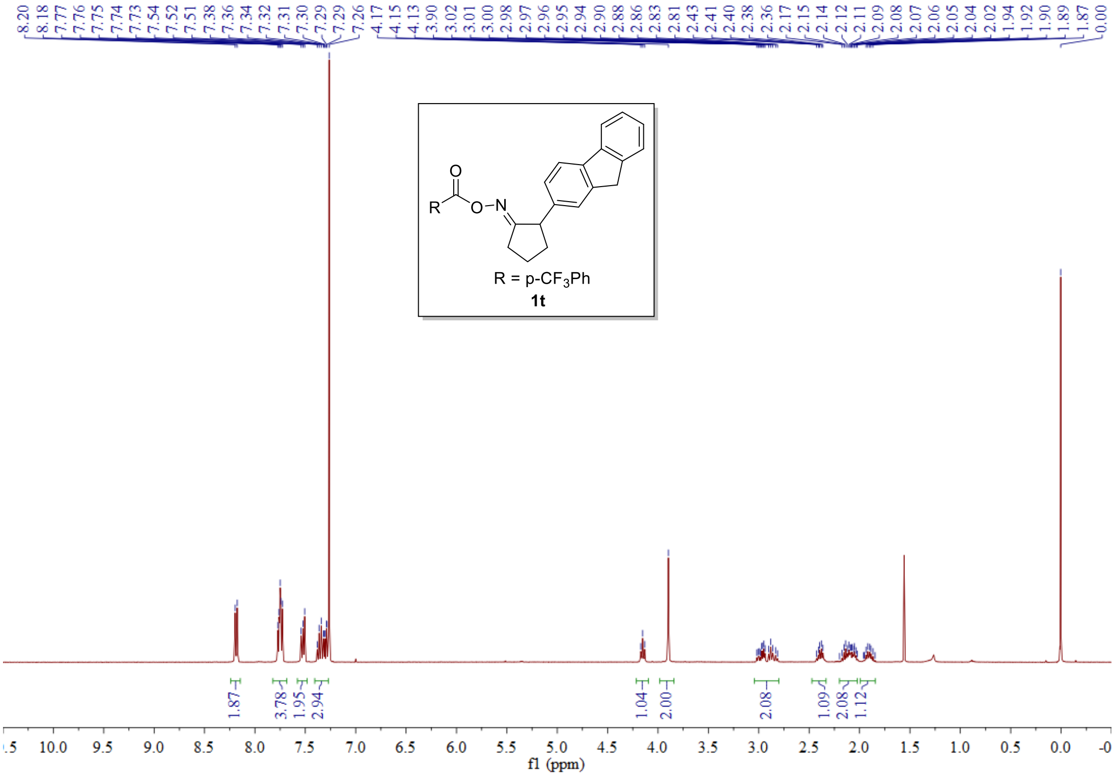

Supplementary Figure 23. ^{1}H NMR of **1t**

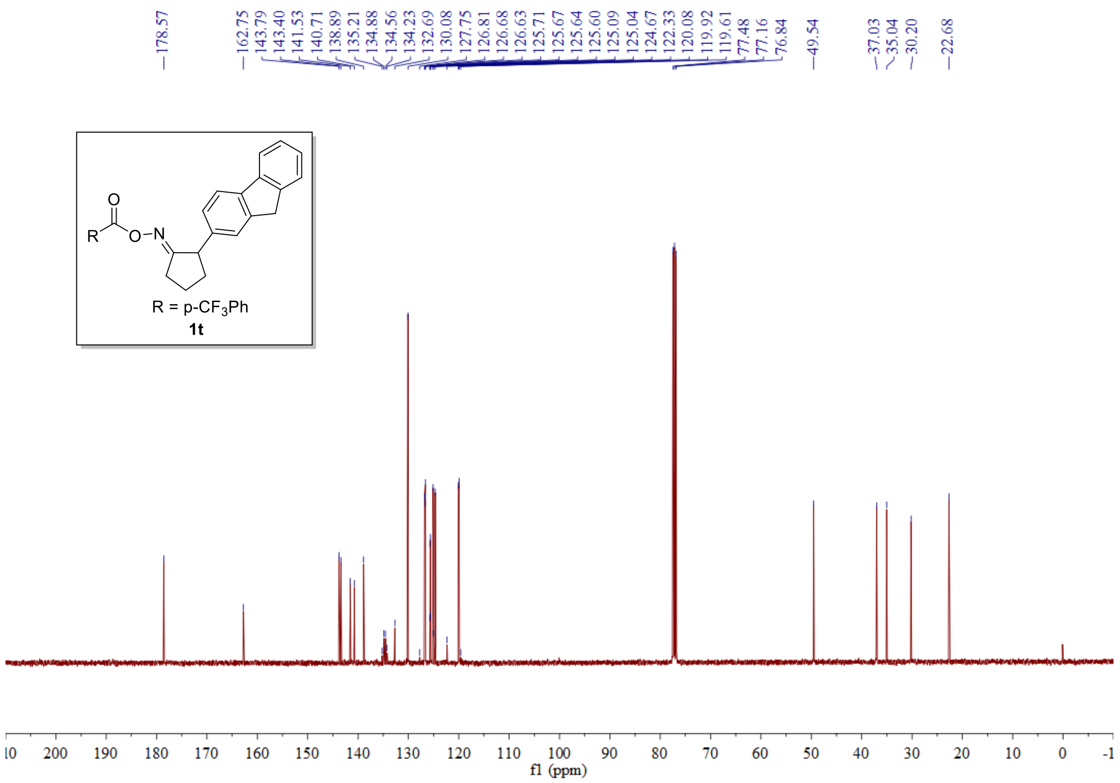

Supplementary Figure 24. ^{13}C NMR of **1t**

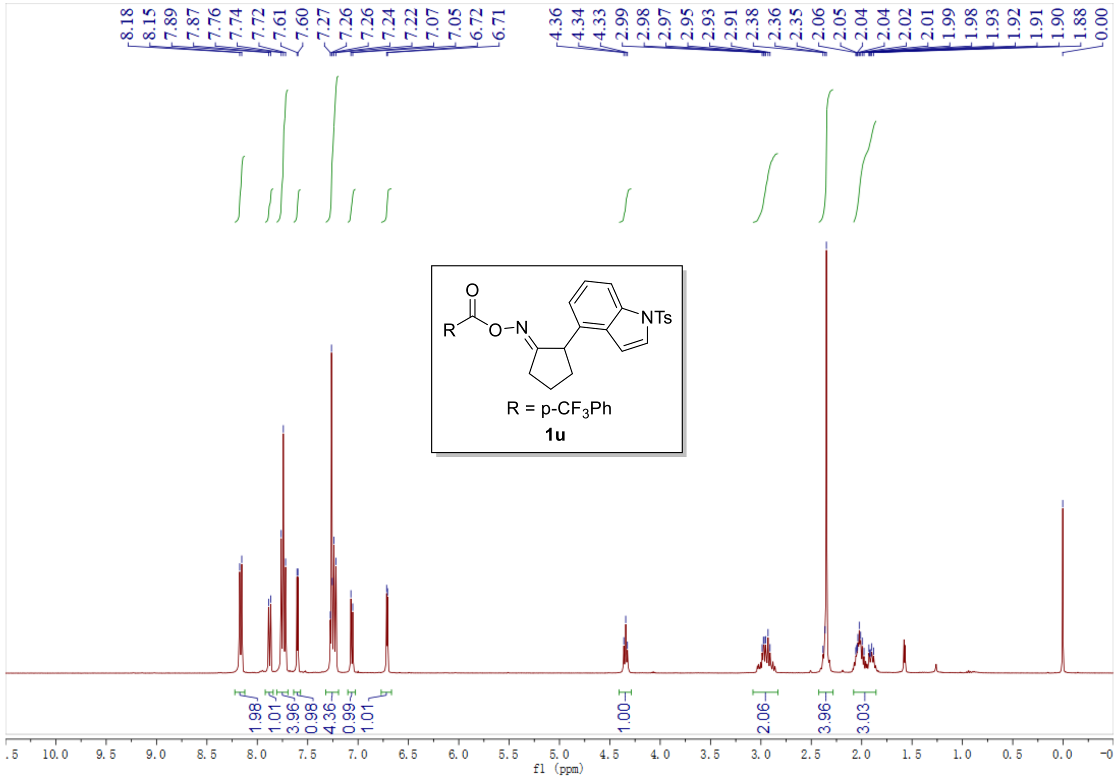

Supplementary Figure 25. ^{1}H NMR of **1u**

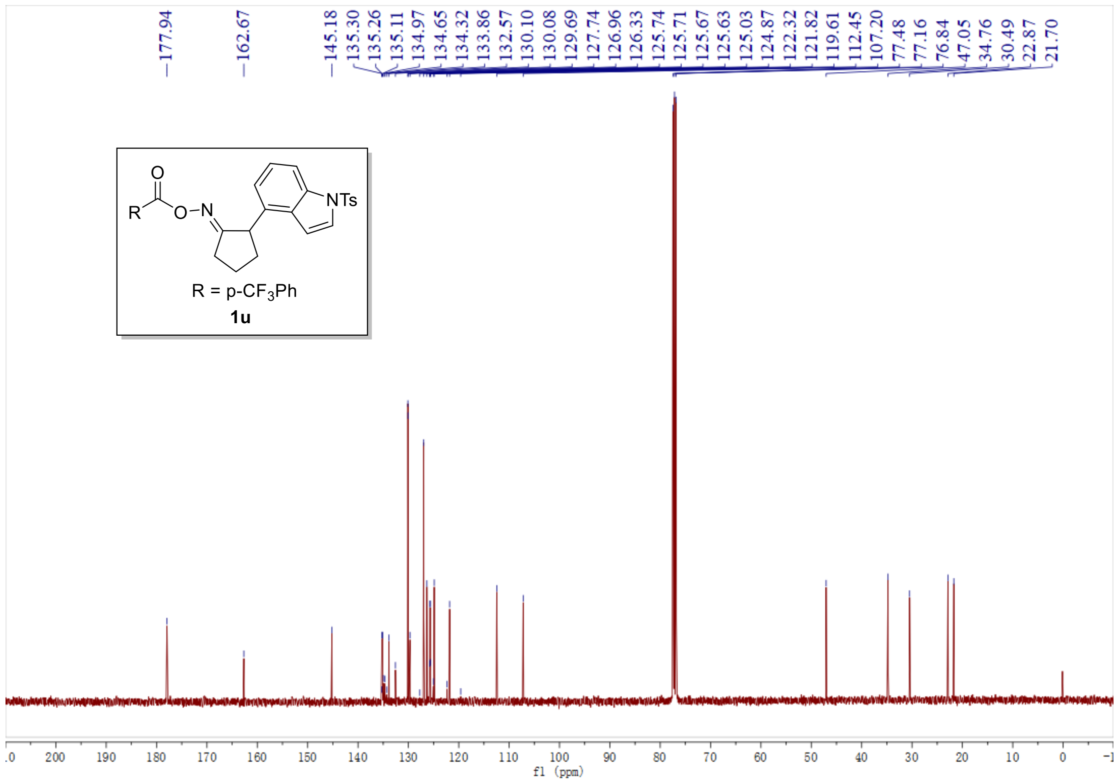

Supplementary Figure 26. ^{13}C NMR of **1u**

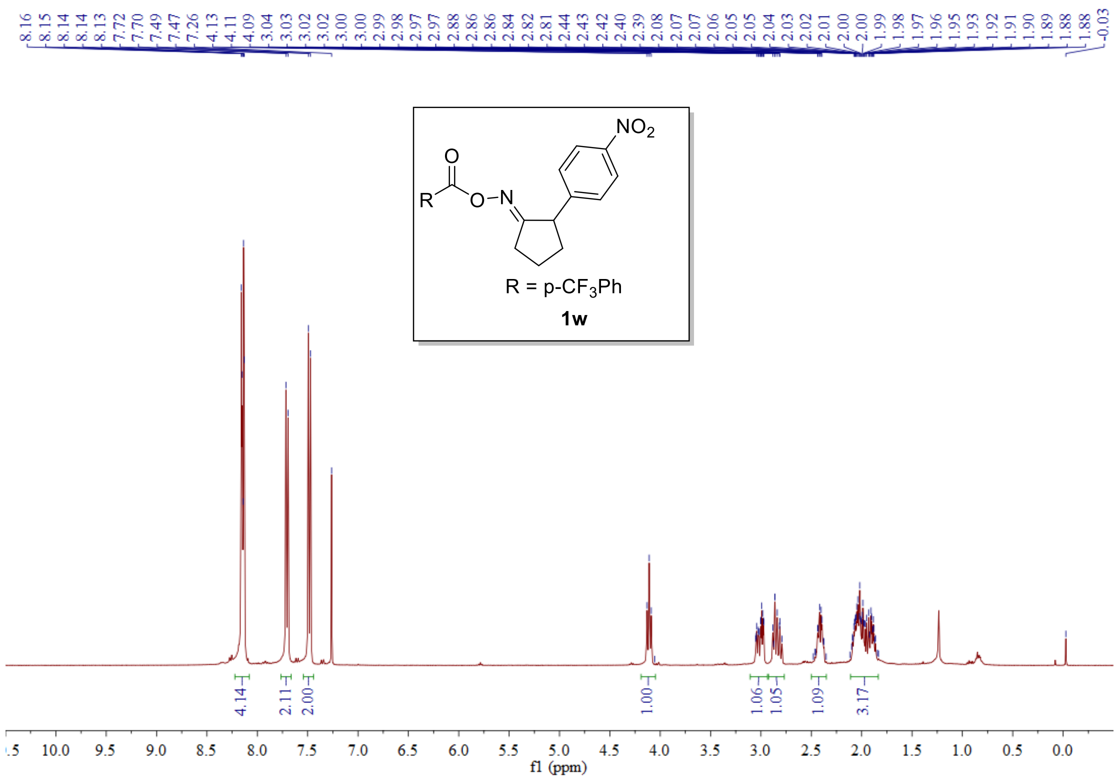

Supplementary Figure 27. ^{1}H NMR of **1w**

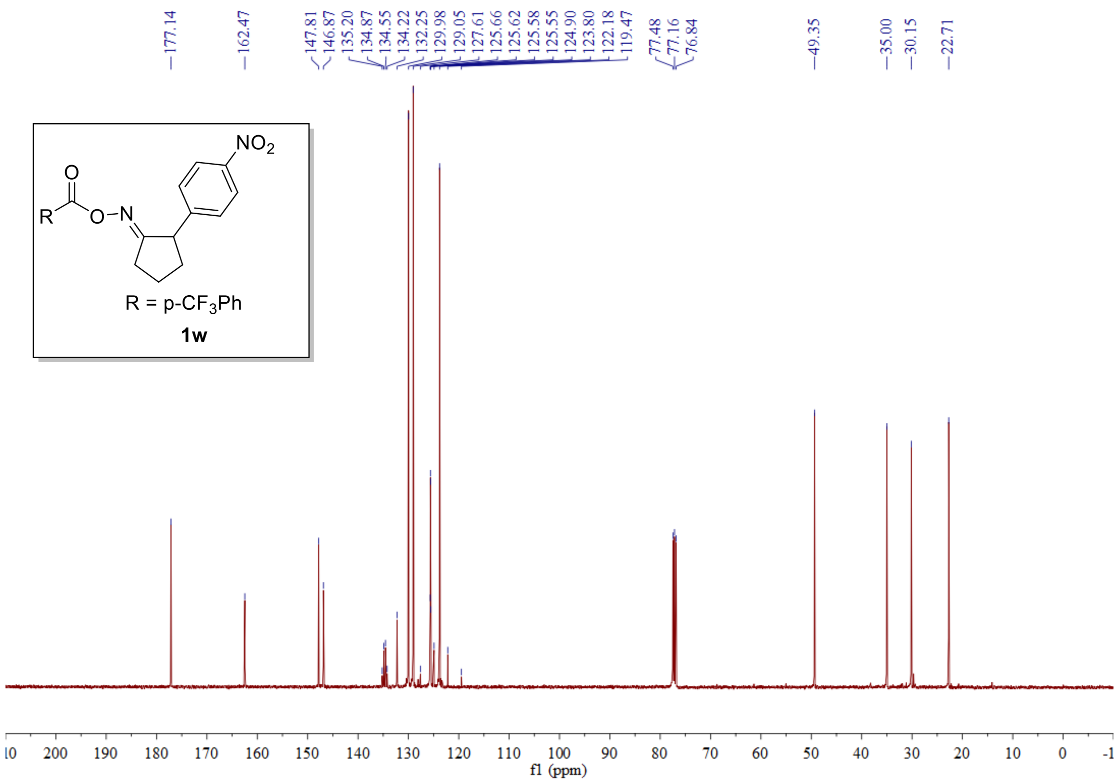

Supplementary Figure 28. ^{13}C NMR of **1w**

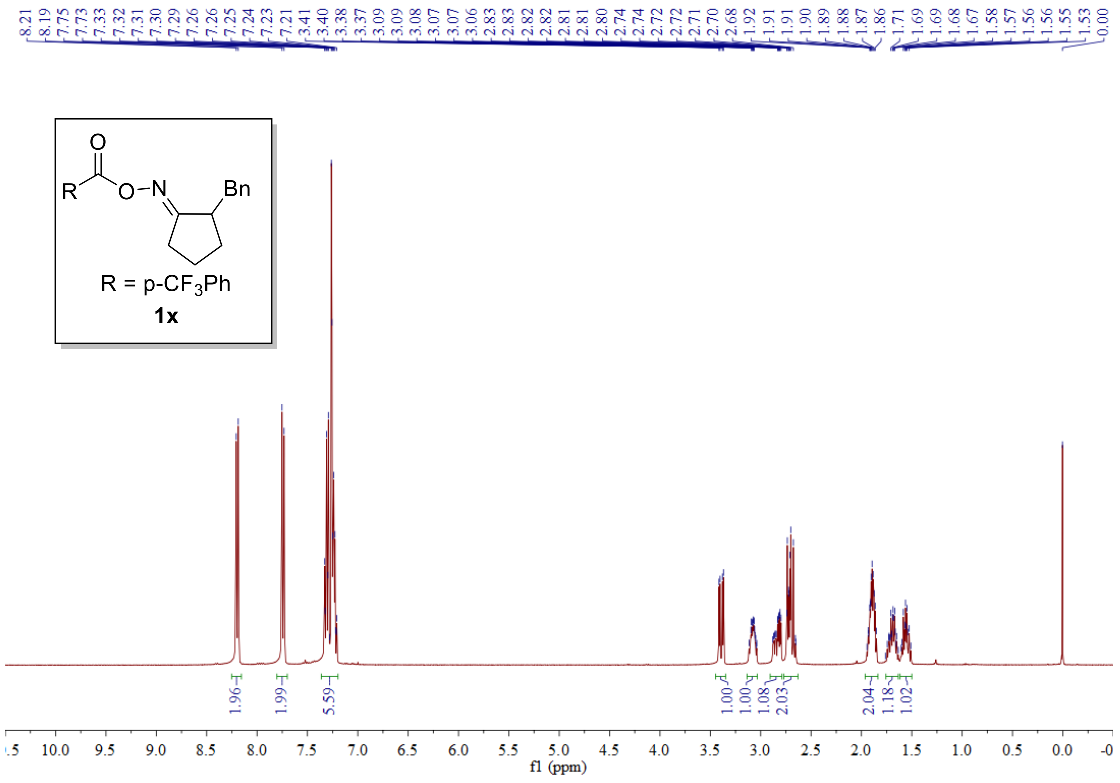

Supplementary Figure 29. ^{1}H NMR of **1x**

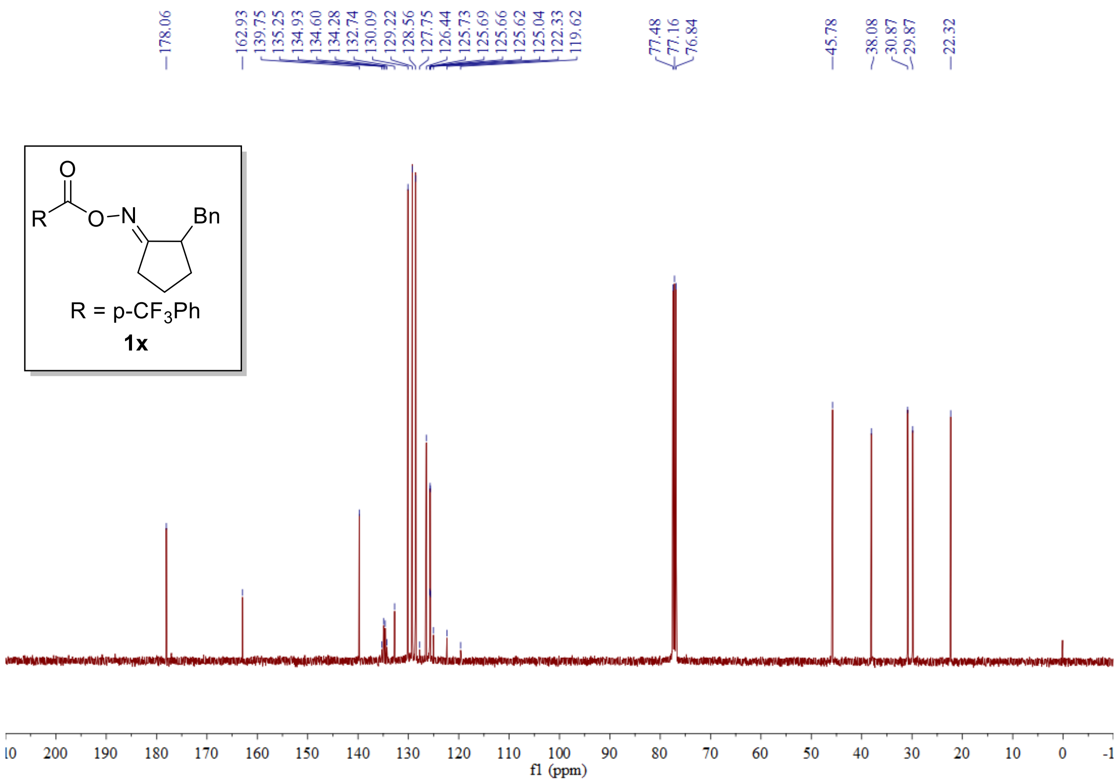

Supplementary Figure 30. ^{13}C NMR of **1x**

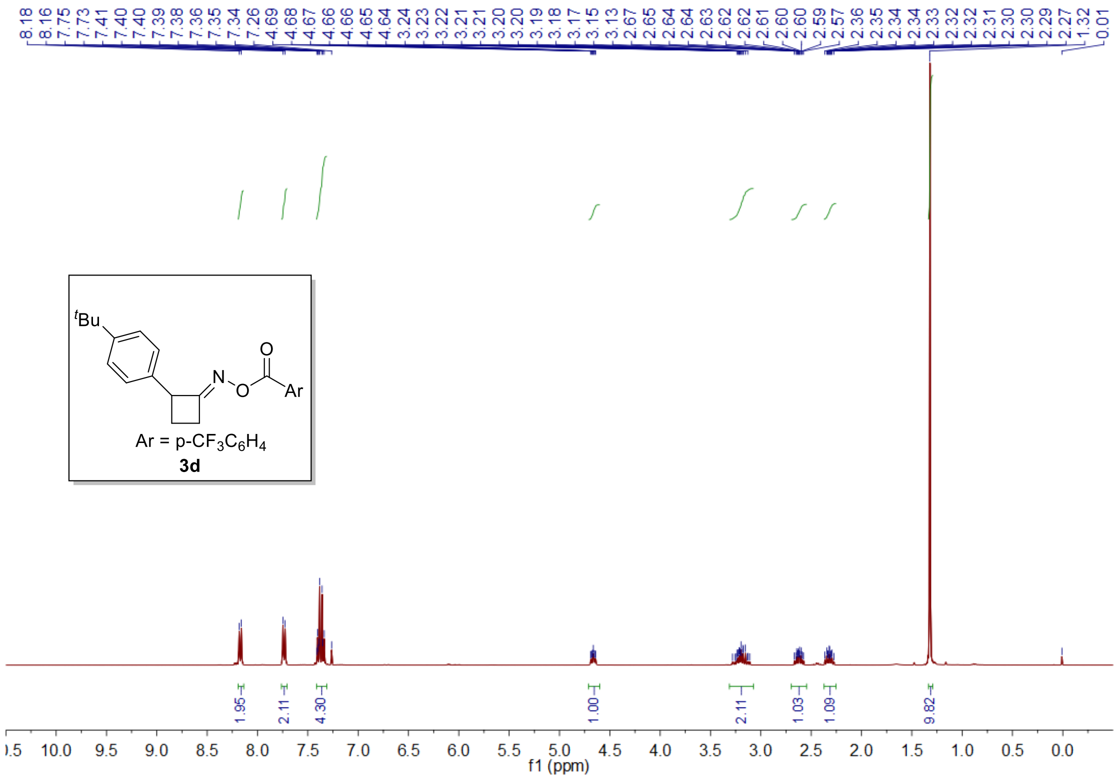

Supplementary Figure 31. ^{1}H NMR of **3d**

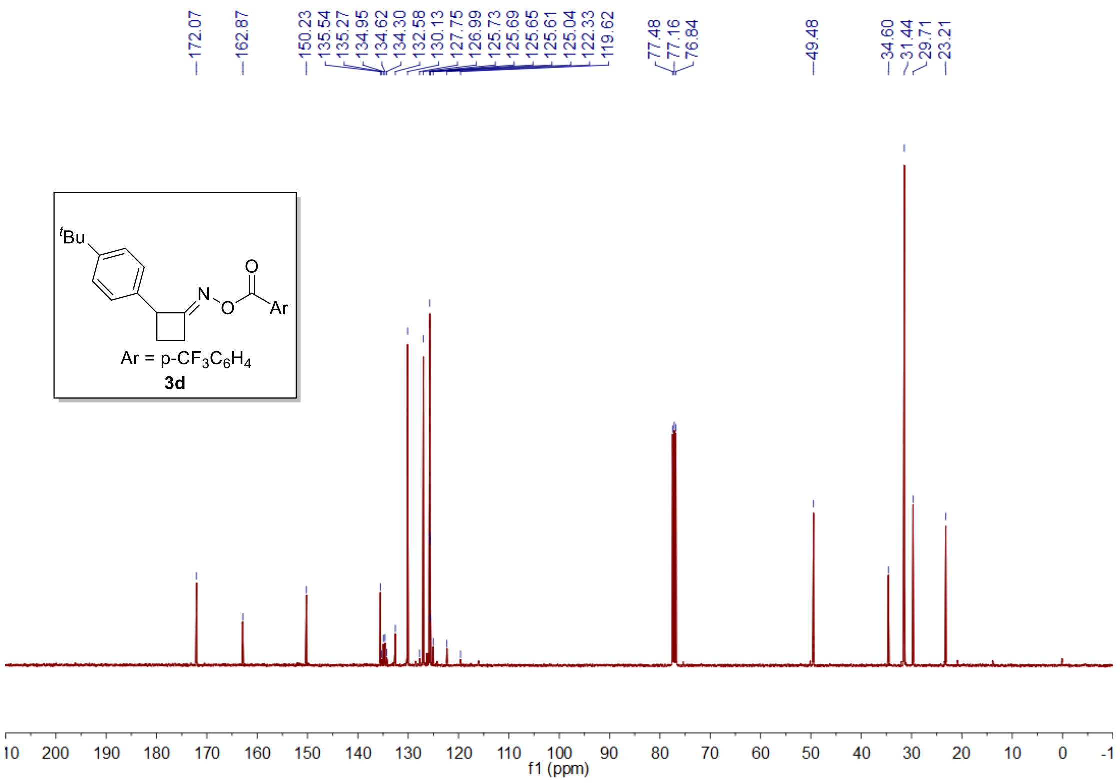

Supplementary Figure 32. ^{13}C NMR of **3d**

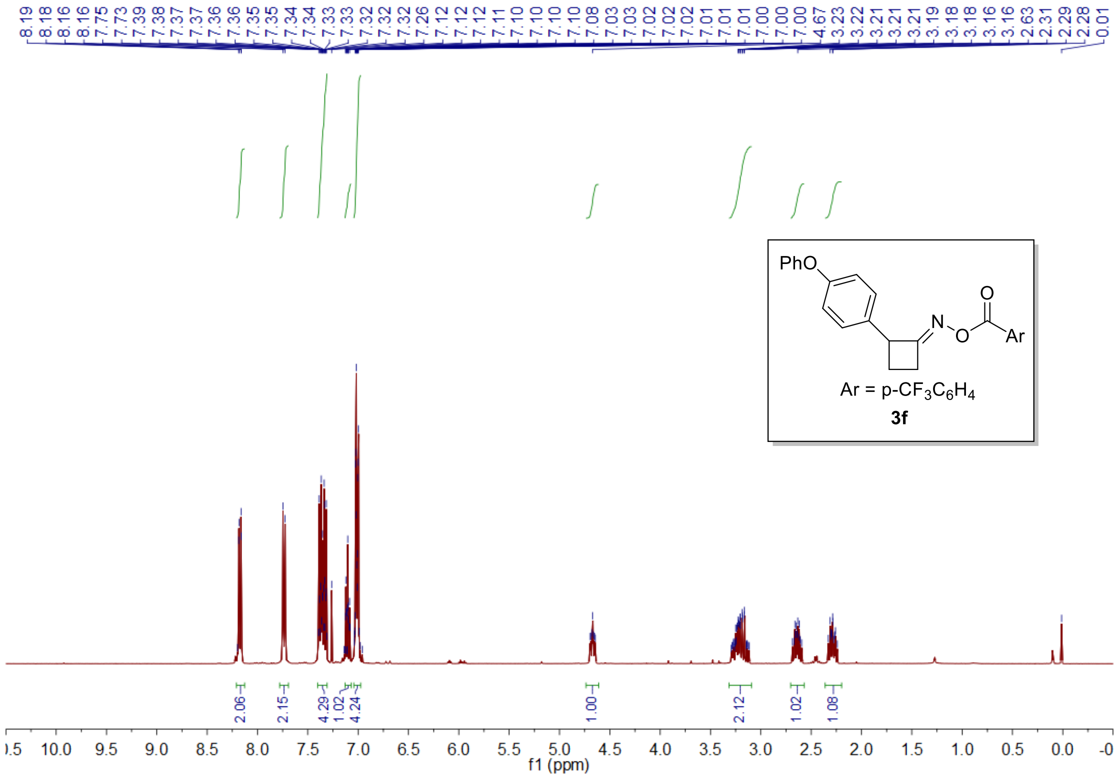

Supplementary Figure 33. ^{1}H NMR of **3f**

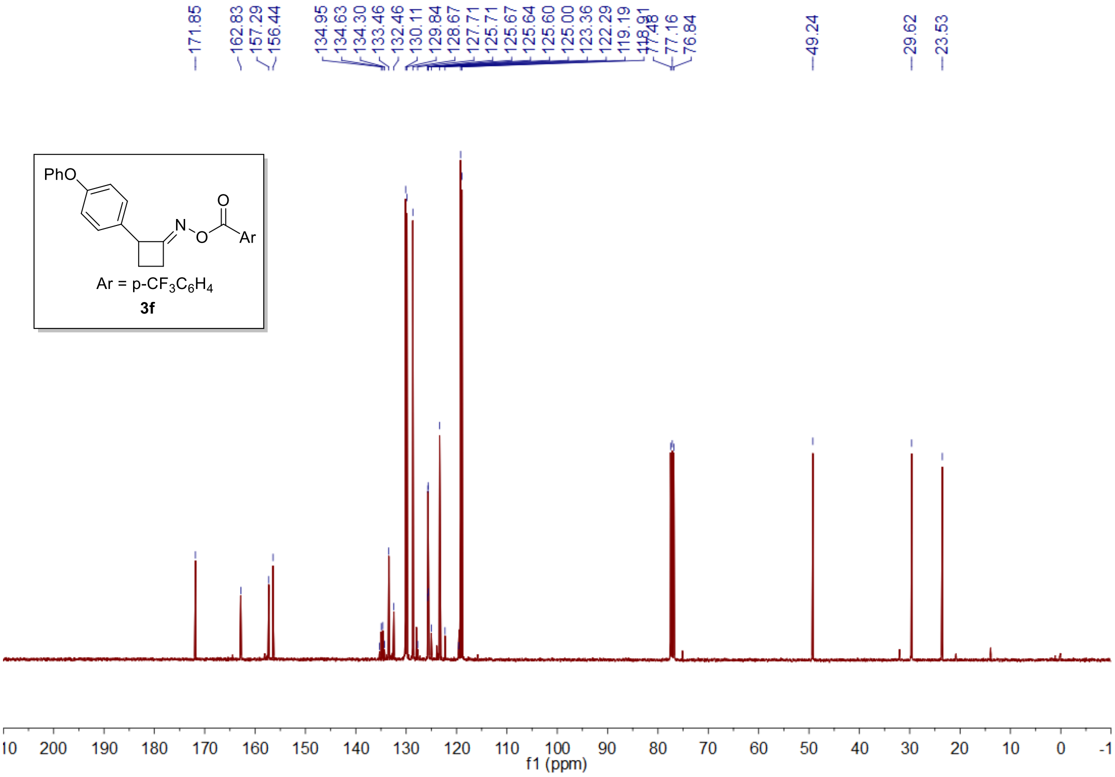

Supplementary Figure 34. ^{13}C NMR of **3f**

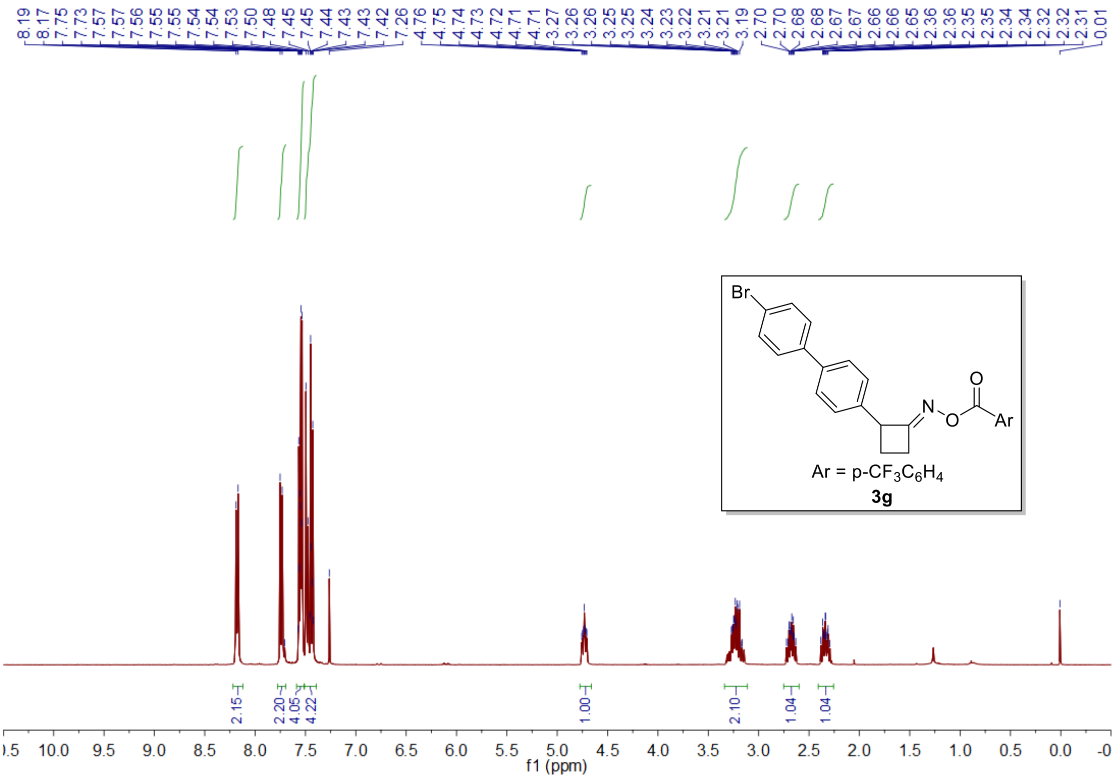

Supplementary Figure 35. ^{1}H NMR of **3g**

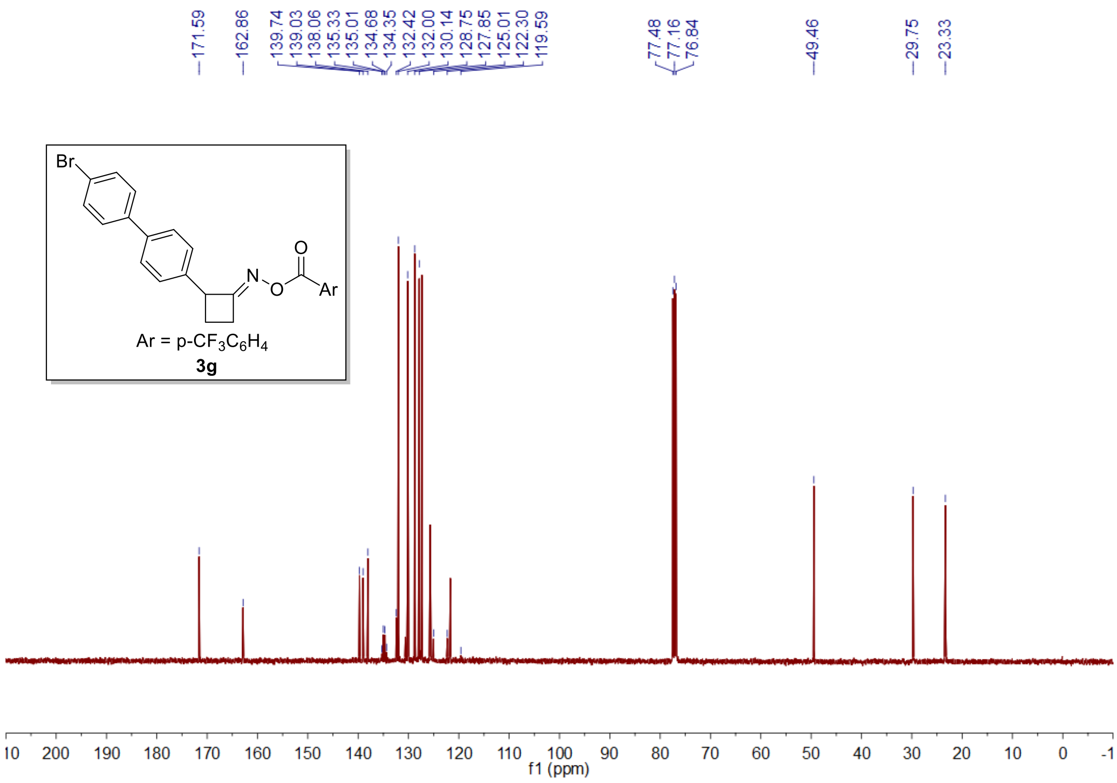

Supplementary Figure 36. ^{13}C NMR of **3g**

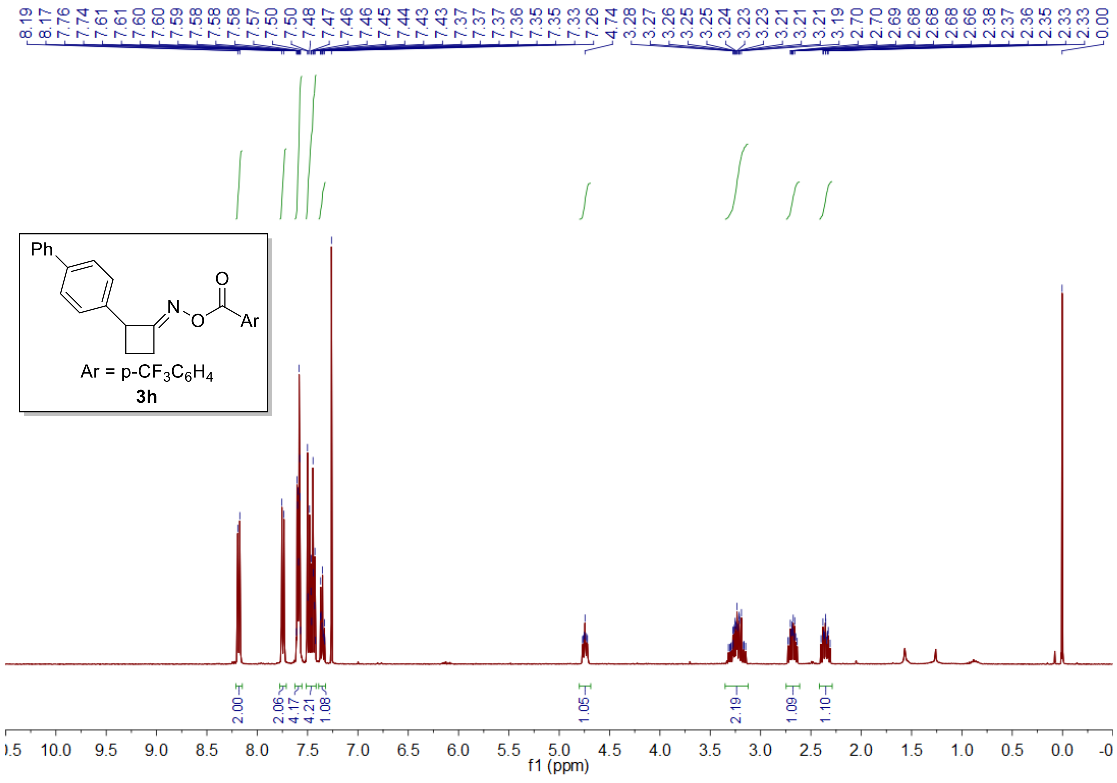

Supplementary Figure 37. ^{1}H NMR of **3h**

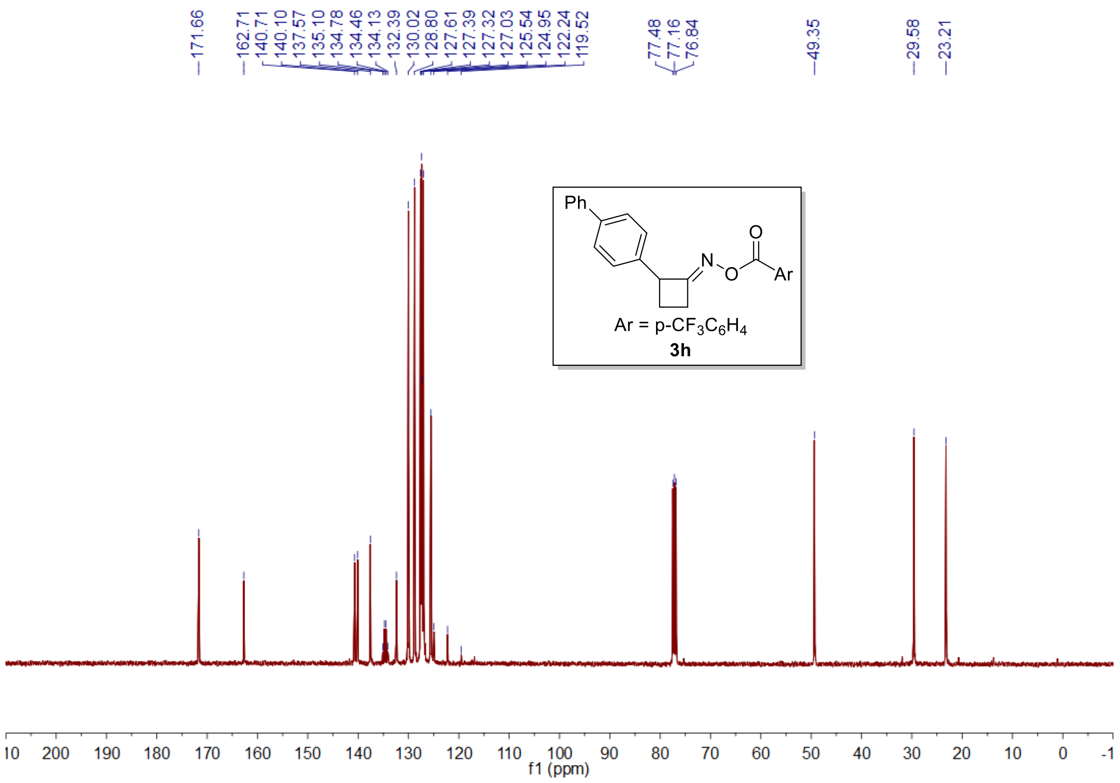

Supplementary Figure 38. ^{13}C NMR of **3h**

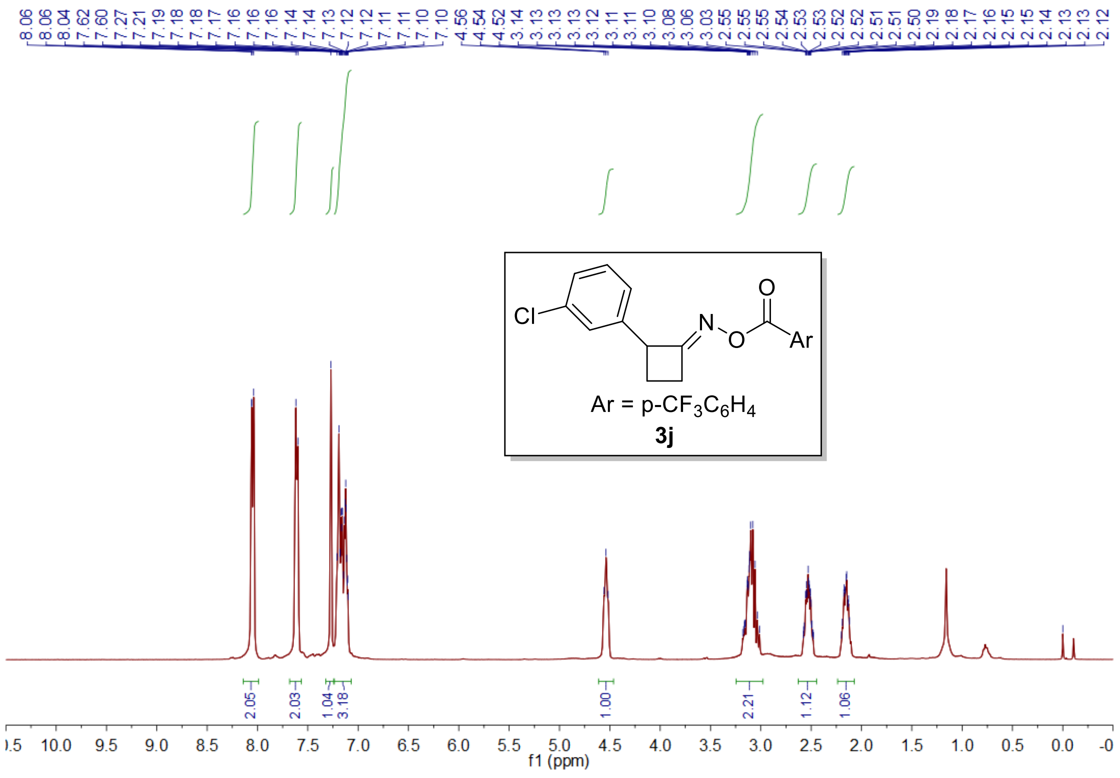

Supplementary Figure 39. ^{1}H NMR of **3j**

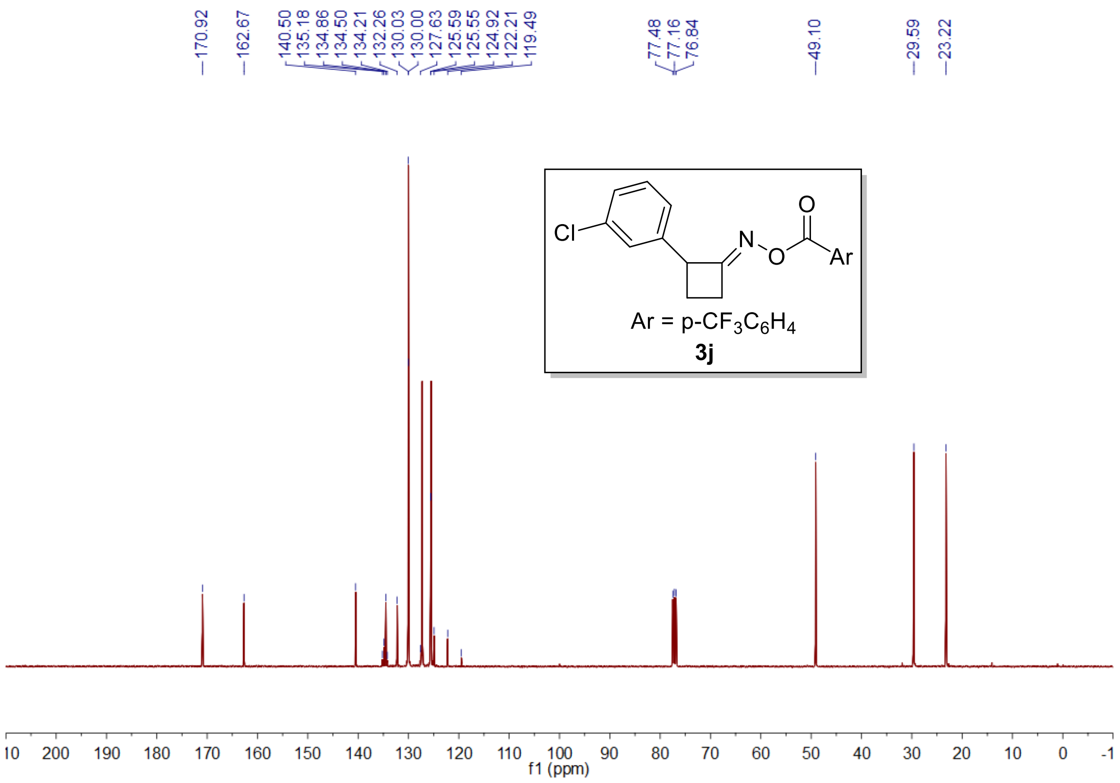

Supplementary Figure 40. ^{13}C NMR of **3j**

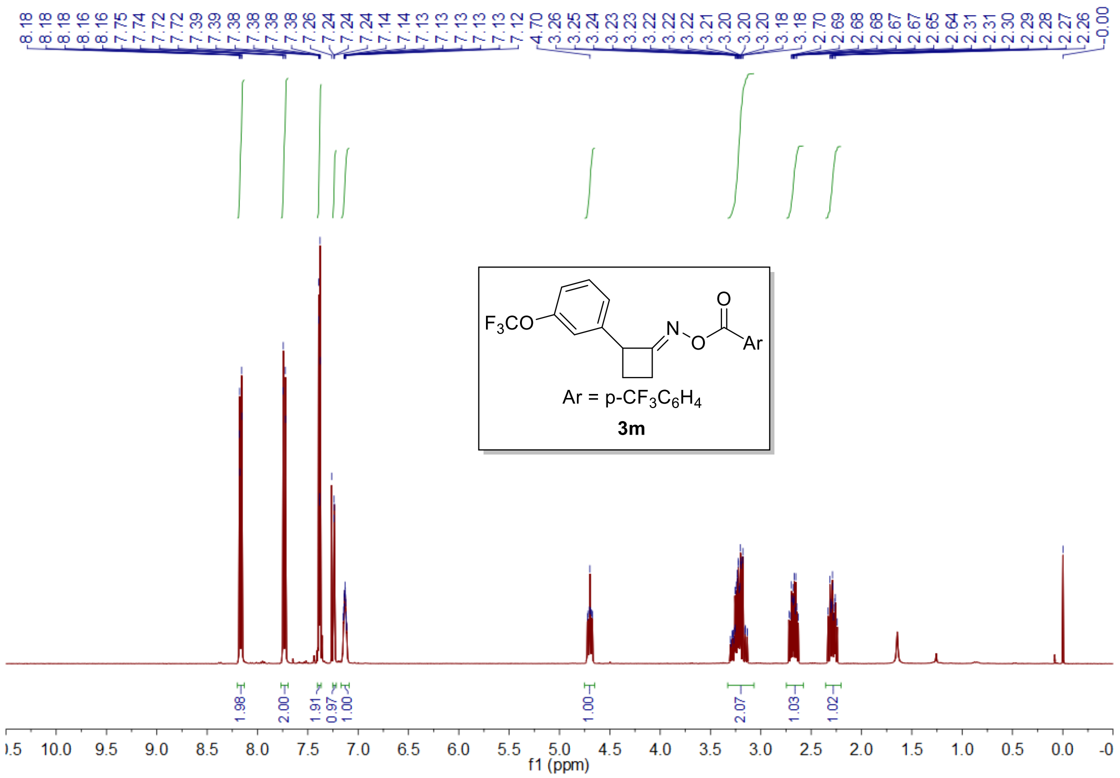

Supplementary Figure 41. ^{1}H NMR of **3m**

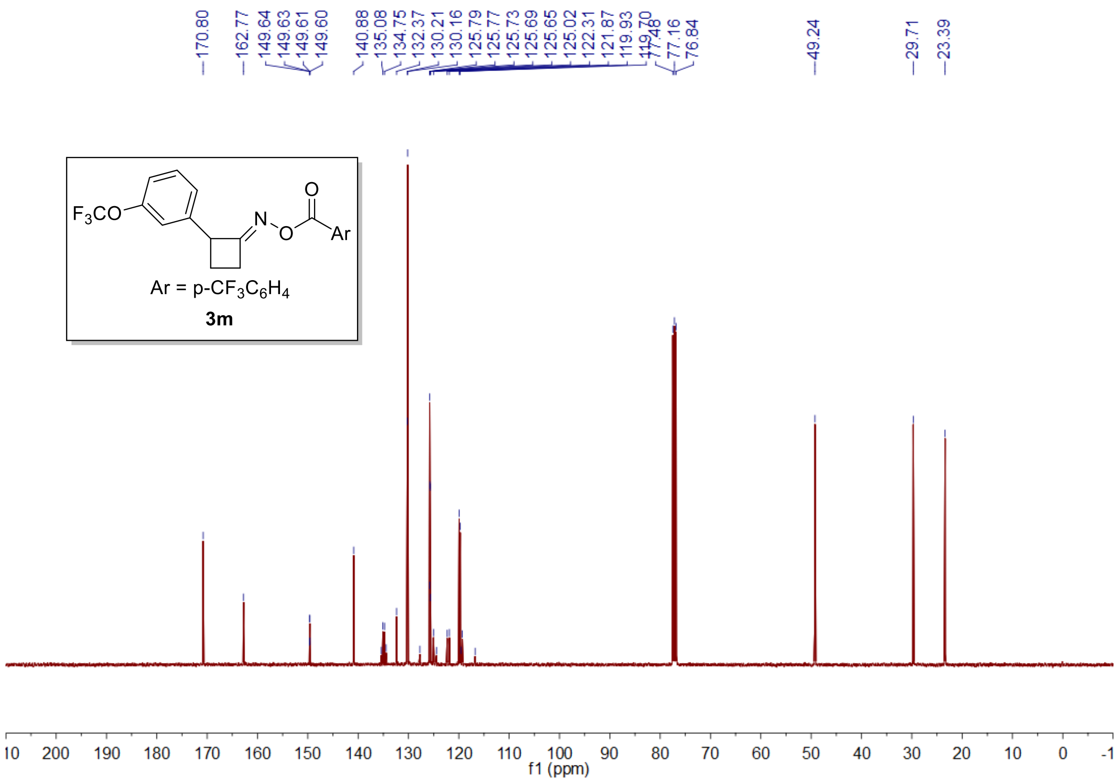

Supplementary Figure 42. ^{13}C NMR of **3m**

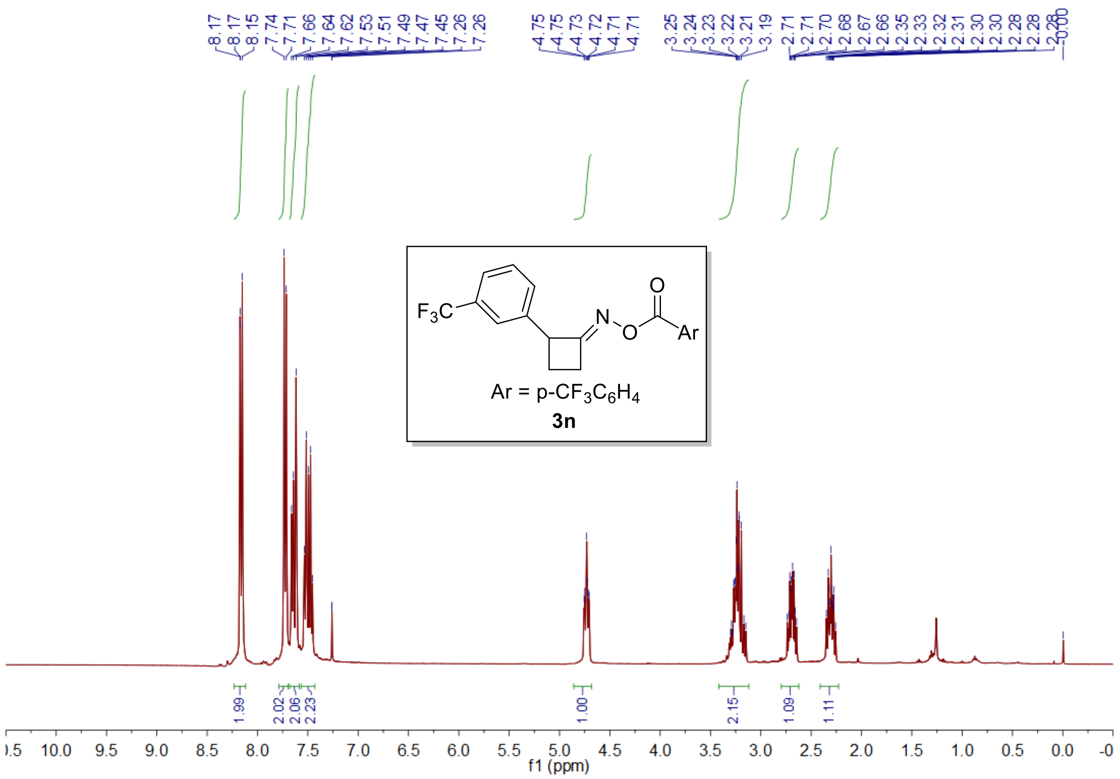

Supplementary Figure 43. ^{1}H NMR of **3n**

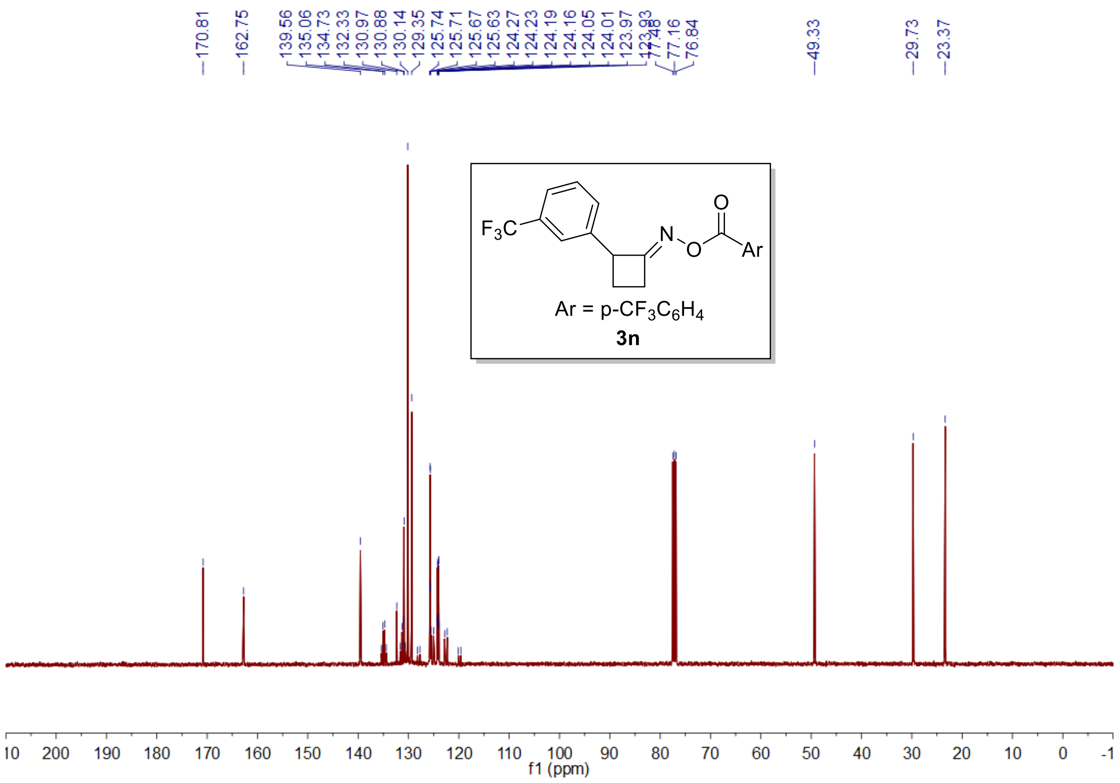

Supplementary Figure 44. ^{13}C NMR of **3n**

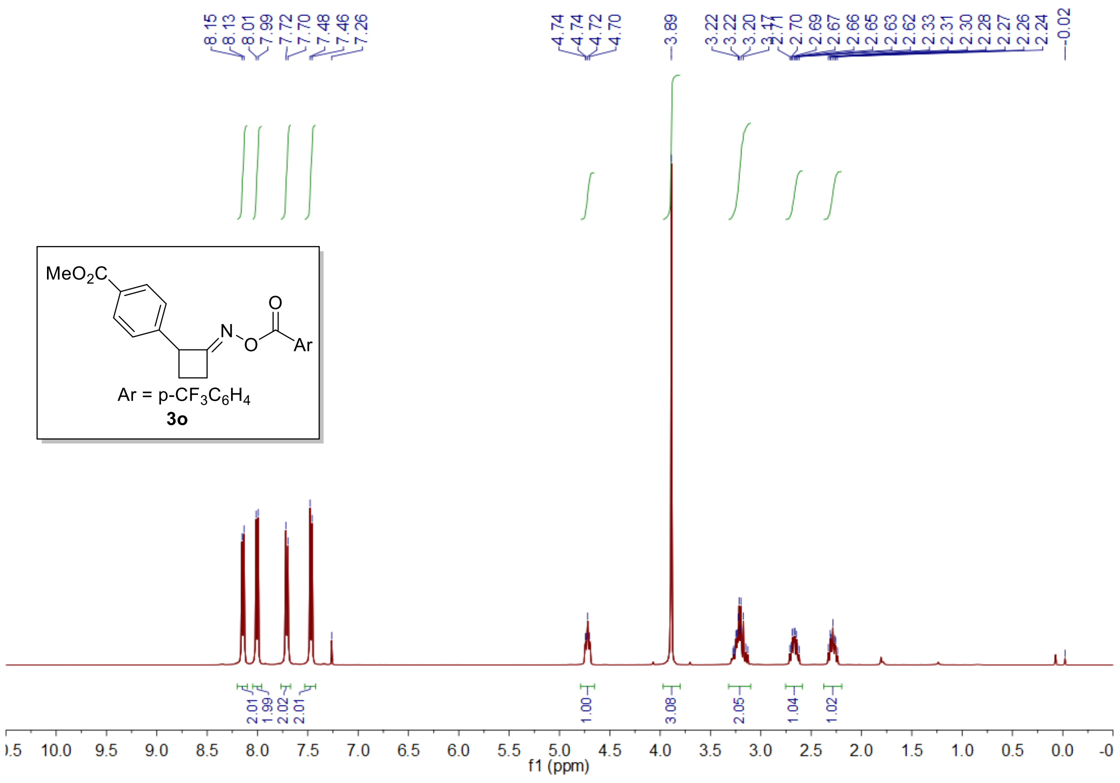

Supplementary Figure 45. ^{1}H NMR of **3o**

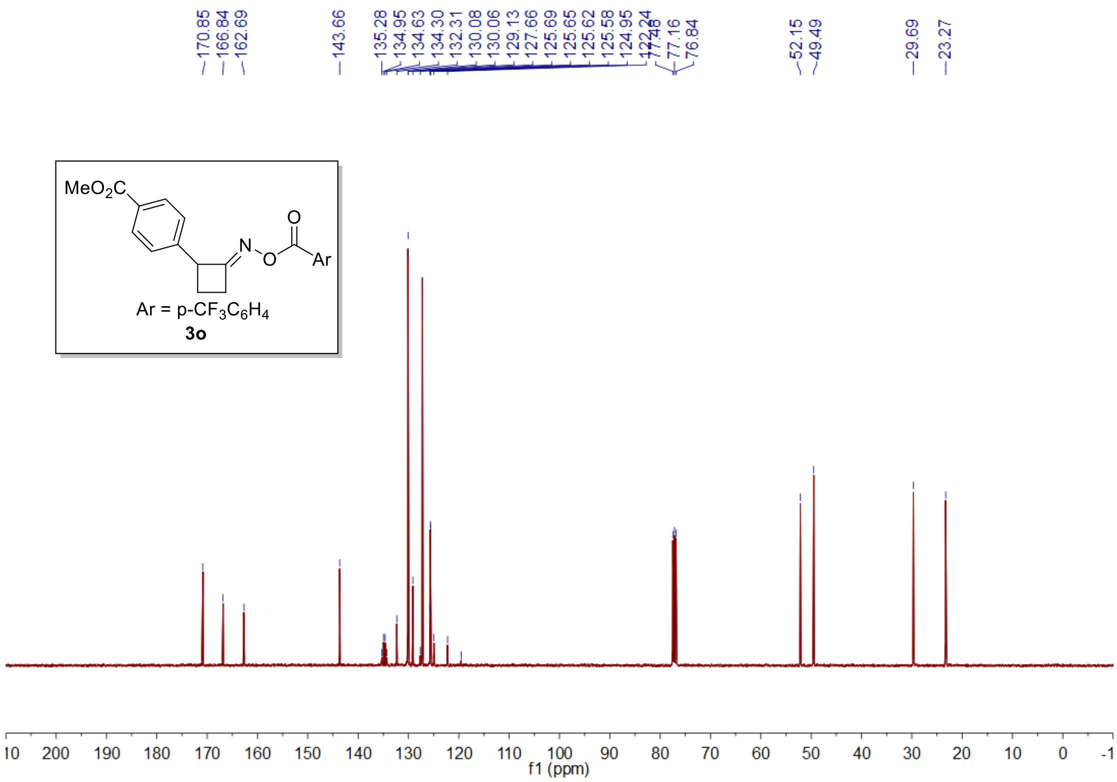

Supplementary Figure 46. ^{13}C NMR of **3o**

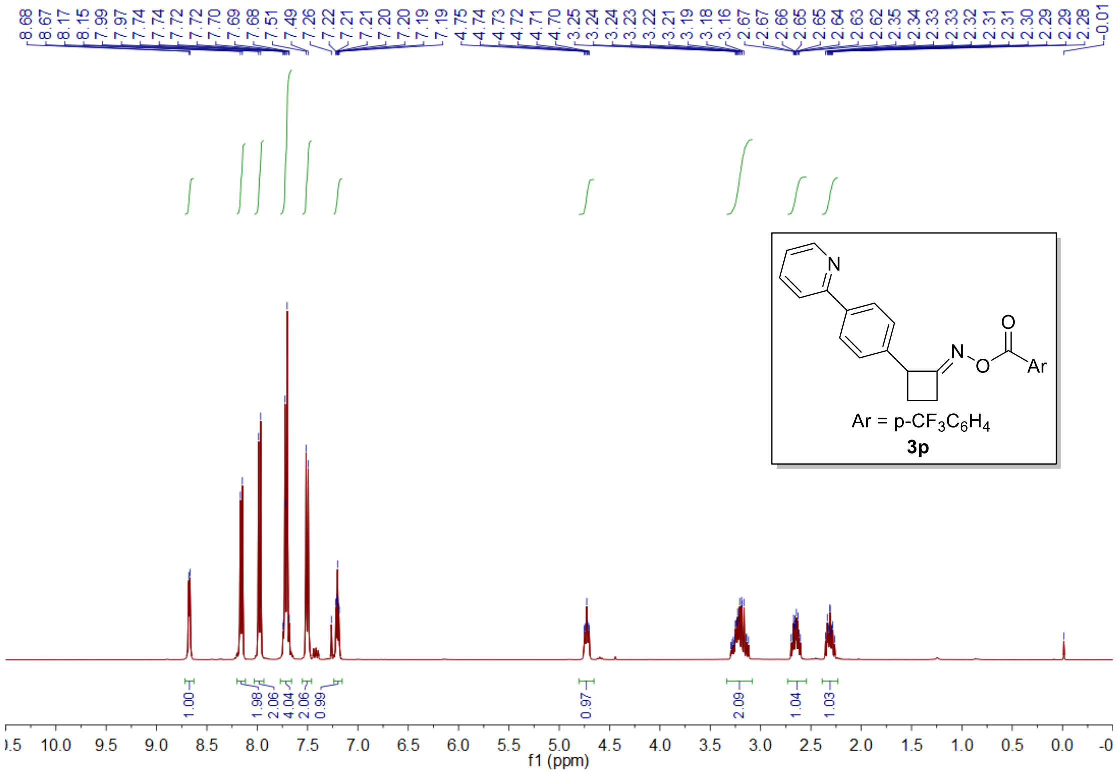

Supplementary Figure 47. ^{1}H NMR of **3p**

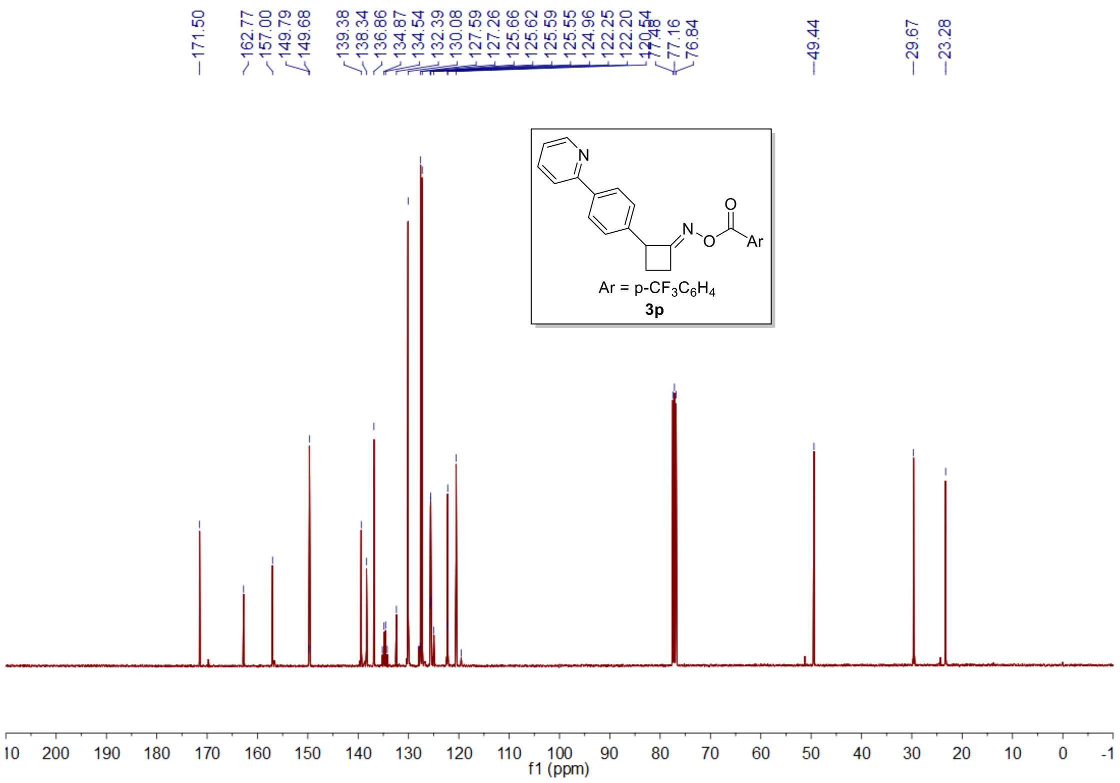

Supplementary Figure 48. ^{13}C NMR of **3p**

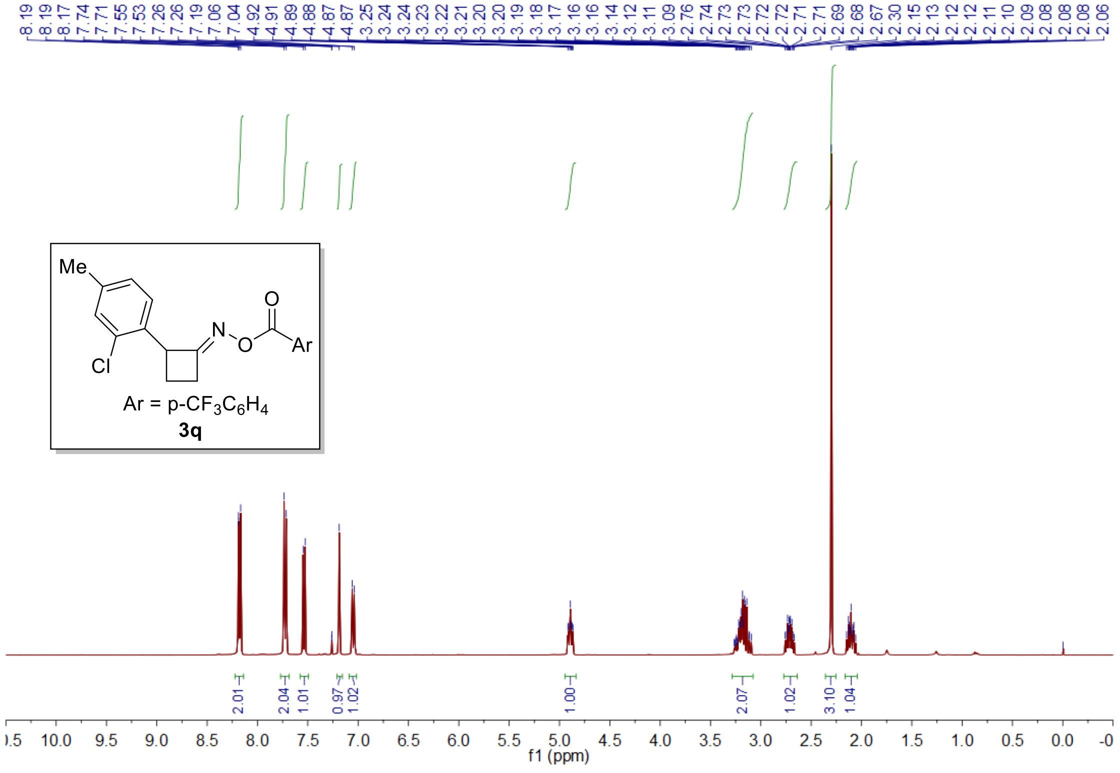

Supplementary Figure 49. ^{1}H NMR of **3q**

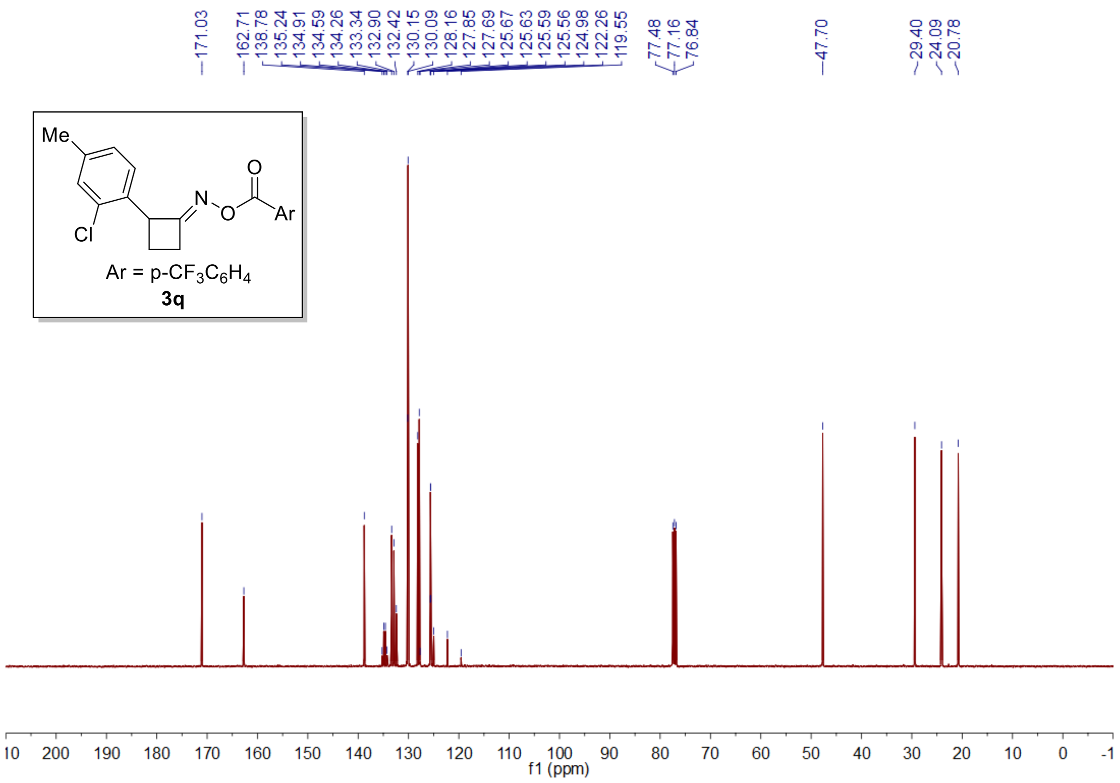

Supplementary Figure 50. ^{13}C NMR of **3q**

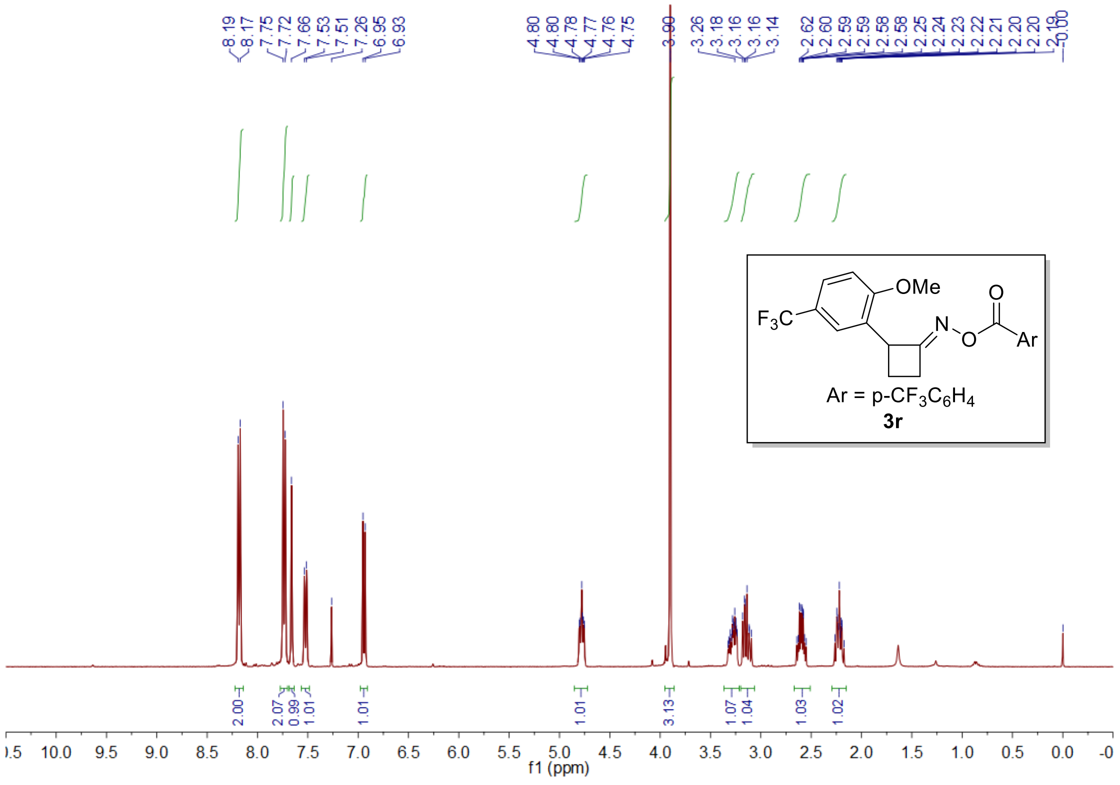

Supplementary Figure 51. ^{1}H NMR of **3r**

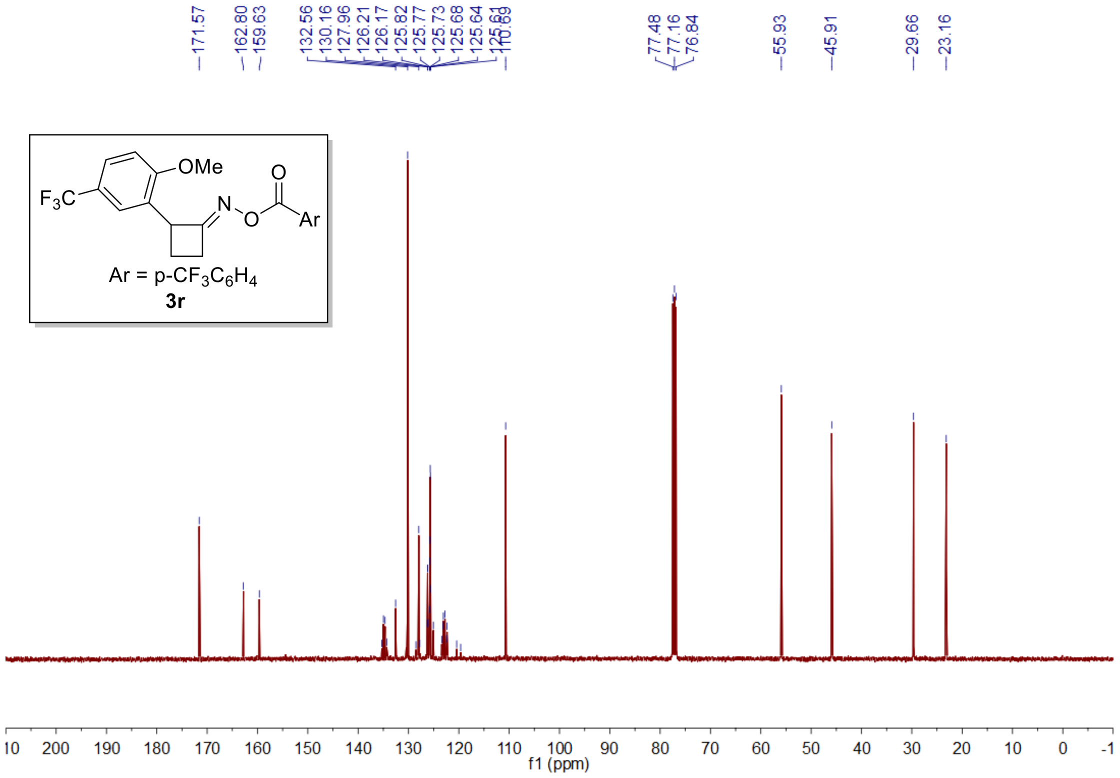

Supplementary Figure 52. ^{13}C NMR of **3r**

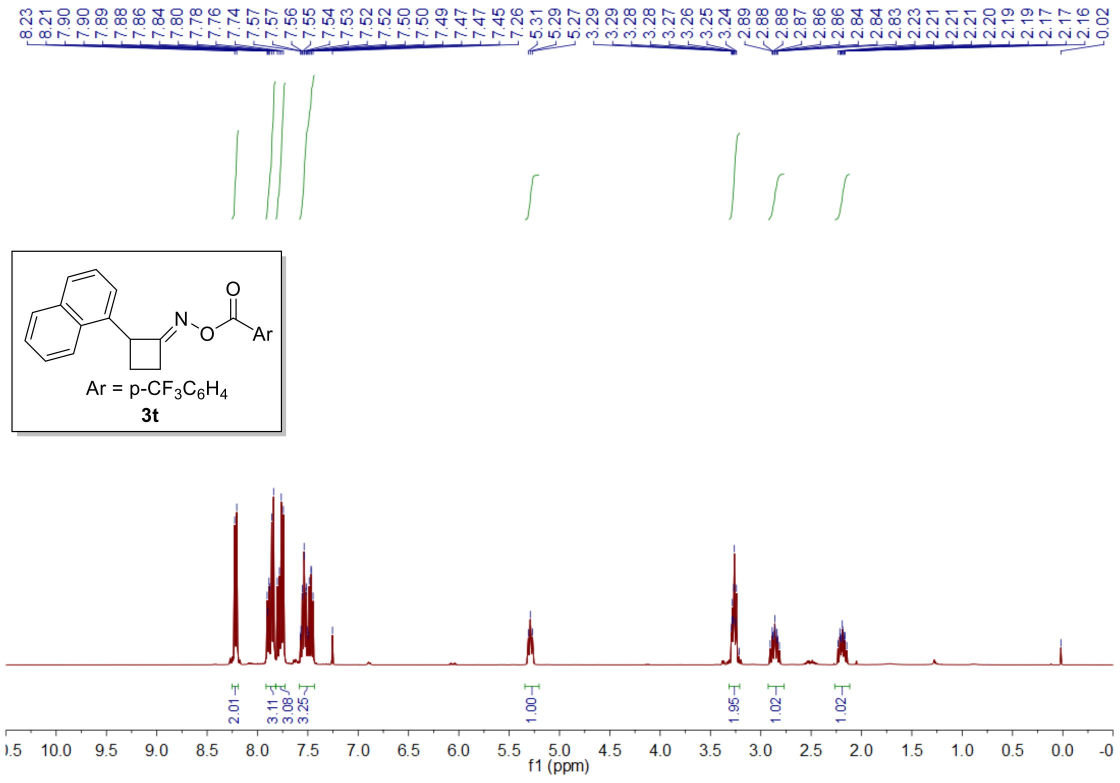

Supplementary Figure 53. ^{1}H NMR of **3t**

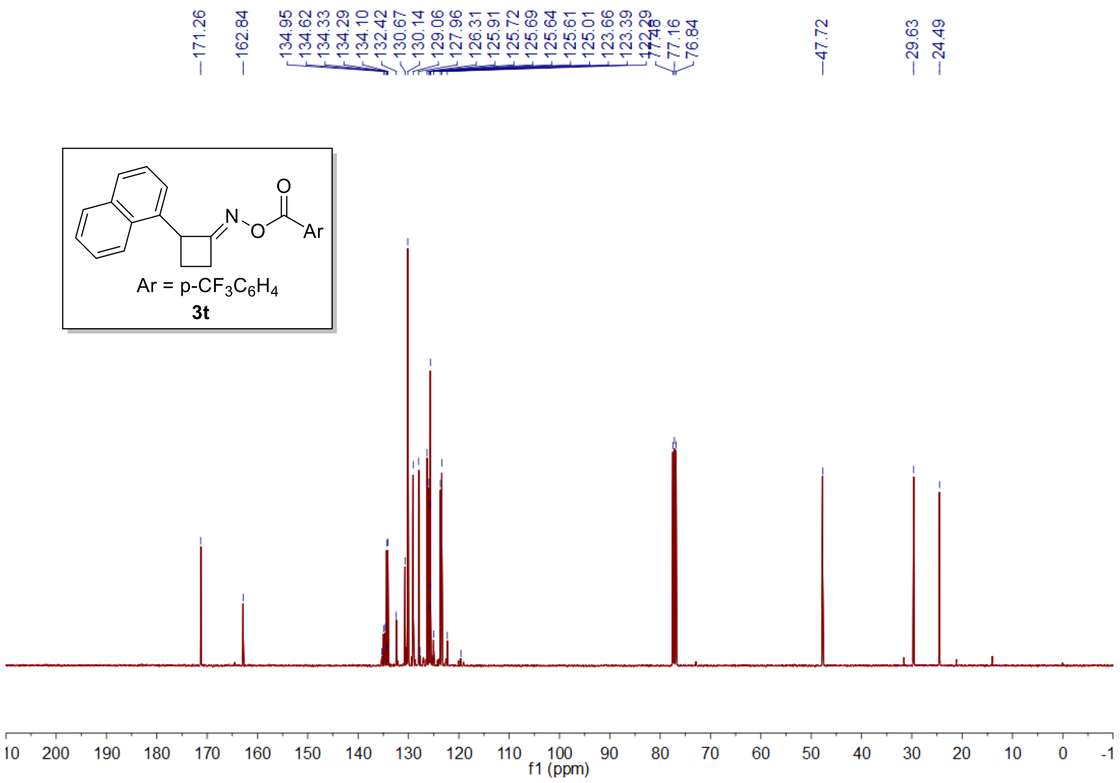

Supplementary Figure 54. ^{13}C NMR of **3t**

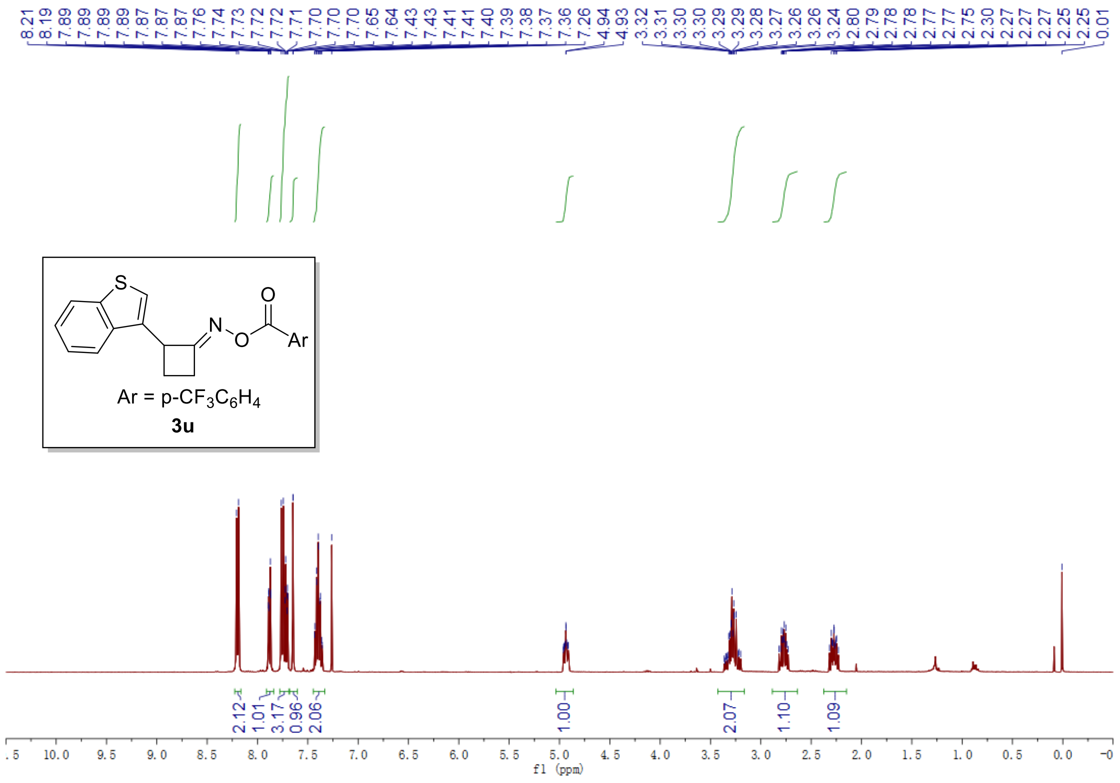

Supplementary Figure 55. ^{1}H NMR of **3u**

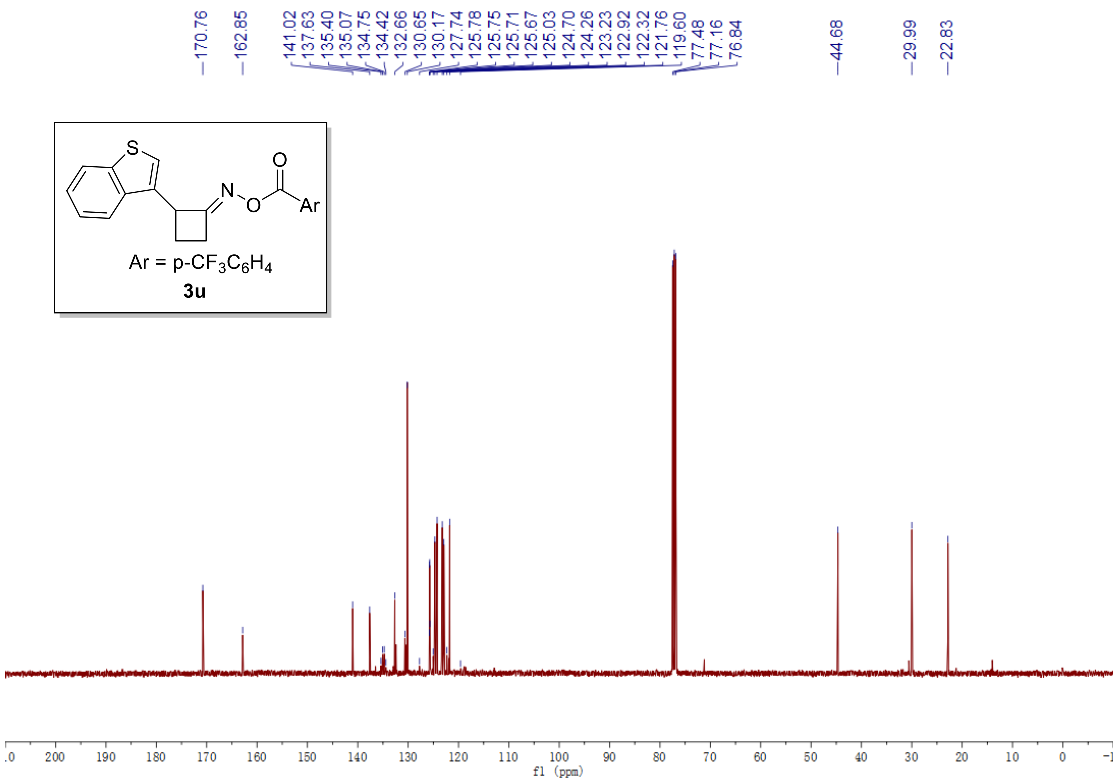

Supplementary Figure 56. ^{13}C NMR of **3u**

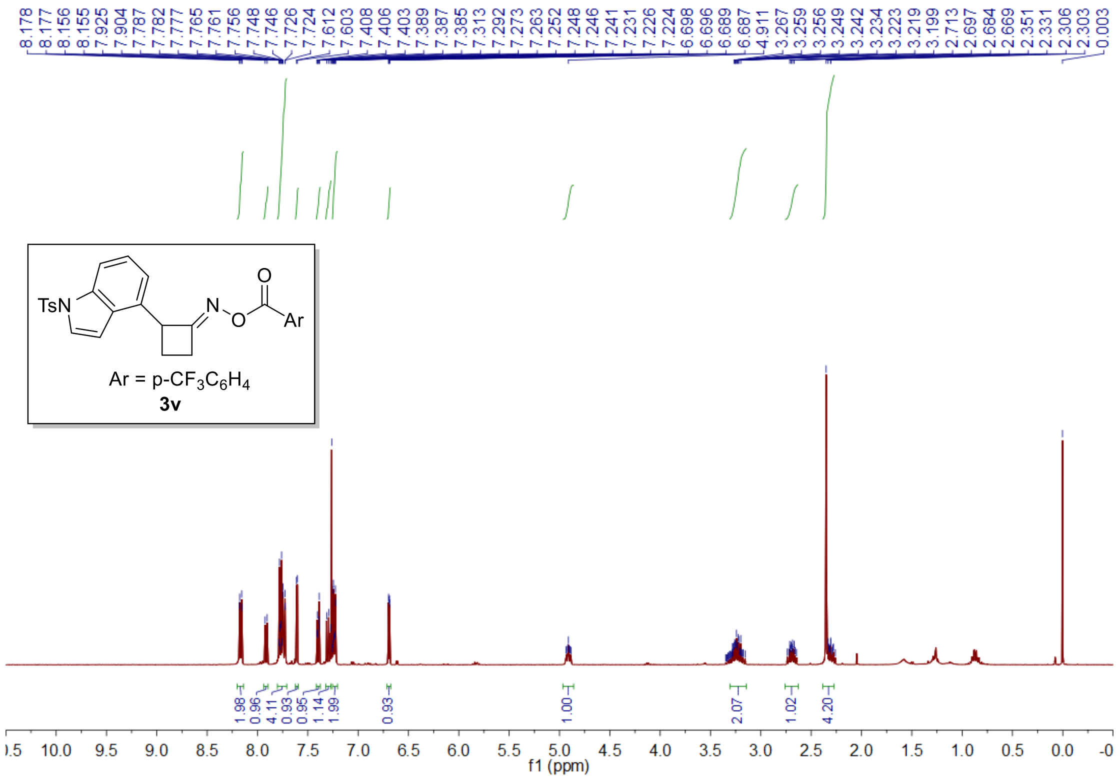

Supplementary Figure 57. ^{1}H NMR of **3v**

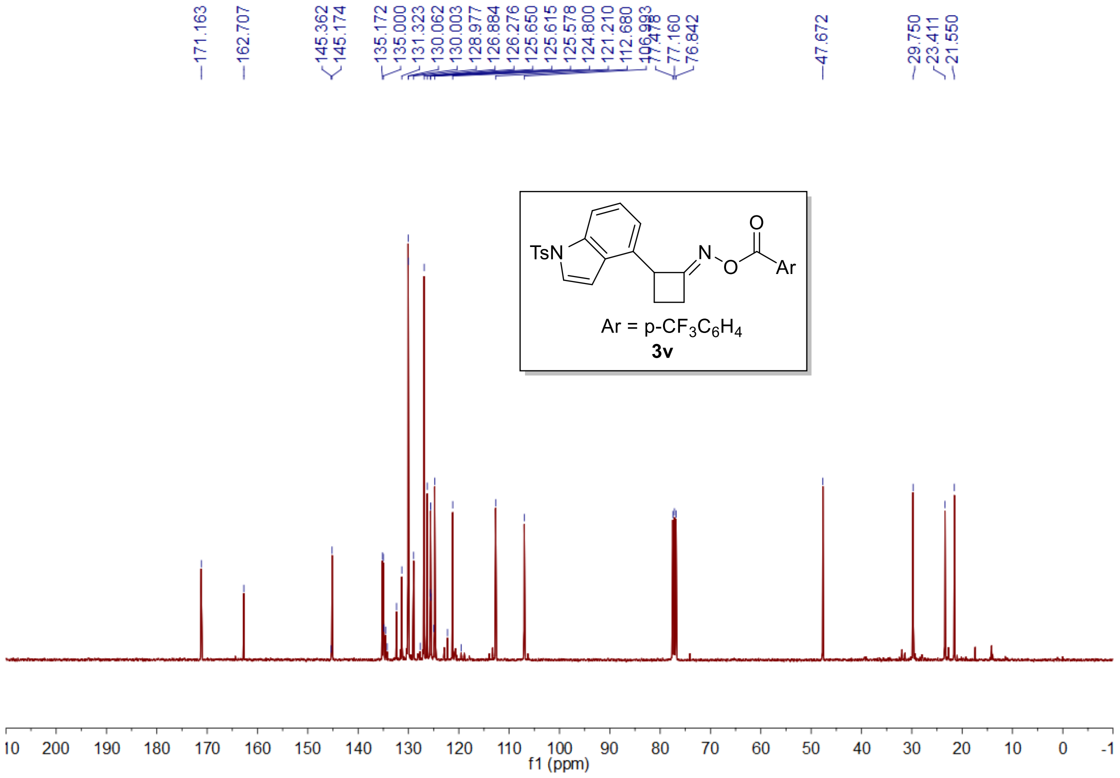

Supplementary Figure 58. ^{13}C NMR of **3v**

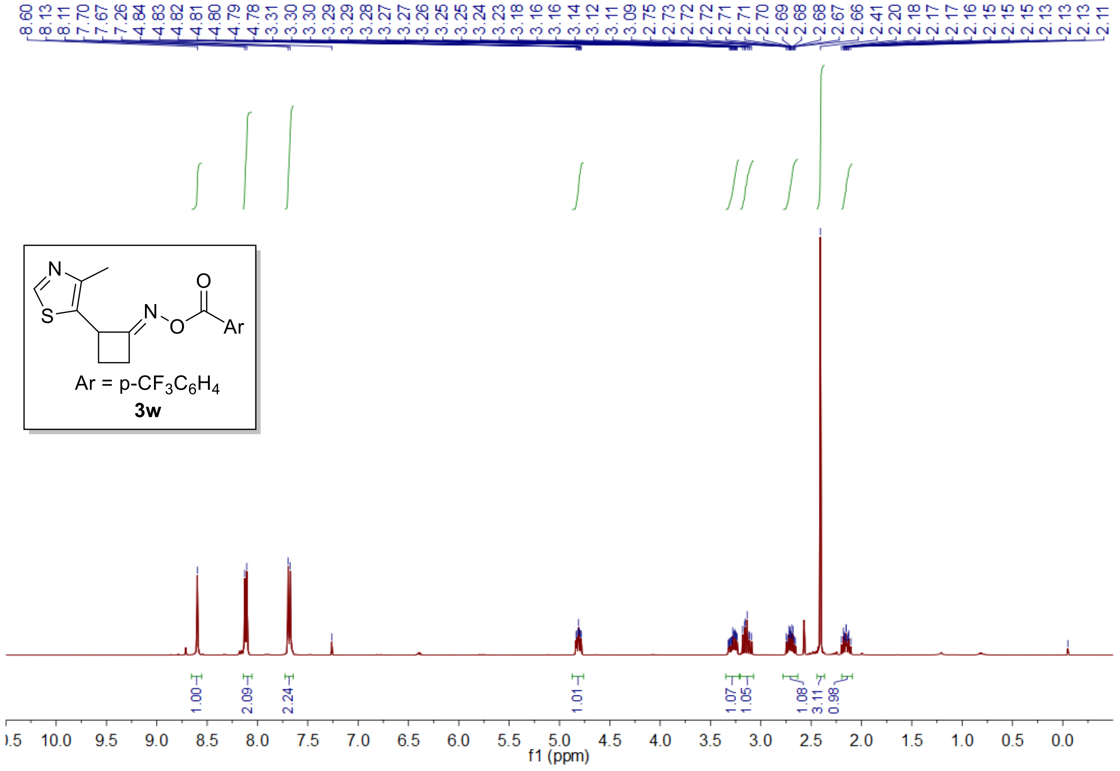

Supplementary Figure 59. ^{1}H NMR of **3w**

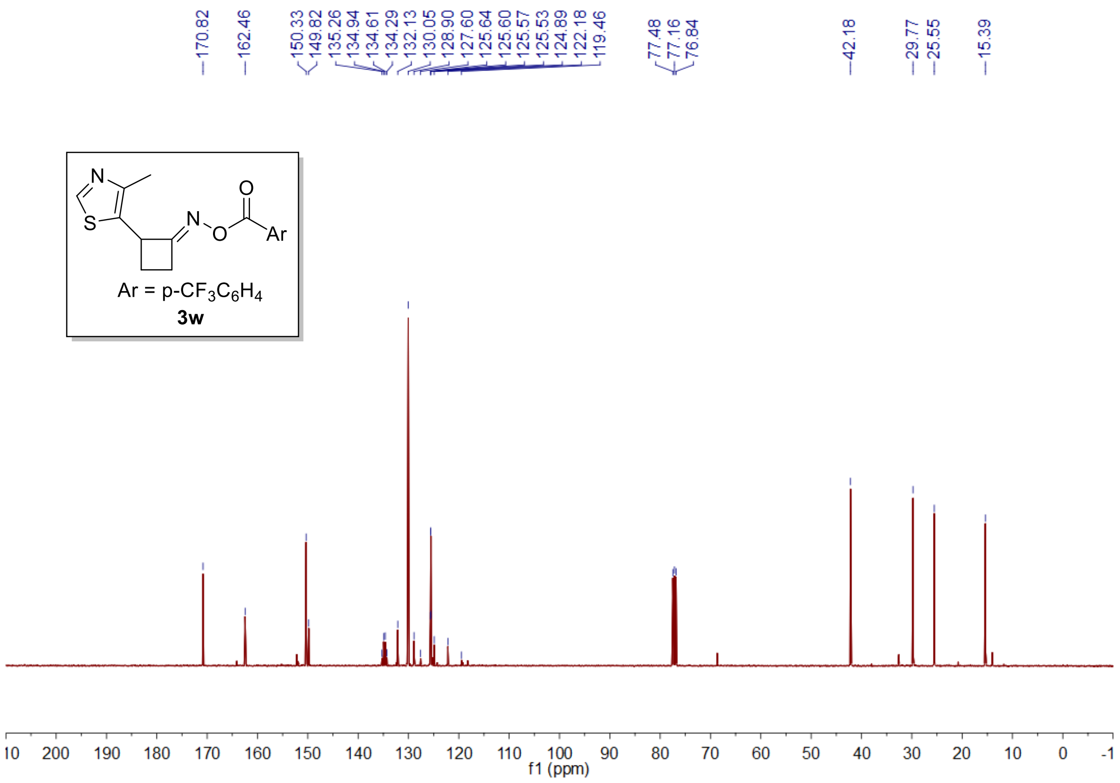

Supplementary Figure 60. ^{13}C NMR of **3w**

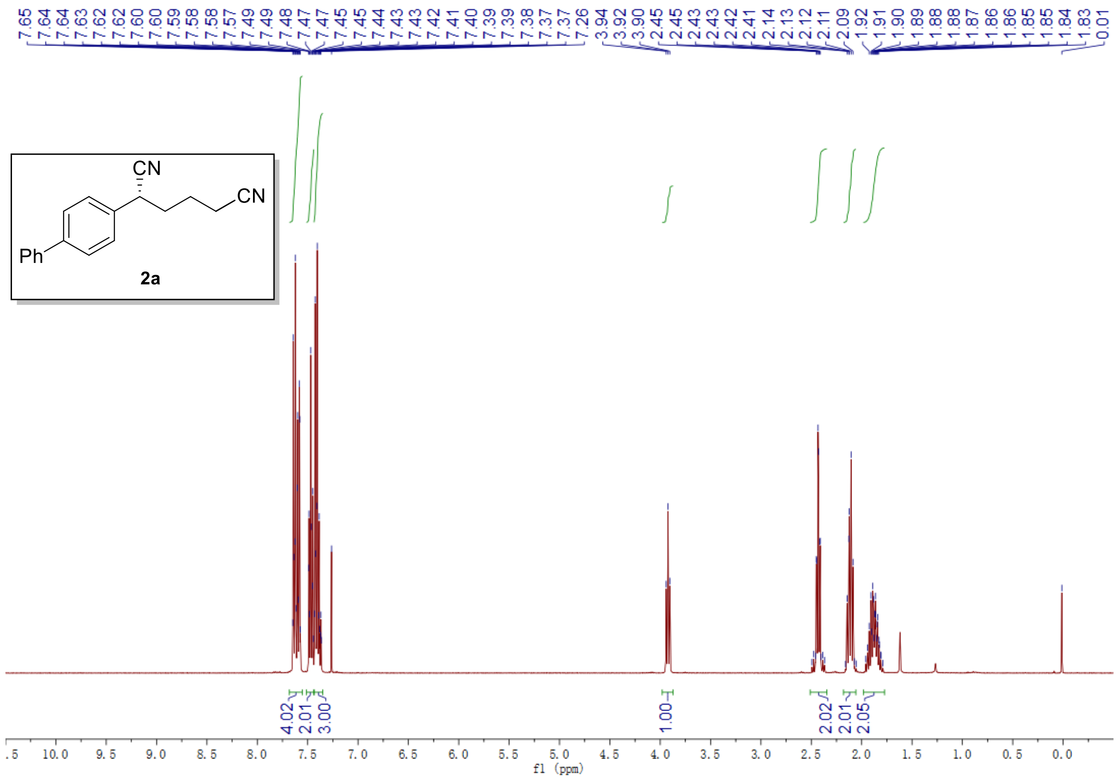

Supplementary Figure 61. ^{1}H NMR of **2a**

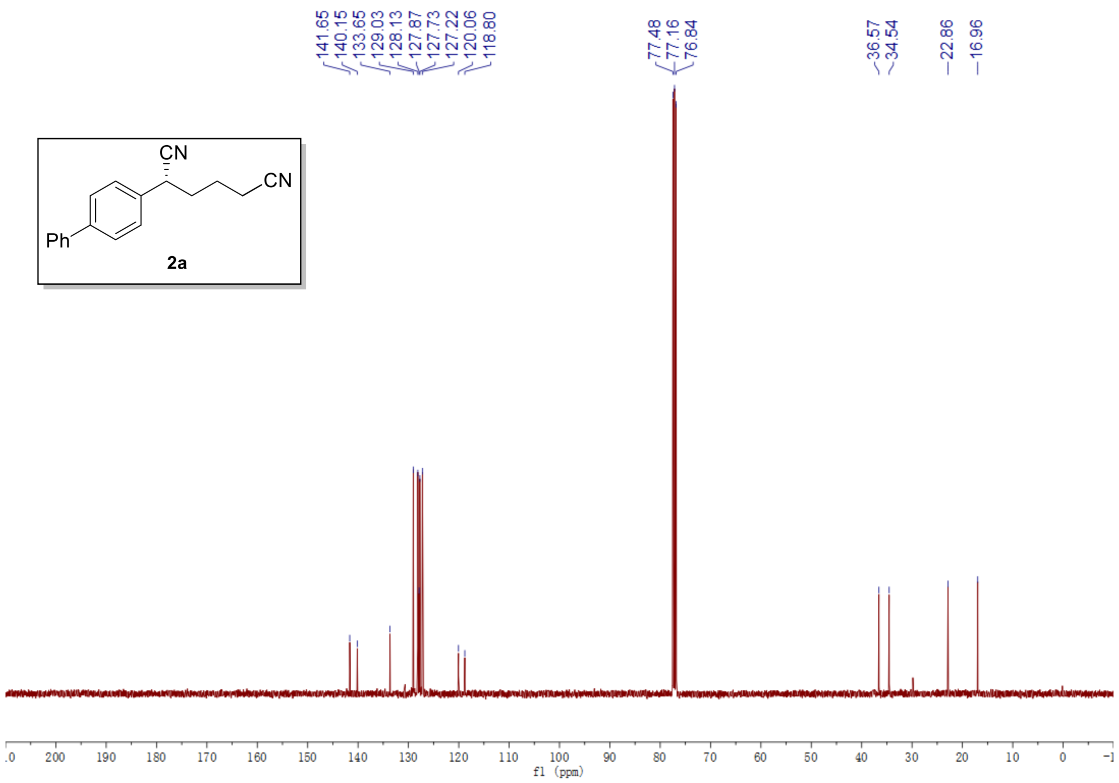

Supplementary Figure 62. ^{13}C NMR of **2a**

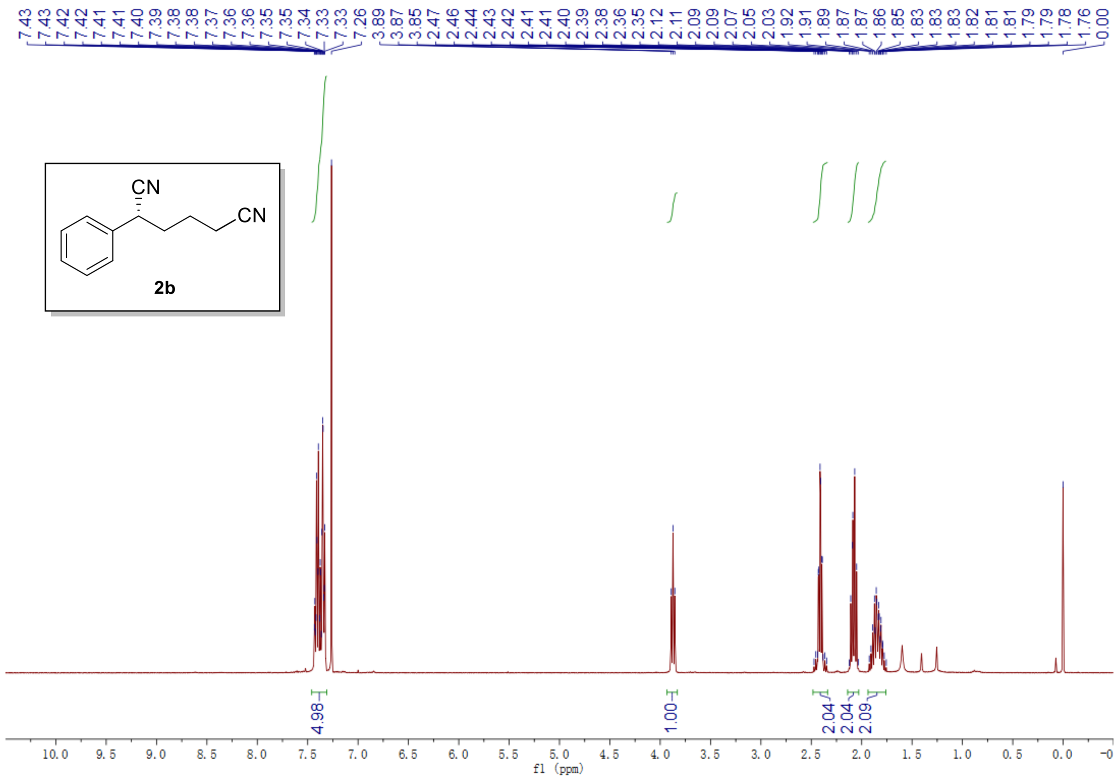

Supplementary Figure 63. ^{1}H NMR of **2b**

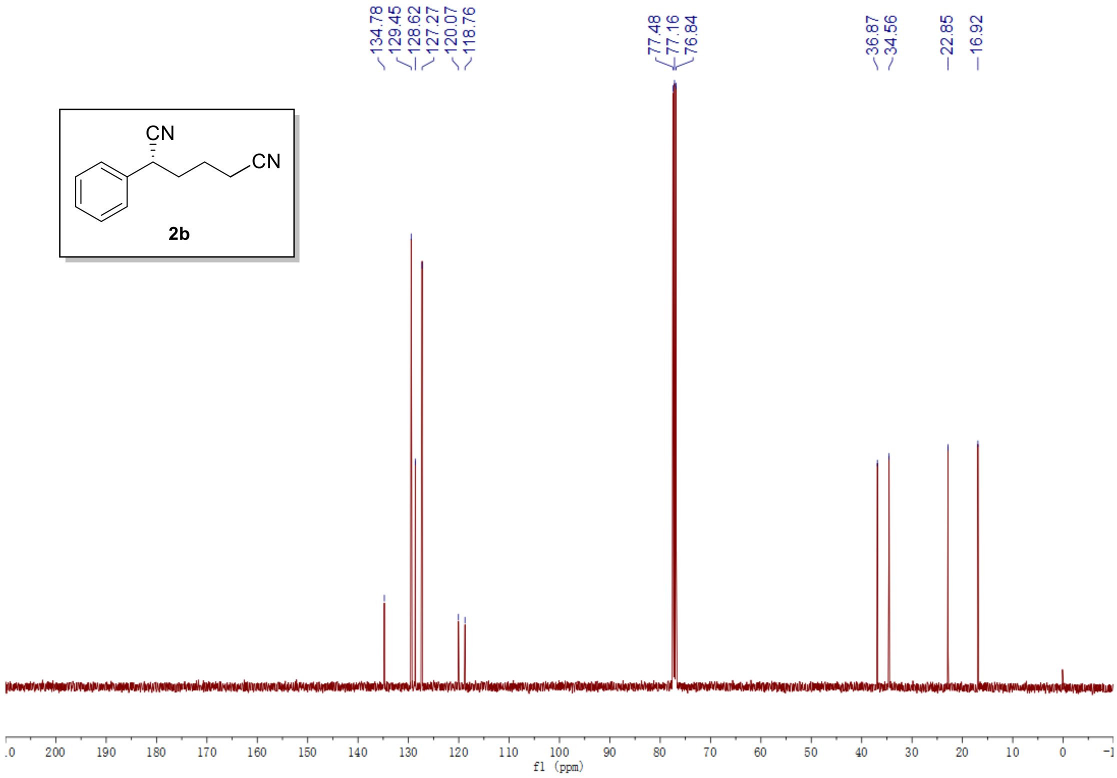

Supplementary Figure 64. ^{13}C NMR of **2b**

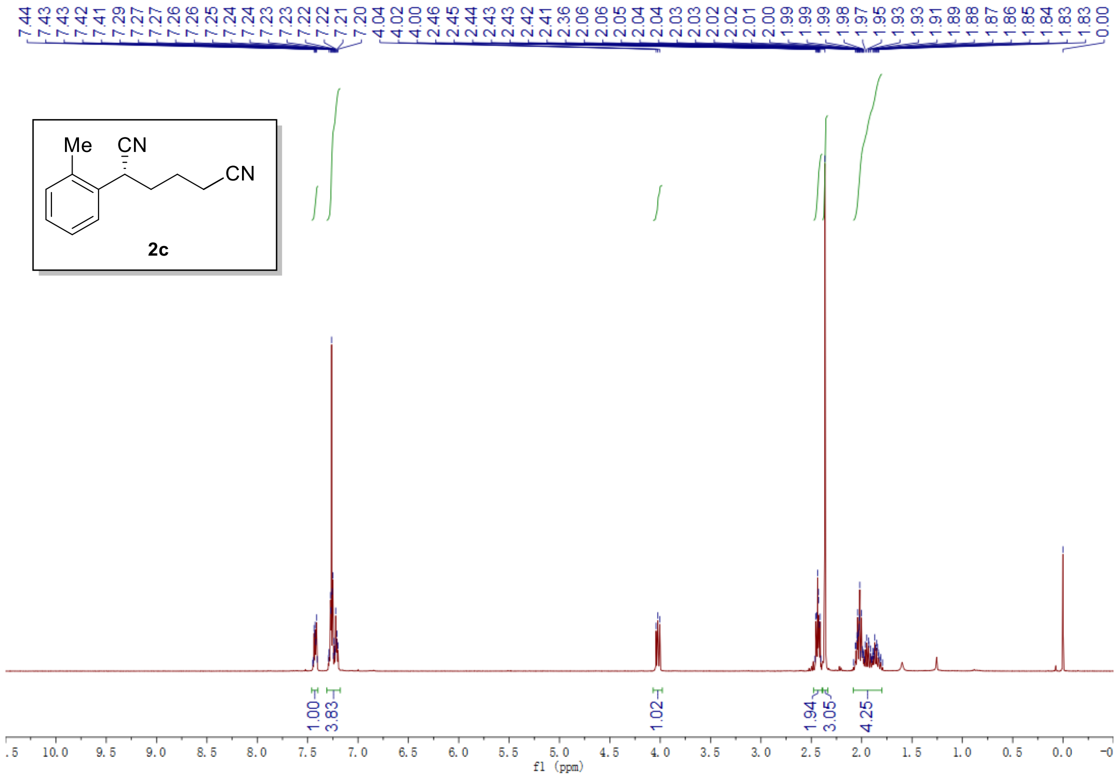

Supplementary Figure 65. ^{1}H NMR of **2c**

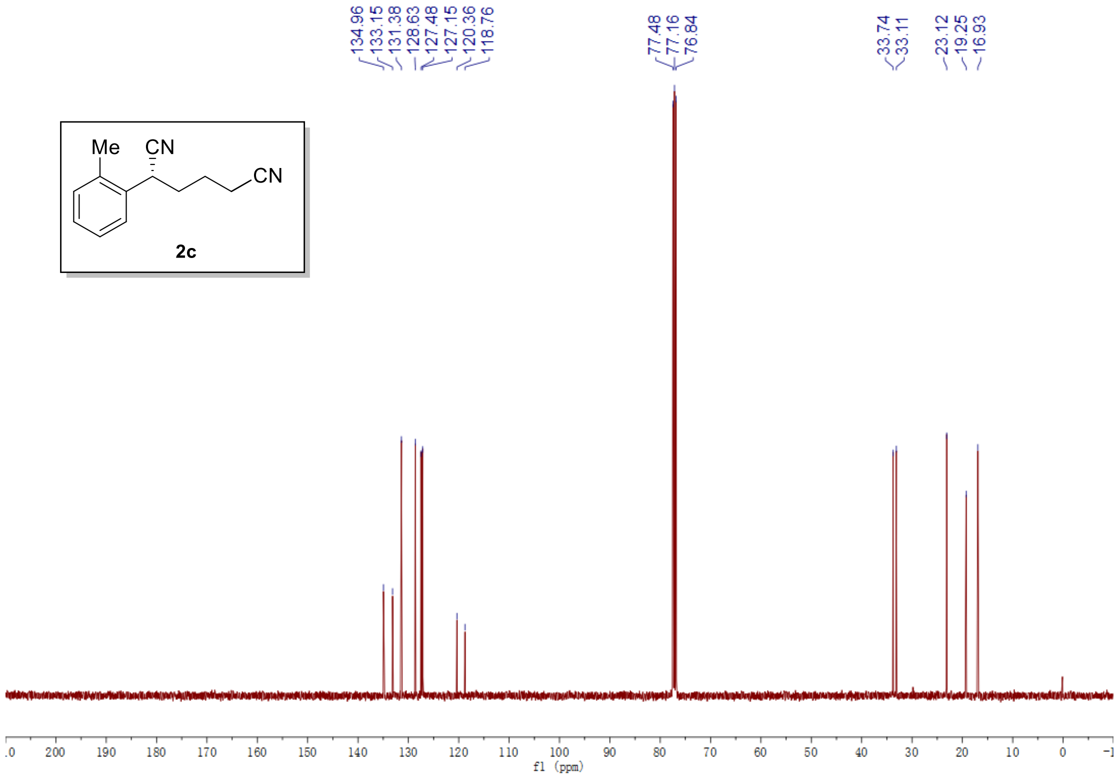

Supplementary Figure 66. ^{13}C NMR of **2c**

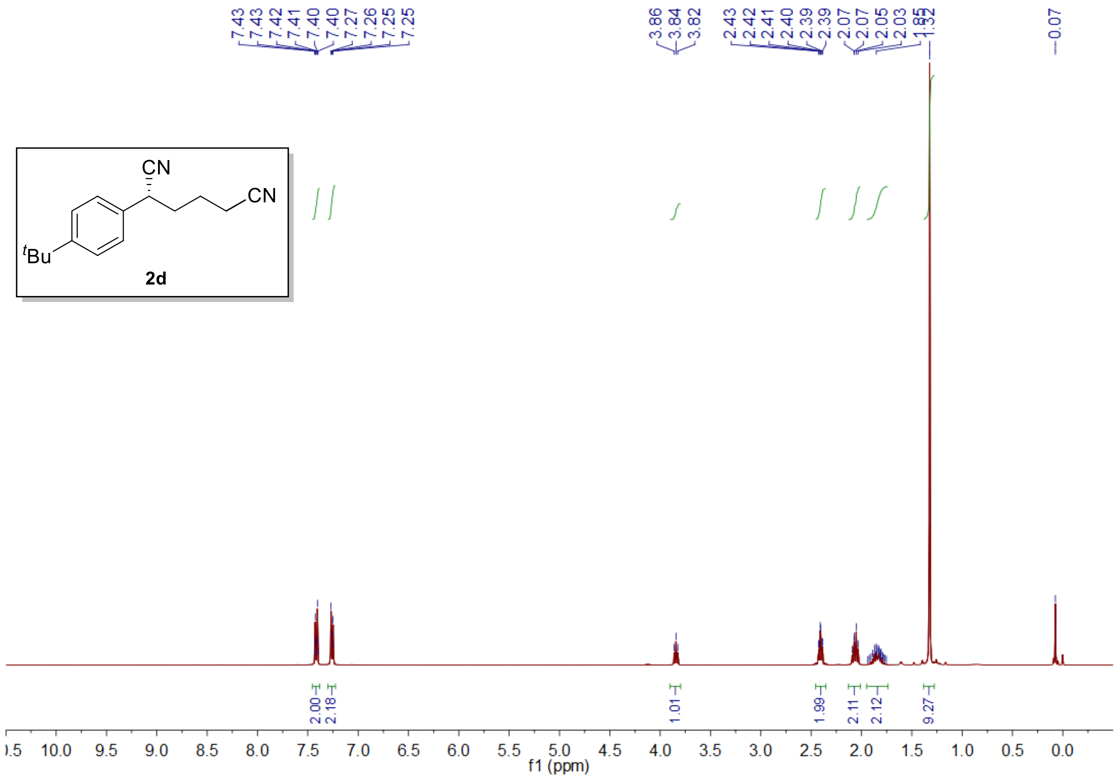

Supplementary Figure 67. ^{1}H NMR of **2d**

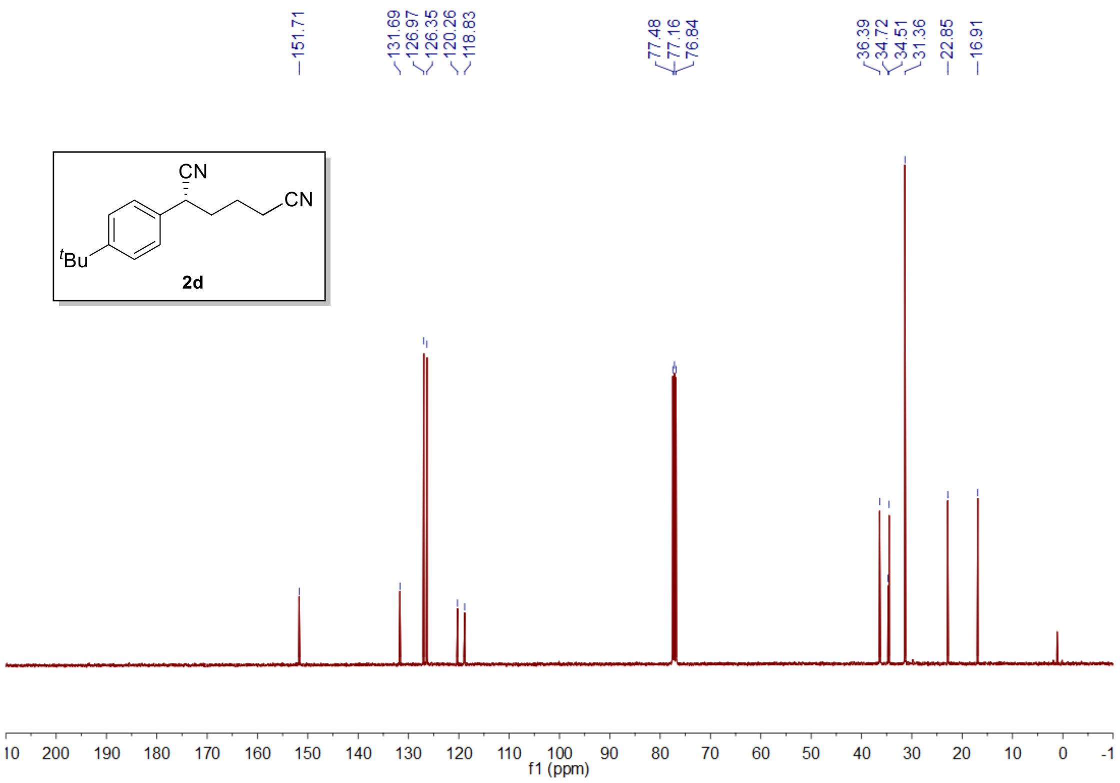

Supplementary Figure 68. ^{13}C NMR of **2d**

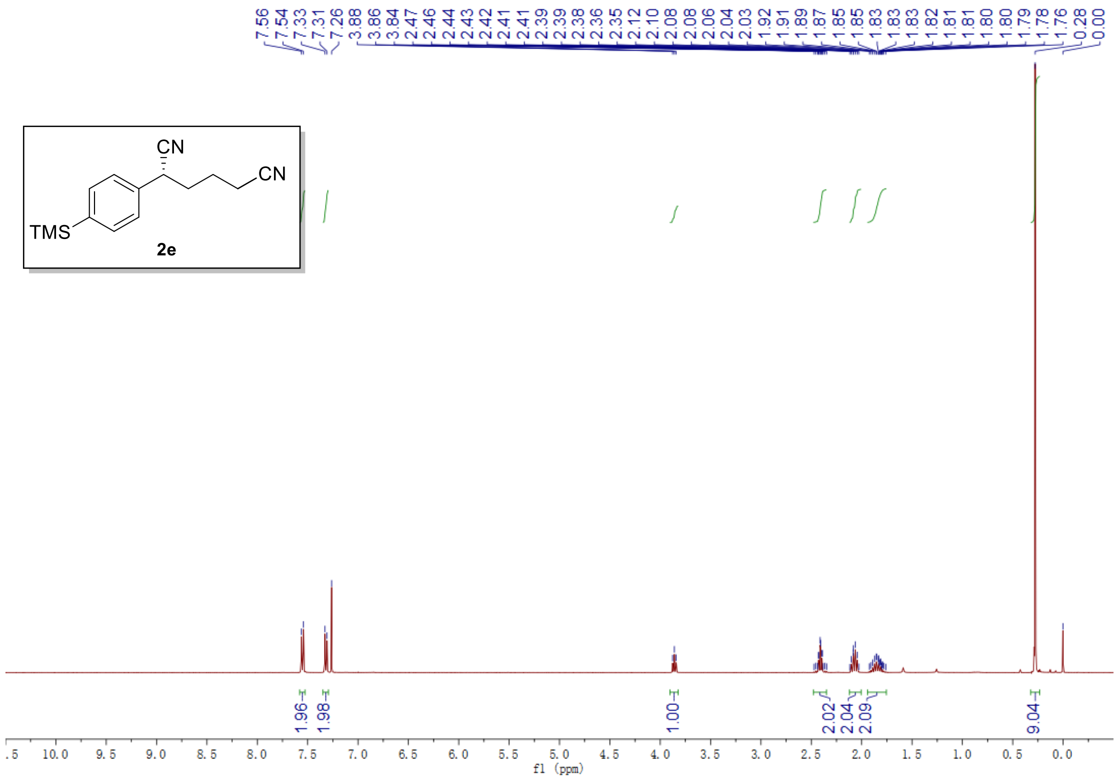

Supplementary Figure 69. ^{1}H NMR of **2e**

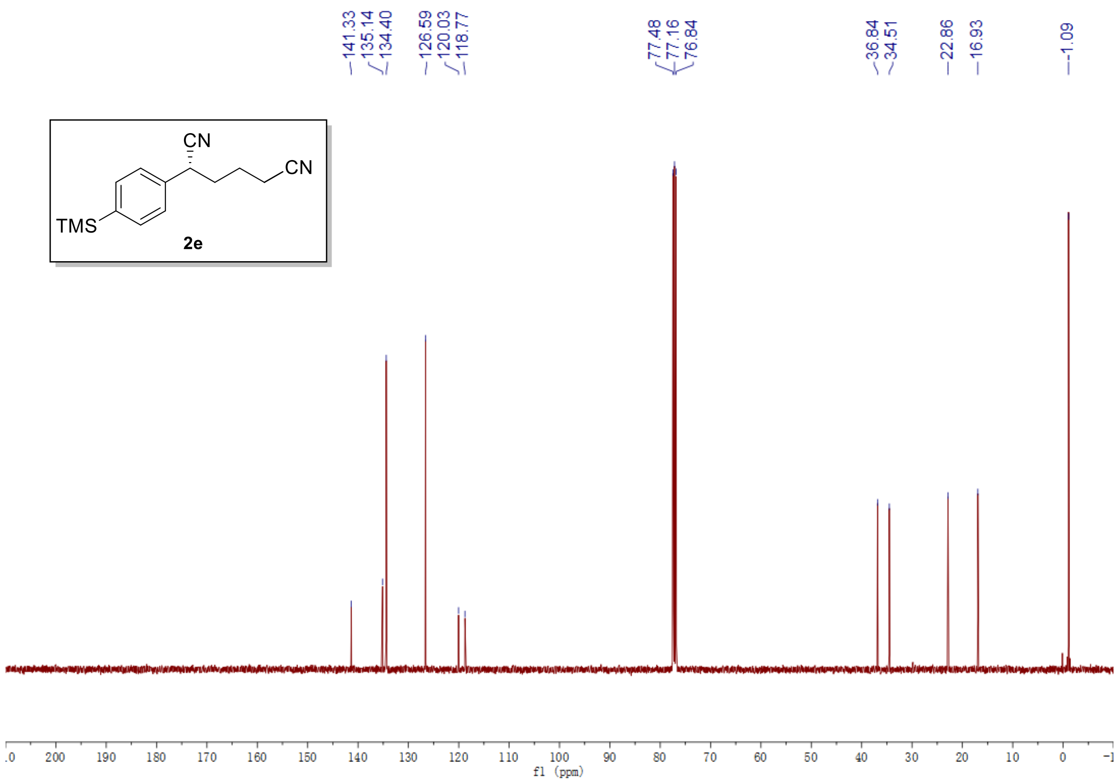

Supplementary Figure 70. ^{13}C NMR of **2e**

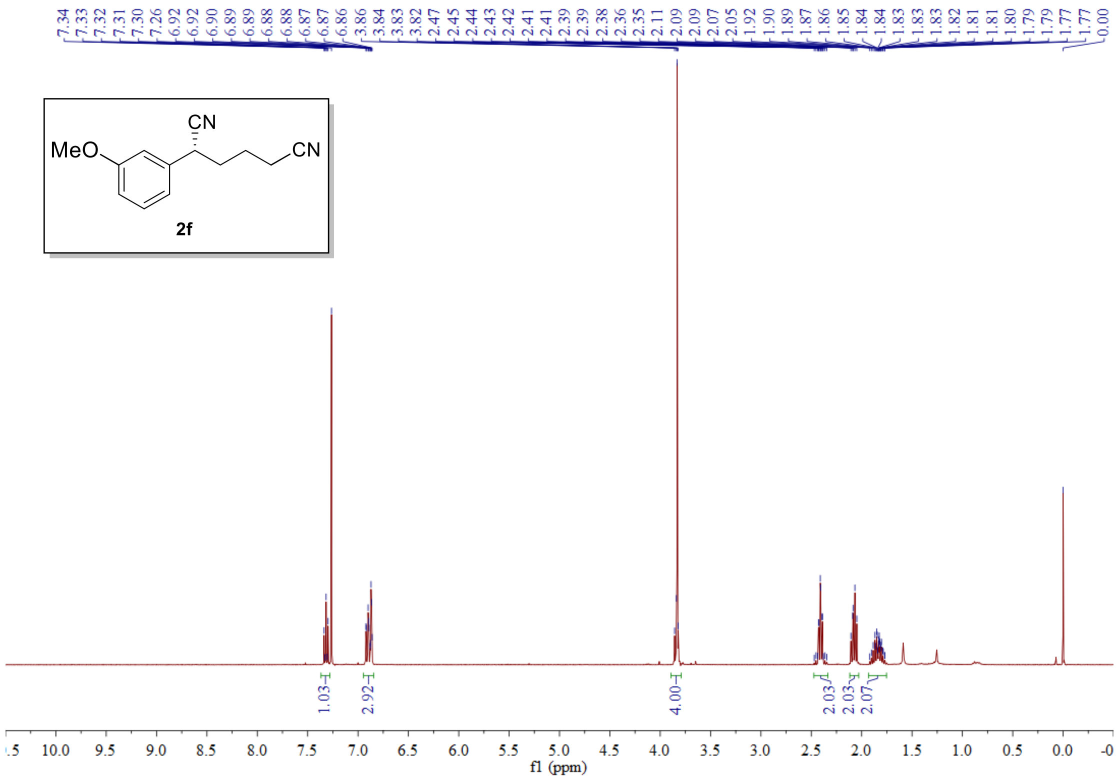

Supplementary Figure 71. ^{1}H NMR of **2f**

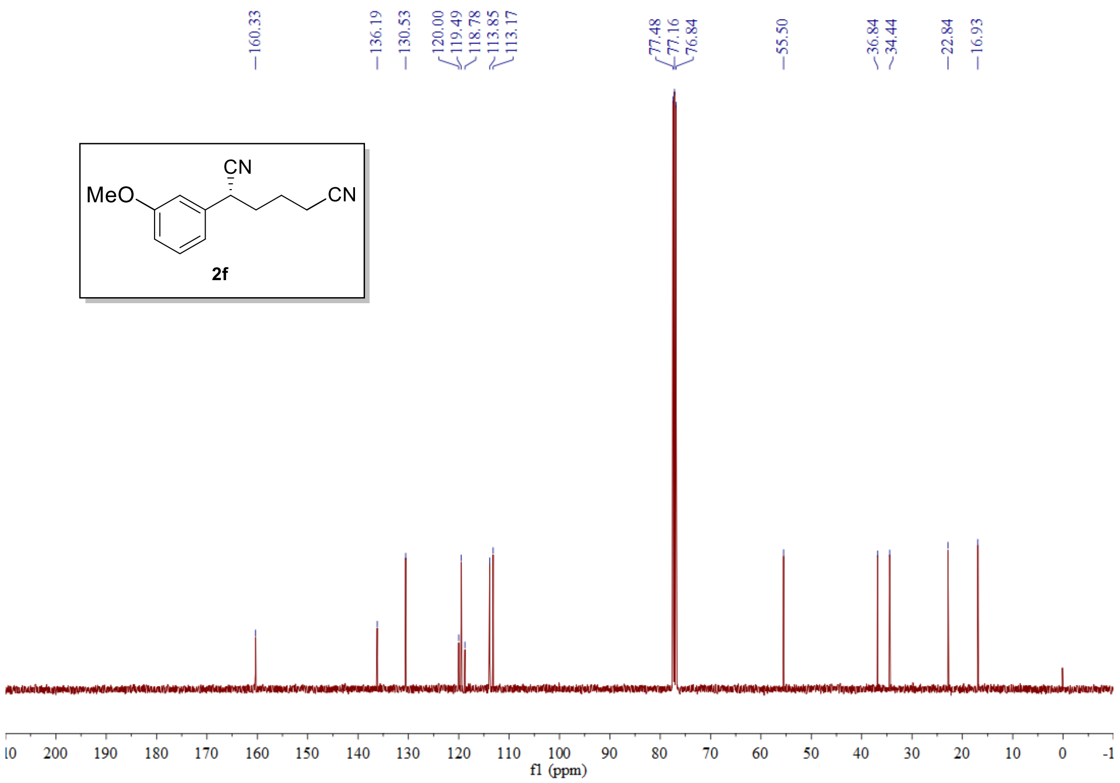

Supplementary Figure 72. ^{13}C NMR of **2f**

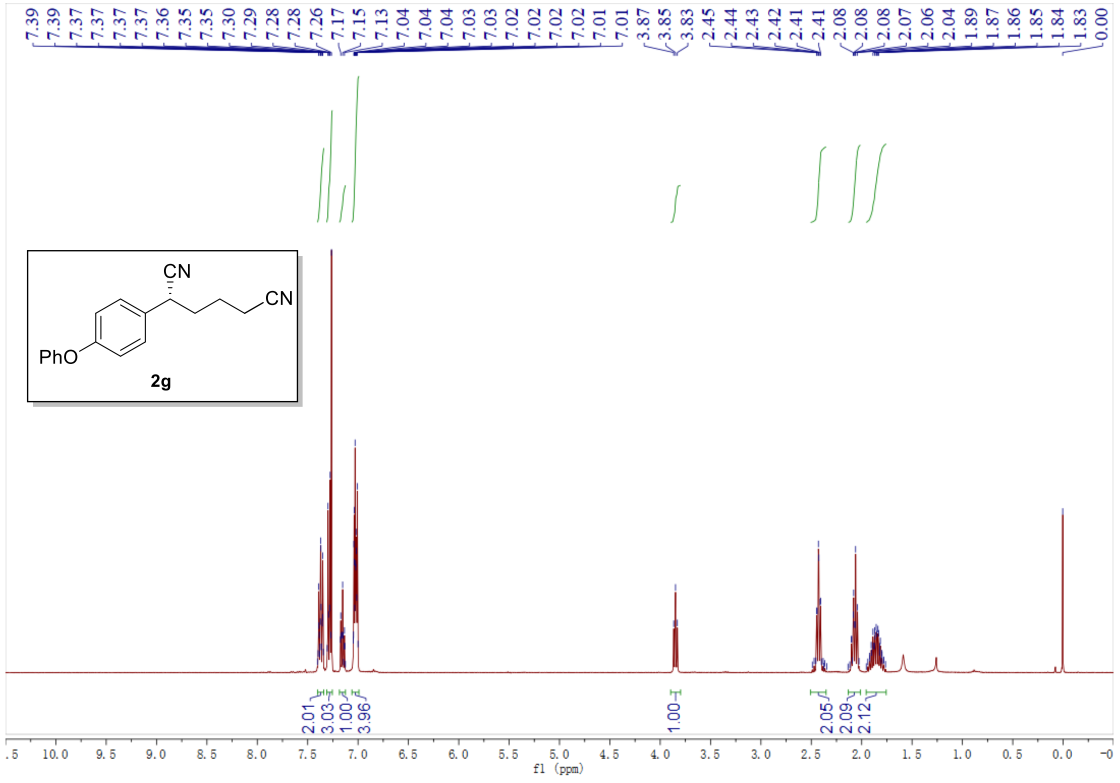

Supplementary Figure 73. ^{1}H NMR of **2g**

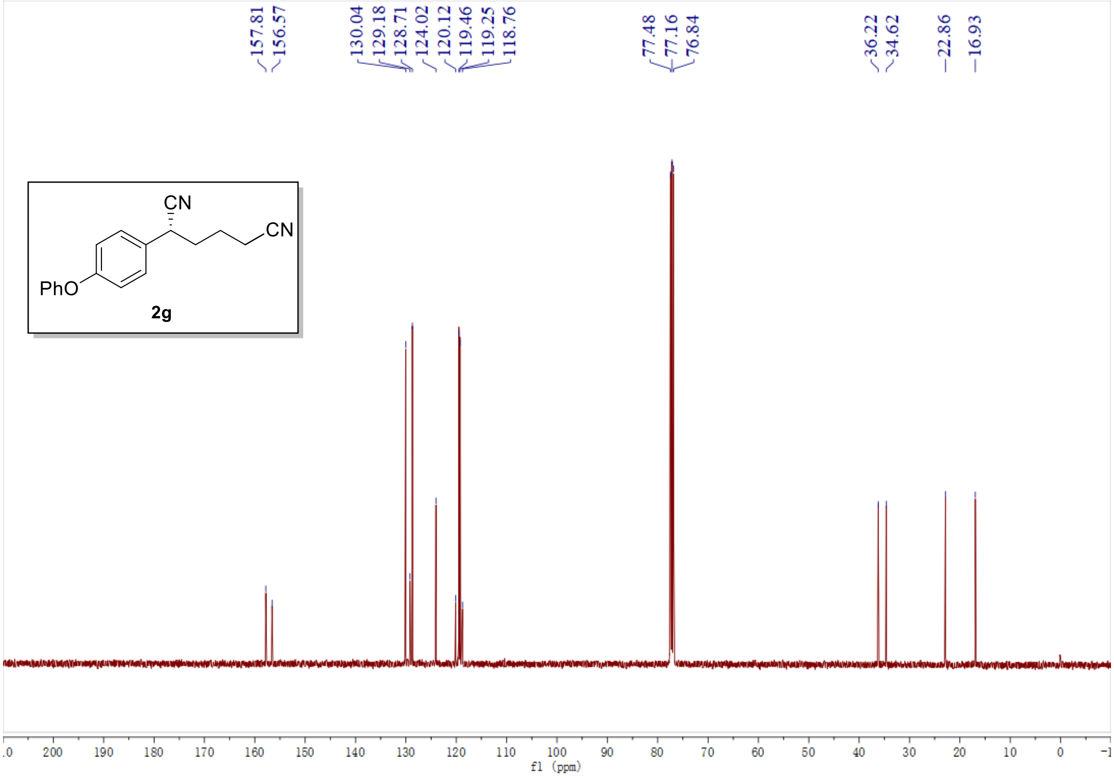

Supplementary Figure 74. ^{13}C NMR of **2g**

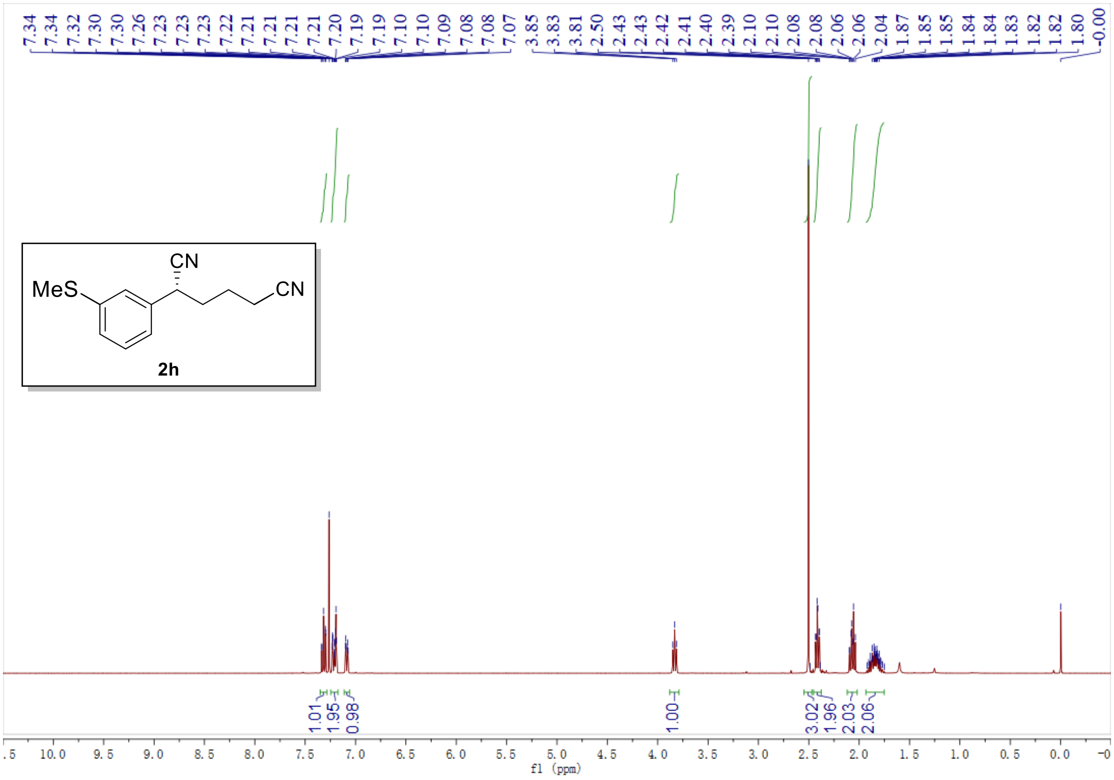

Supplementary Figure 75. ^{1}H NMR of **2h**

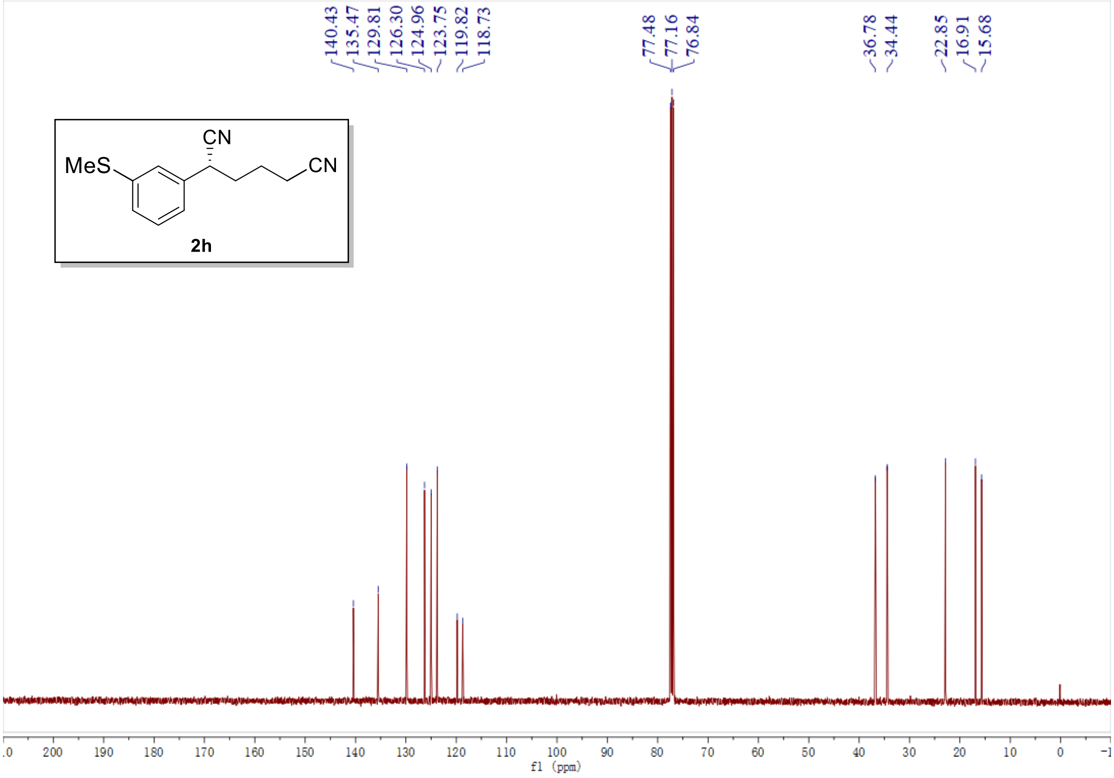

Supplementary Figure 76. ^{13}C NMR of **2h**

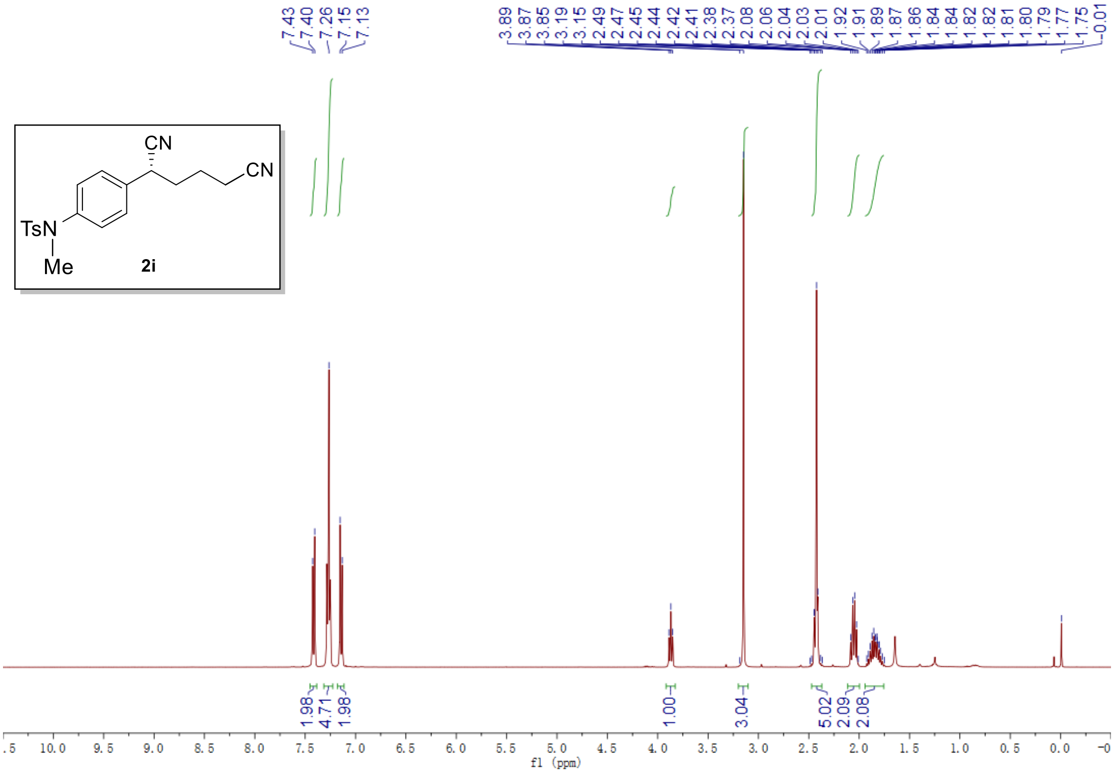

Supplementary Figure 77. ^{1}H NMR of **2i**

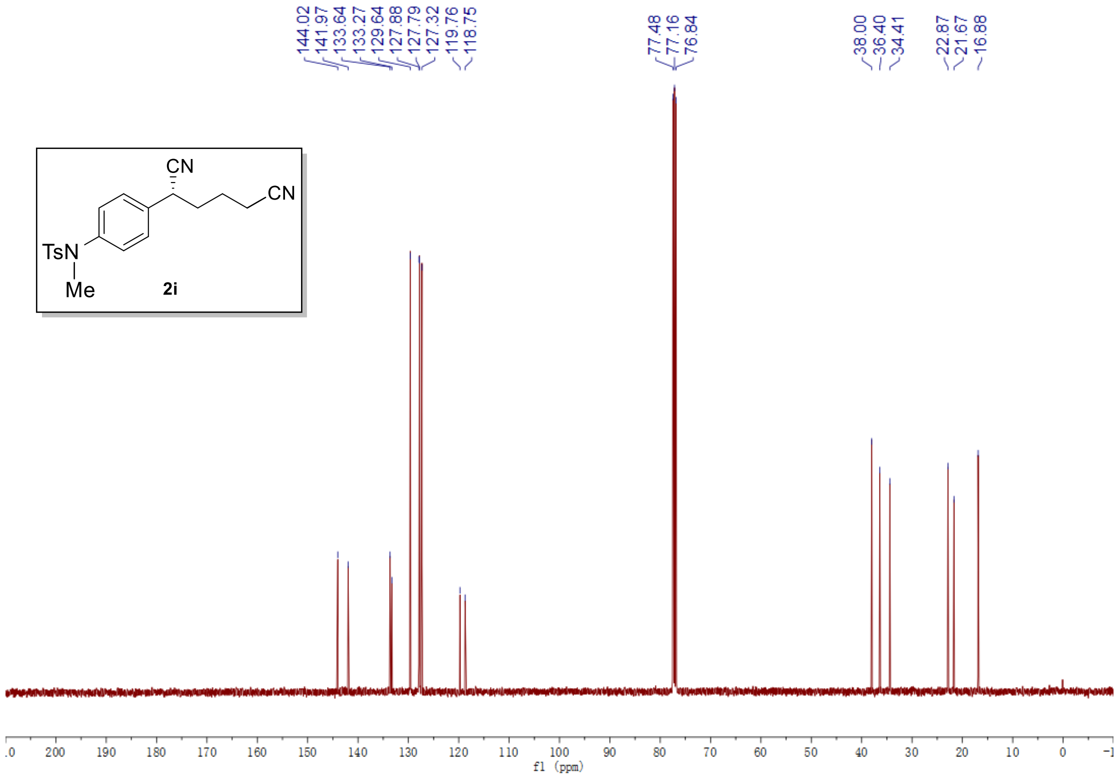

Supplementary Figure 78. ^{13}C NMR of **2i**

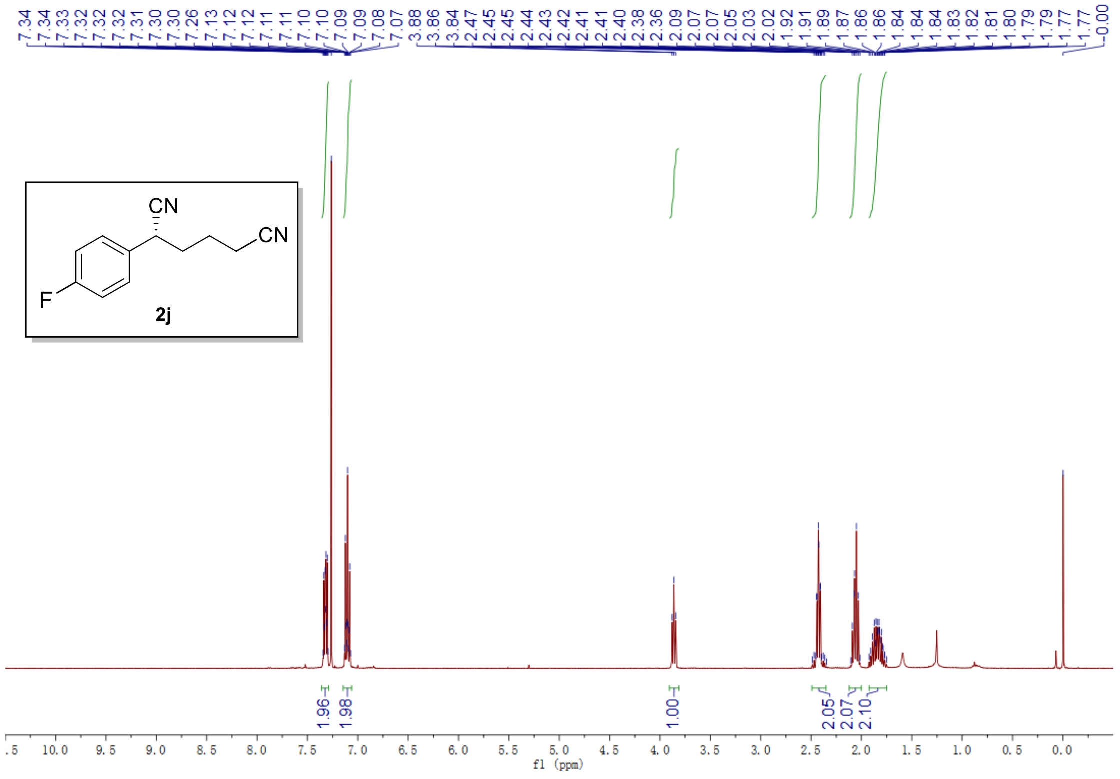

Supplementary Figure 79. ^{1}H NMR of **2j**

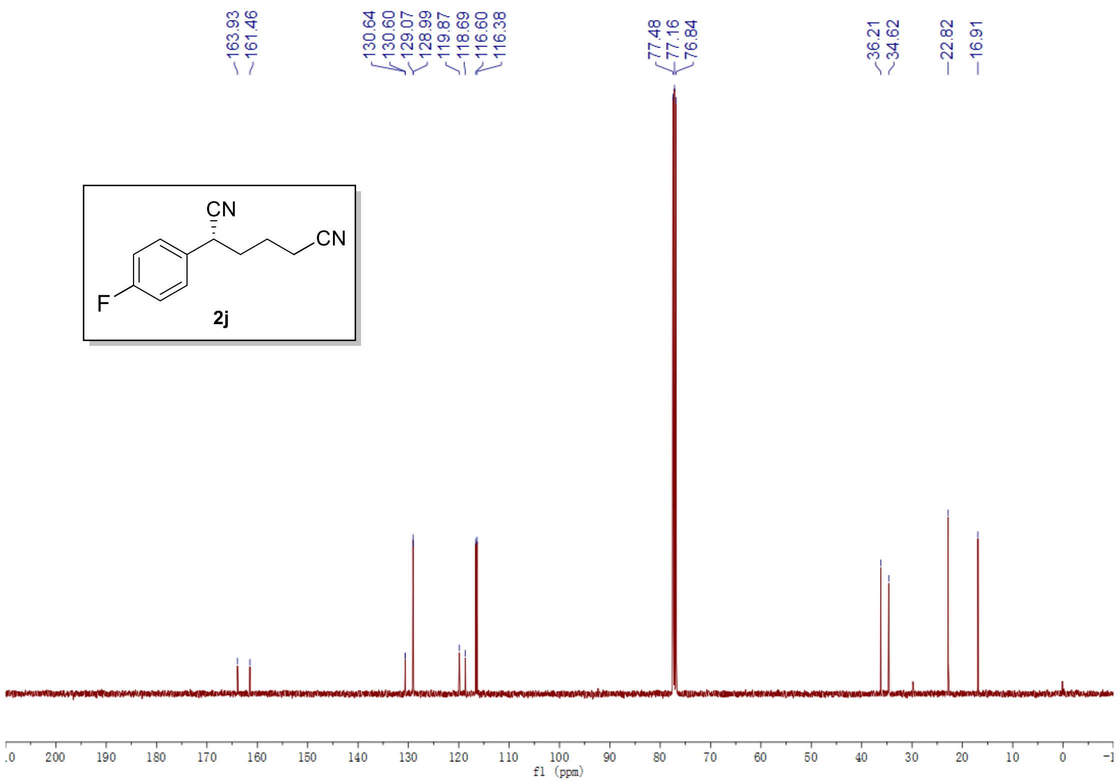

Supplementary Figure 80. ^{13}C NMR of **2j**

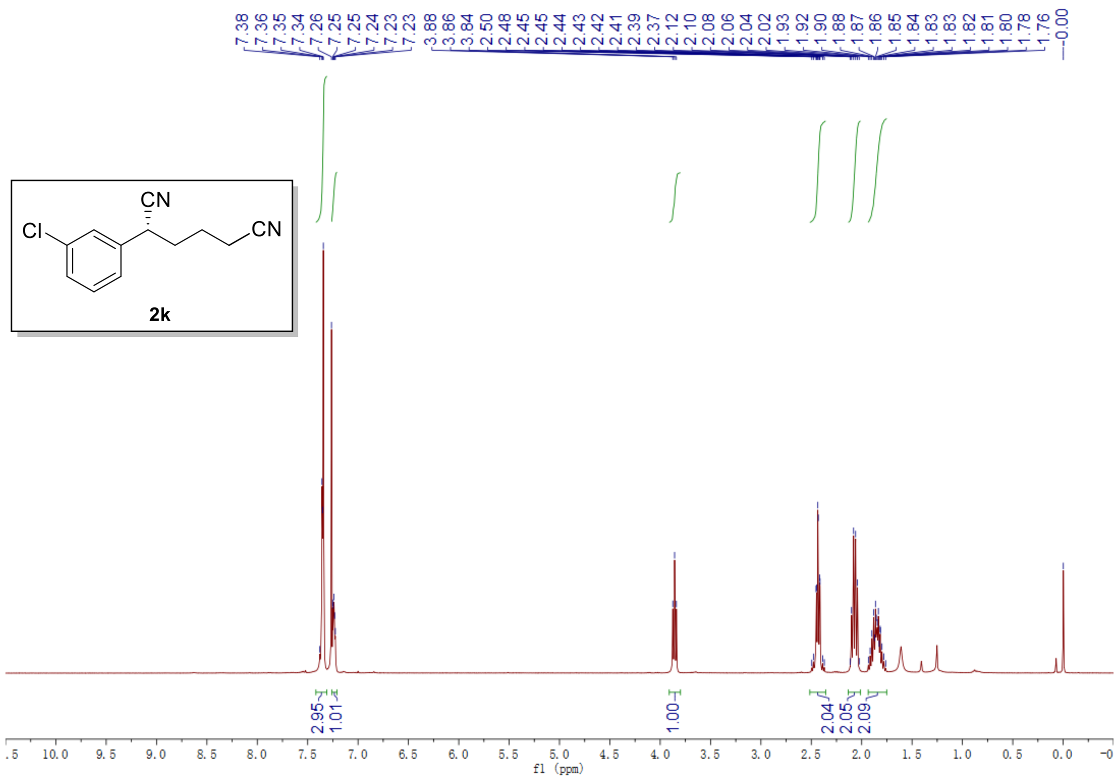

Supplementary Figure 81. ^{1}H NMR of **2k**

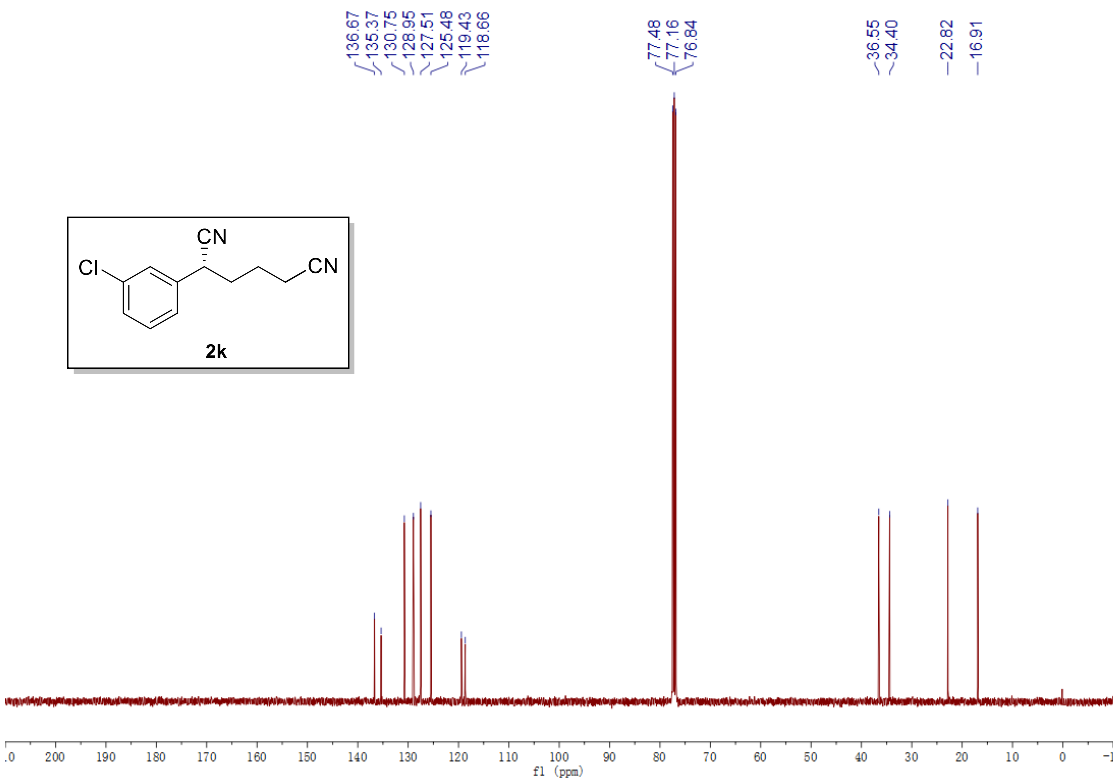

Supplementary Figure 82. ^{13}C NMR of **2k**

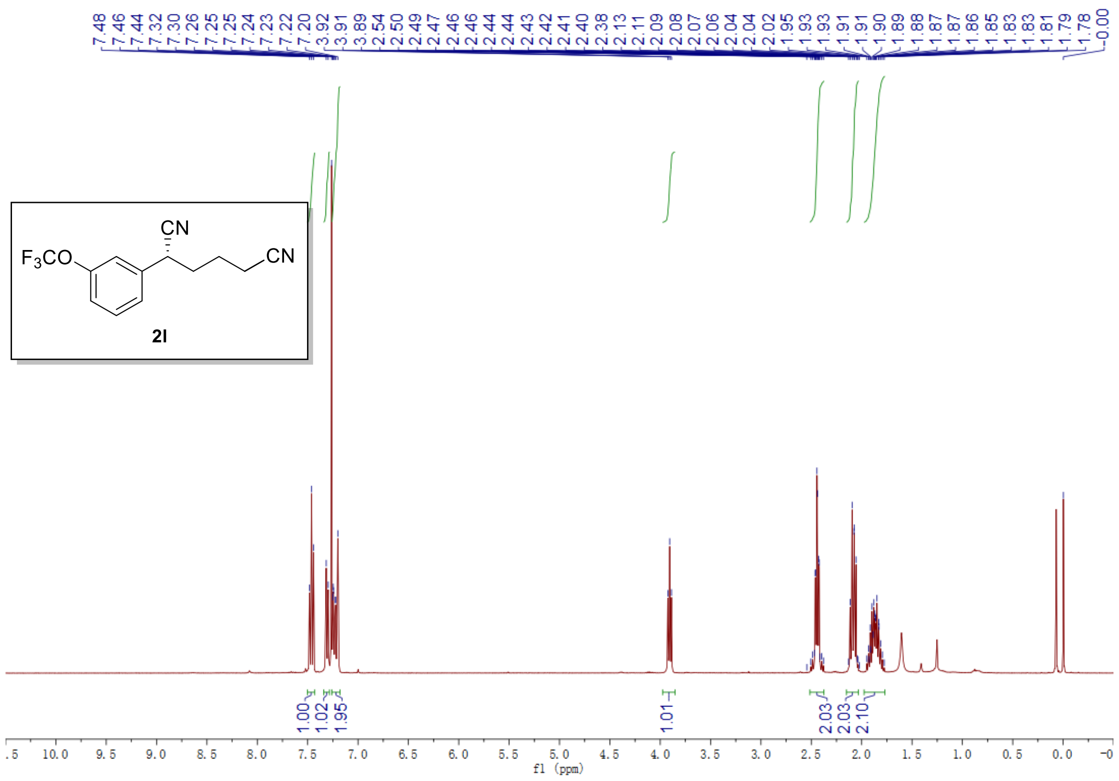

Supplementary Figure 83. ^{1}H NMR of **2l**

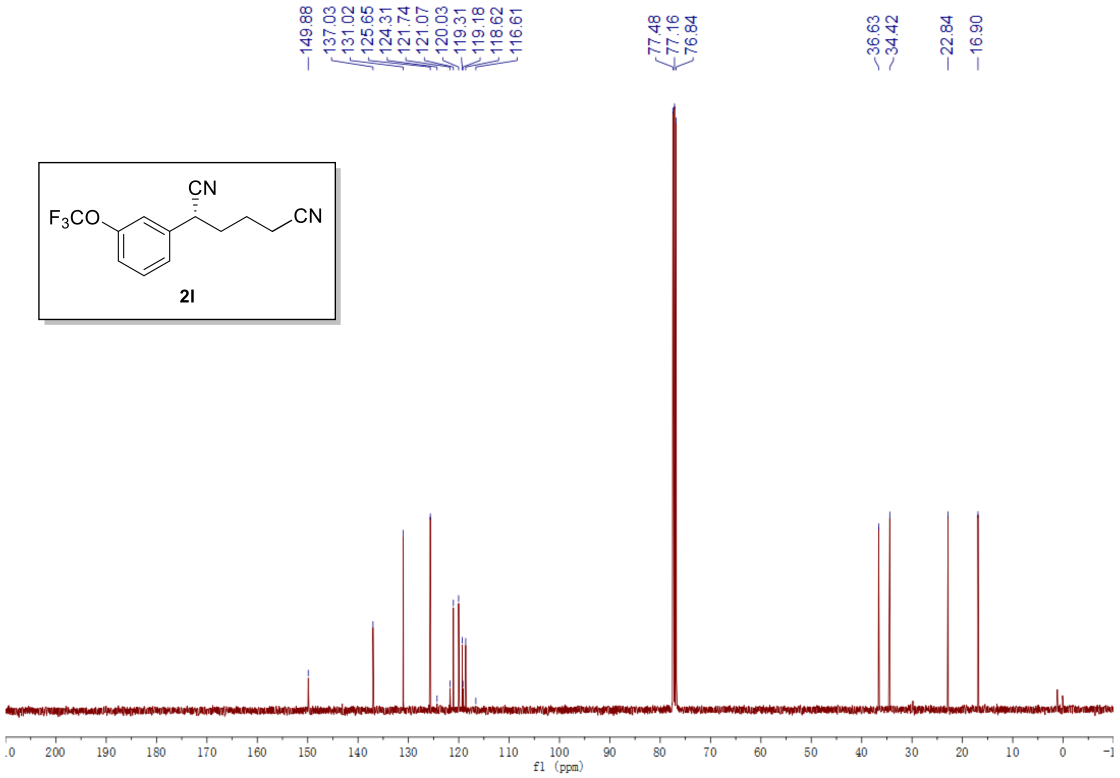

Supplementary Figure 84. ^{13}C NMR of **2l**

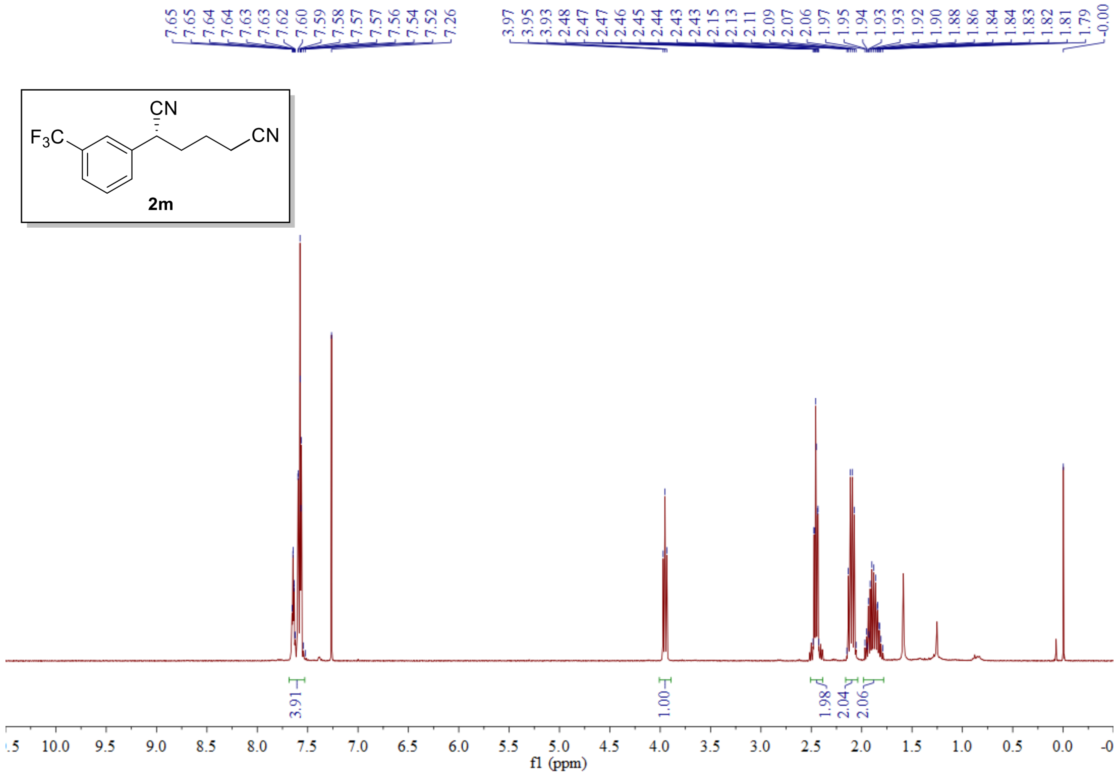

Supplementary Figure 85. ^{1}H NMR of **2m**

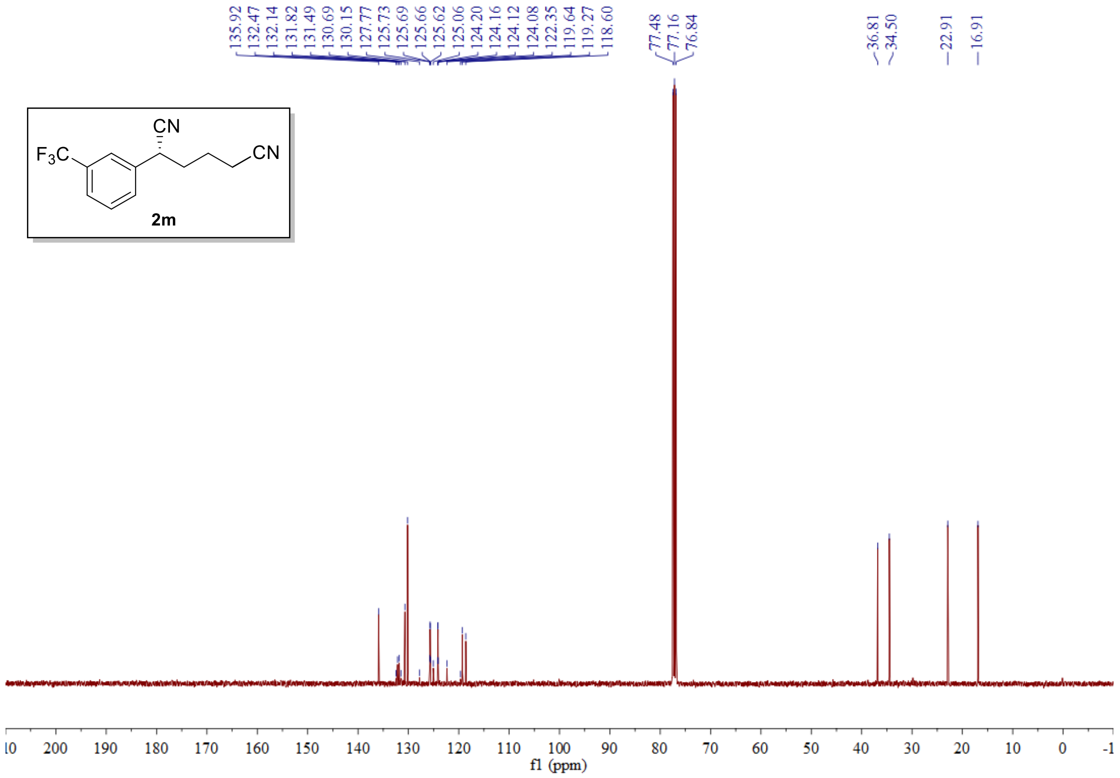

Supplementary Figure 86. ^{13}C NMR of **2m**

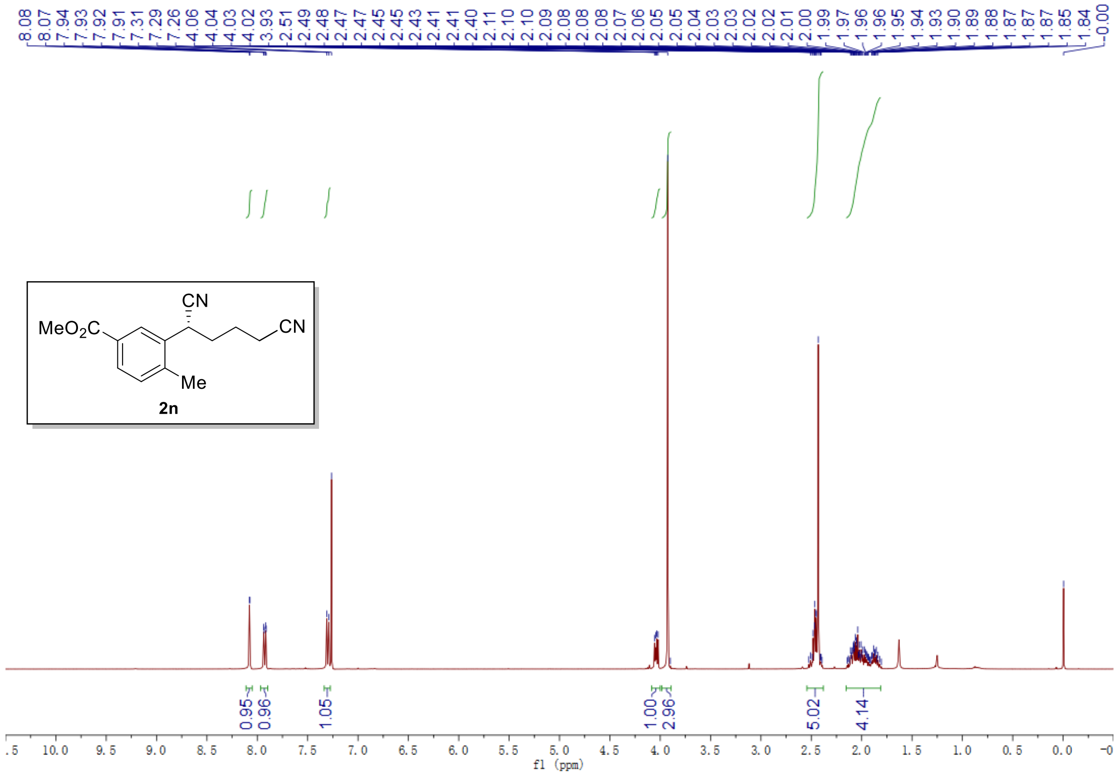

Supplementary Figure 87. ^{1}H NMR of **2n**

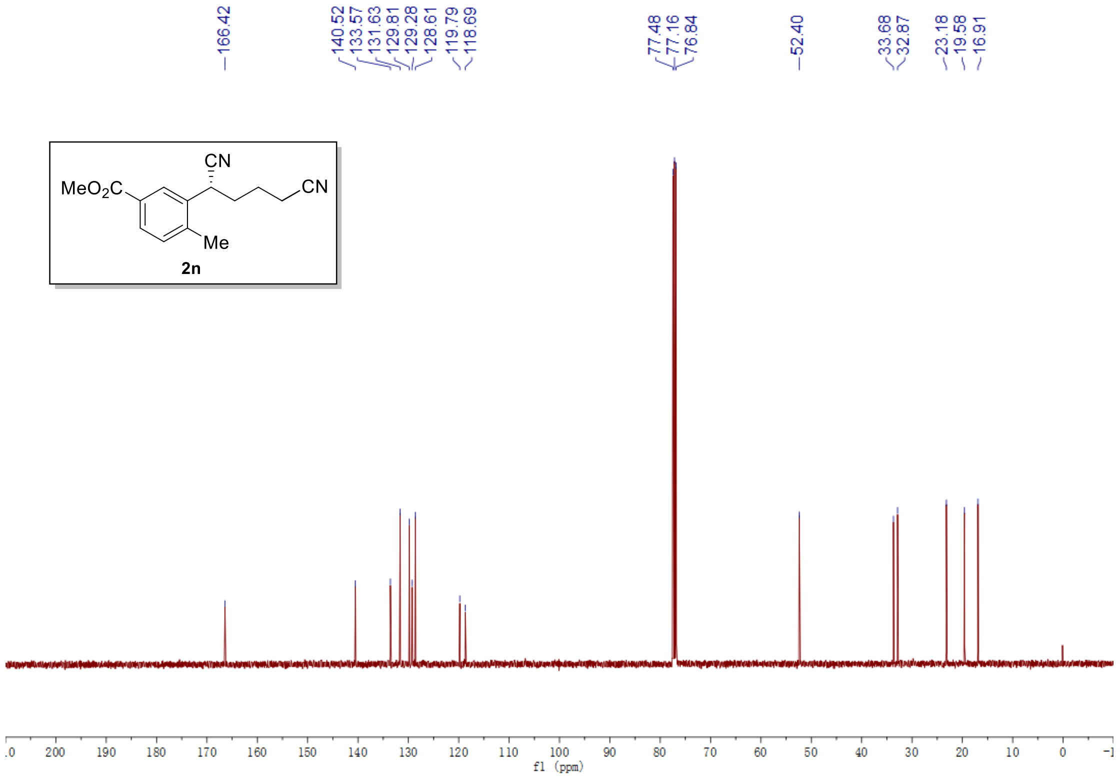

Supplementary Figure 88. ^{13}C NMR of **2n**

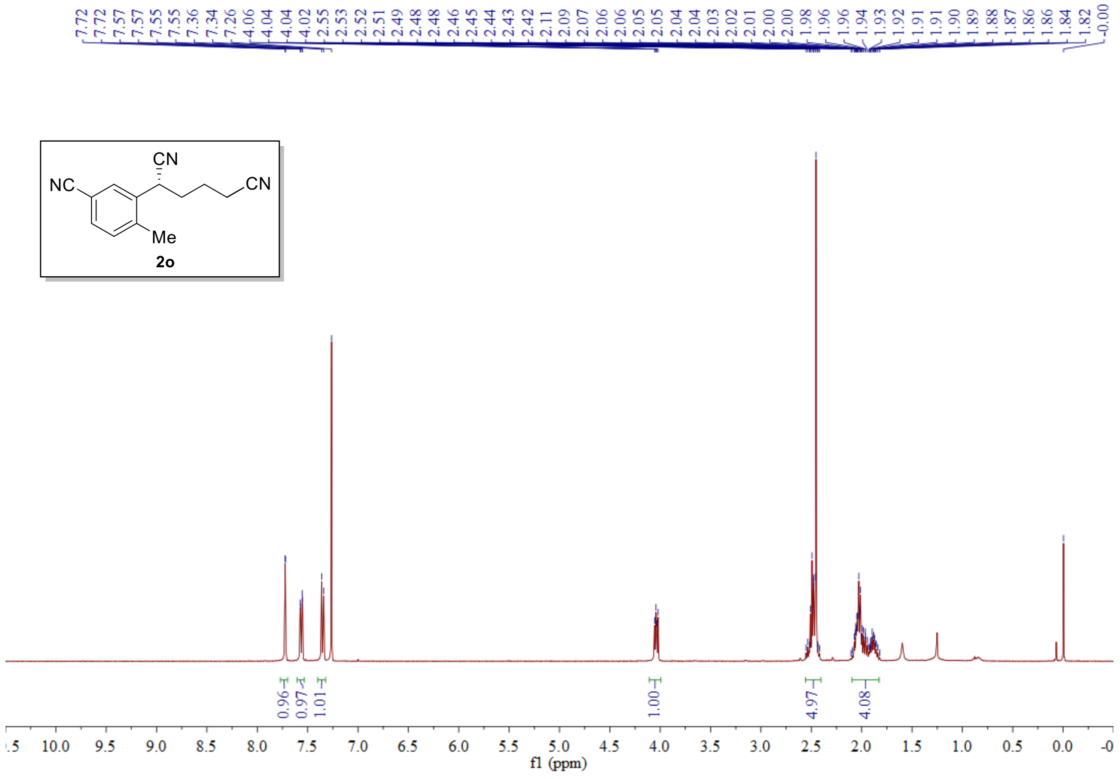

Supplementary Figure 89. ^{1}H NMR of **2o**

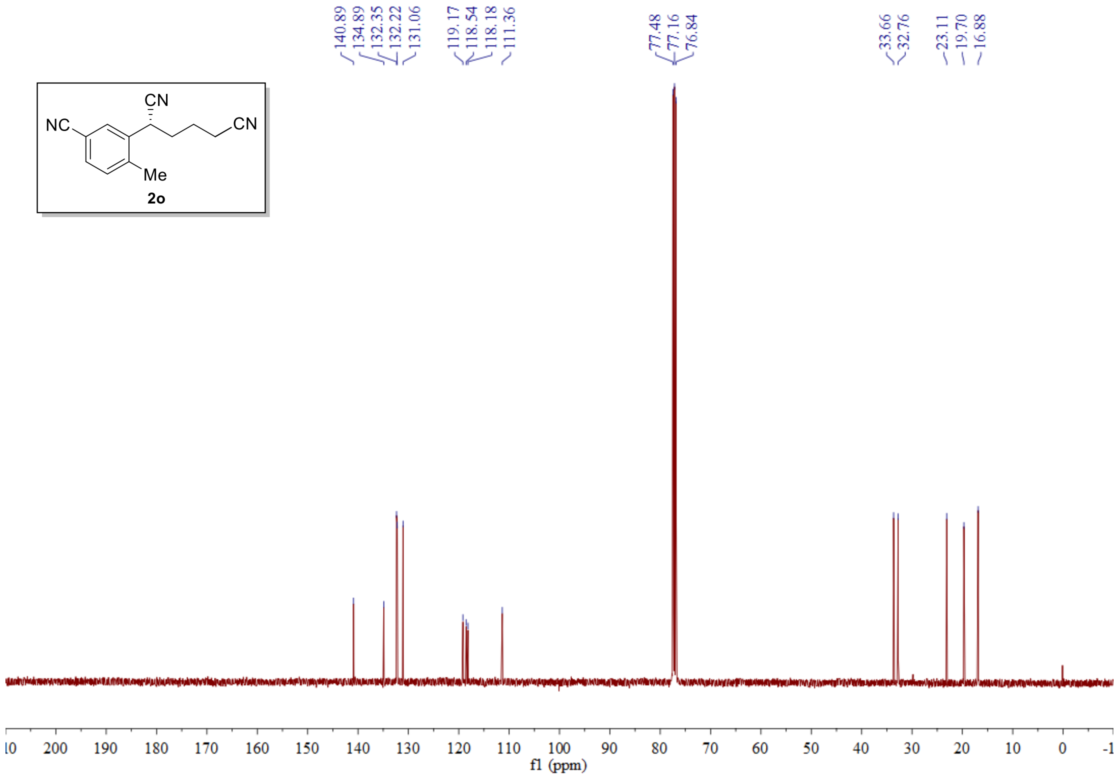

Supplementary Figure 90. ^{13}C NMR of **2o**

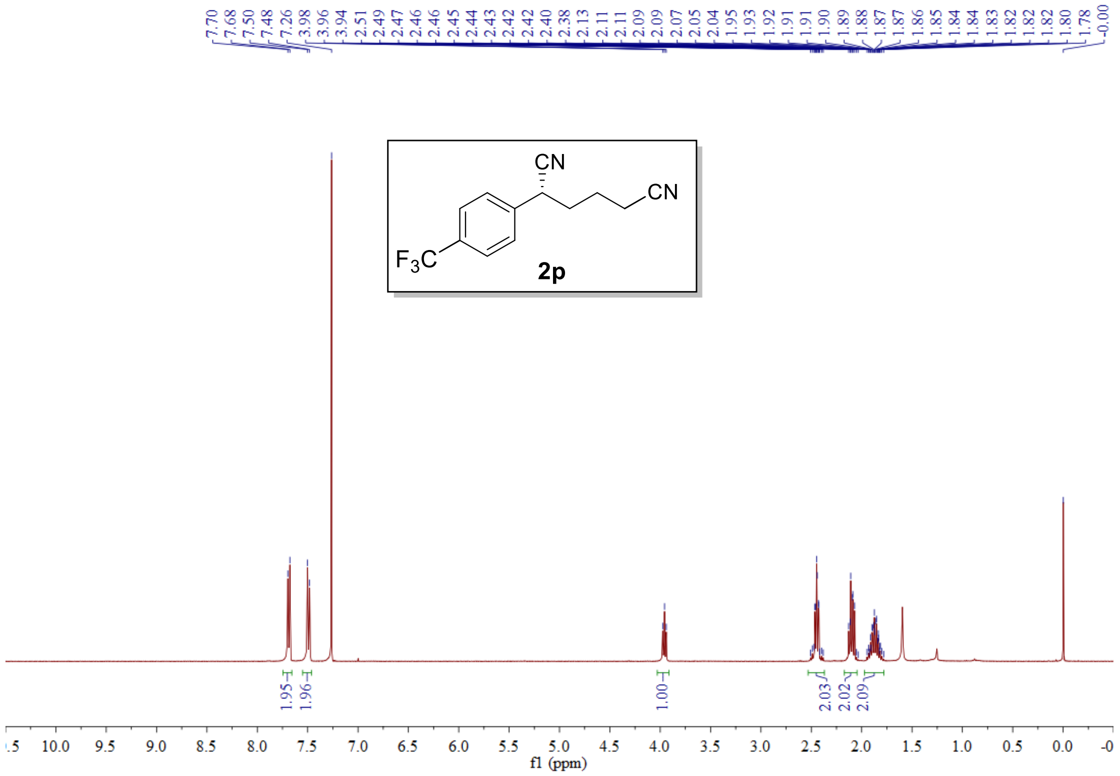

Supplementary Figure 91. ^{1}H NMR of **2p**

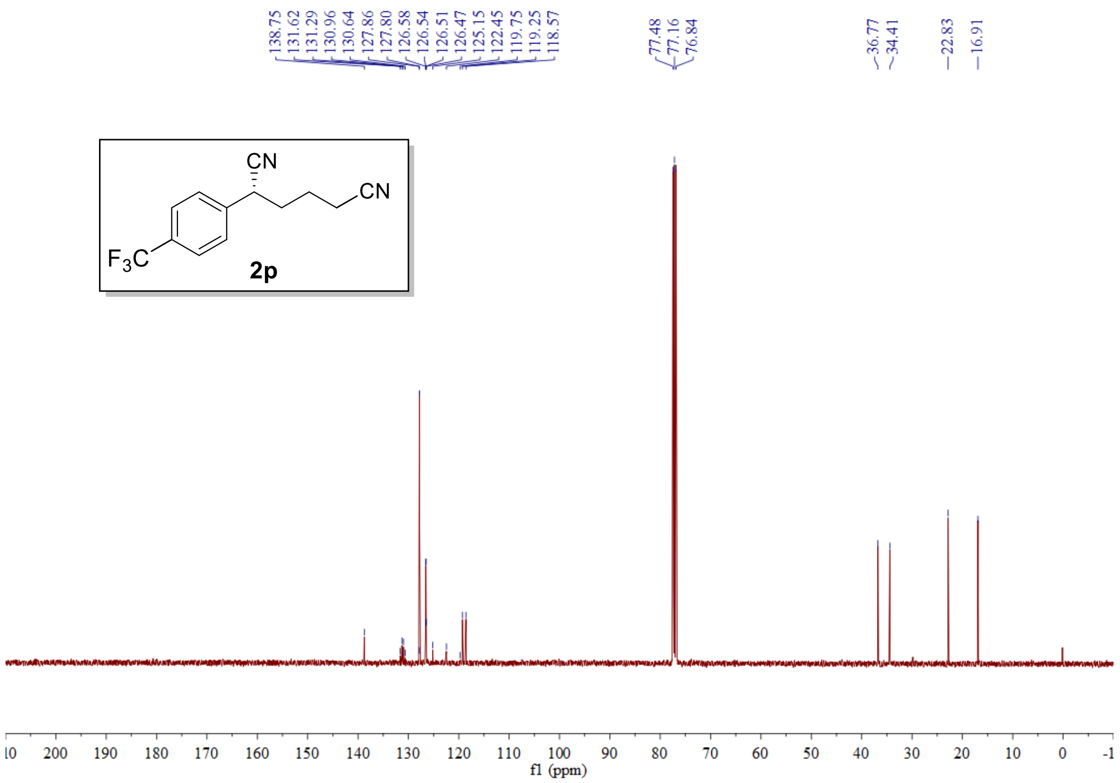

Supplementary Figure 92. ^{13}C NMR of **2p**

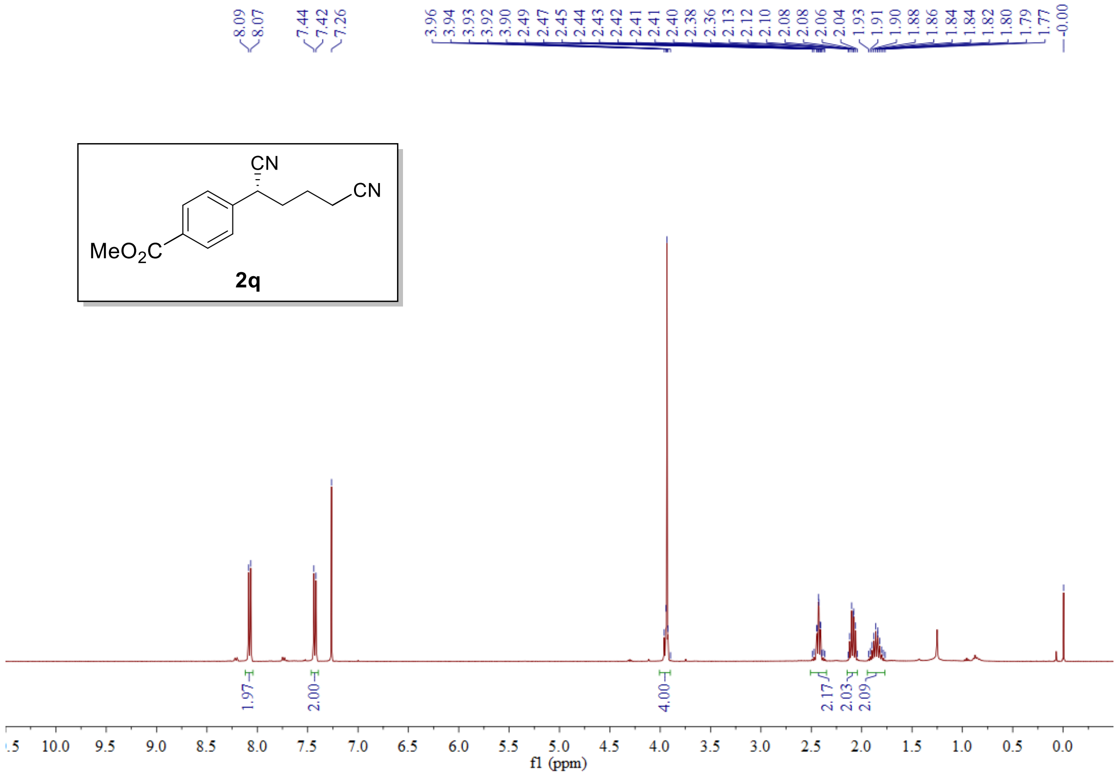

Supplementary Figure 93. ^{1}H NMR of **2q**

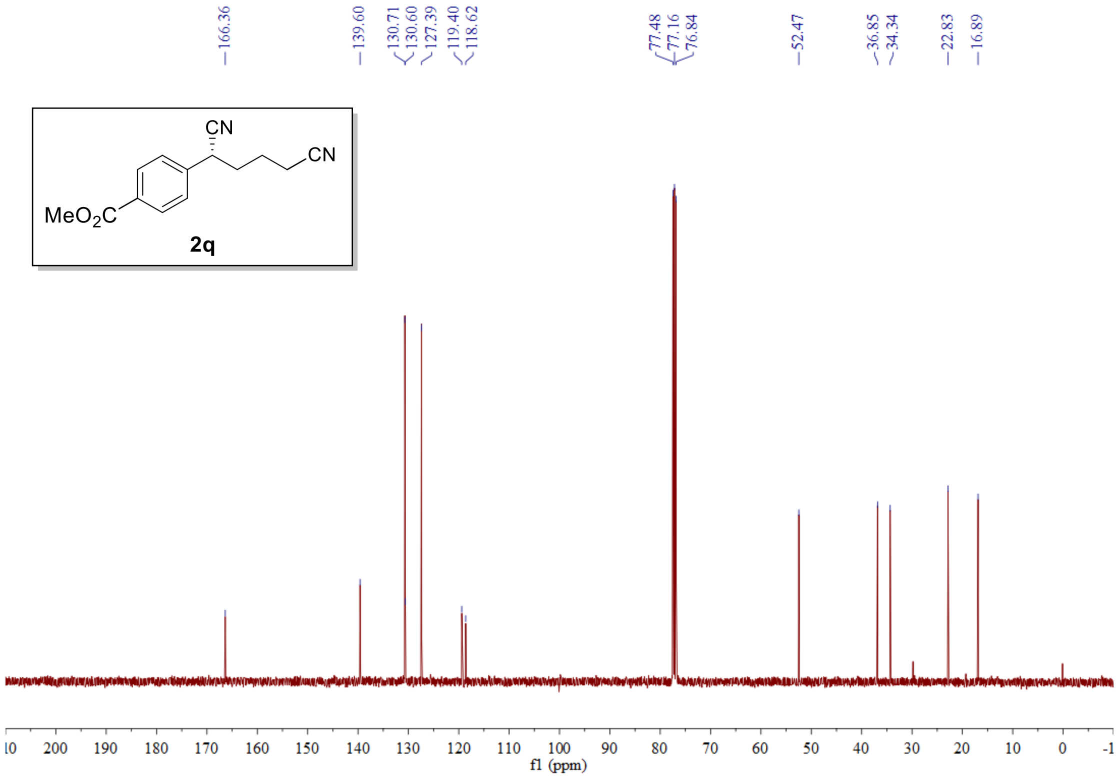

Supplementary Figure 94. ^{13}C NMR of **2q**

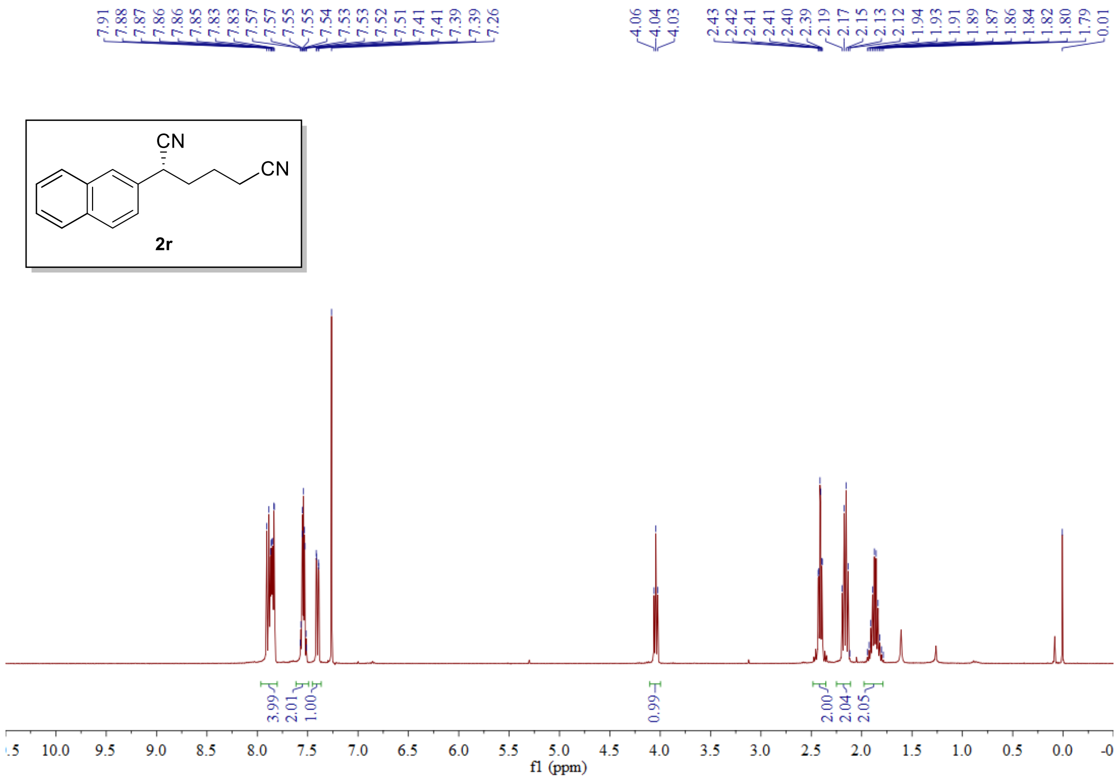

Supplementary Figure 95. ^{1}H NMR of **2r**

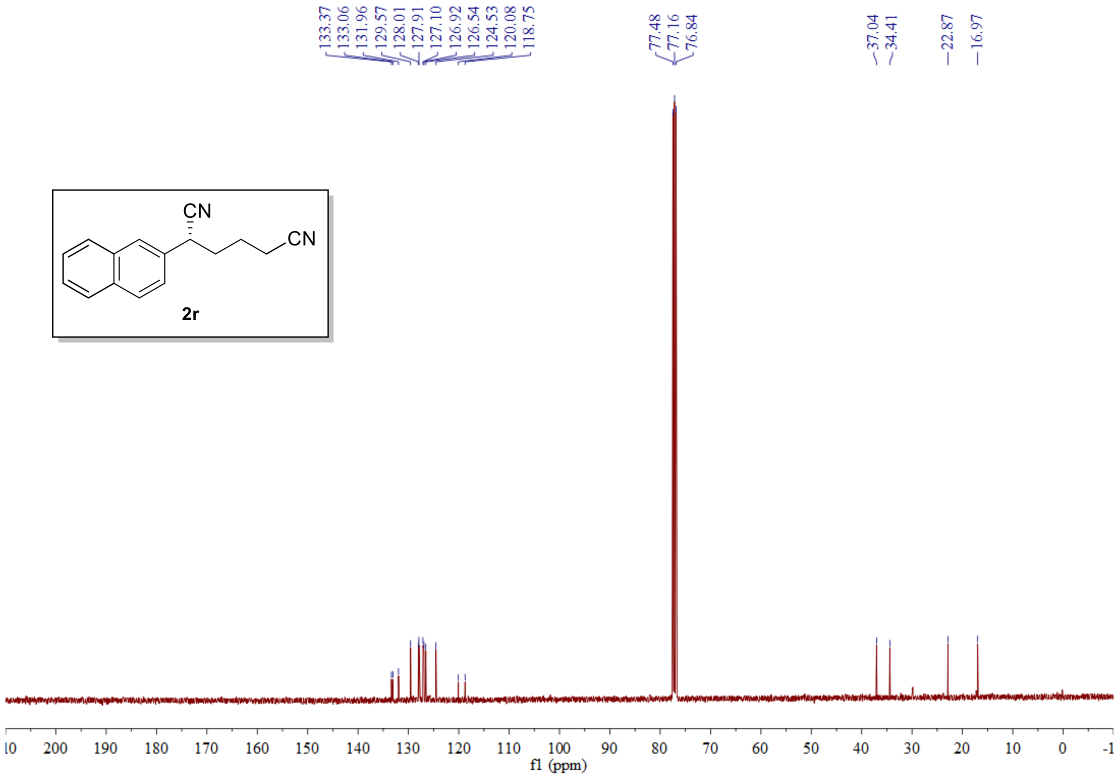

Supplementary Figure 96. ^{13}C NMR of **2r**

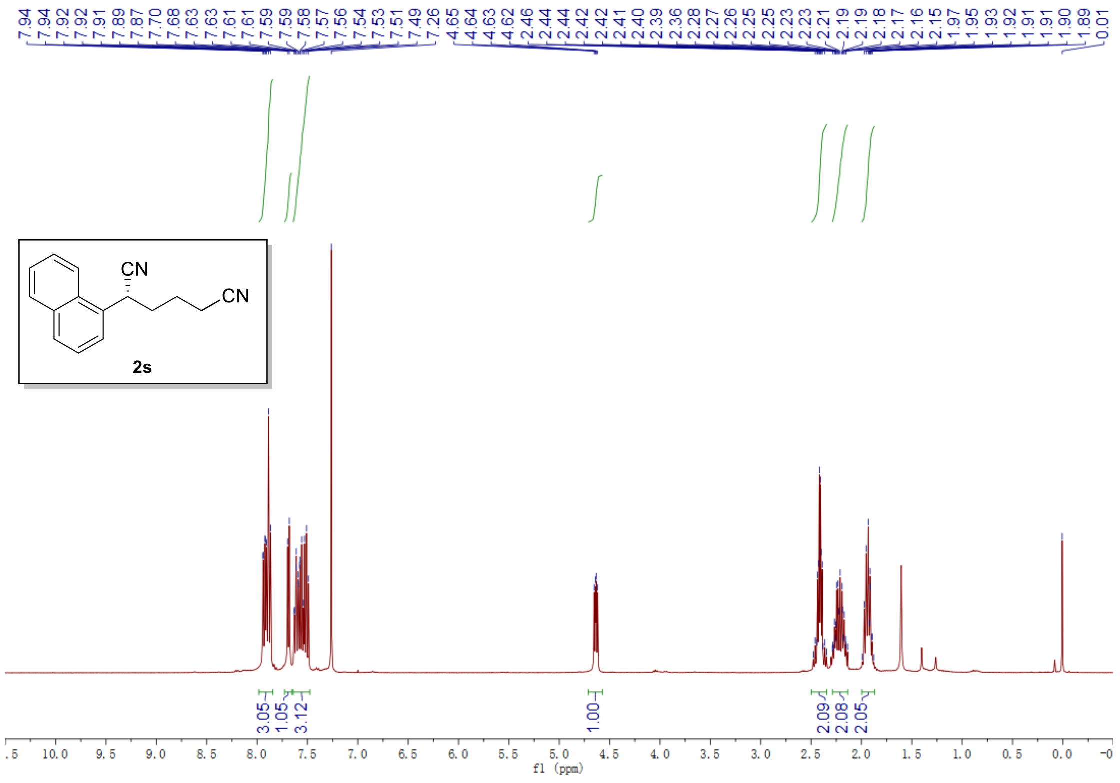

Supplementary Figure 97. ^{1}H NMR of **2s**

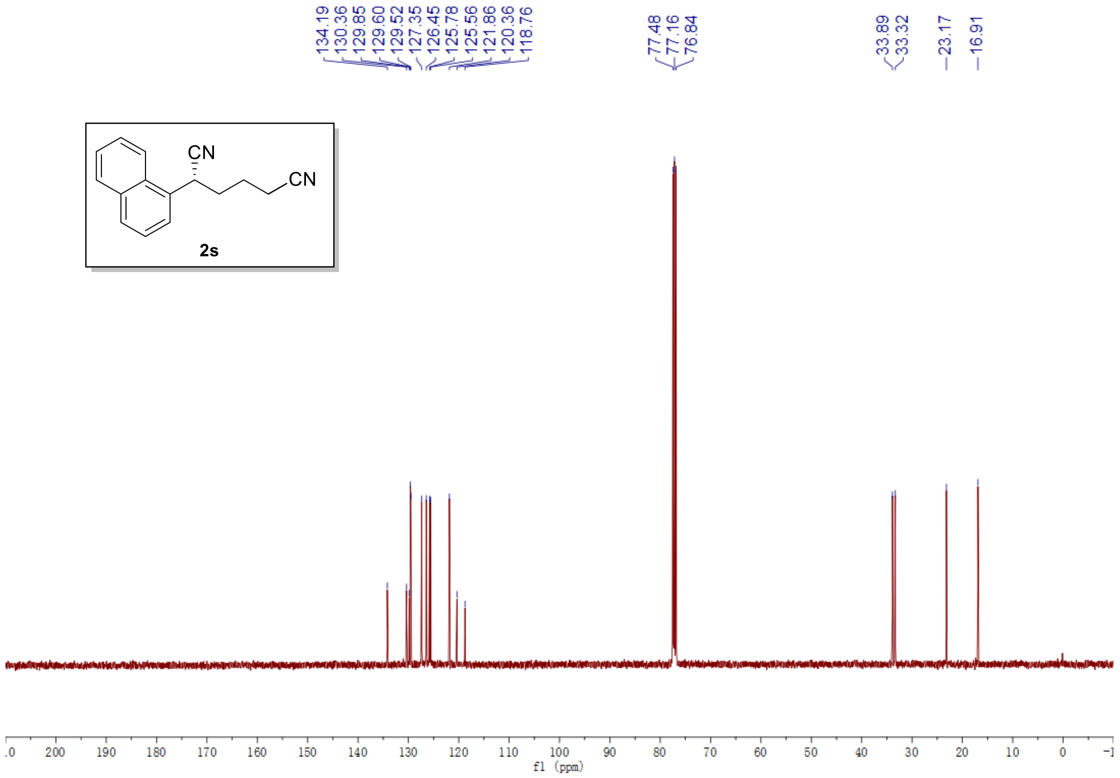

Supplementary Figure 98. ^{13}C NMR of **2s**

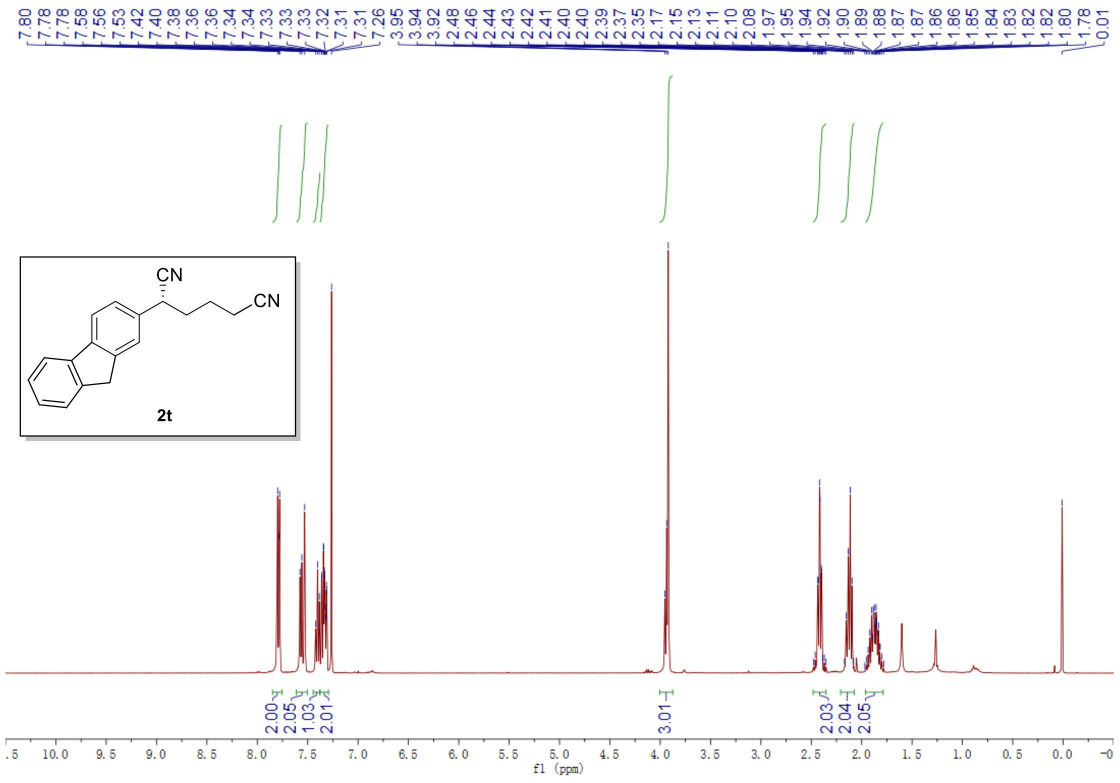

Supplementary Figure 99. ^{1}H NMR of **2t**

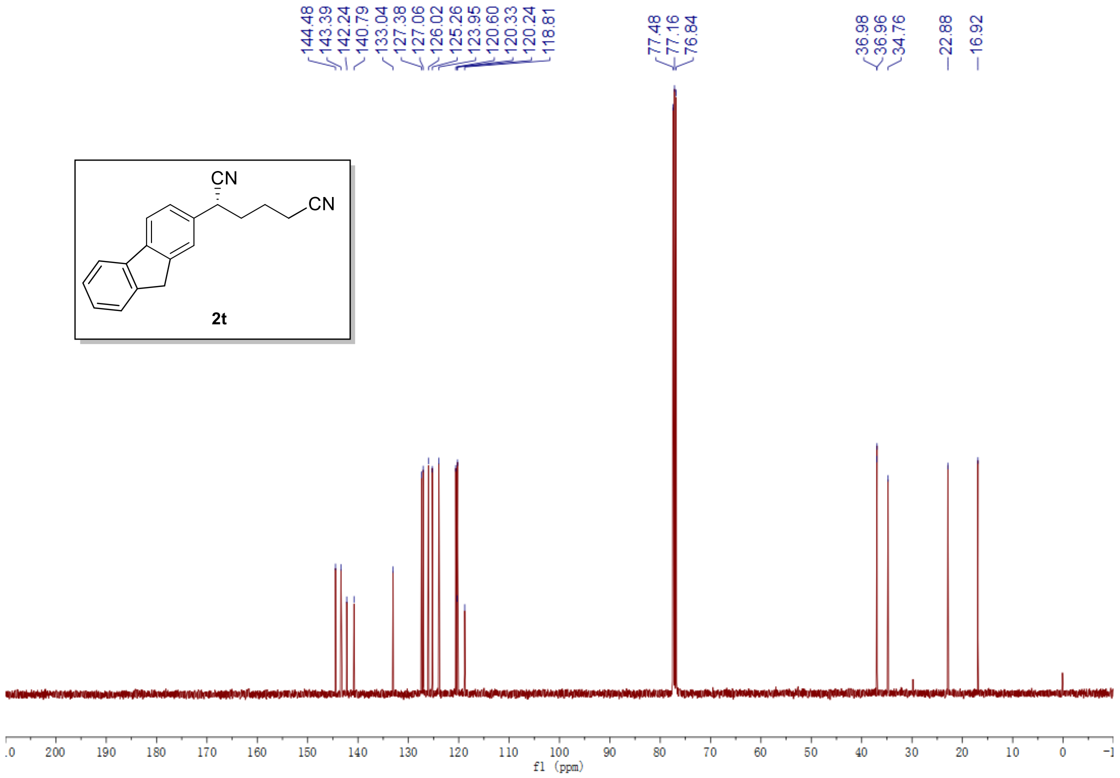

Supplementary Figure 100. ^{13}C NMR of **2t**

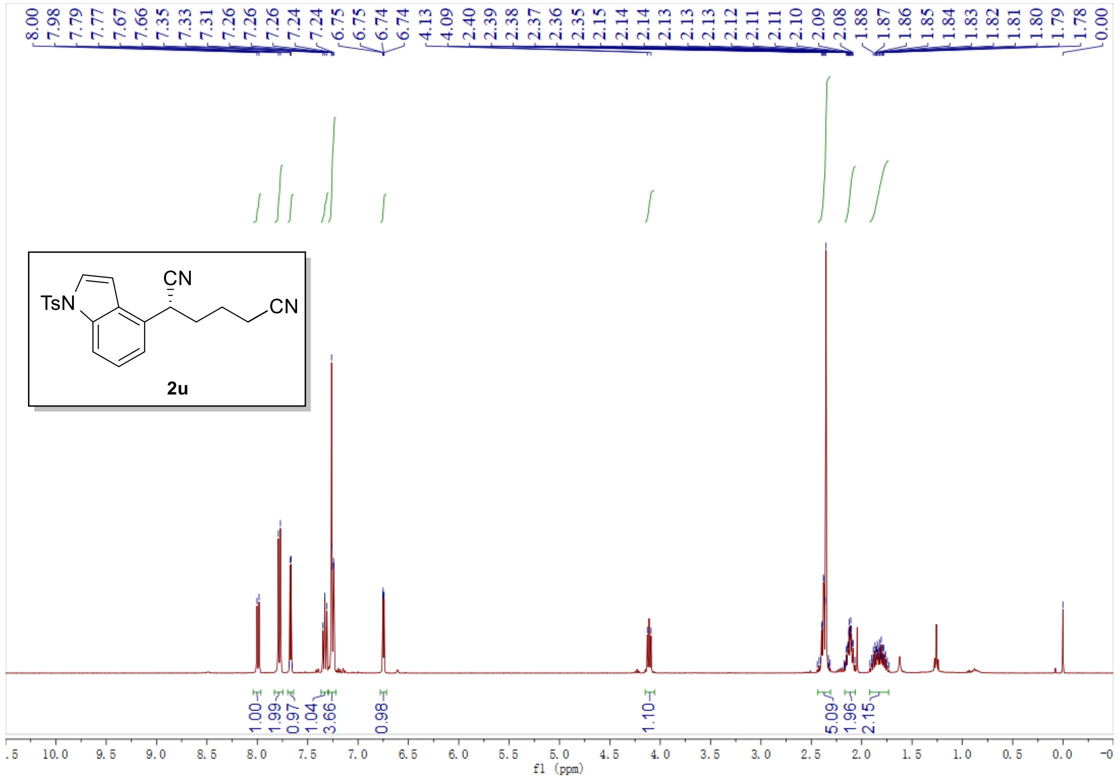

Supplementary Figure 101. ^{1}H NMR of **2u**

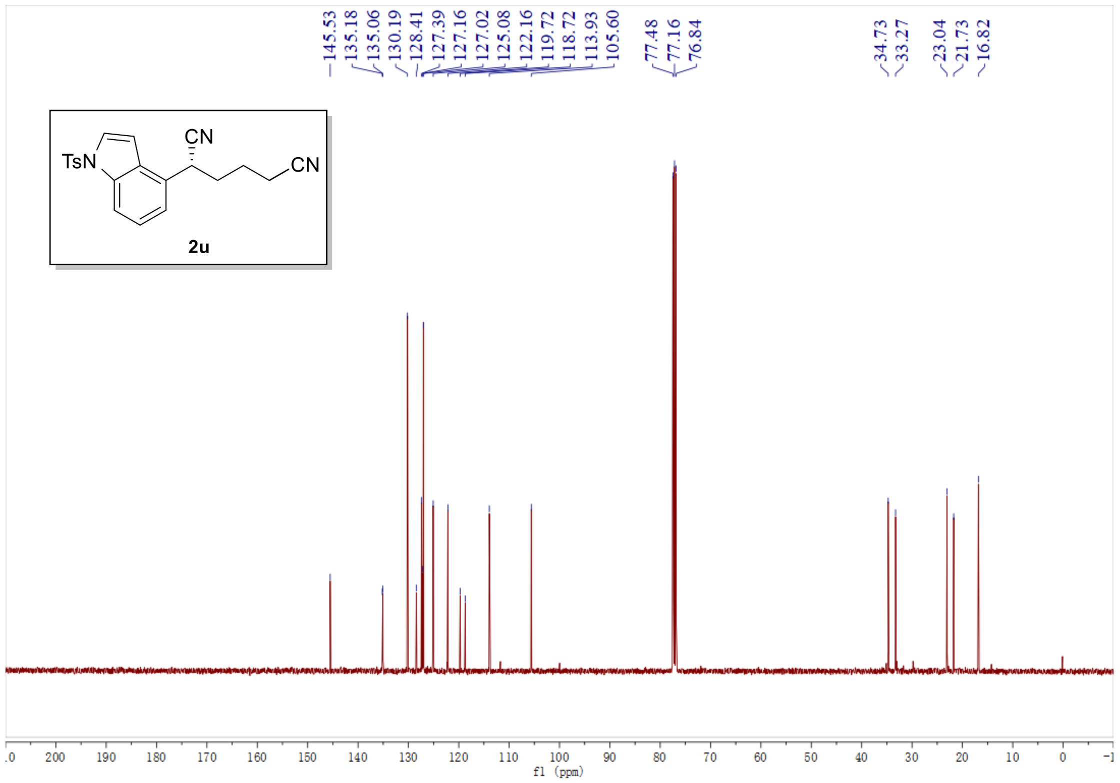

Supplementary Figure 102. ^{13}C NMR of **2u**

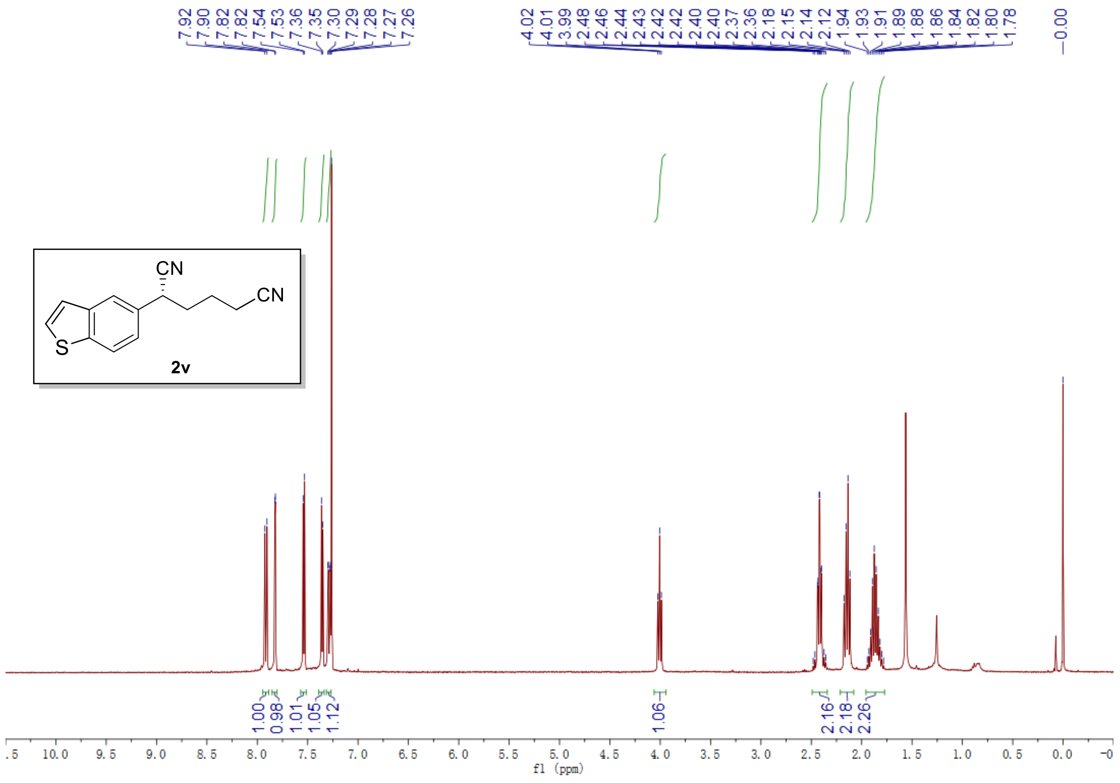

Supplementary Figure 103. ^{1}H NMR of **2v**

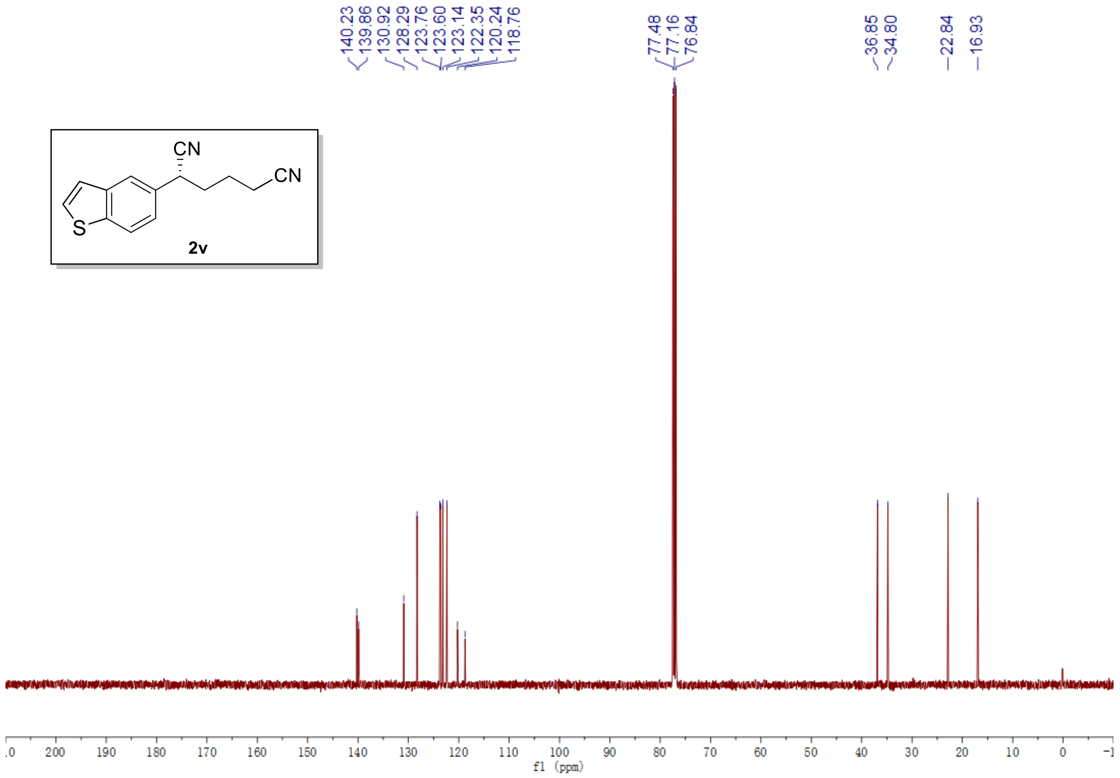

Supplementary Figure 104. ^{13}C NMR of **2v**

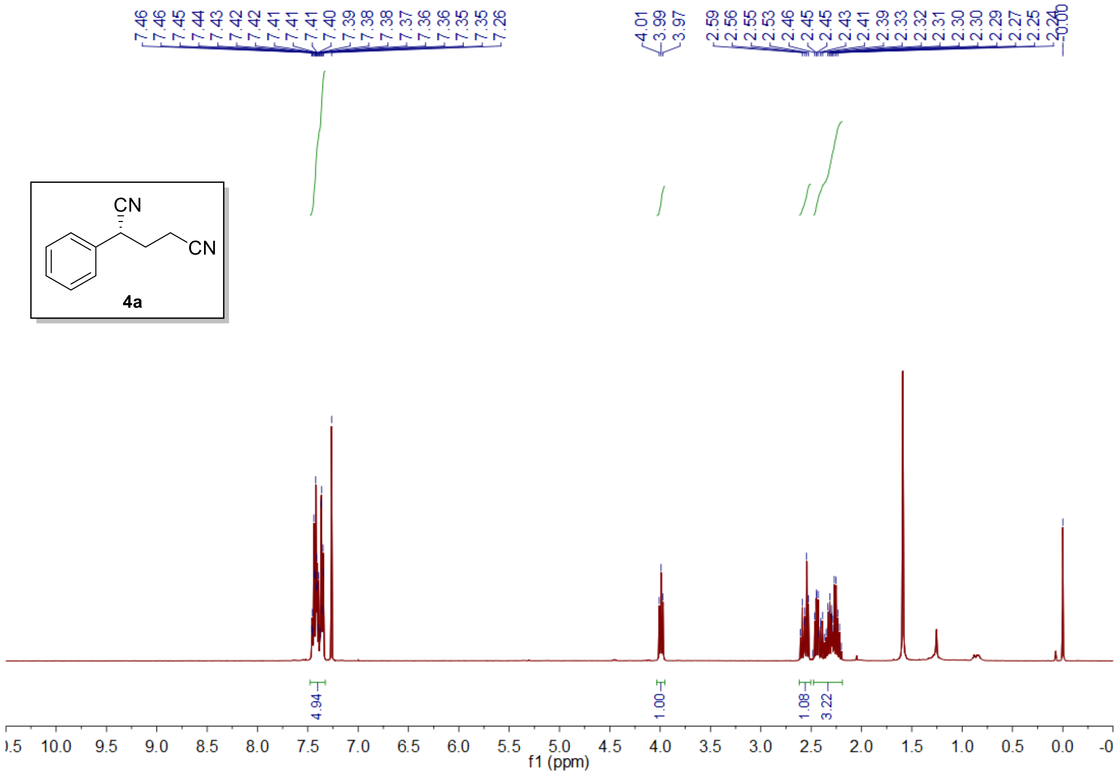

Supplementary Figure 105. ^{1}H NMR of **4a**

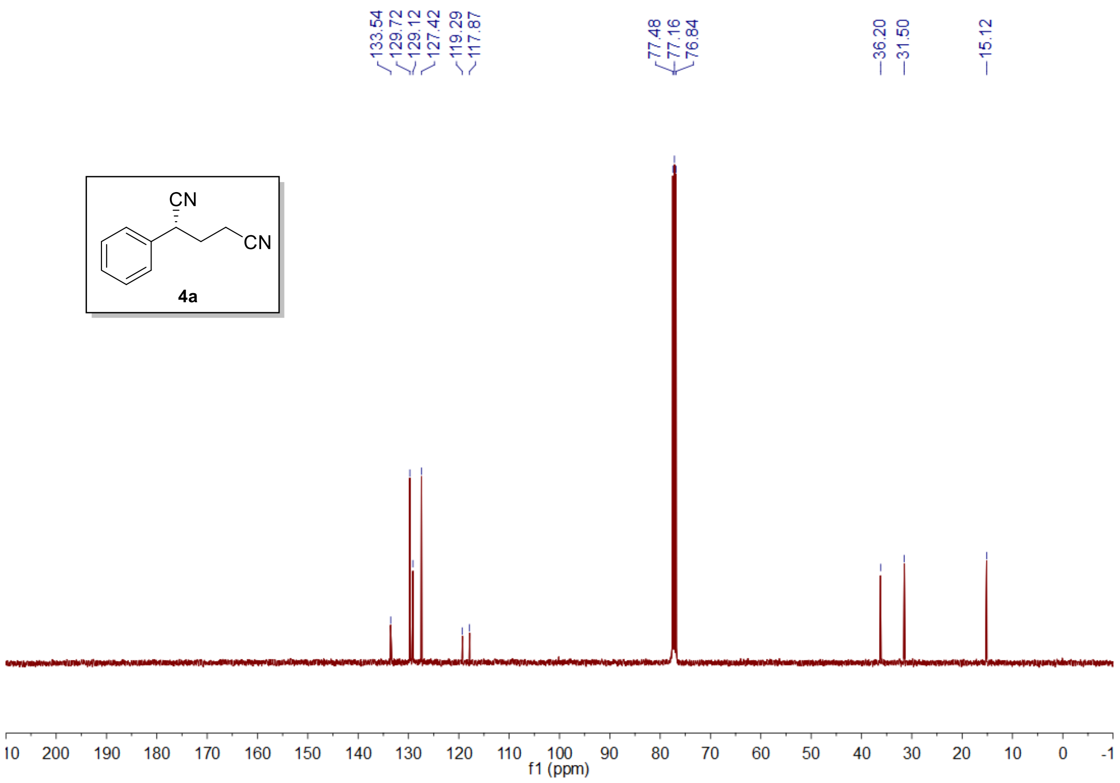

Supplementary Figure 106. ^{13}C NMR of **4a**

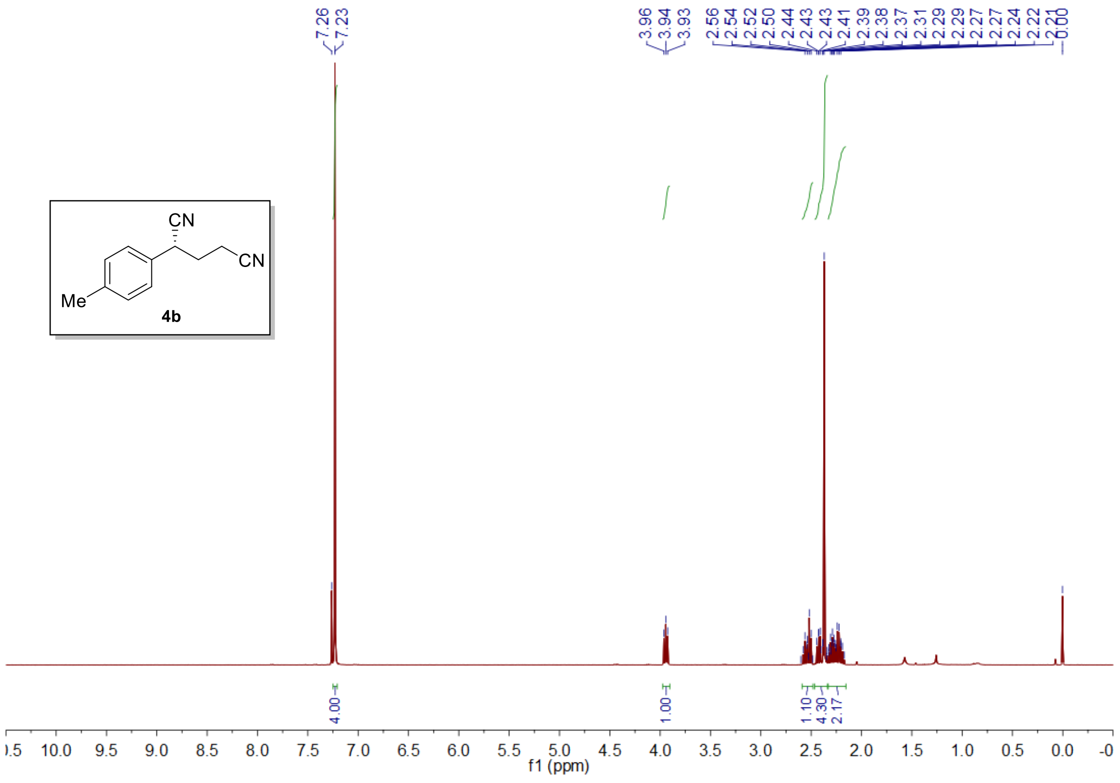

Supplementary Figure 107. ^{1}H NMR of **4b**

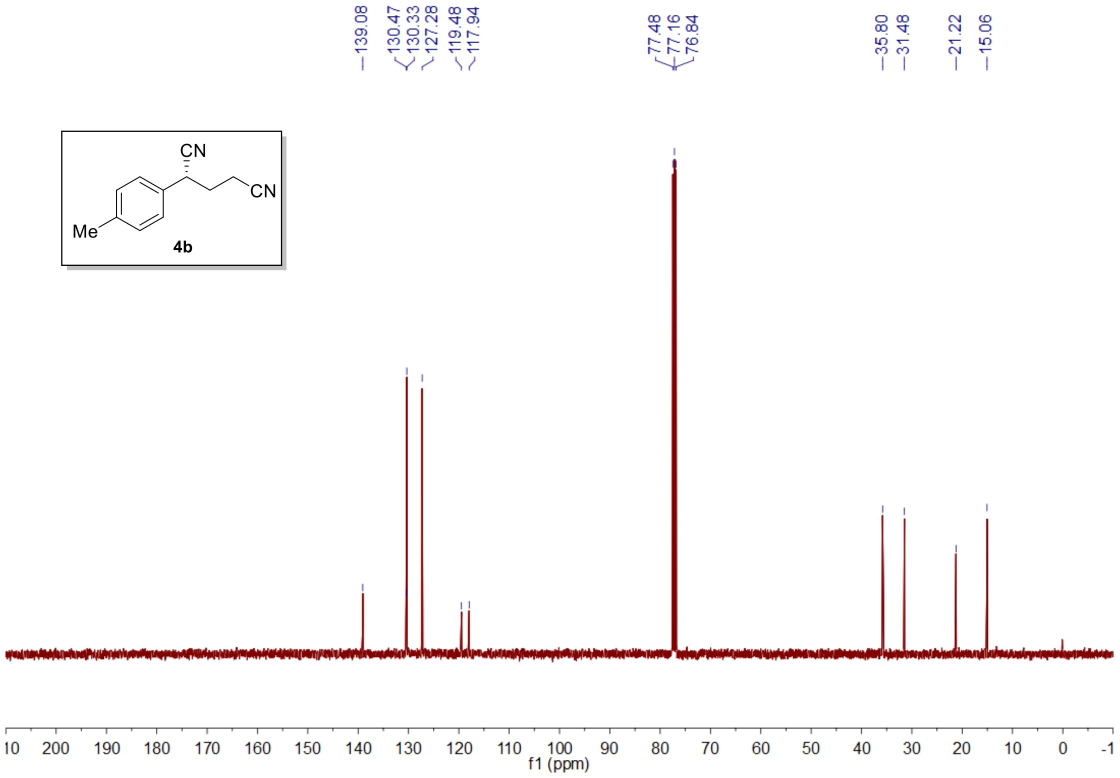

Supplementary Figure 108. ^{13}C NMR of **4b**

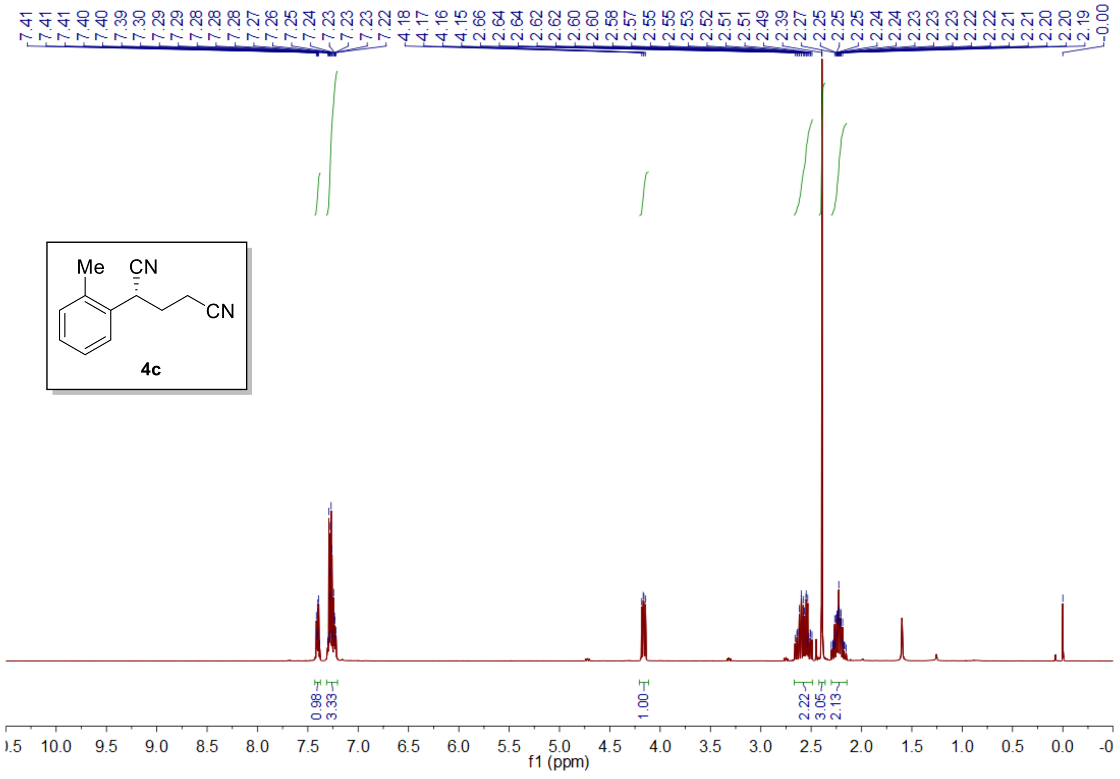

Supplementary Figure 109. ^{1}H NMR of **4c**

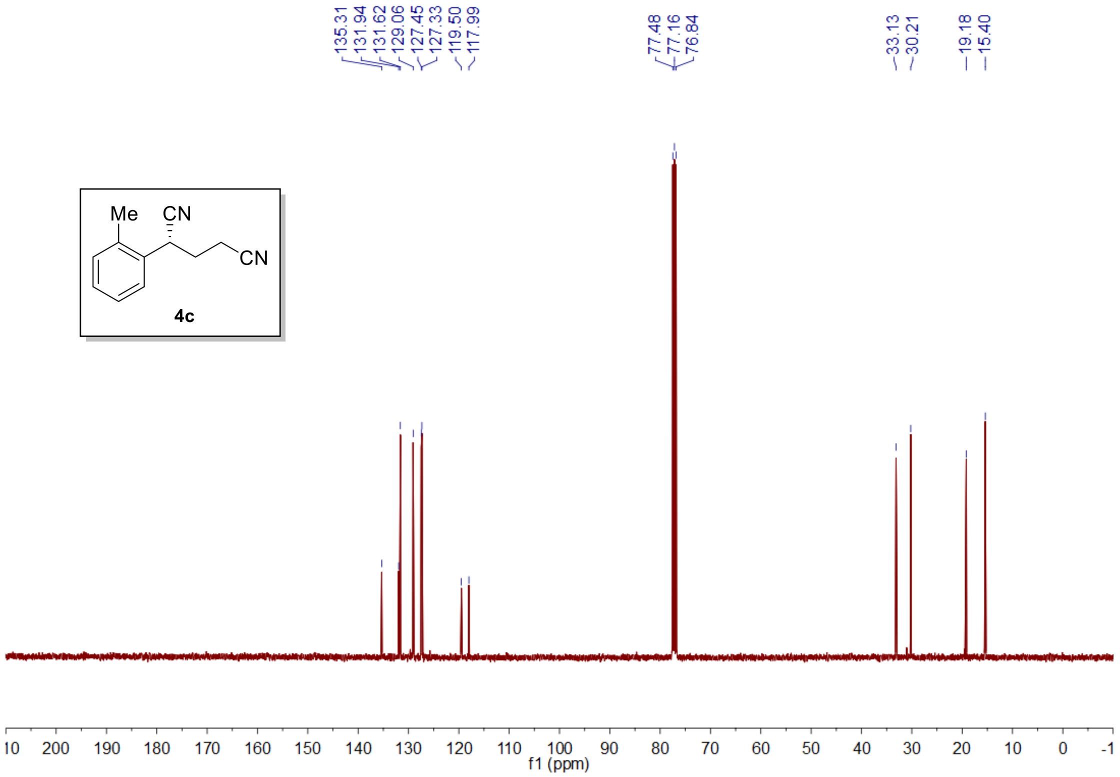

Supplementary Figure 110. ^{13}C NMR of **4c**

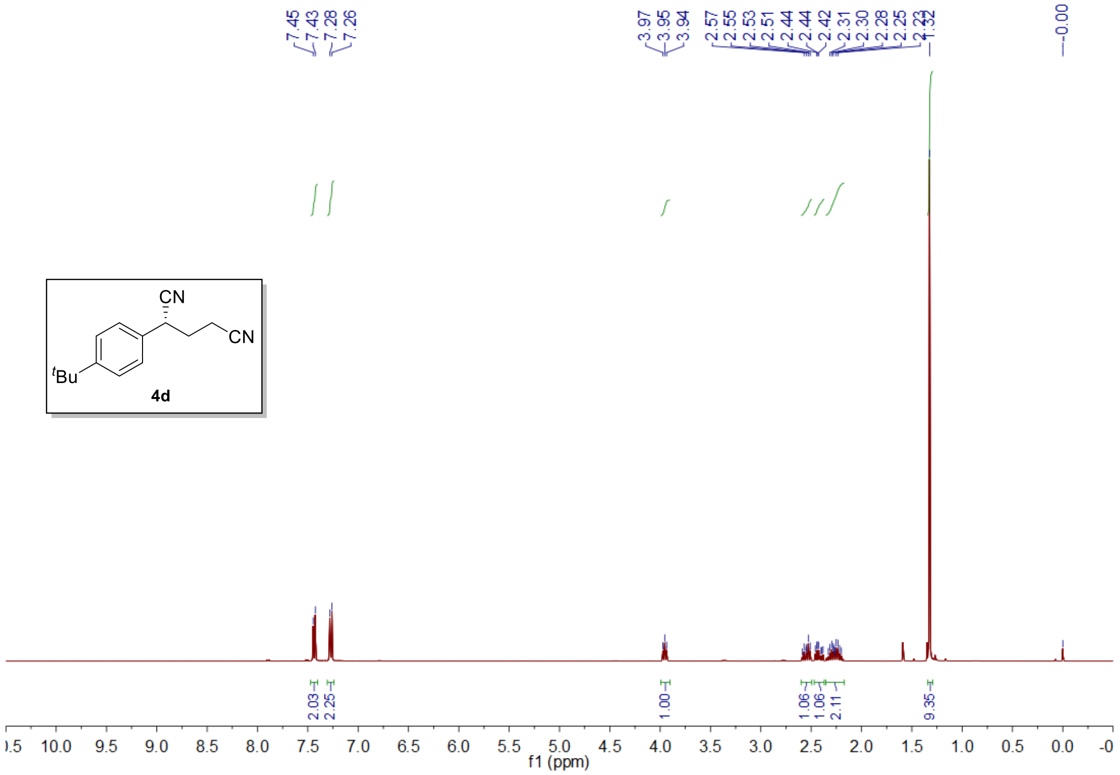

Supplementary Figure 111. ^{1}H NMR of **4d**

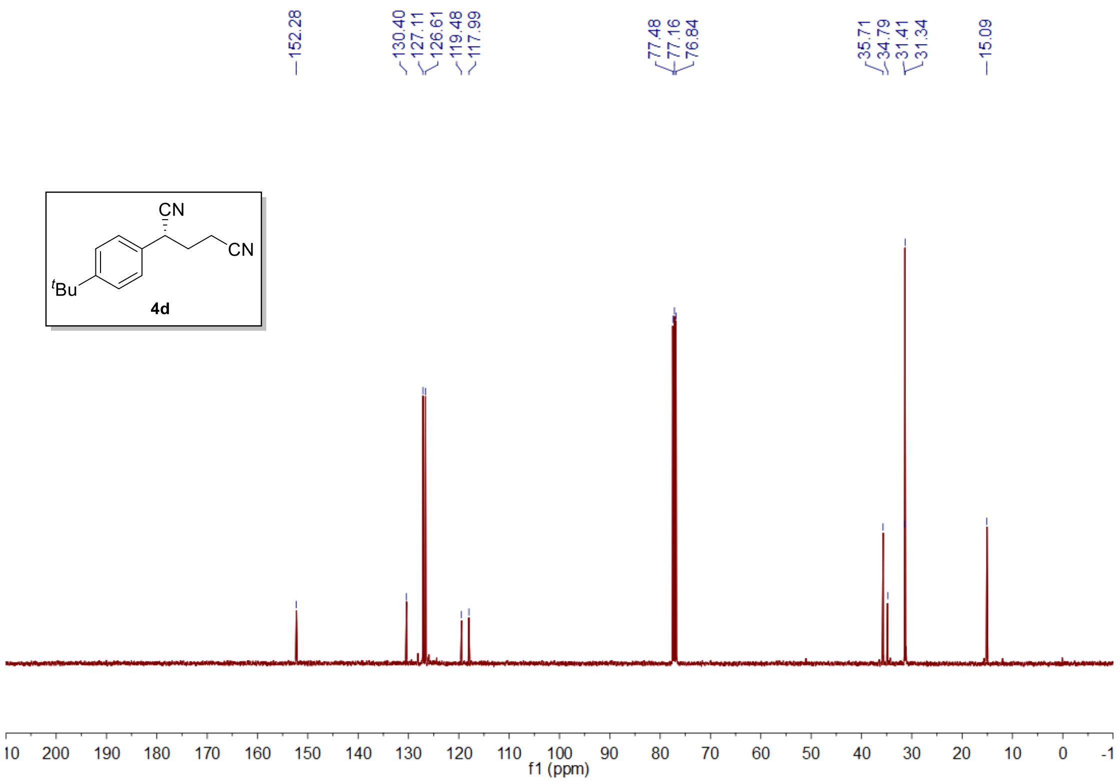

Supplementary Figure 112. ^{13}C NMR of **4d**

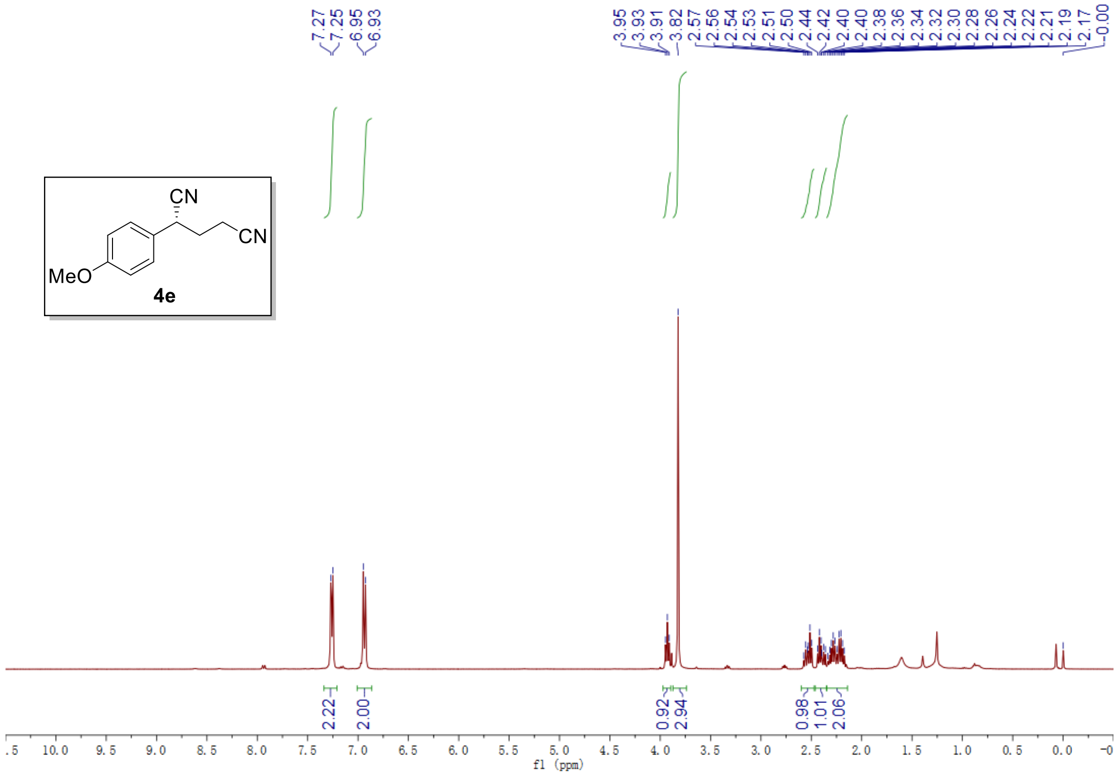

Supplementary Figure 113. ^{1}H NMR of **4e**

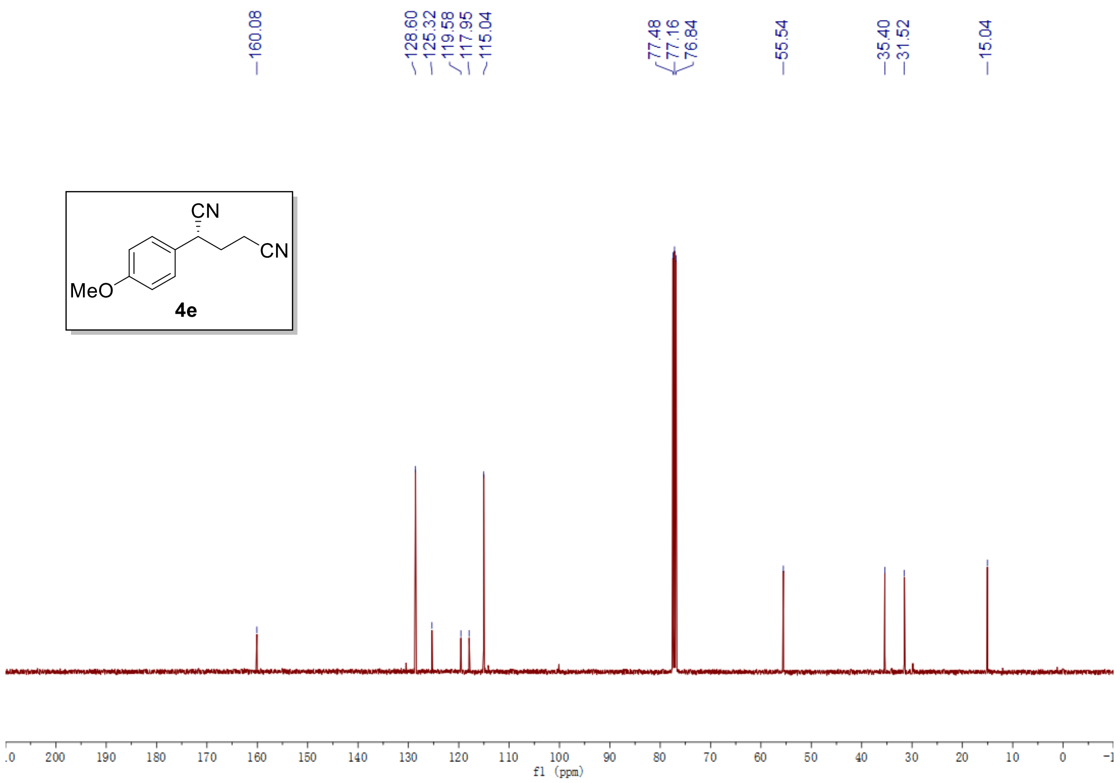

Supplementary Figure 114. ^{13}C NMR of **4e**

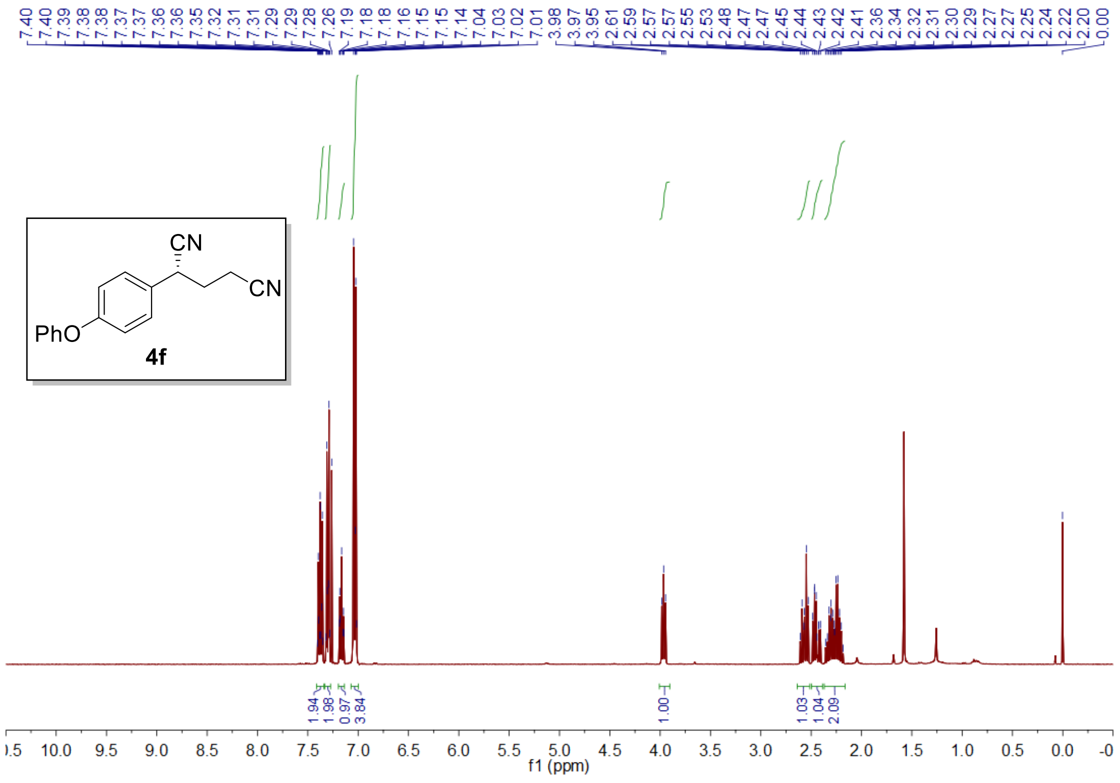

Supplementary Figure 115. ^{1}H NMR of **4f**

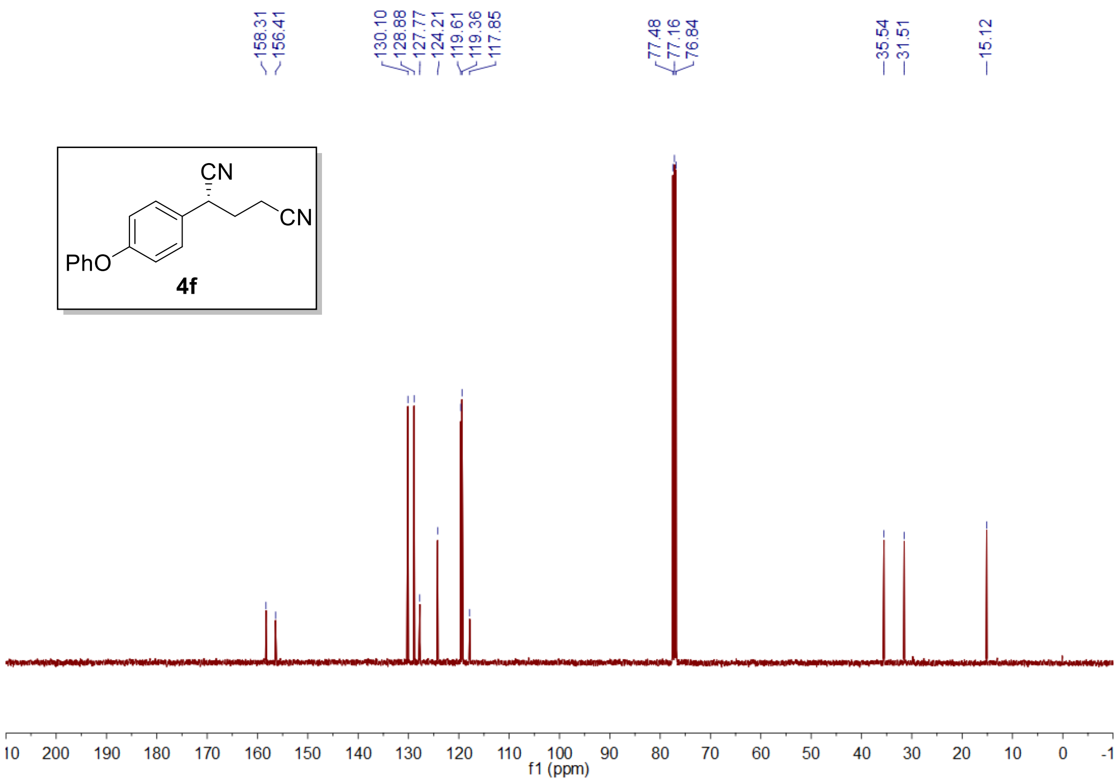

Supplementary Figure 116. ^{13}C NMR of **4f**

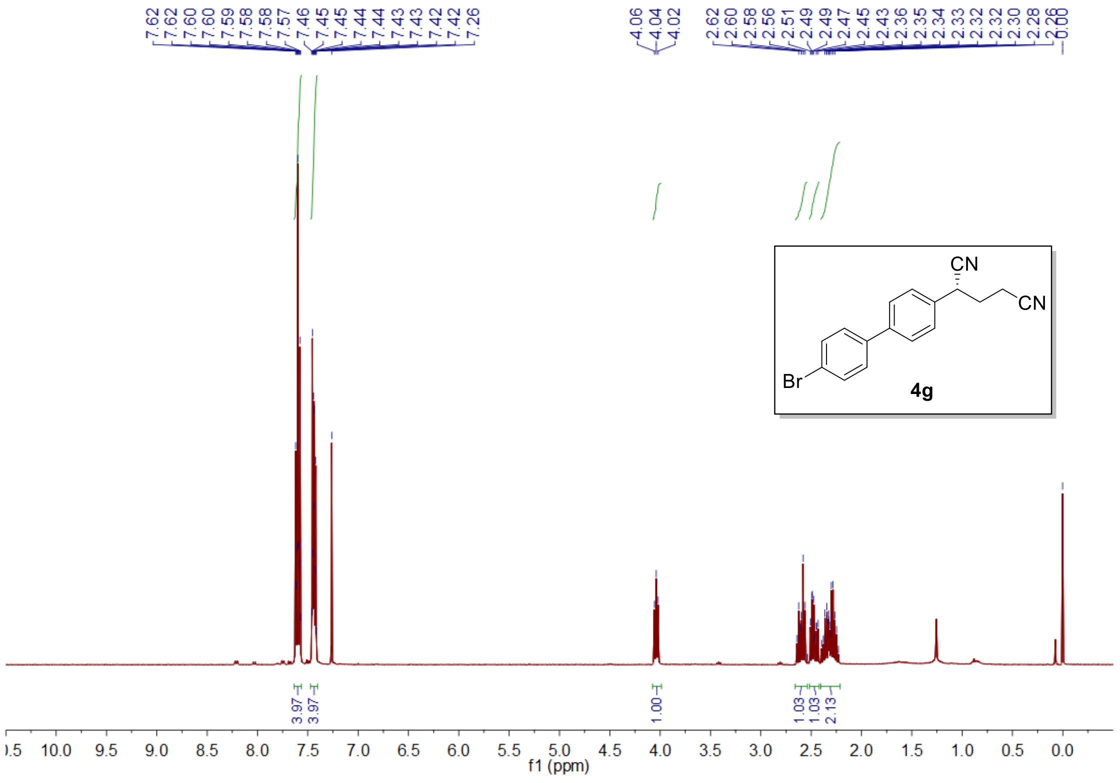

Supplementary Figure 117. ^{1}H NMR of **4g**

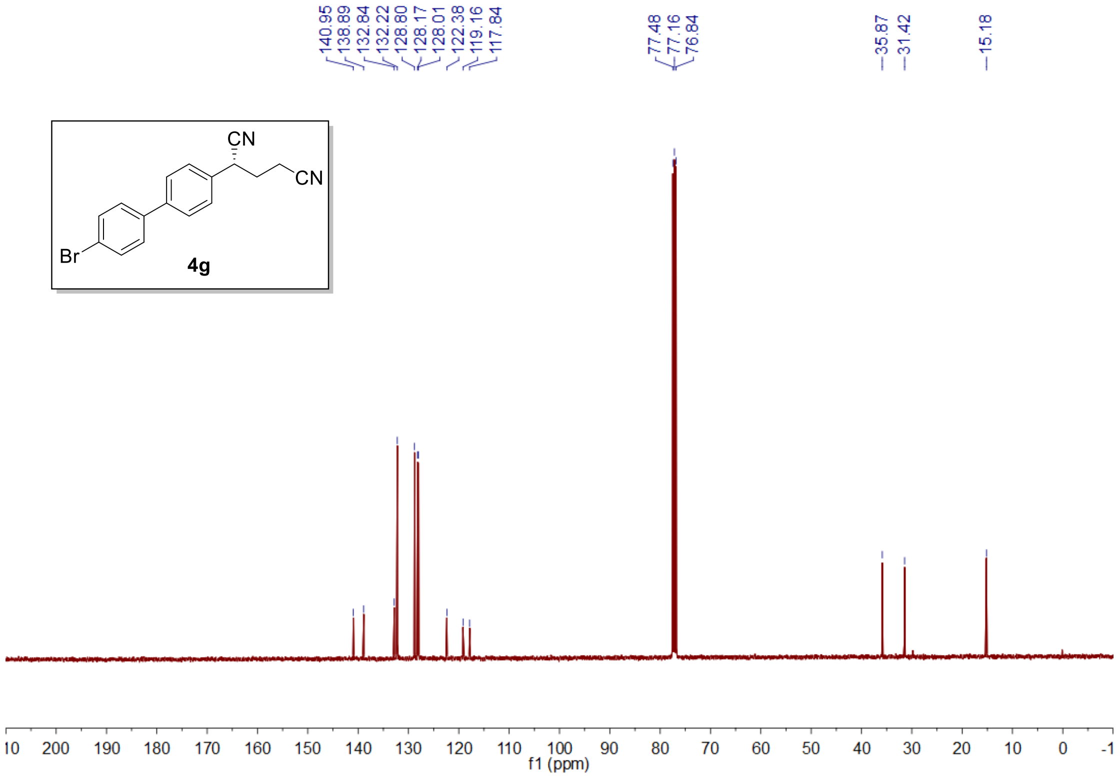

Supplementary Figure 118. ^{13}C NMR of **4g**

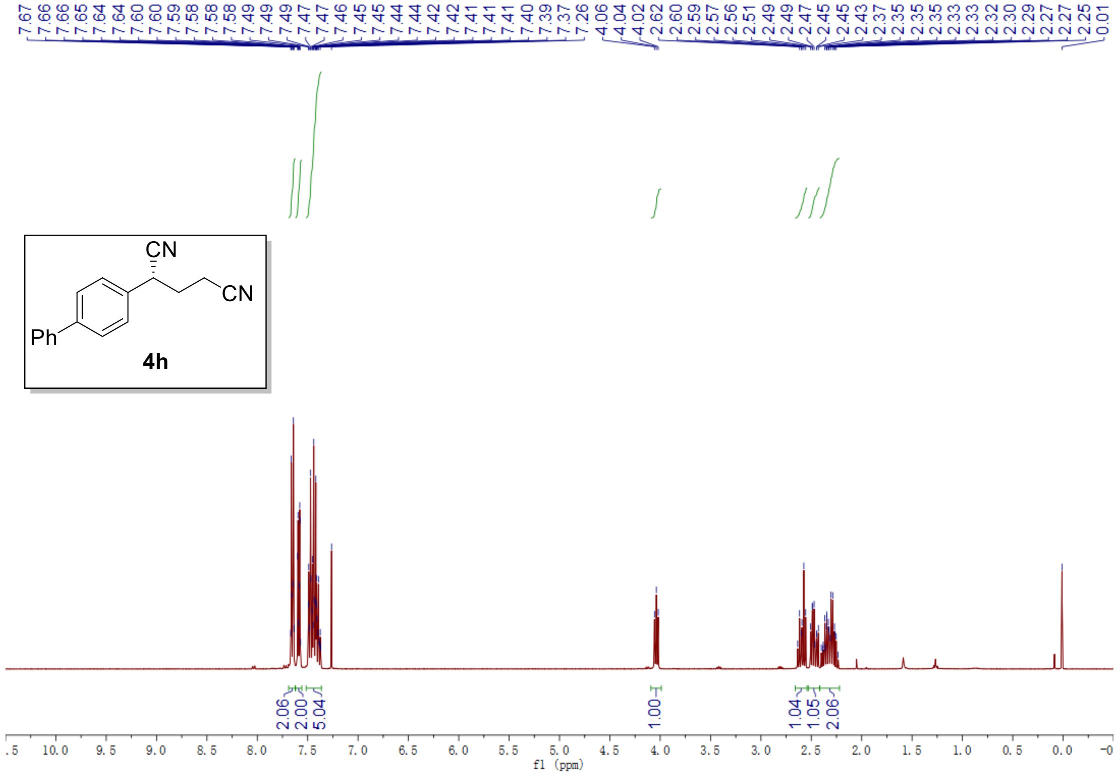

Supplementary Figure 119. ^{1}H NMR of **4h**

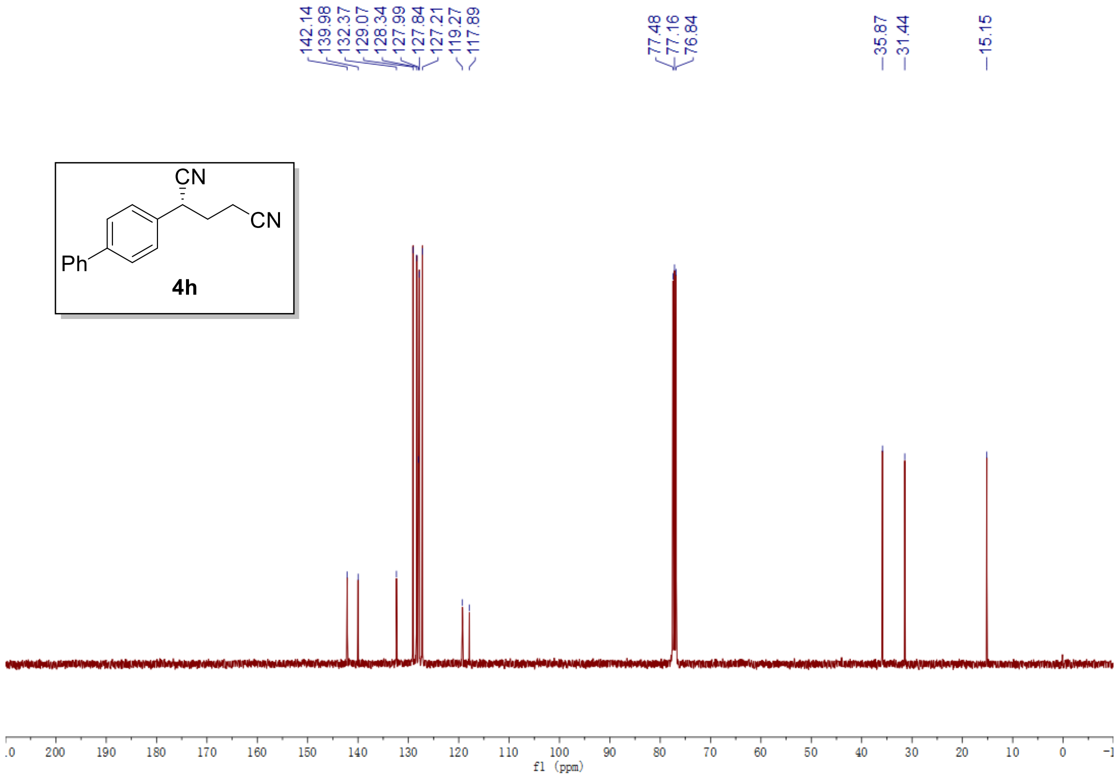

Supplementary Figure 120. ^{13}C NMR of **4h**

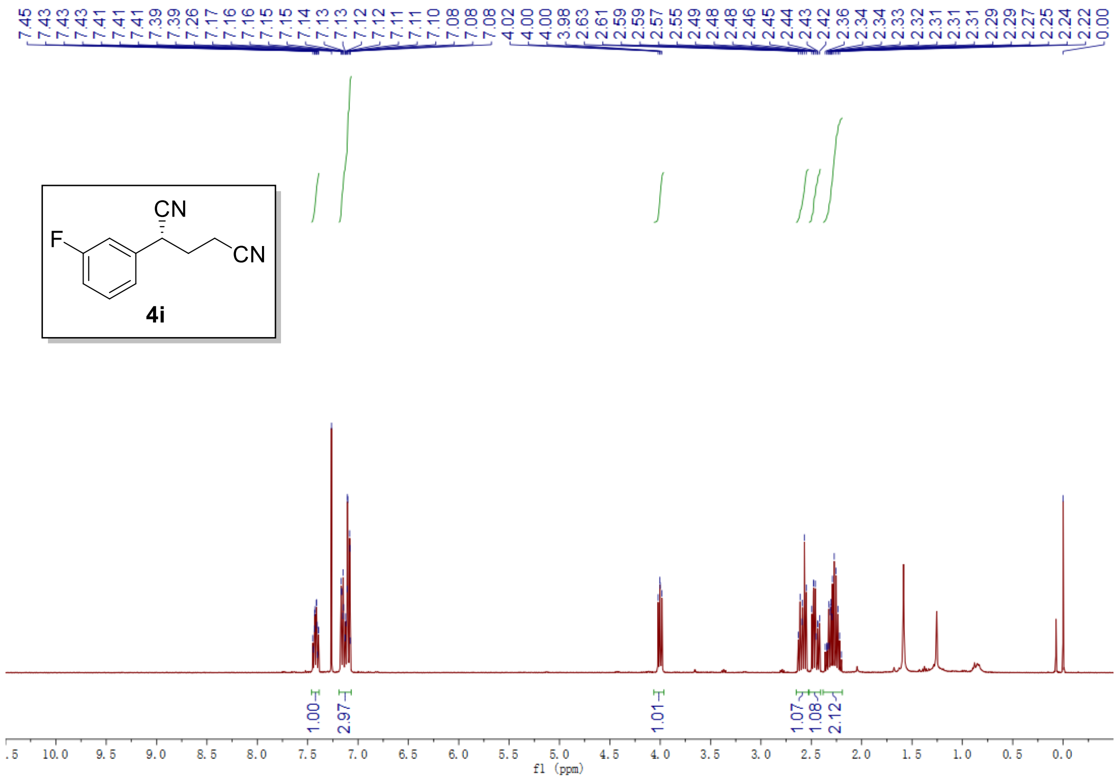

Supplementary Figure 121. ^{1}H NMR of **4i**

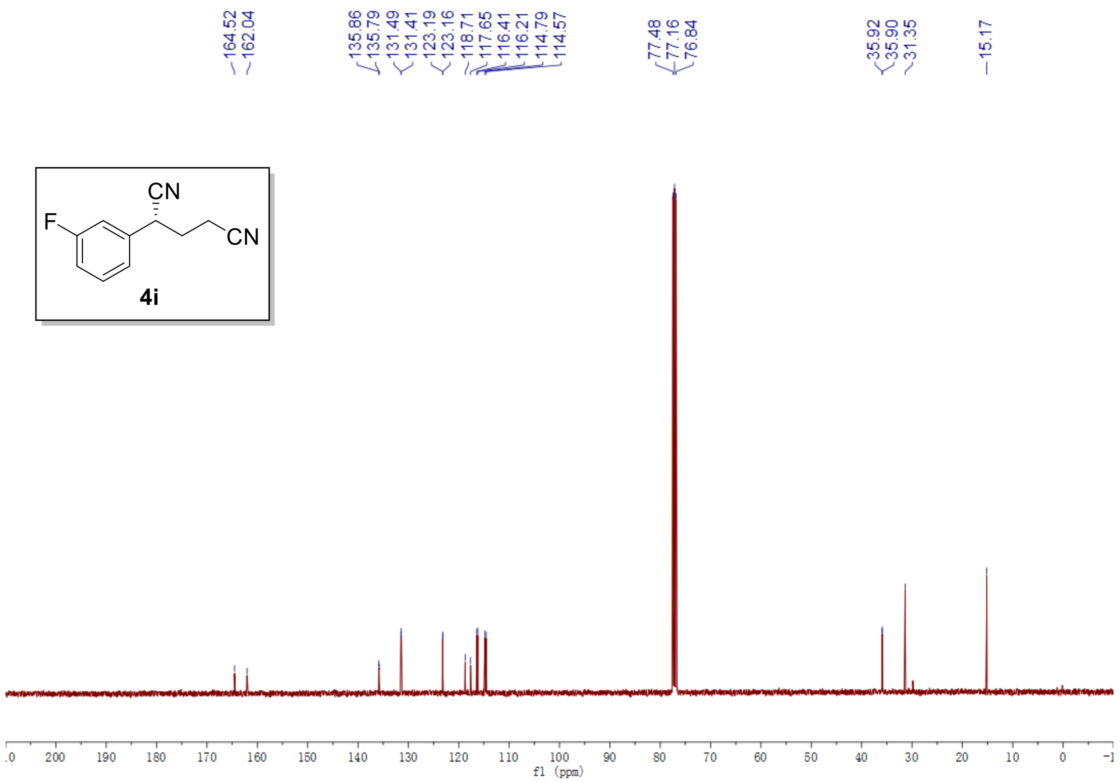

Supplementary Figure 122. ^{13}C NMR of **4i**

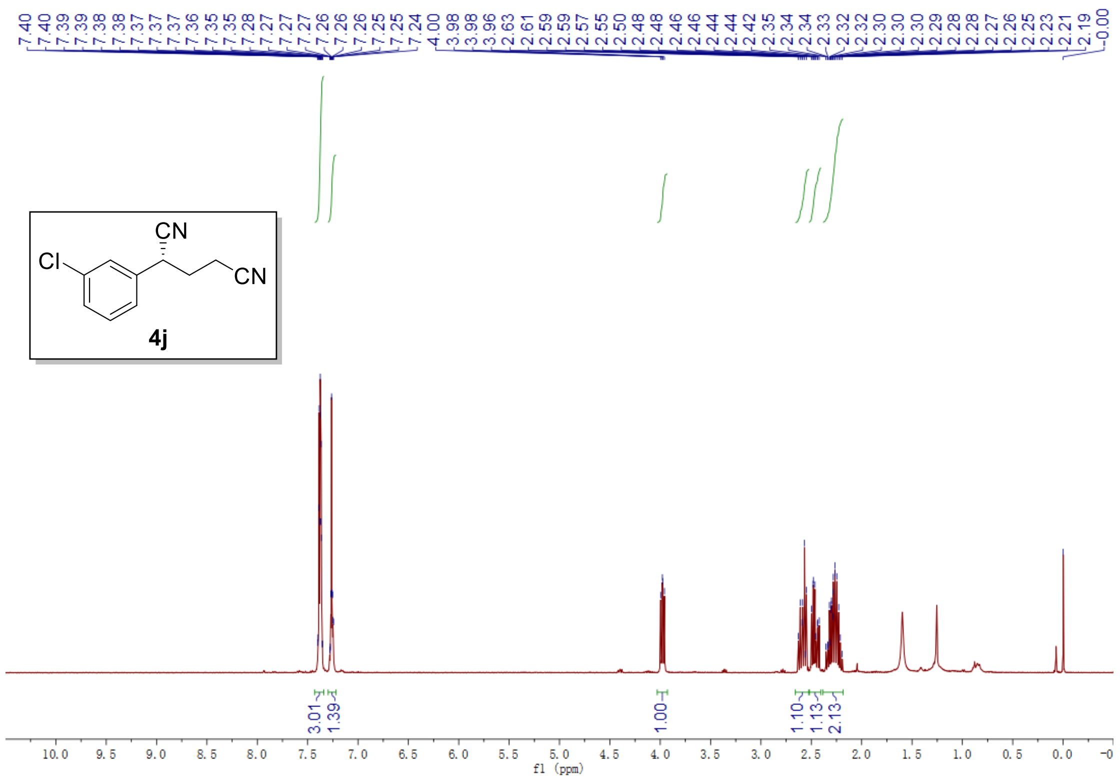

Supplementary Figure 123. ^{1}H NMR of **4j**

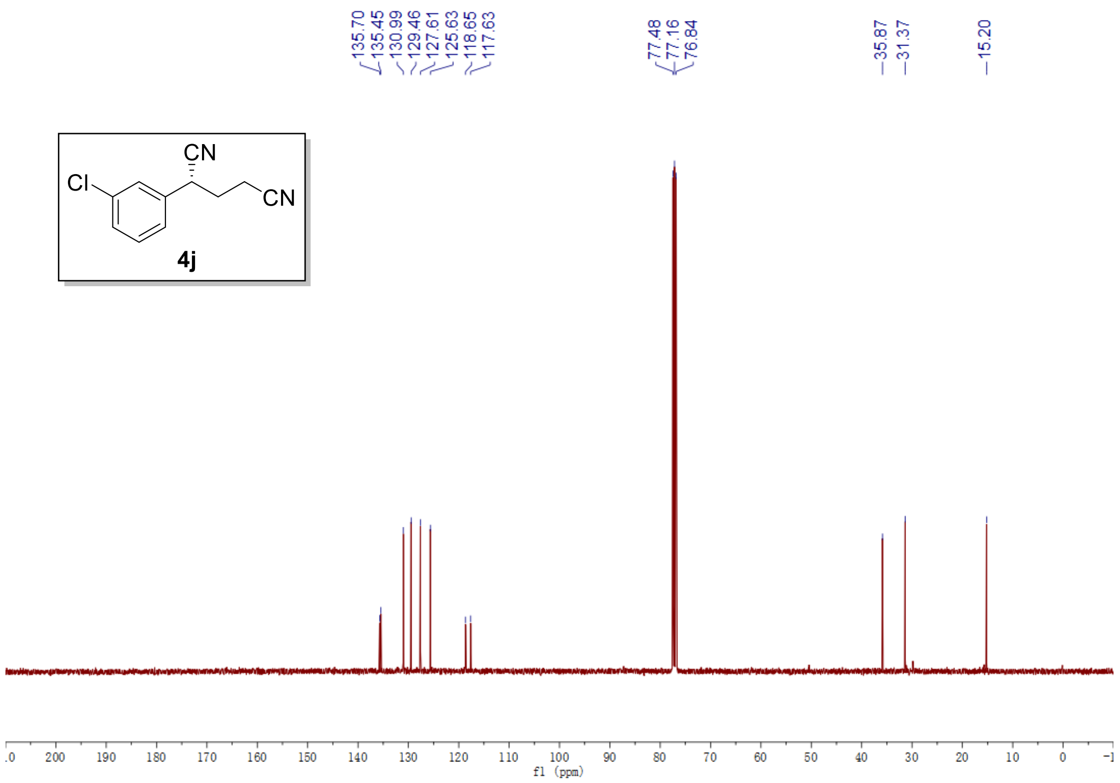

Supplementary Figure 124. ^{13}C NMR of **4j**

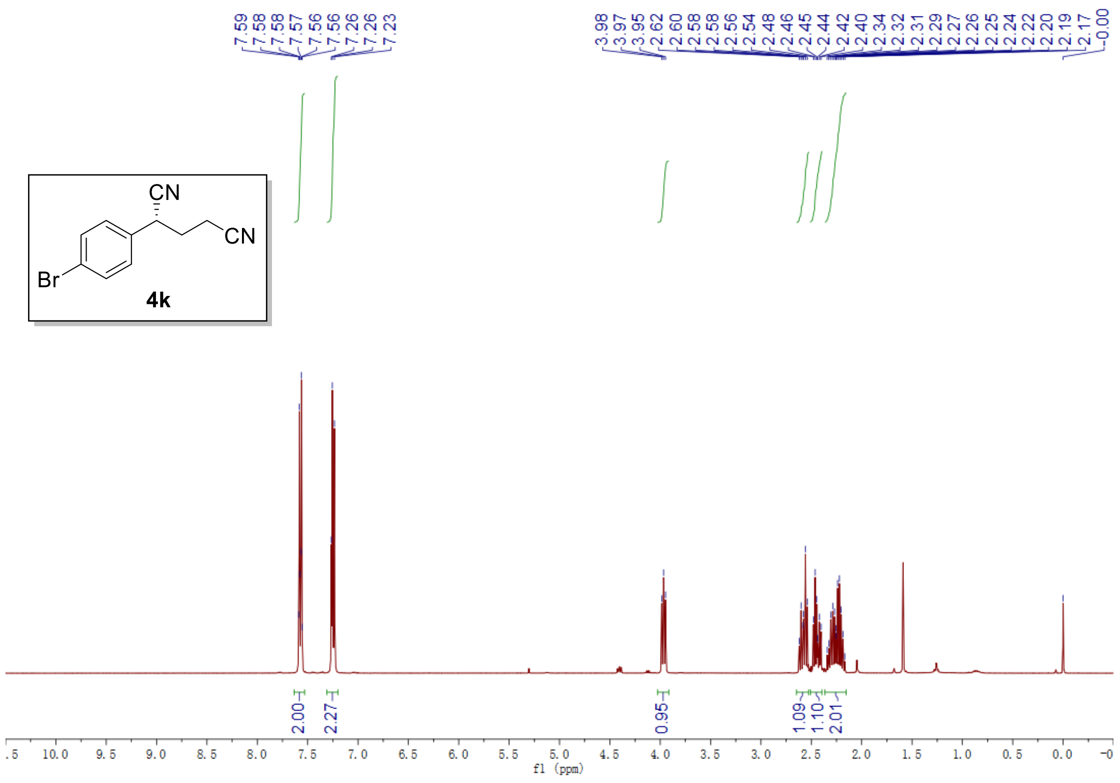

Supplementary Figure 125. ^{1}H NMR of **4k**

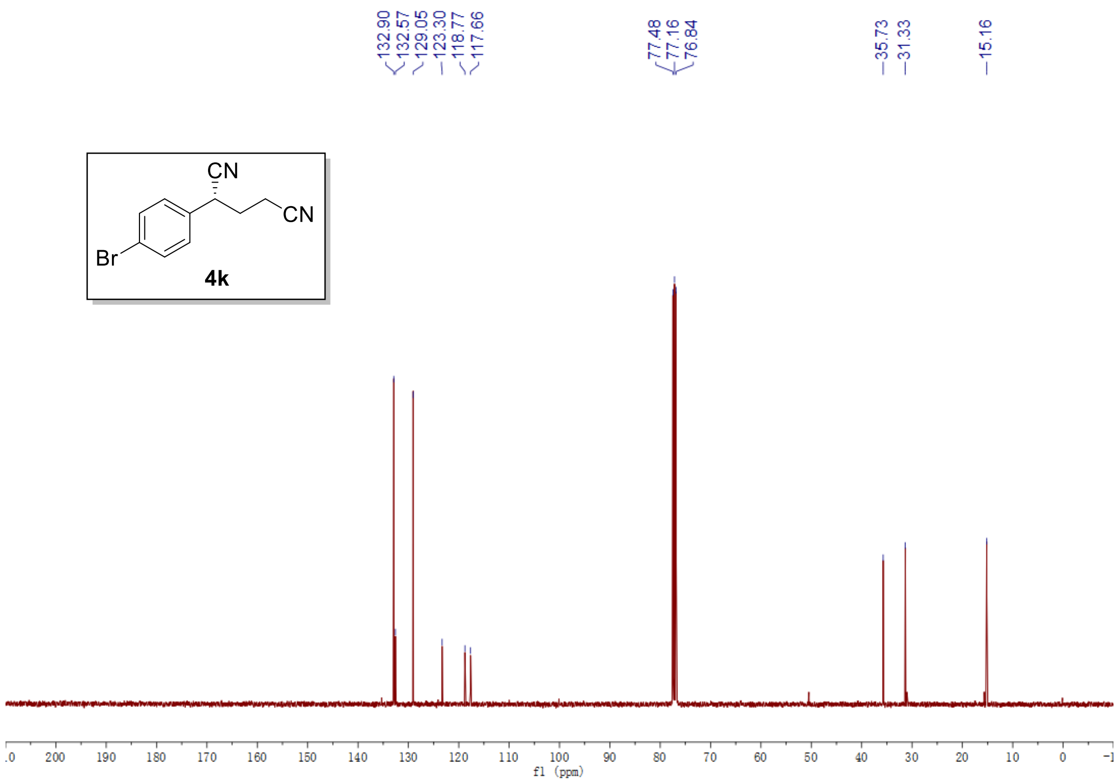

Supplementary Figure 126. ^{13}C NMR of **4k**

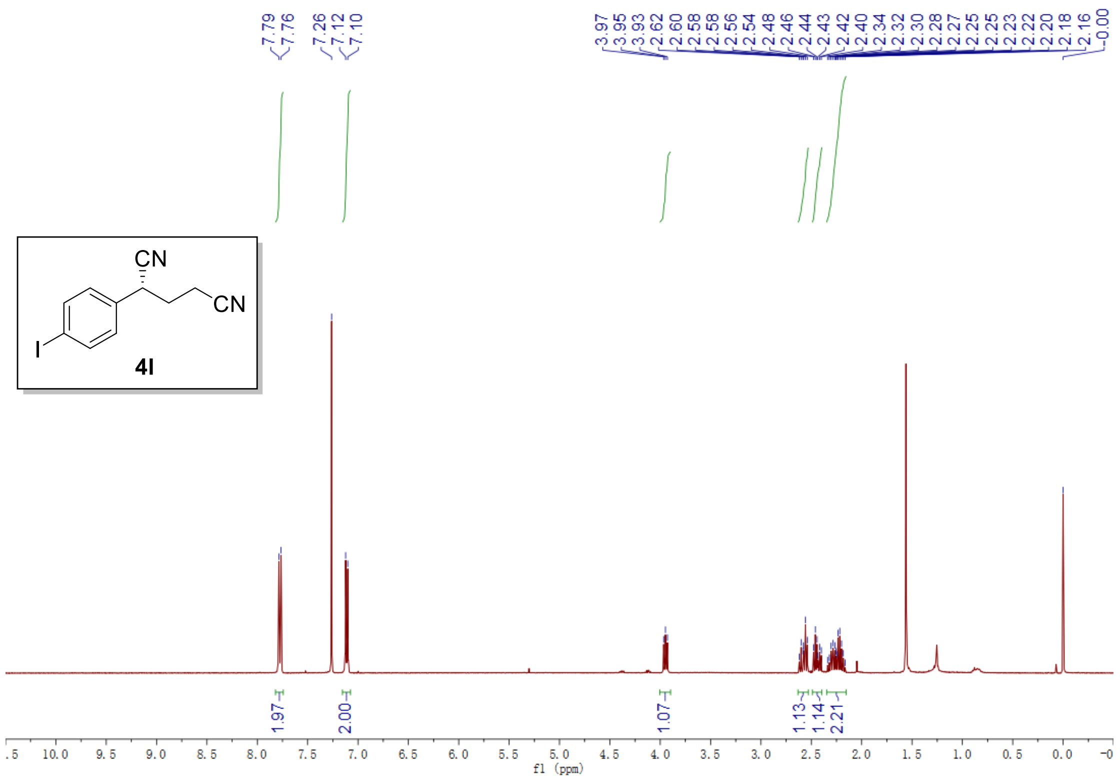

Supplementary Figure 127. ^{1}H NMR of **4l**

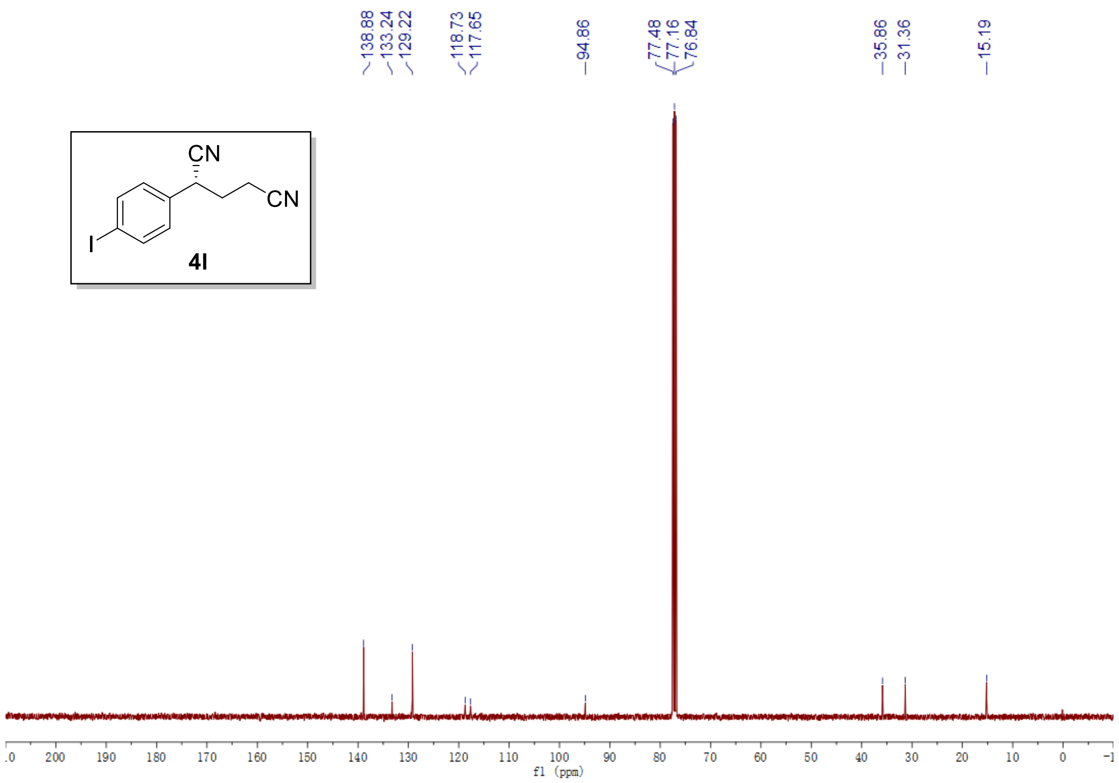

Supplementary Figure 128. ^{13}C NMR of **4l**

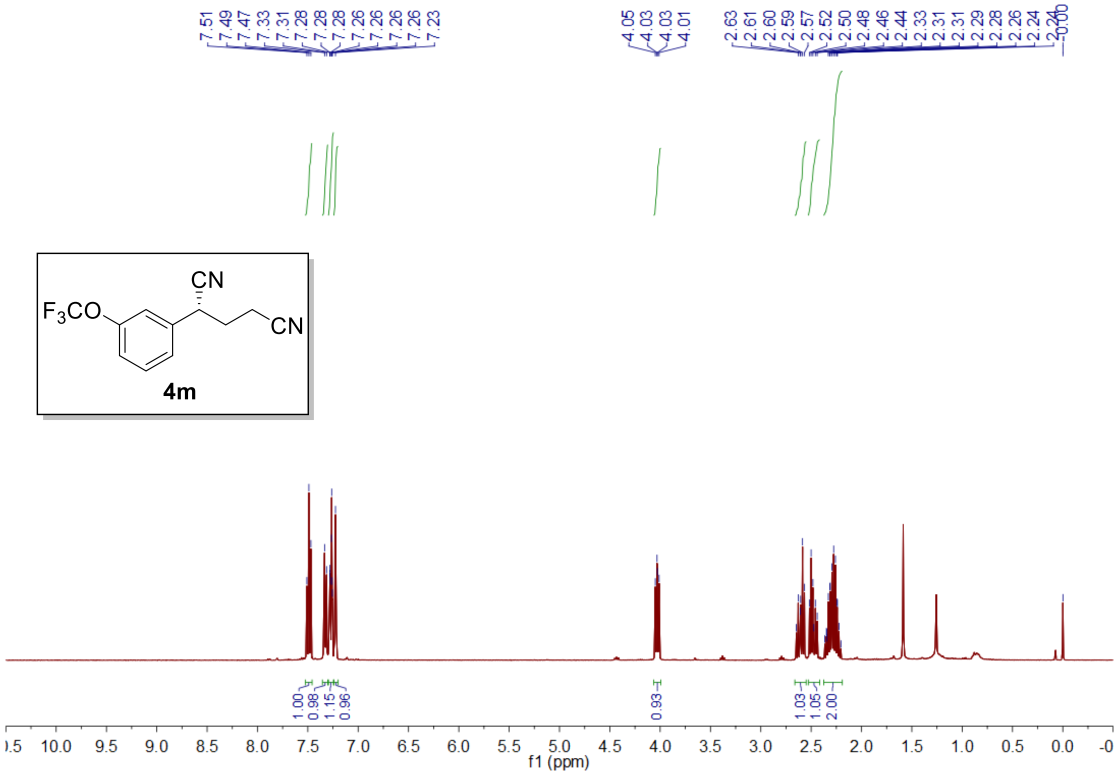

Supplementary Figure 129. ^{1}H NMR of **4m**

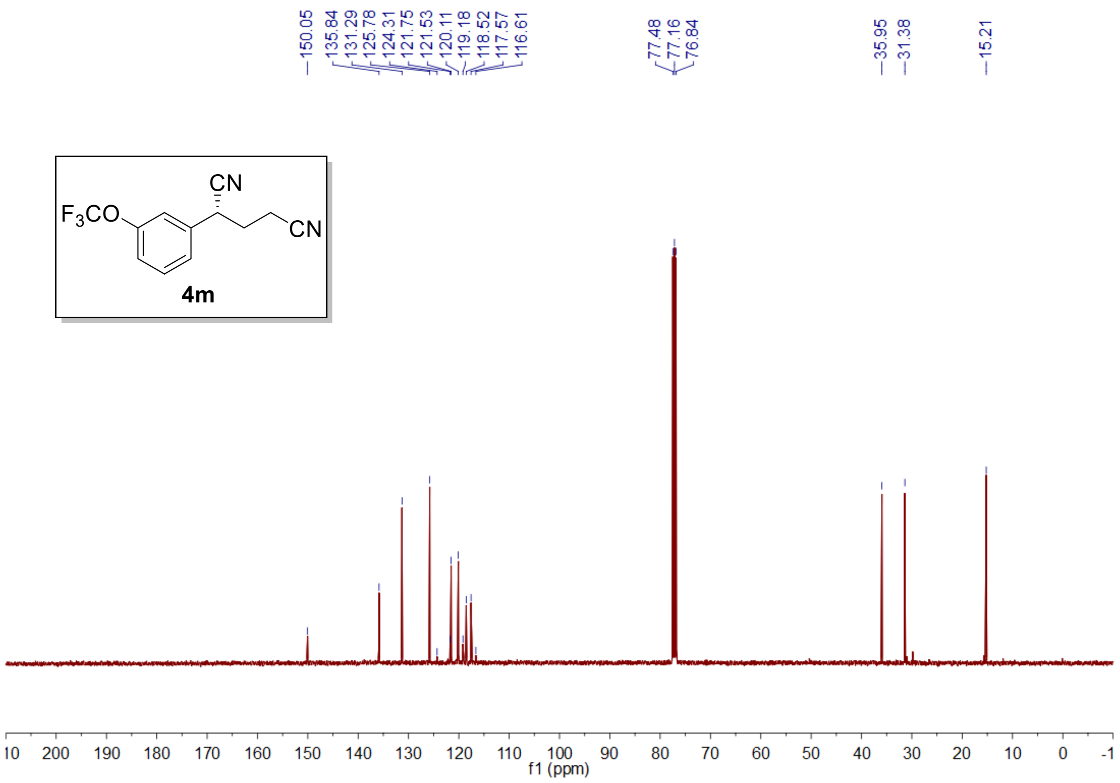

Supplementary Figure 130. ^{13}C NMR of **4m**

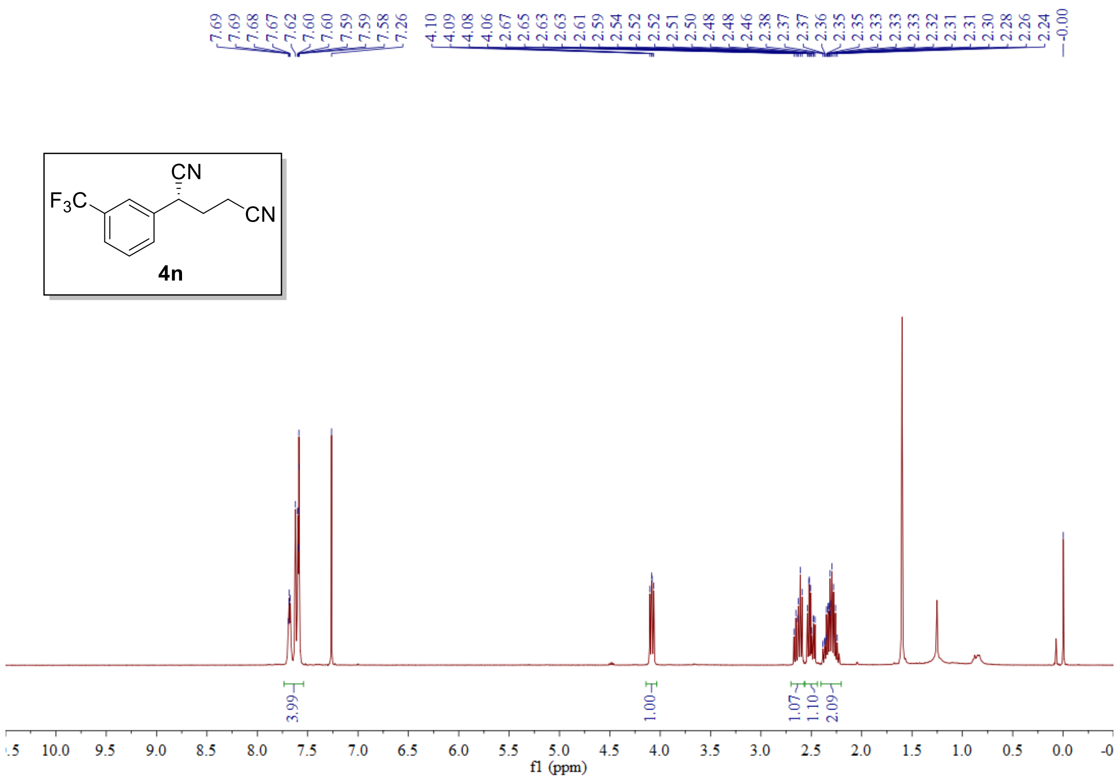

Supplementary Figure 131. ^{1}H NMR of **4n**

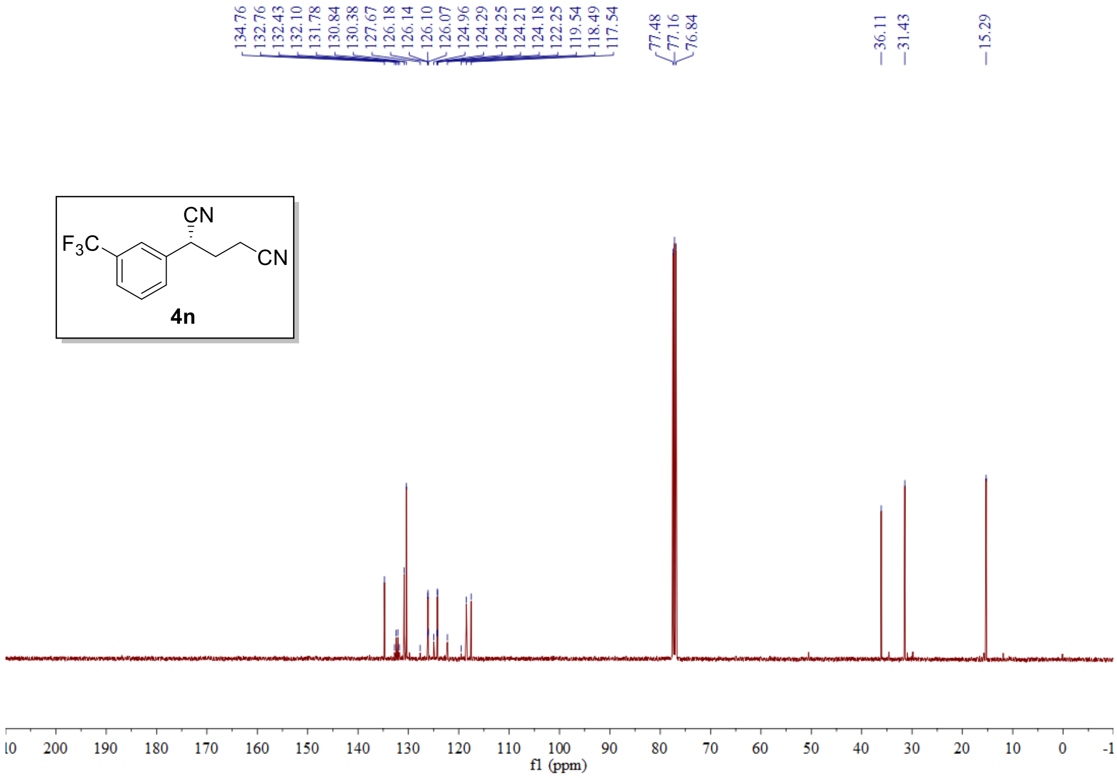

Supplementary Figure 132. ^{13}C NMR of **4n**

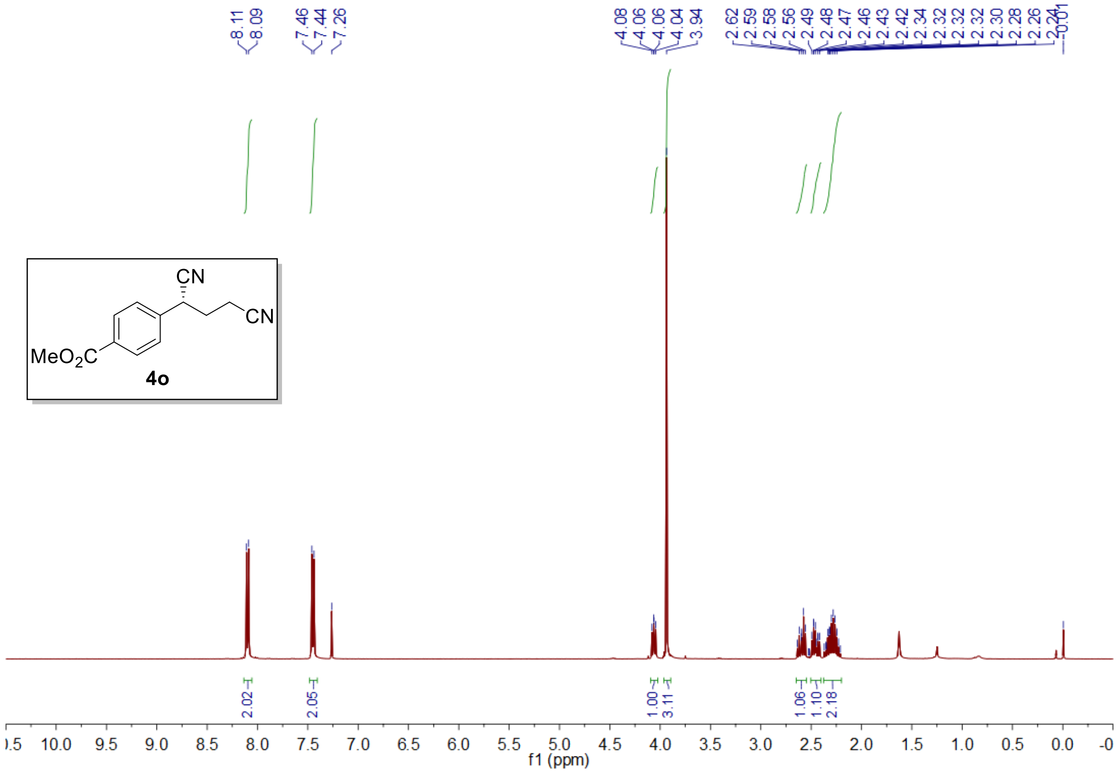

Supplementary Figure 133. ^{1}H NMR of **4o**

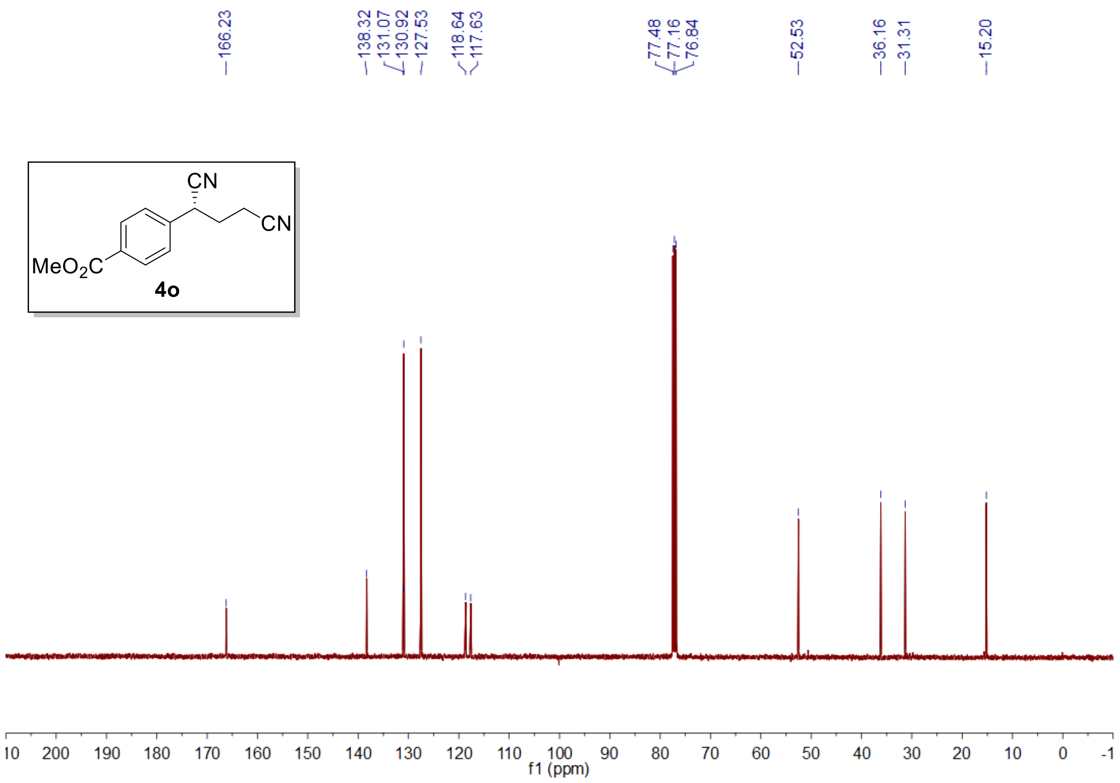

Supplementary Figure 134. ^{13}C NMR of **4o**

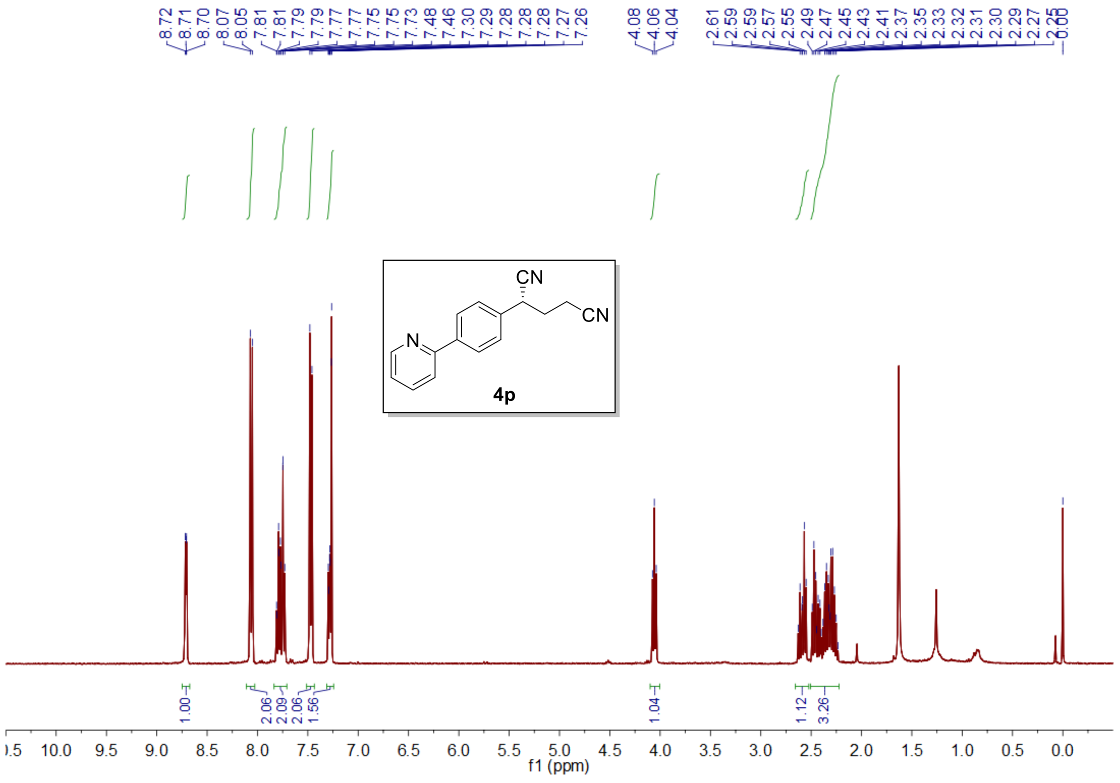

Supplementary Figure 135. ^{1}H NMR of **4p**

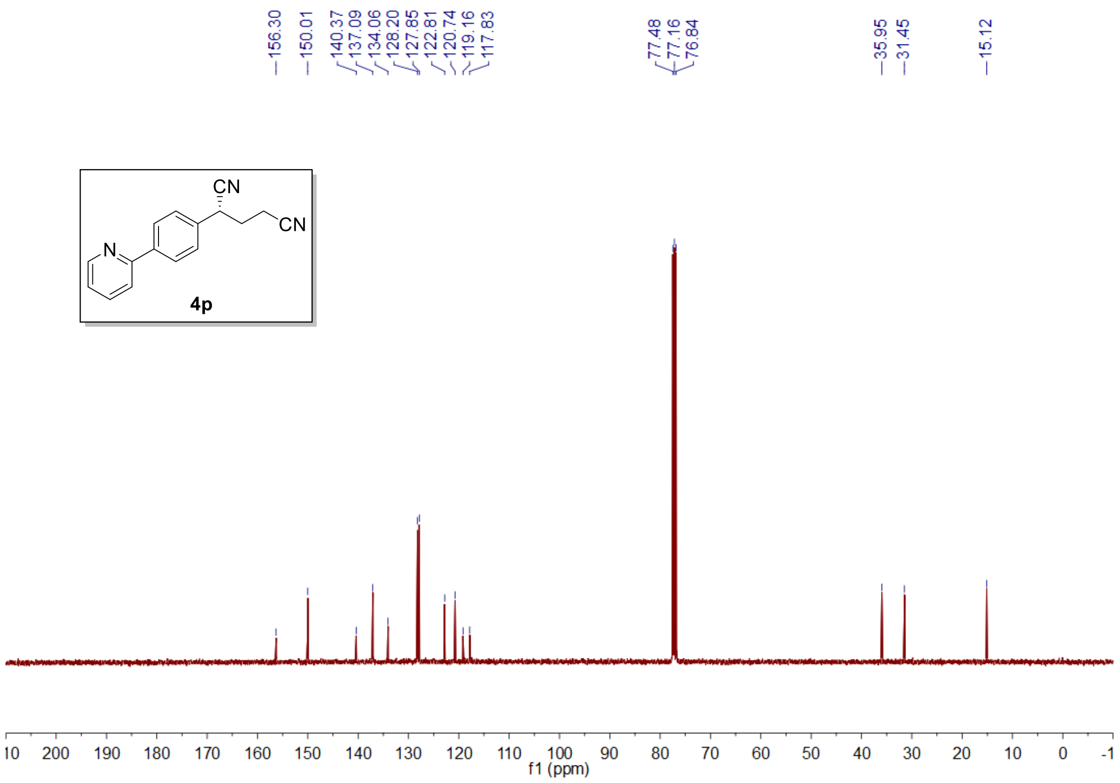

Supplementary Figure 136. ^{13}C NMR of **4p**

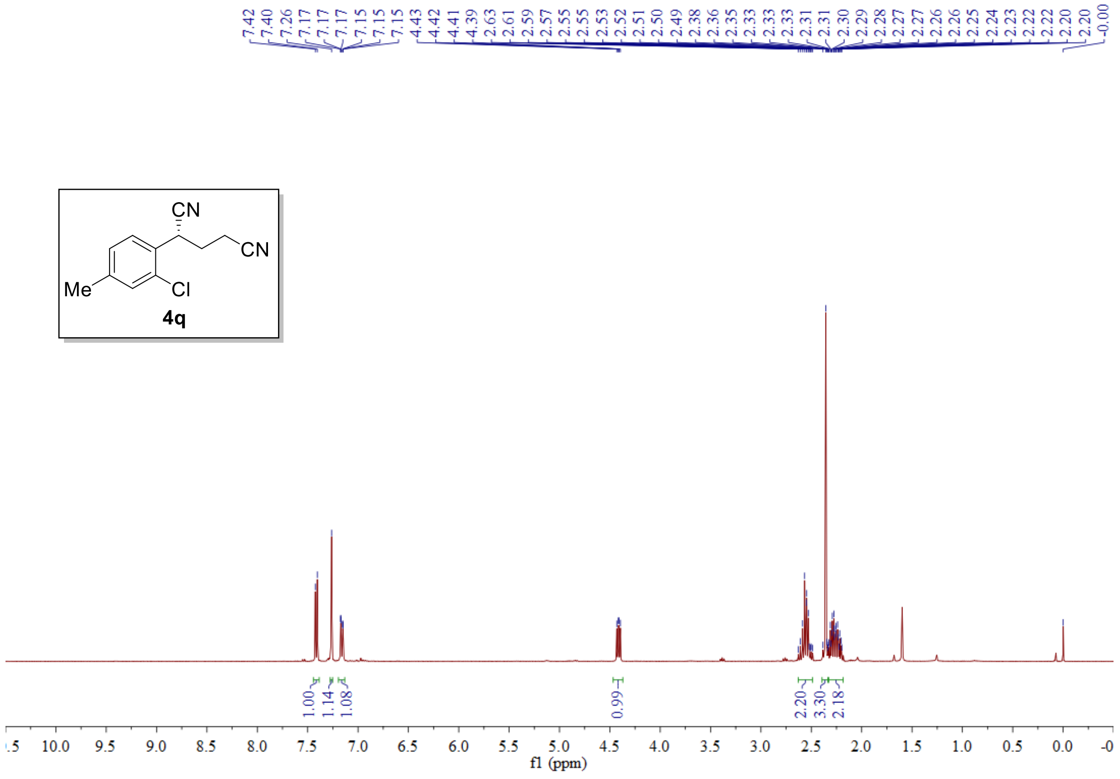

Supplementary Figure 137. ^{1}H NMR of **4q**

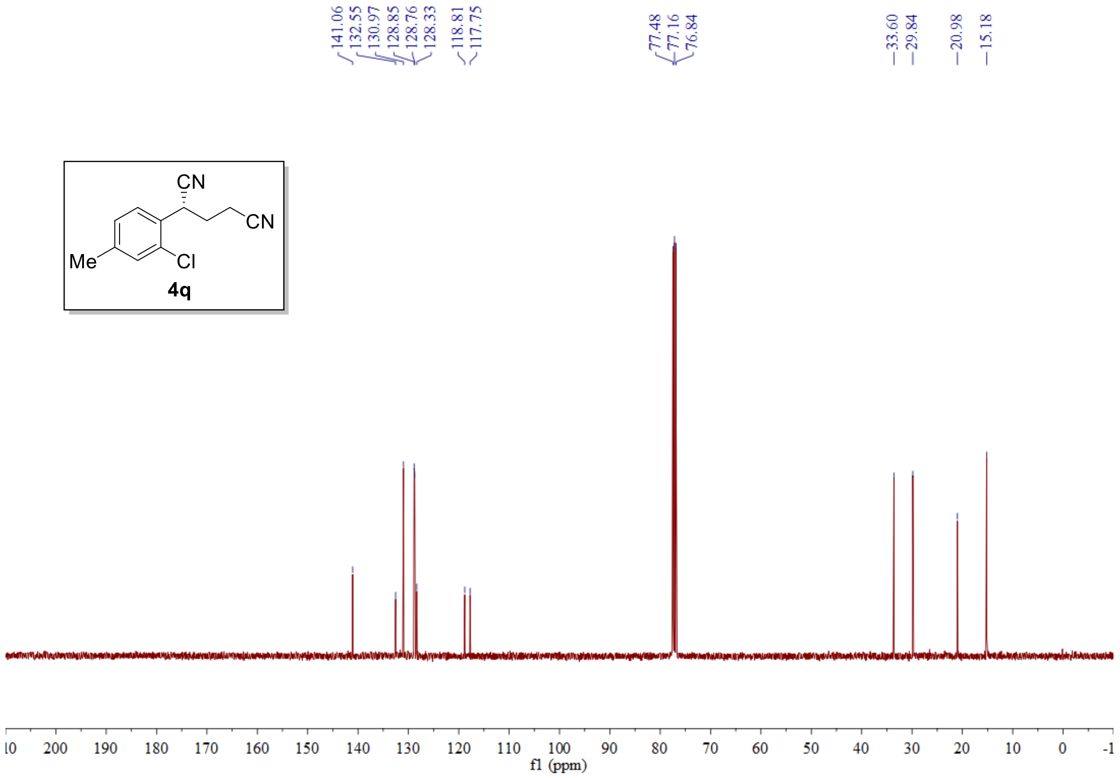

Supplementary Figure 138. ^{13}C NMR of **4q**

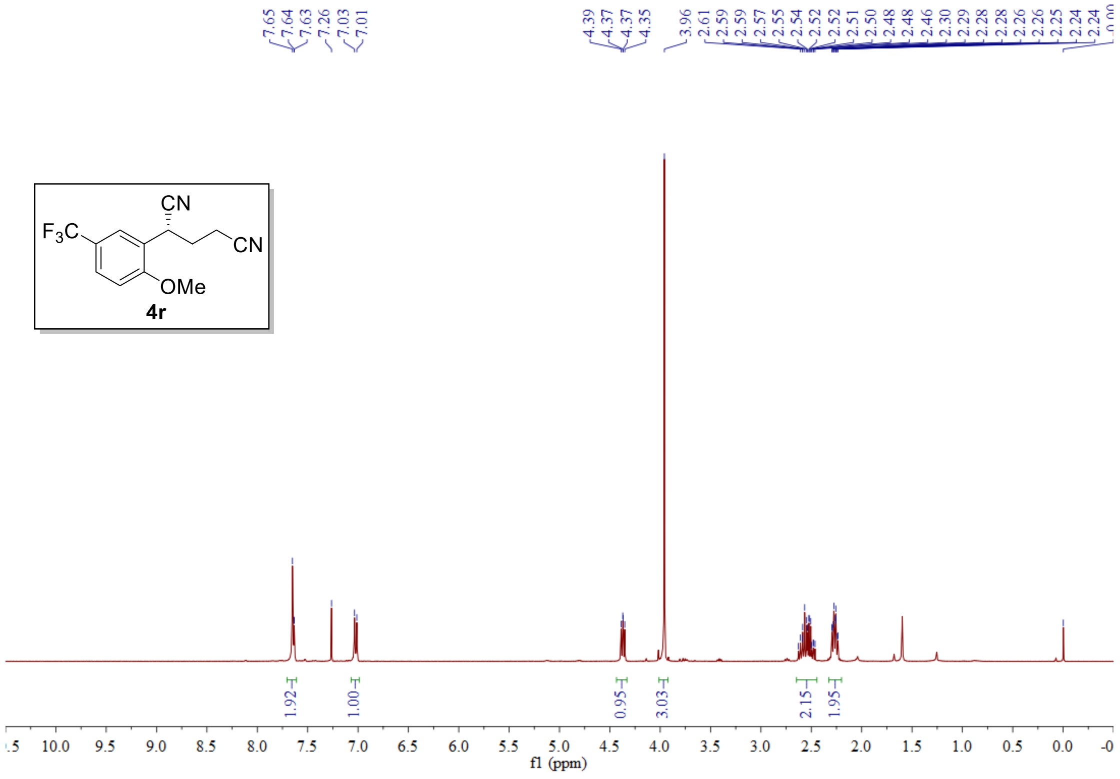

Supplementary Figure 139. ^{1}H NMR of **4r**

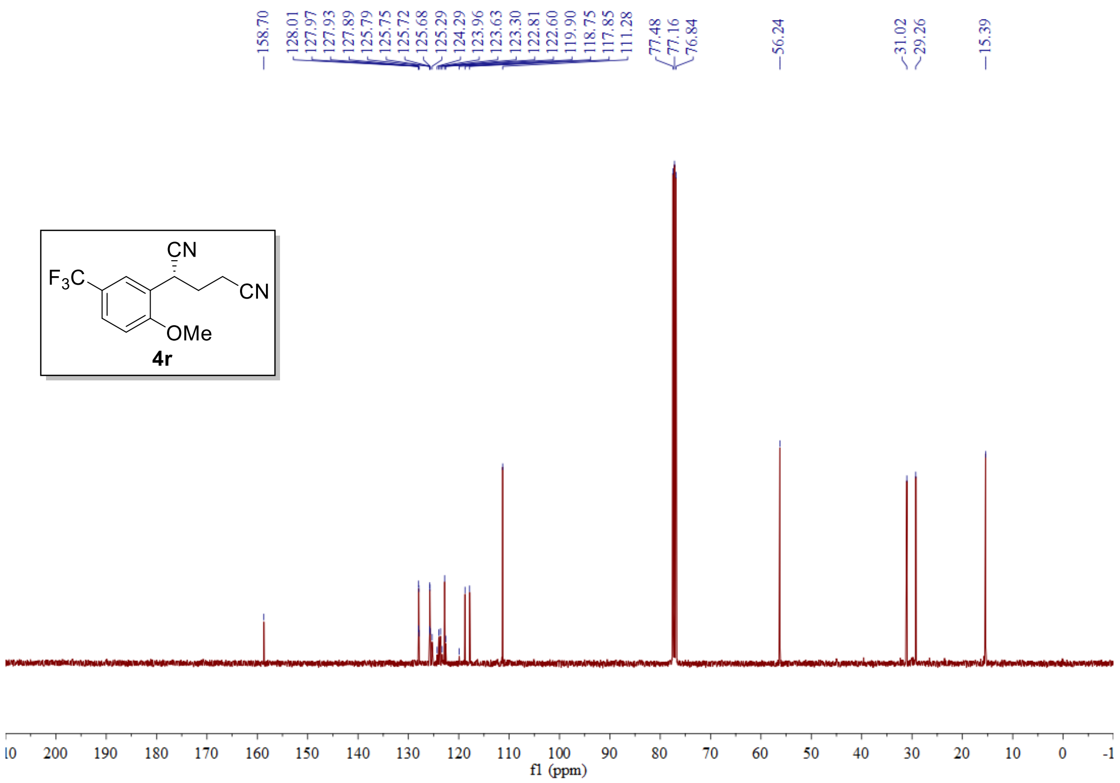

Supplementary Figure 140. ^{13}C NMR of **4r**

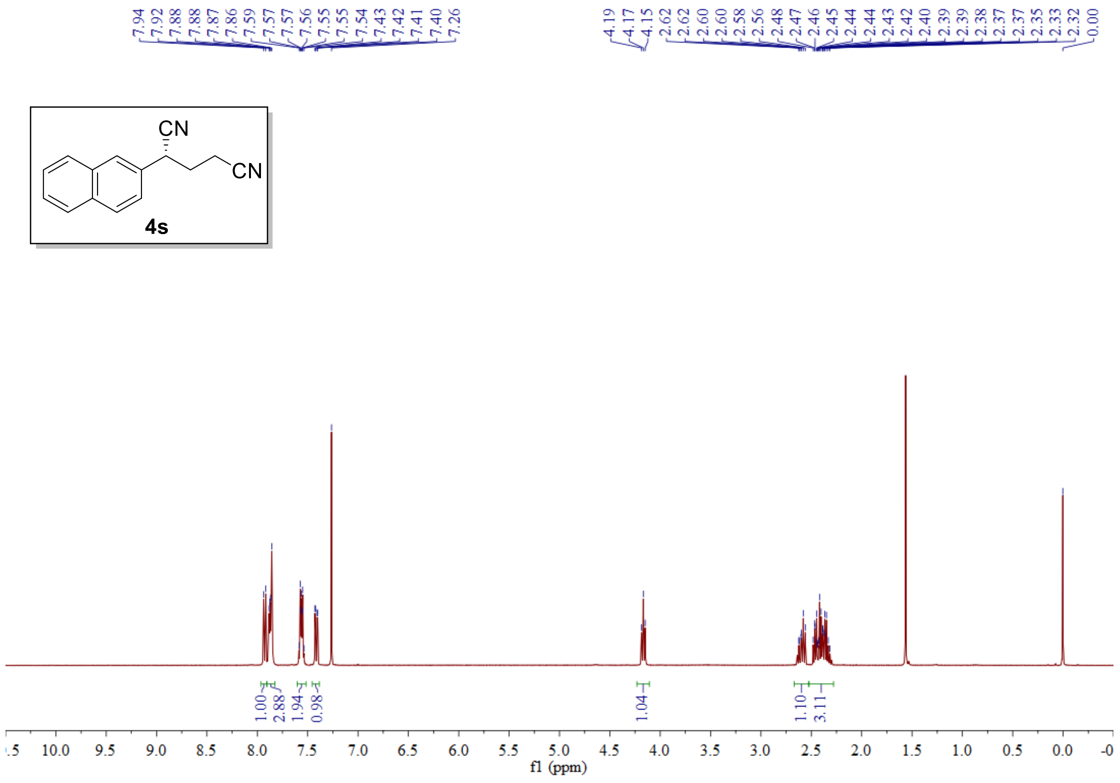

Supplementary Figure 141. ^{1}H NMR of **4s**

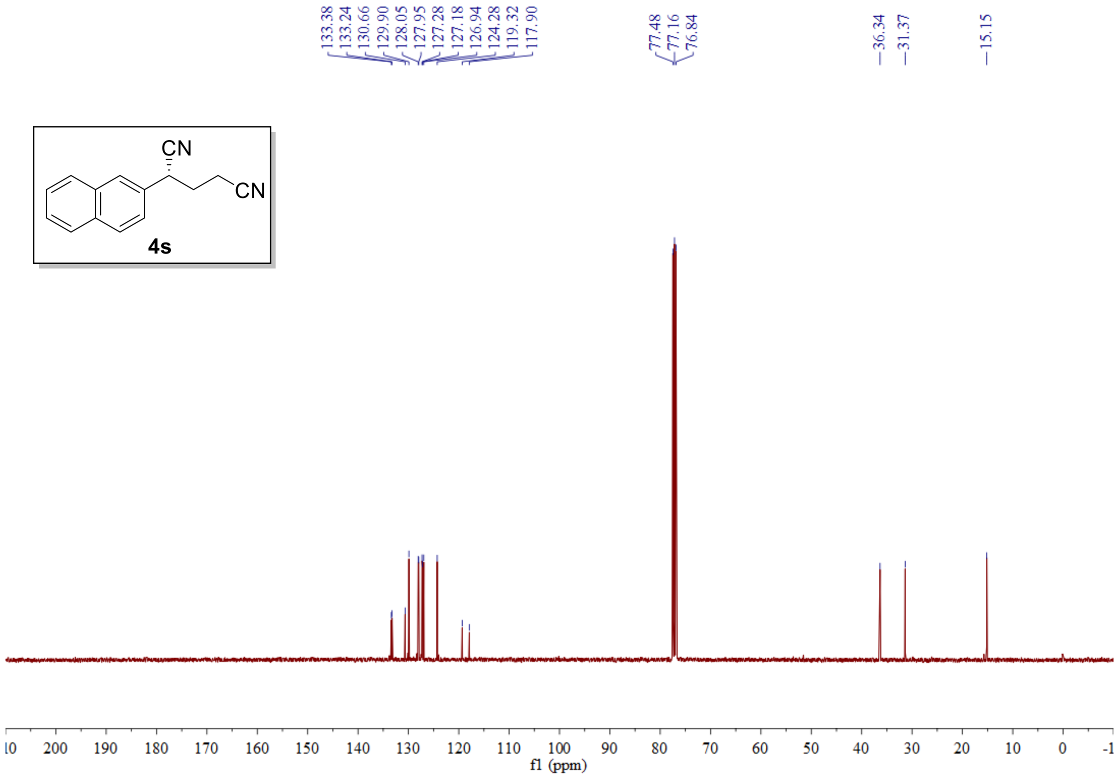

Supplementary Figure 142. ^{13}C NMR of **4s**

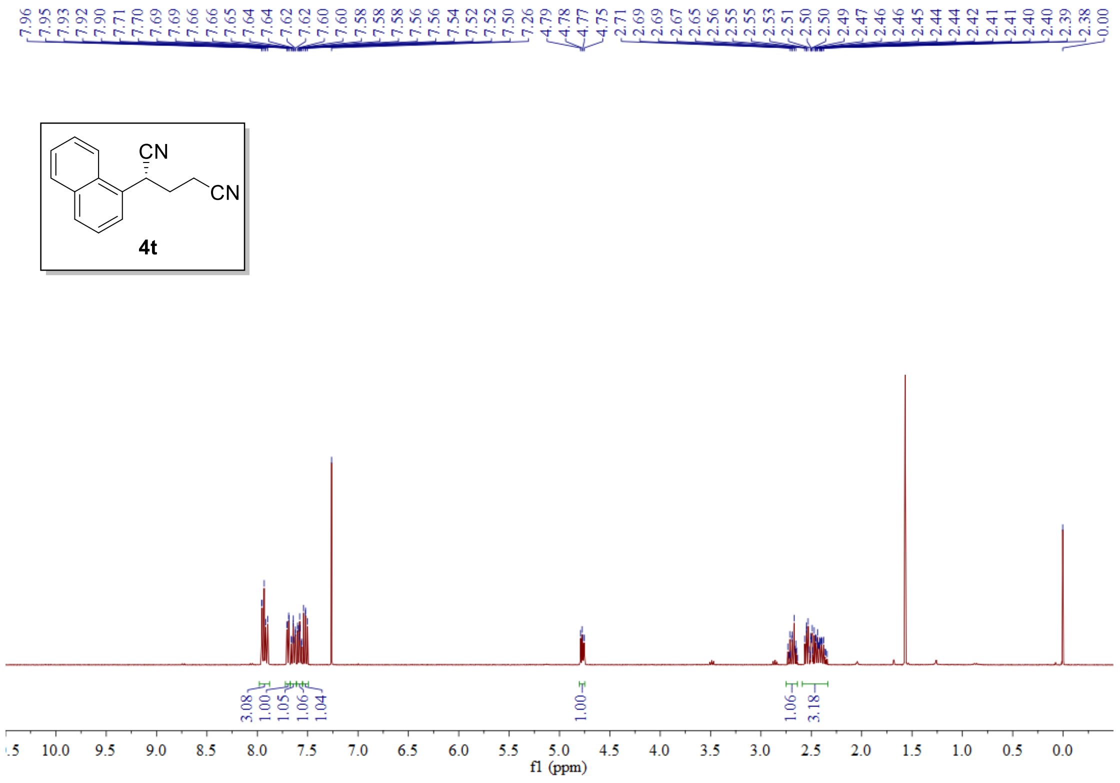

Supplementary Figure 143. ^{1}H NMR of **4t**

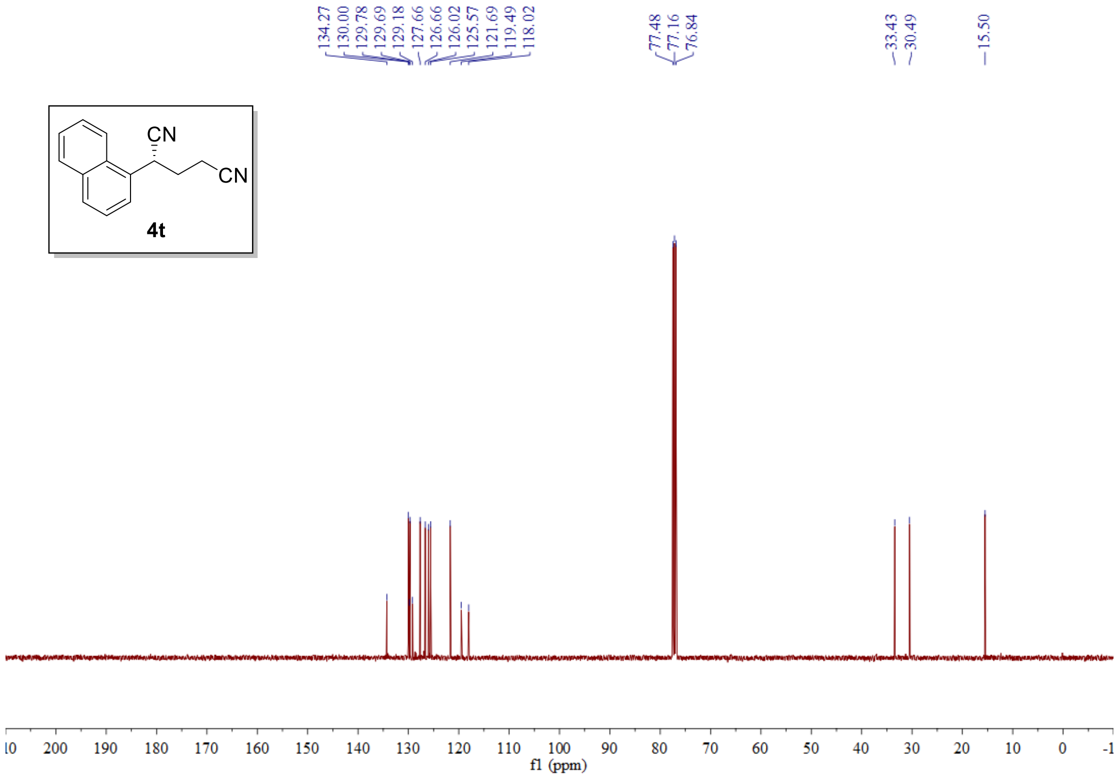

Supplementary Figure 144. ^{13}C NMR of **4t**

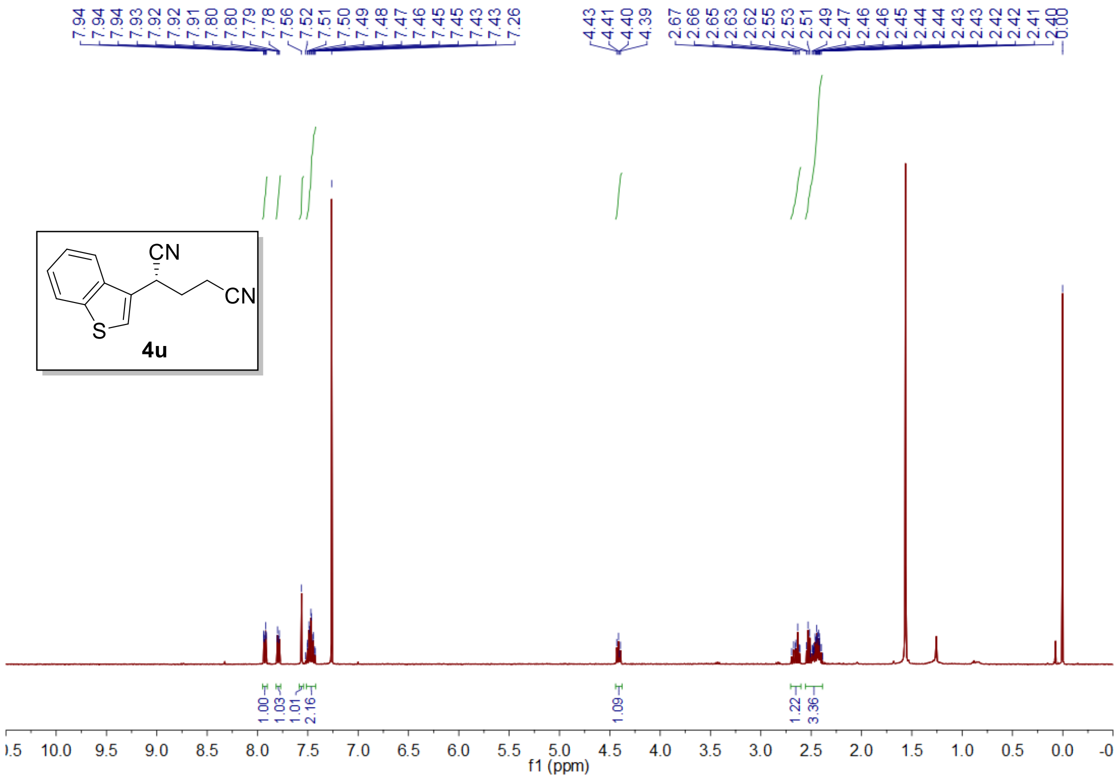

Supplementary Figure 145. ^{1}H NMR of **4u**

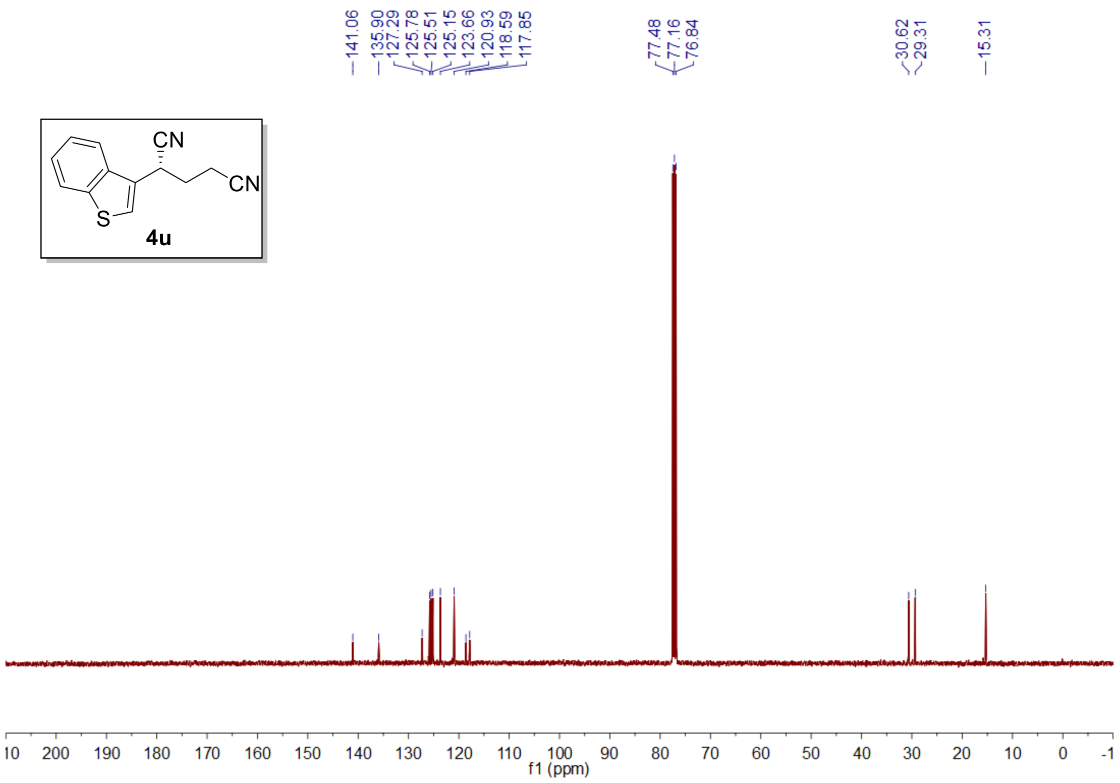

Supplementary Figure 146. ^{13}C NMR of **4u**

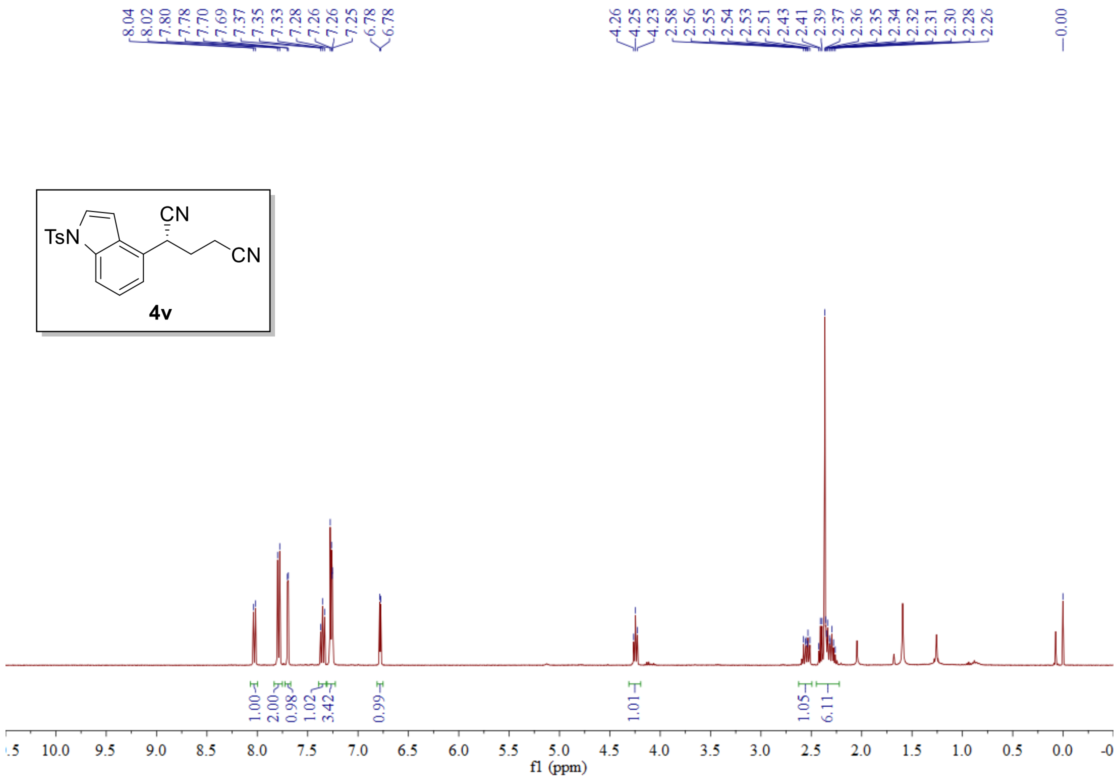

Supplementary Figure 147. ^{1}H NMR of **4v**

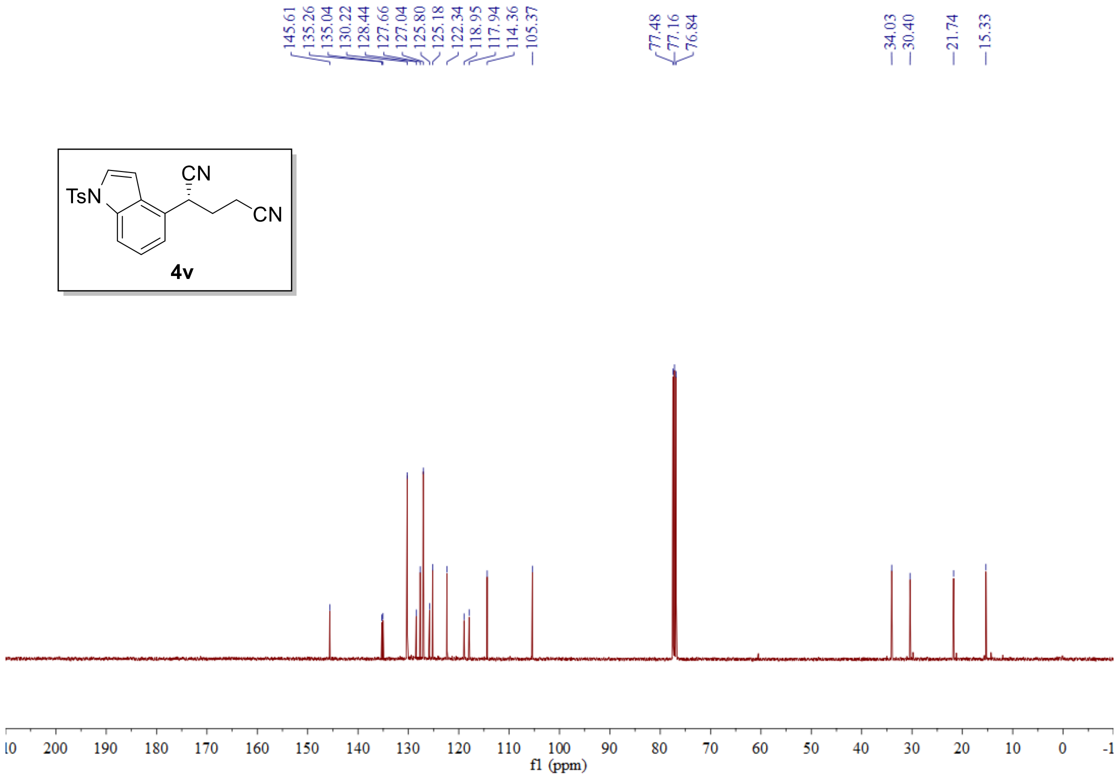

Supplementary Figure 148. ^{13}C NMR of **4v**

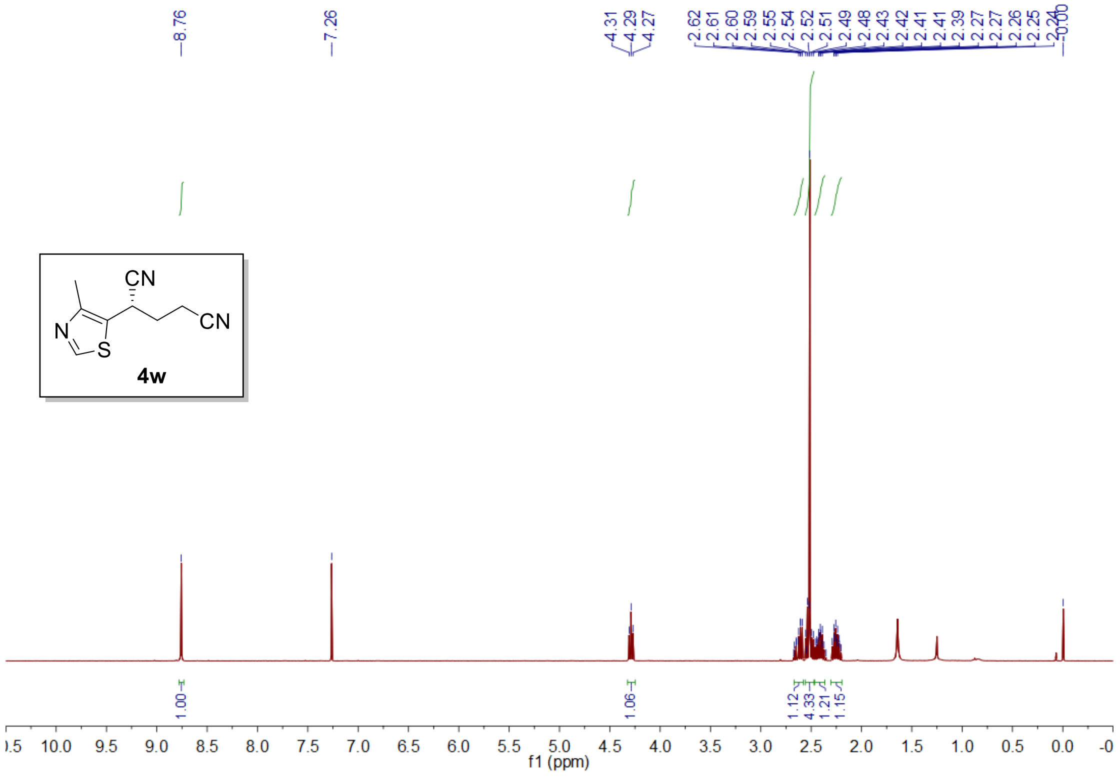

Supplementary Figure 149. ^{1}H NMR of **4w**

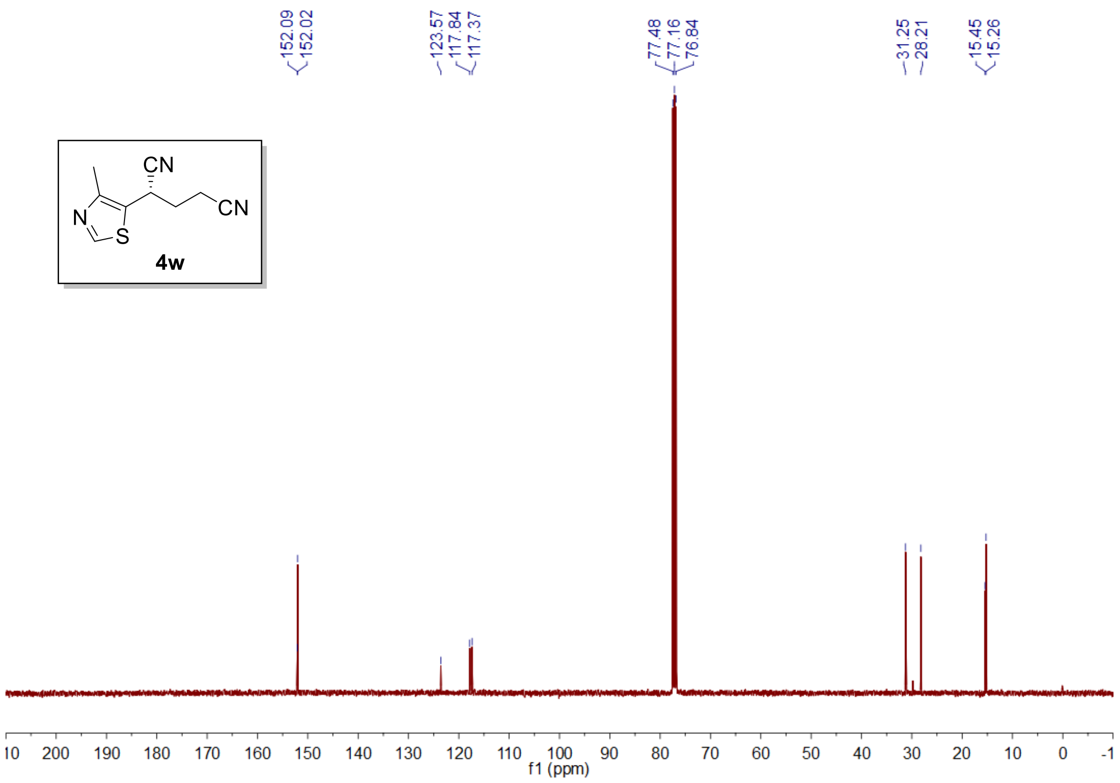

Supplementary Figure 150. ^{13}C NMR of **4w**

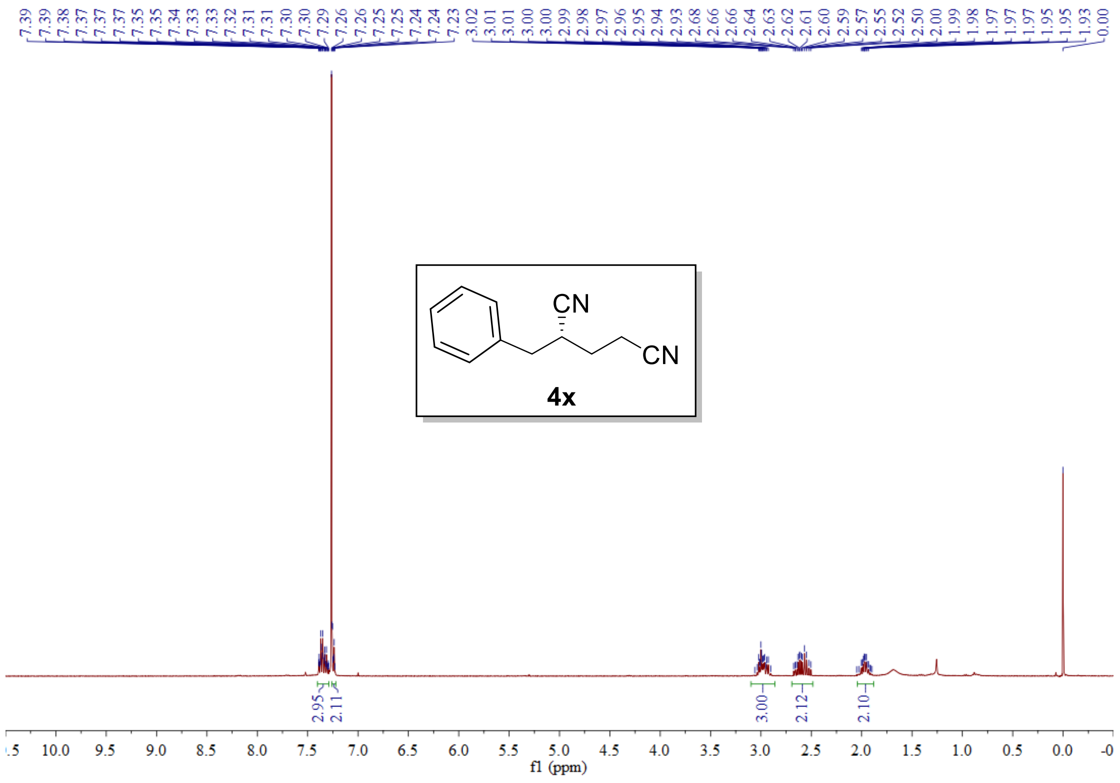

Supplementary Figure 151. ^{1}H NMR of **4x**

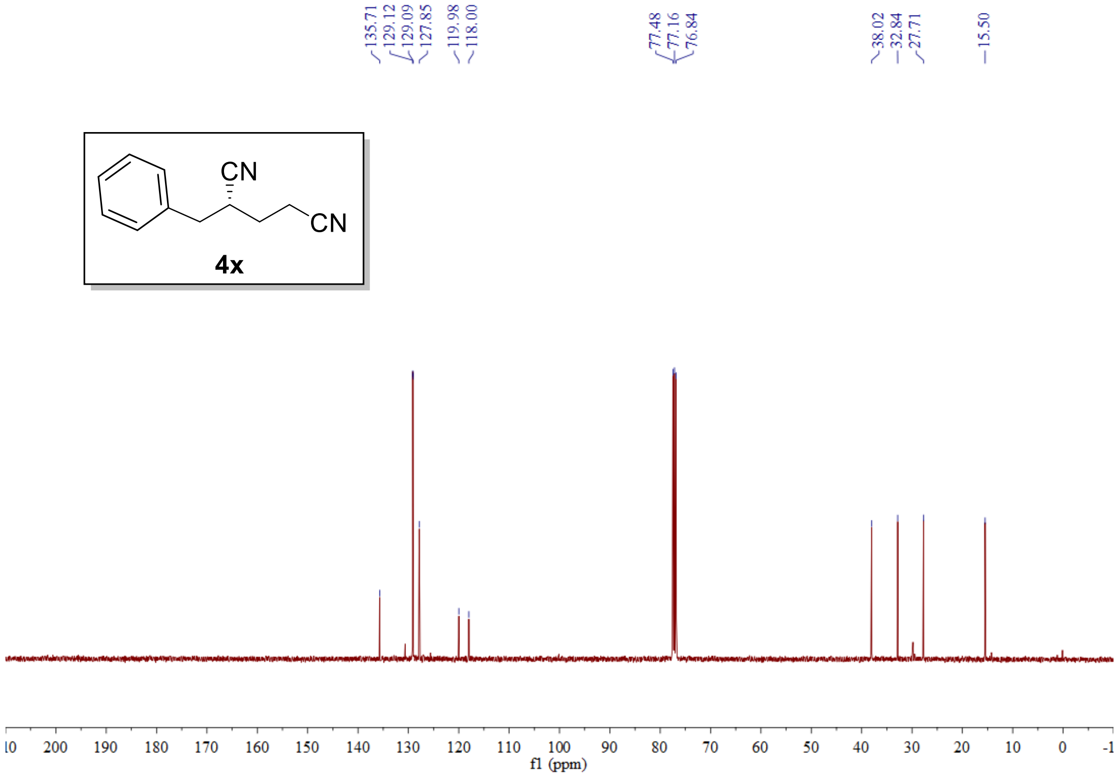

Supplementary Figure 152. ^{13}C NMR of **4x**

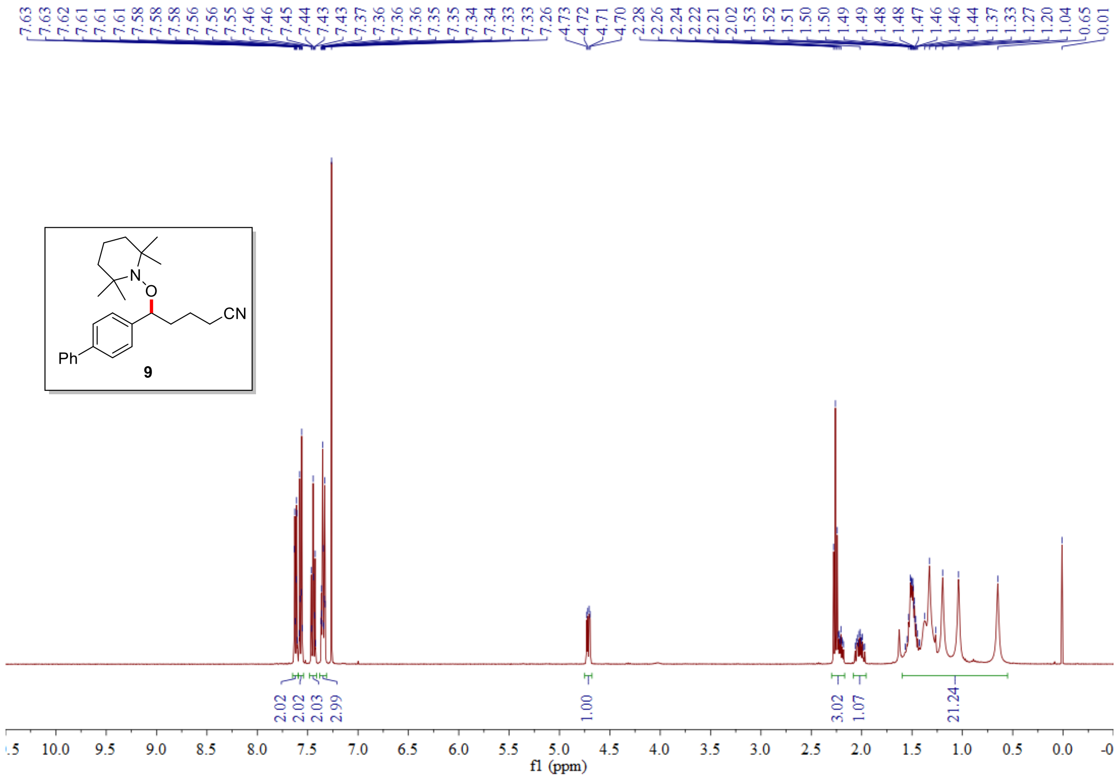

Supplementary Figure 153. ^{1}H NMR of **9**

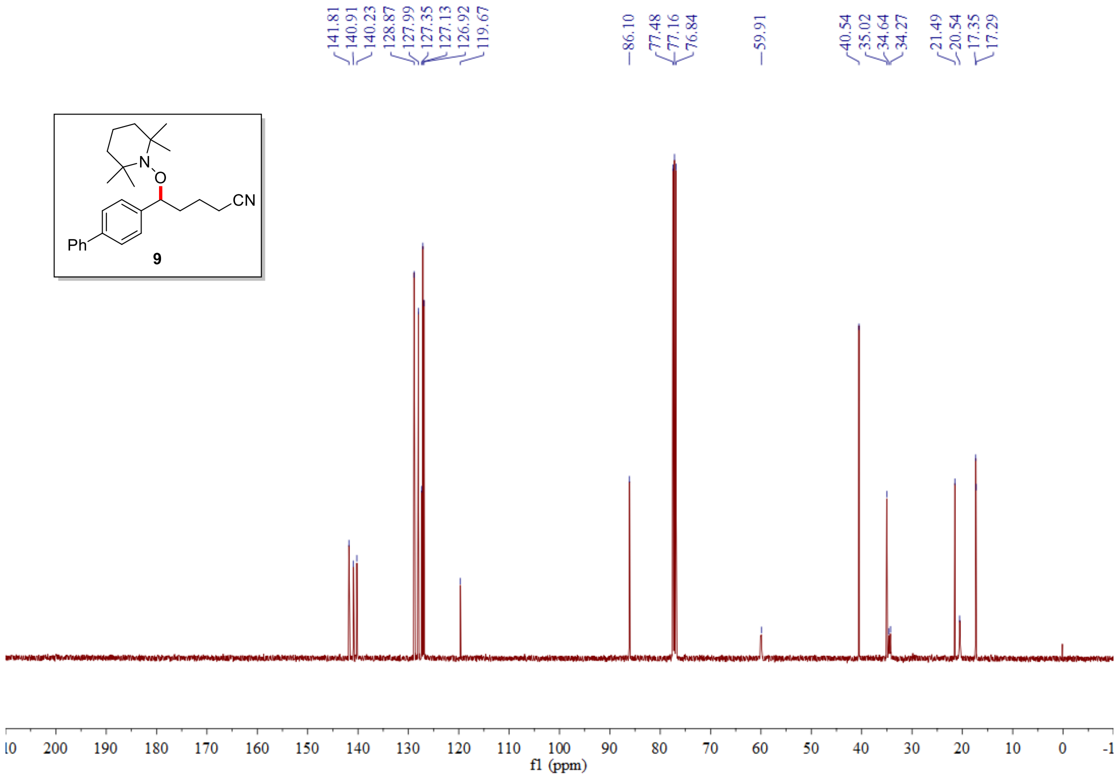

Supplementary Figure 154. ^{13}C NMR of **9**

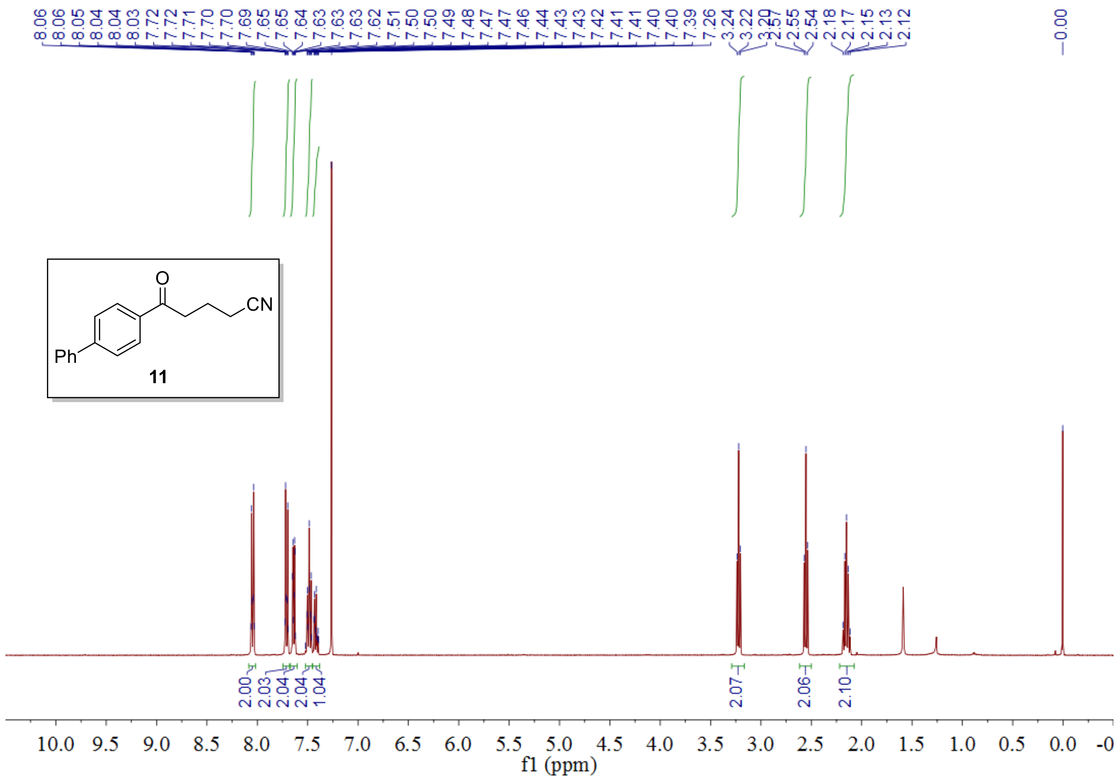

Supplementary Figure 155. ^{1}H NMR of **11**

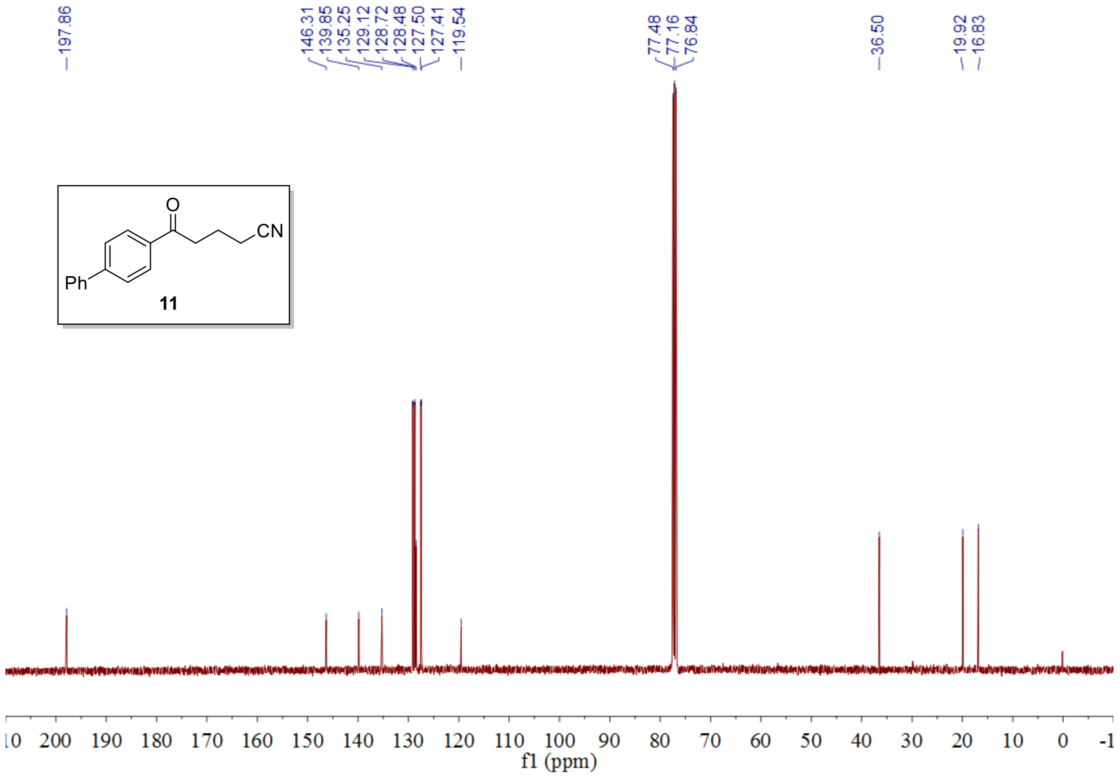

Supplementary Figure 156. ^{13}C NMR of **11**

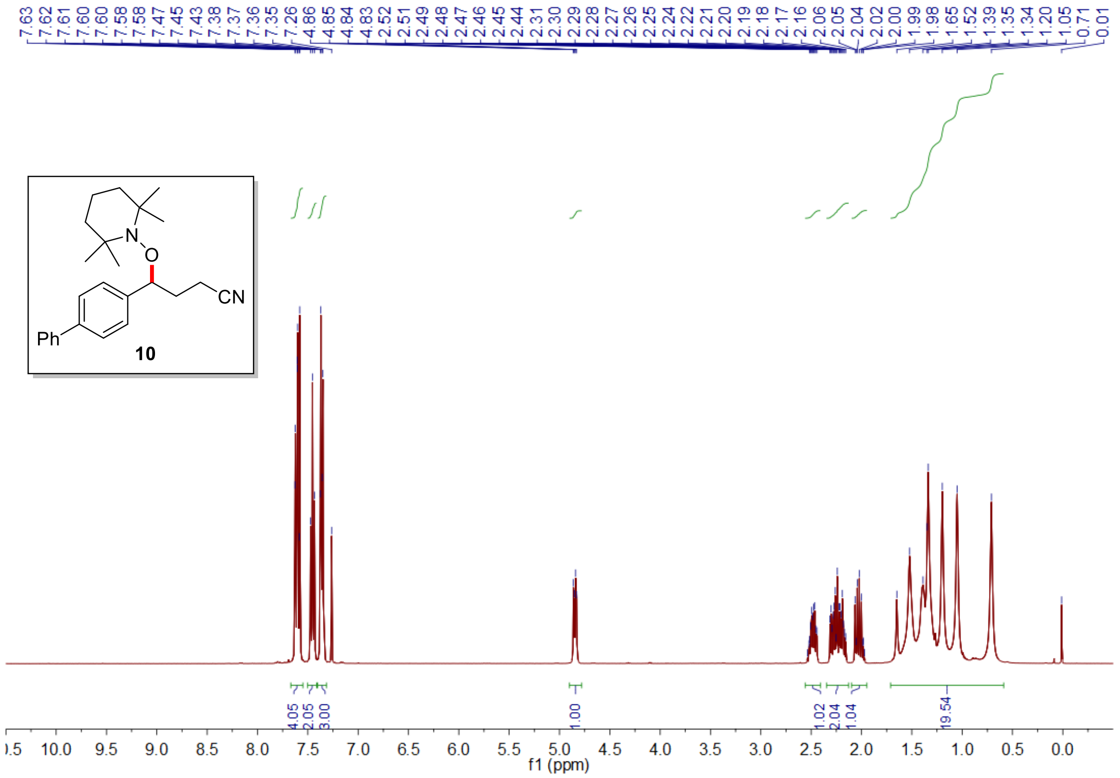

Supplementary Figure 157. ^{1}H NMR of **10**

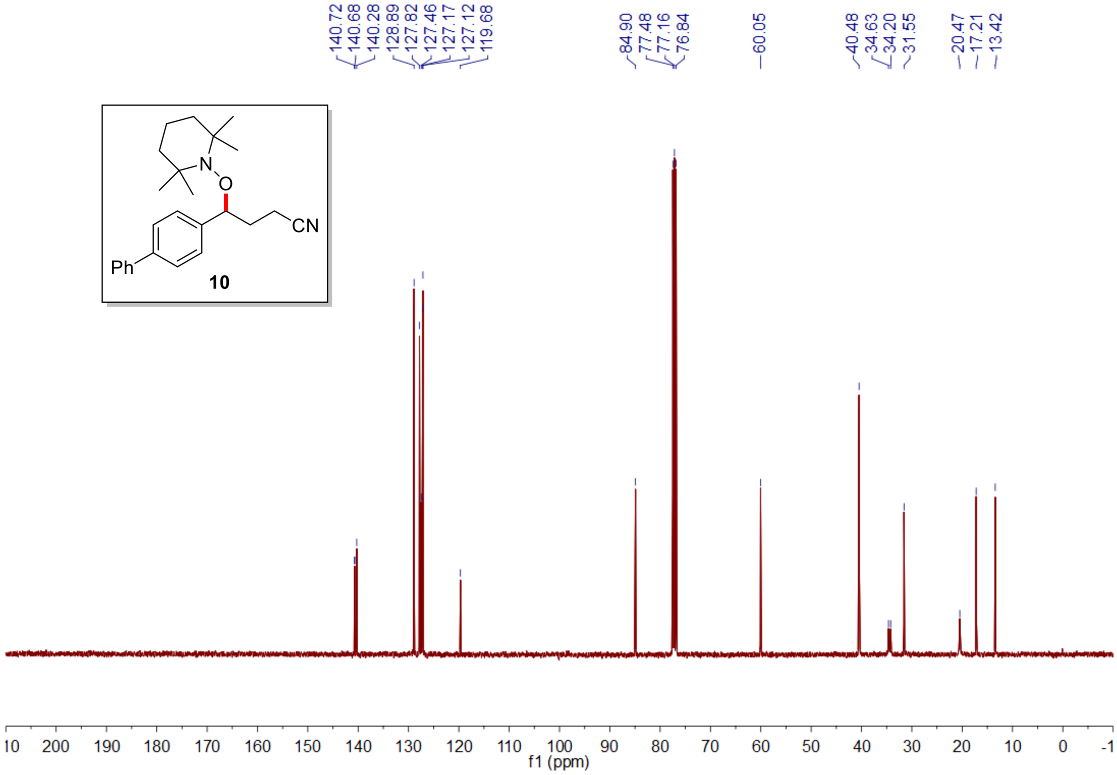

Supplementary Figure 158. ^{13}C NMR of **10**

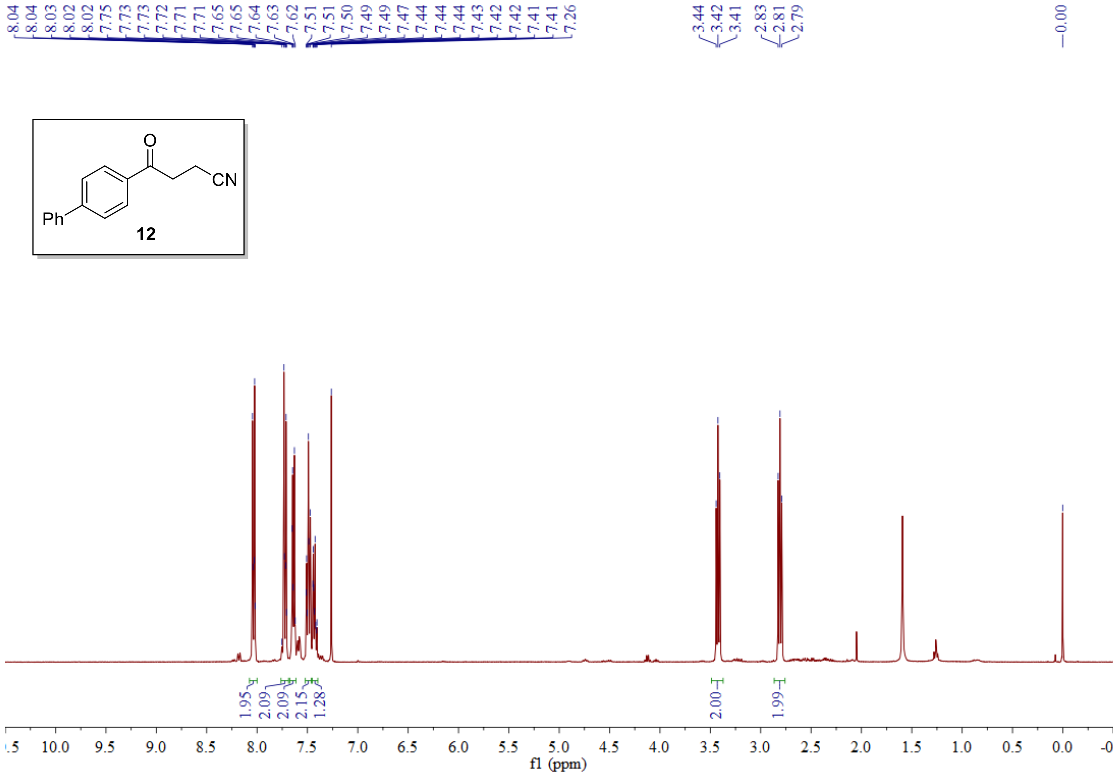

Supplementary Figure 159. ^{1}H NMR of **12**

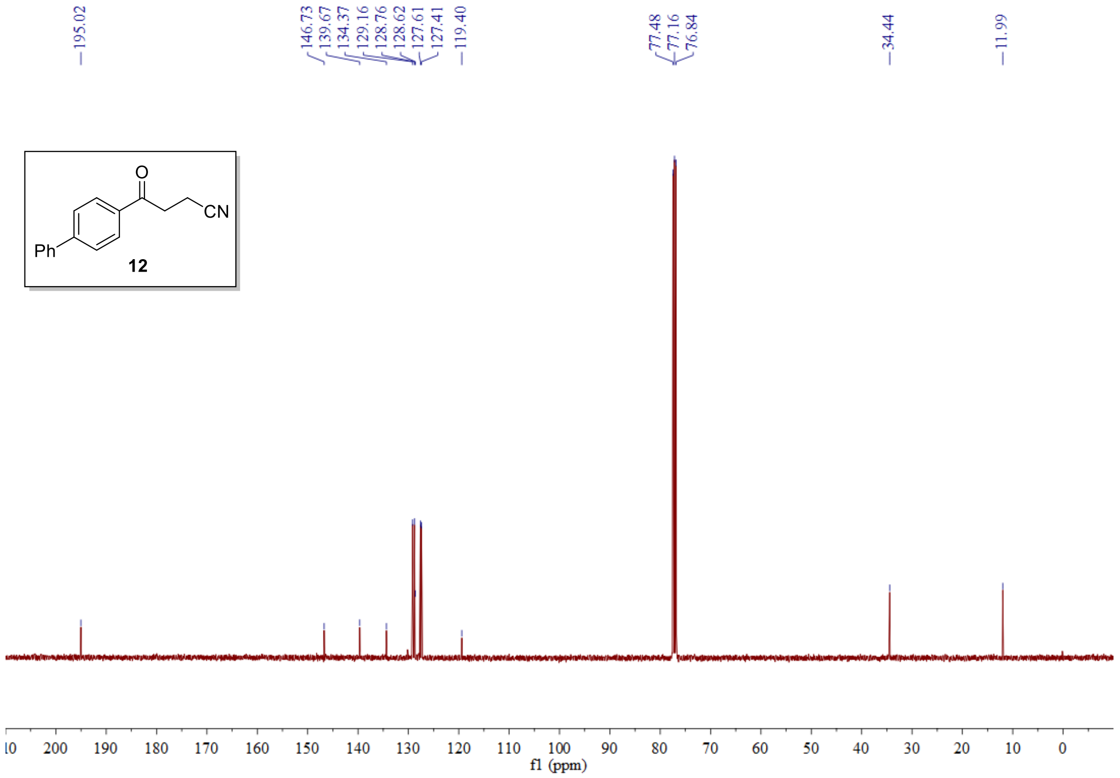

Supplementary Figure 160. ^{13}C NMR of **12**

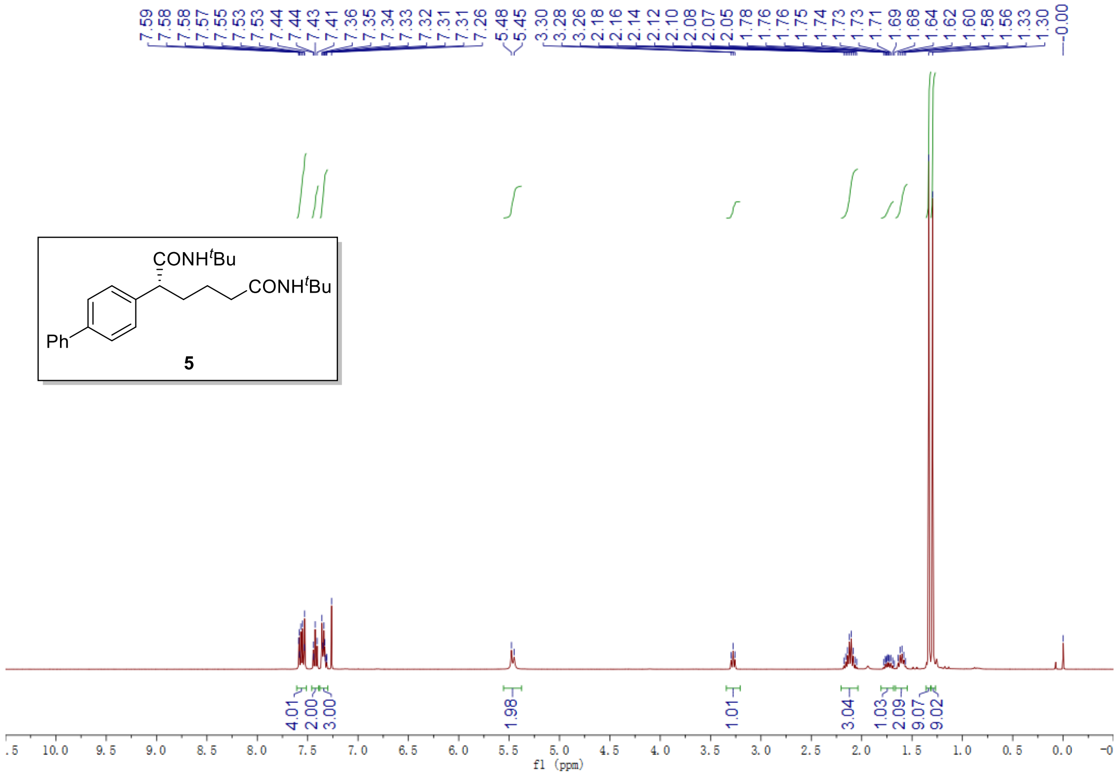

Supplementary Figure 161. ^{1}H NMR of **5**

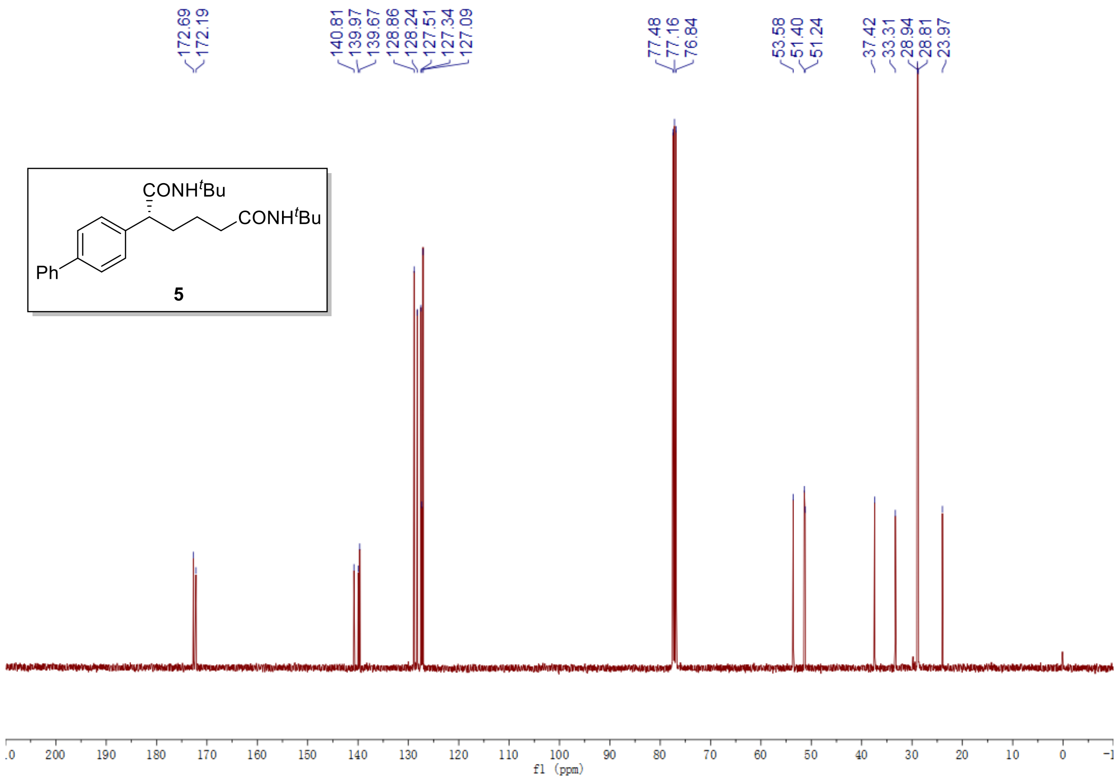

Supplementary Figure 162. ^{13}C NMR of **5**

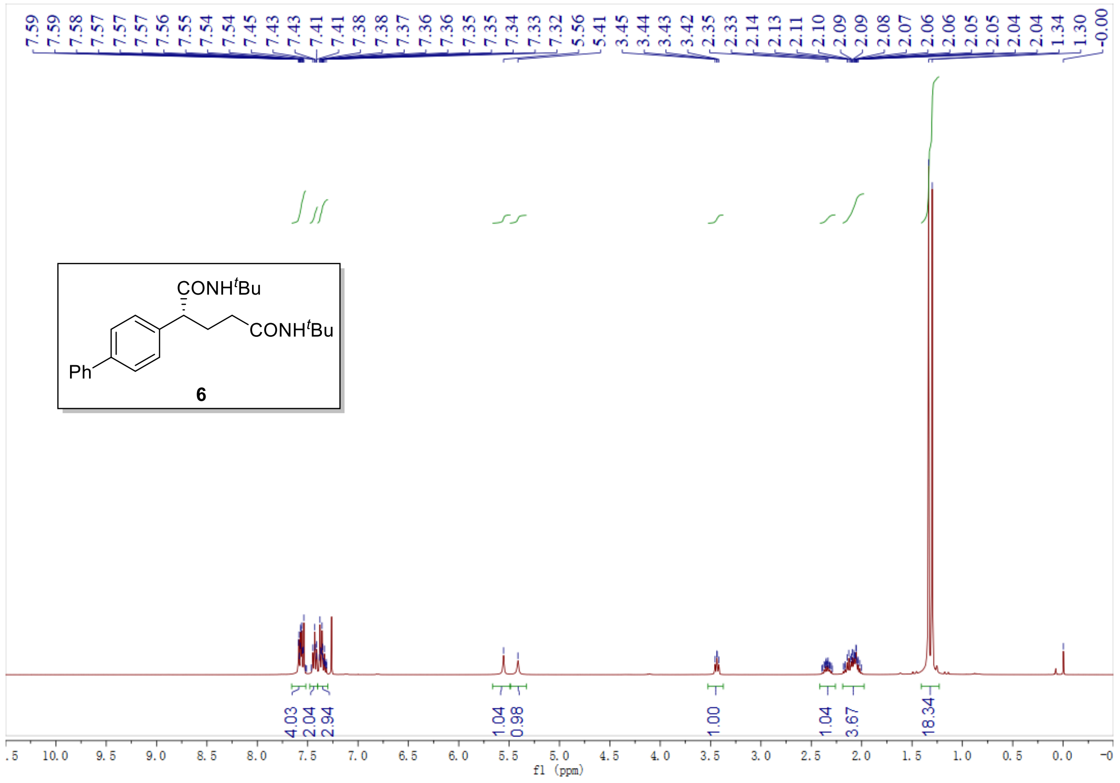

Supplementary Figure 163. ^{1}H NMR of **6**

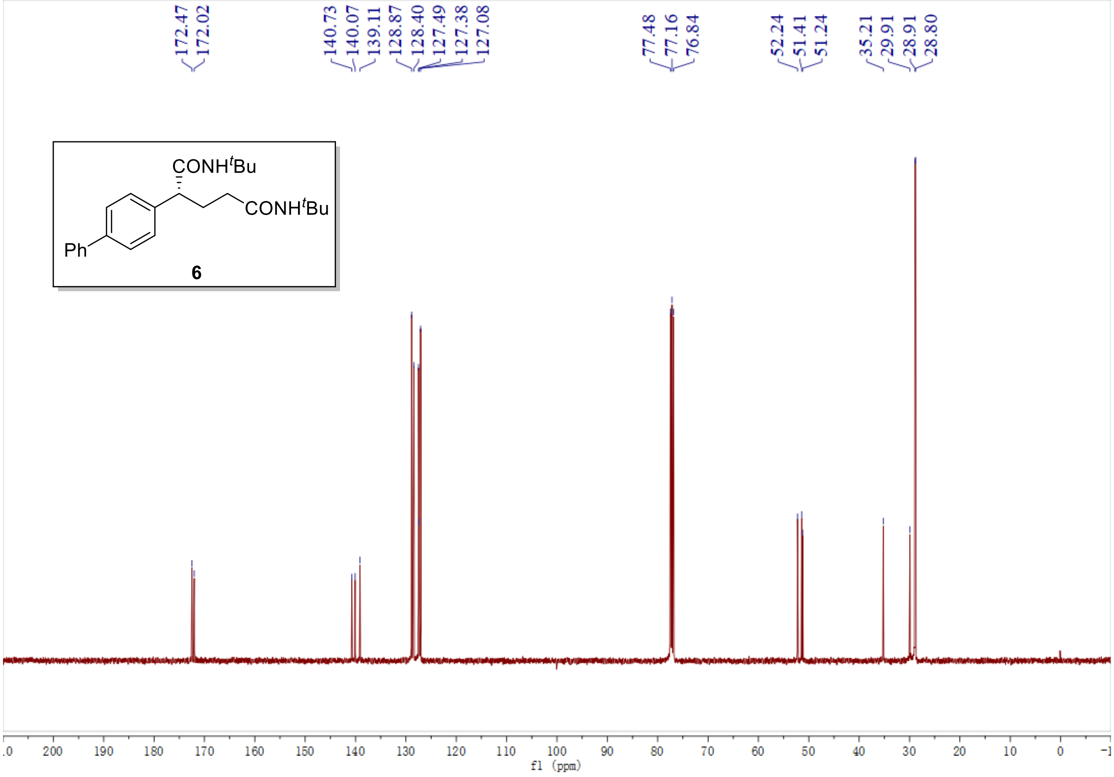

Supplementary Figure 164. ^{13}C NMR of **6**

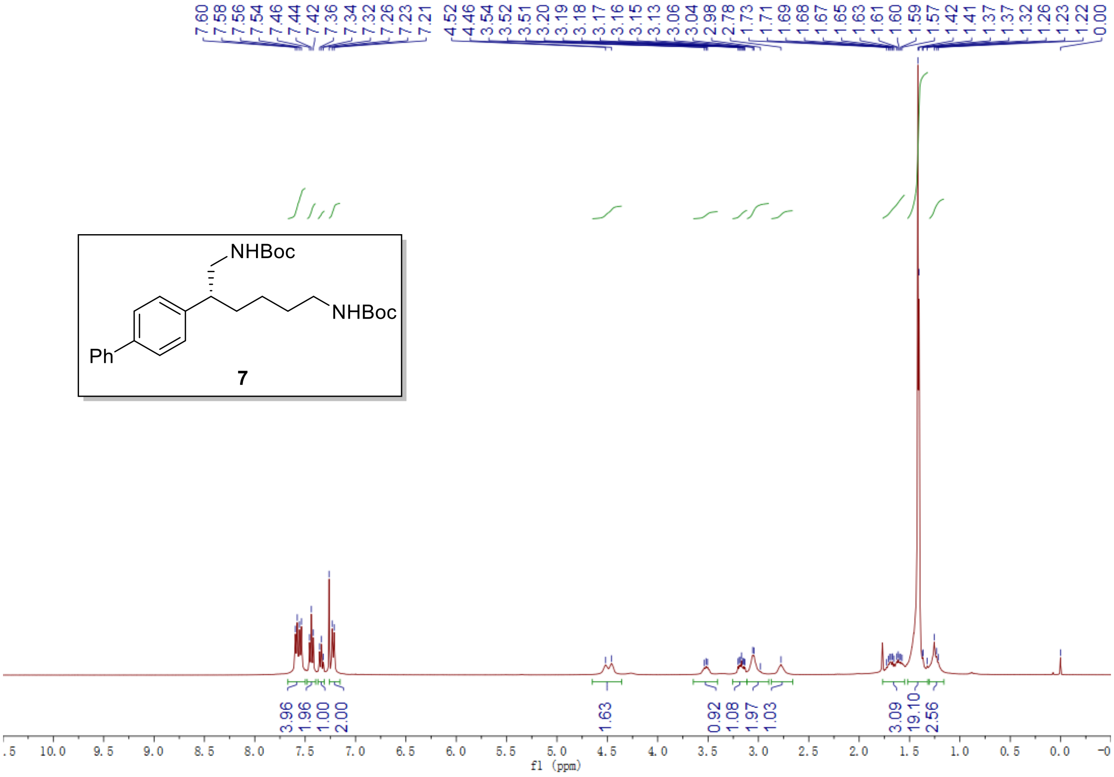

Supplementary Figure 165. ^{1}H NMR of **7**

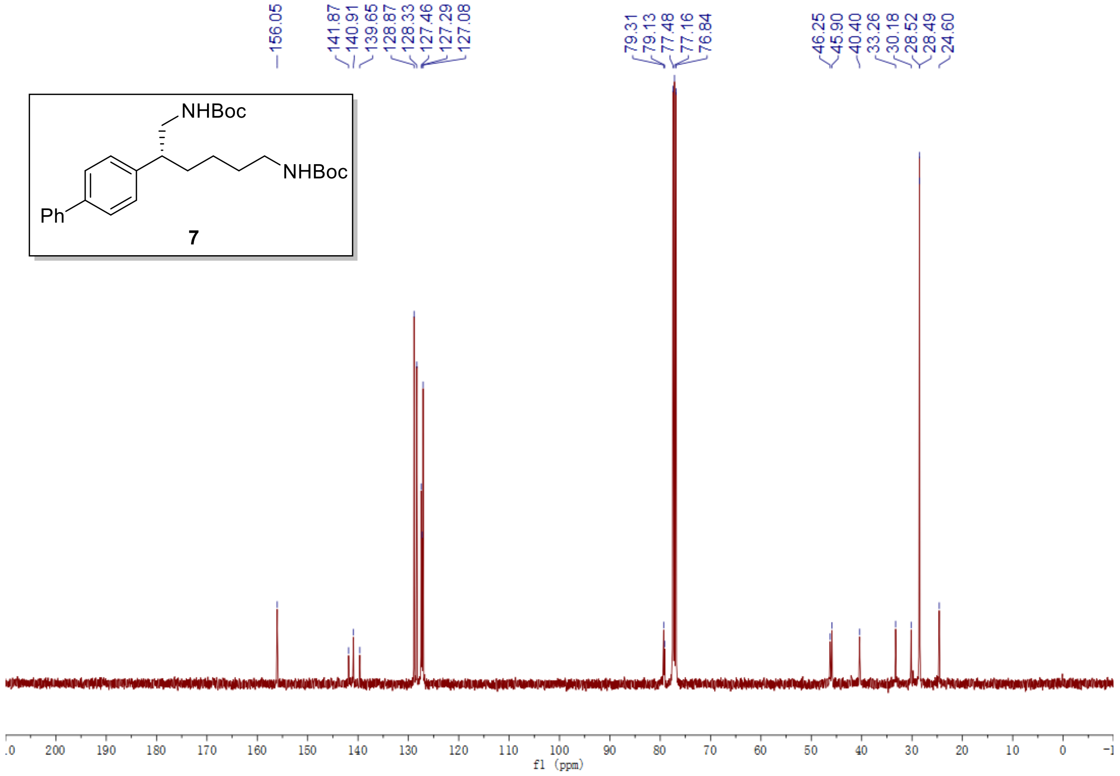

Supplementary Figure 166. ^{13}C NMR of **7**

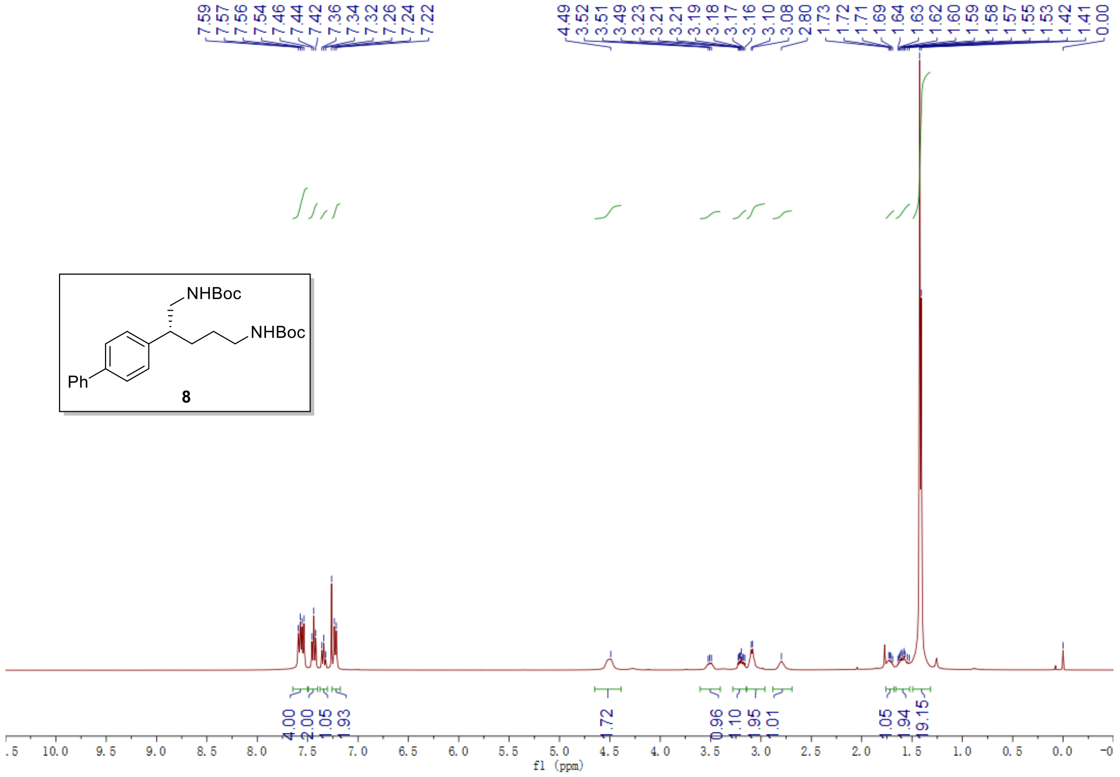

Supplementary Figure 167. ^{1}H NMR of **8**

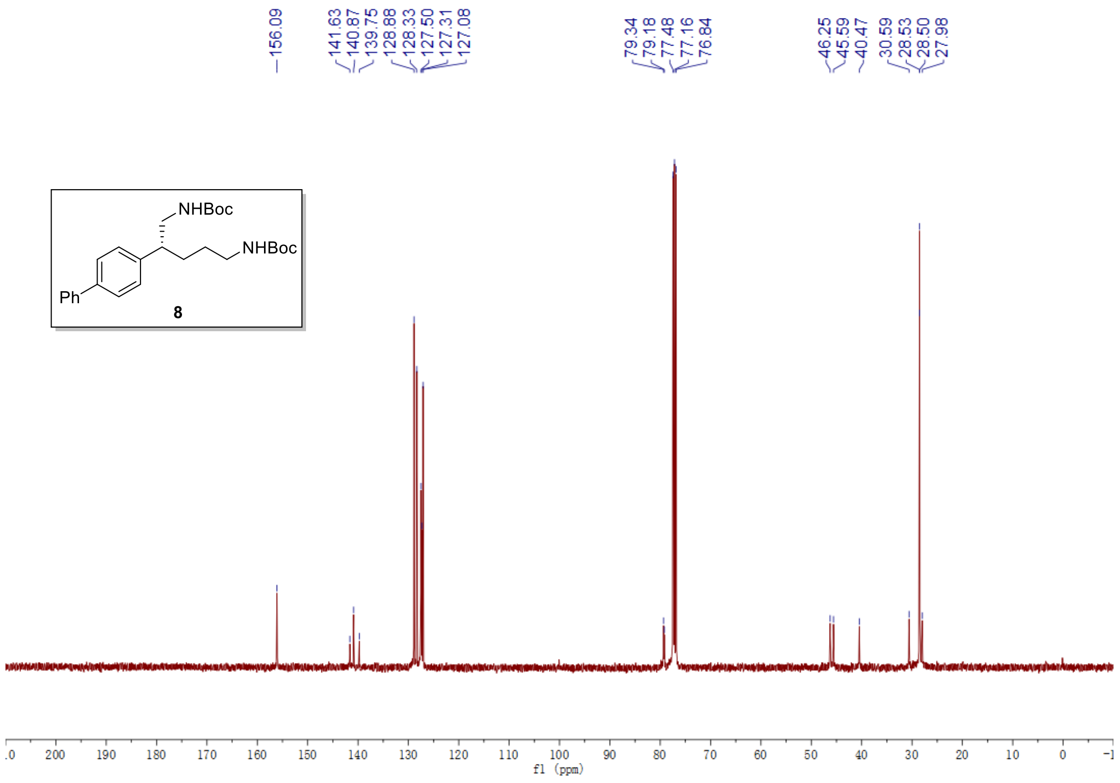

Supplementary Figure 168. ^{13}C NMR of **8**

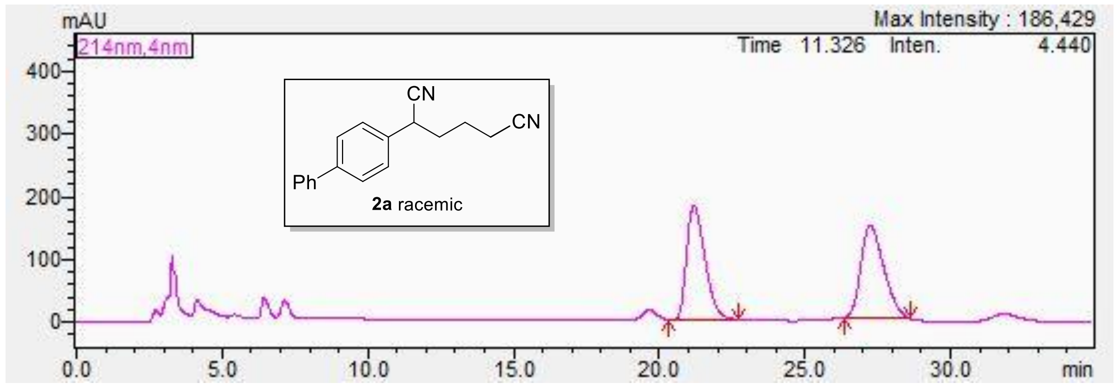

Peak	Ret. time	Area %	Height	Area
1	21.193	49.497	182329	7962872
2	27.239	50.503	147469	8124776
Totol:		100.000	329797	16087649

Supplementary Figure 169. HPLC data of rac-**2a**

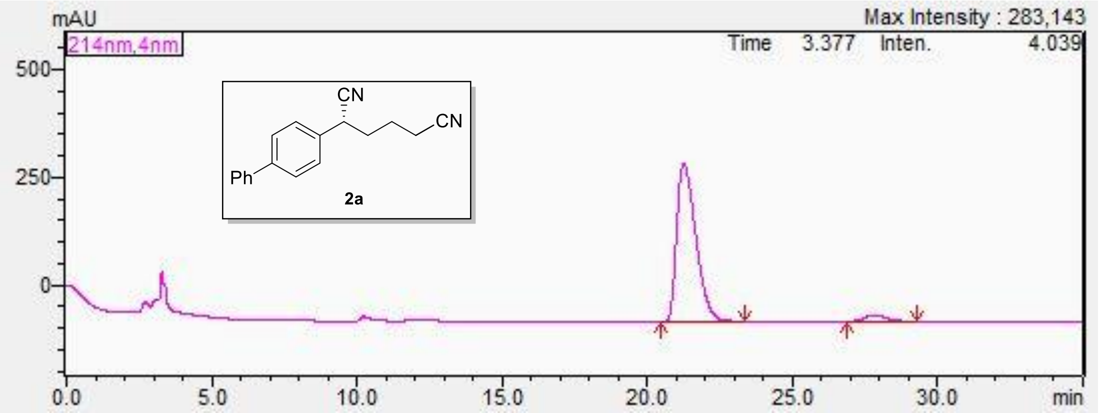

Peak	Ret. time	Area %	Height	Area
1	21.263	95.858	367380	16671598
2	27.826	4.142	13746	720306
Totol:		100.000	381126	17391904

Supplementary Figure 170. HPLC data of **2a**

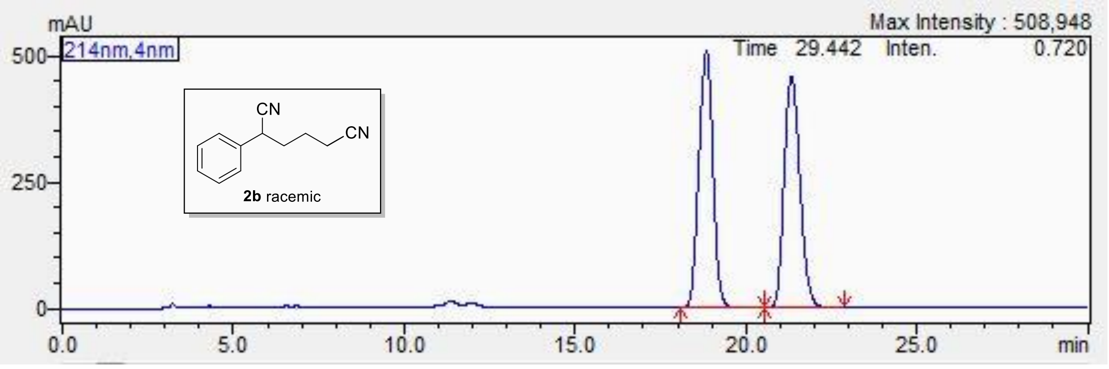

Peak	Ret. time	Area %	Height	Area
1	18.849	49.750	508132	14665027
2	21.339	50.250	458583	14812368
Totol:		100.000	966715	29477395

Supplementary Figure 171. HPLC data of rac-**2b**

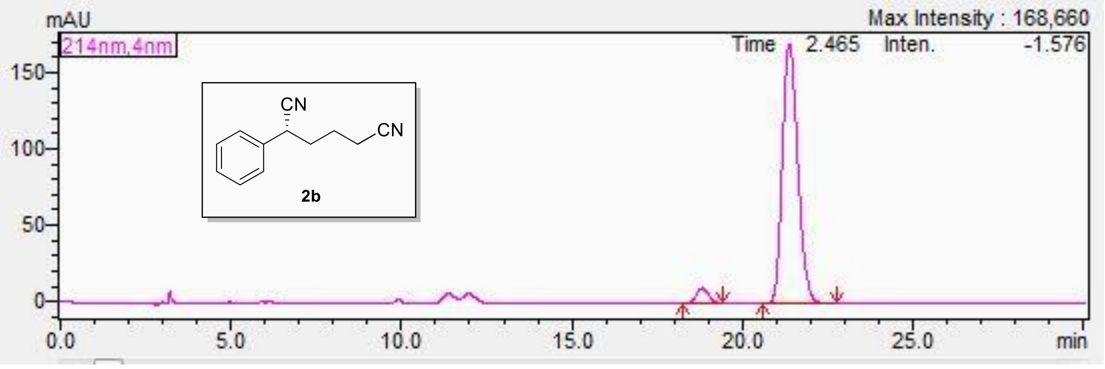

Peak	Ret. time	Area %	Height	Area
1	18.828	4.712	10158	264766

2	21.376	95.288	170088	5354716
Totol:		100.000	180246	5619482

Supplementary Figure 172. HPLC data of **2b**

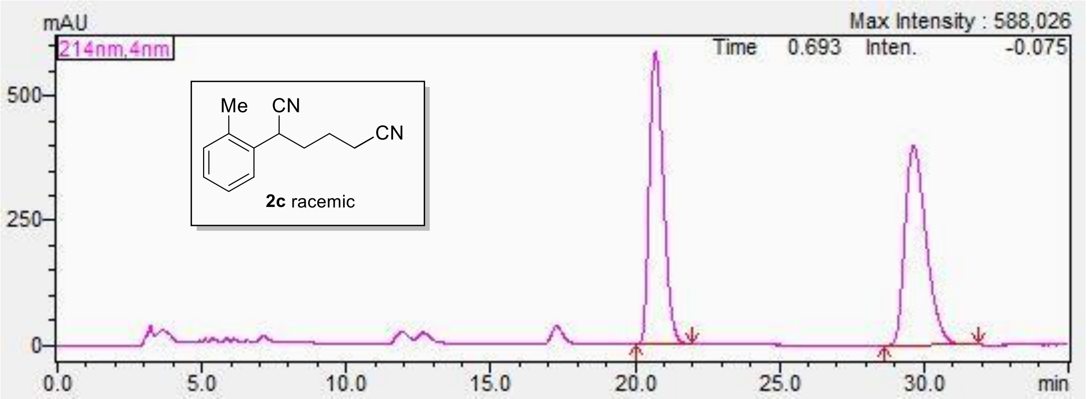

Peak	Ret. time	Area %	Height	Area
1	20.706	49.081	586181	19740769
2	29.646	50.919	399264	20480290
Totol:		100.000	985445	40221059

Supplementary Figure 173. HPLC data of rac-**2c**

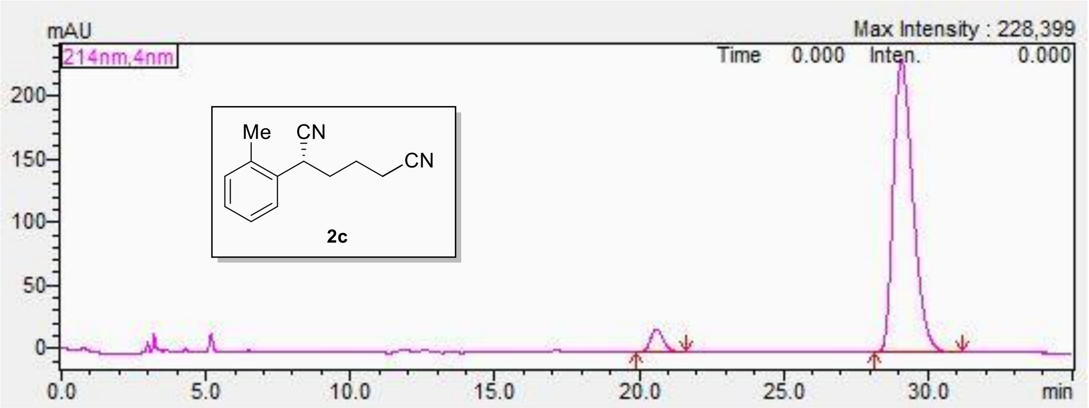

Peak	Ret. time	Area %	Height	Area
1	20.601	4.980	18454	573471
2	29.091	95.020	231685	10941981
Totol:		100.000	250140	11515452

Supplementary Figure 174. HPLC data of **2c**

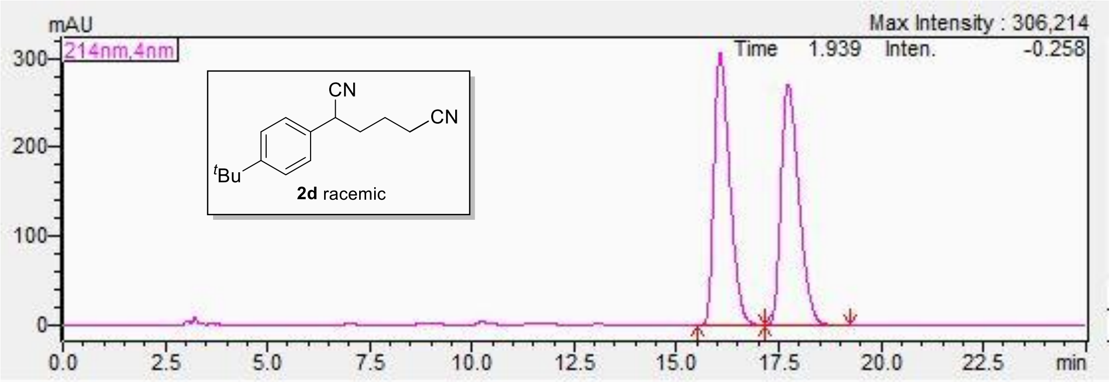

Peak	Ret. time	Area %	Height	Area
1	16.068	49.889	306448	8207774
2	17.730	50.111	271171	8244137
Totol:		100.000	577619	16451911

Supplementary Figure 175. HPLC data of rac-**2d**

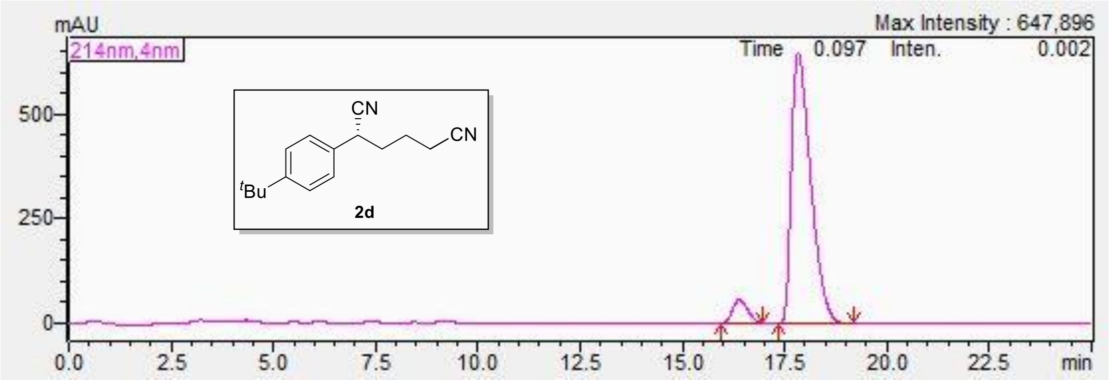

Peak	Ret. time	Area %	Height	Area
1	16.393	6.233	55535	1407118
2	17.831	93.767	647704	21169690
Totol:		100.000	703240	22576809

Supplementary Figure 176. HPLC data of **2d**

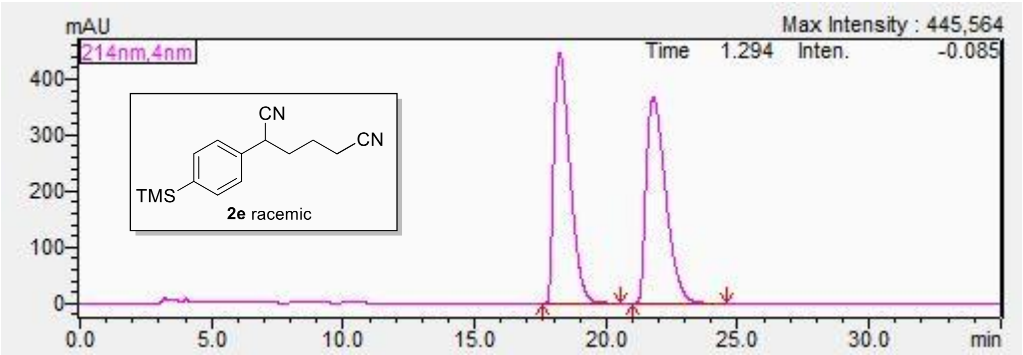

Peak	Ret. time	Area %	Height	Area

1	18.237	49.938	445578	18926413
2	21.794	50.062	369114	18973695
Totol:		100.000	814692	37900108

Supplementary Figure 177. HPLC data of rac-**2e**

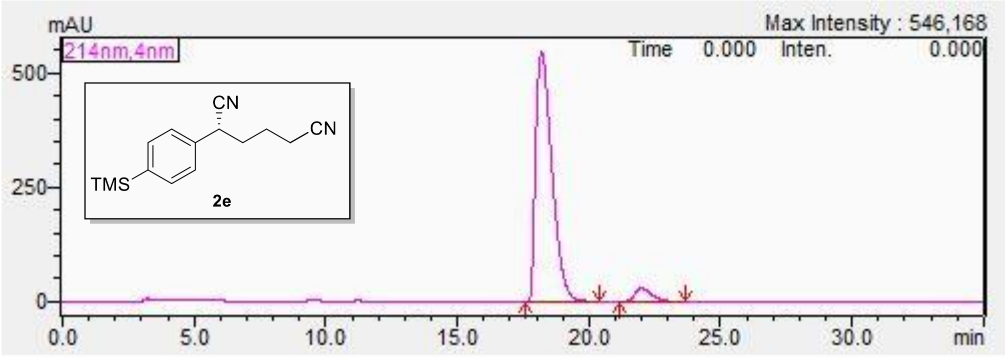

Peak	Ret. time	Area %	Height	Area
1	18.198	94.977	546278	23390003
2	22.009	5.023	28322	1236980
Totol:		100.000	574599	24626983

Supplementary Figure 178. HPLC data of **2e**

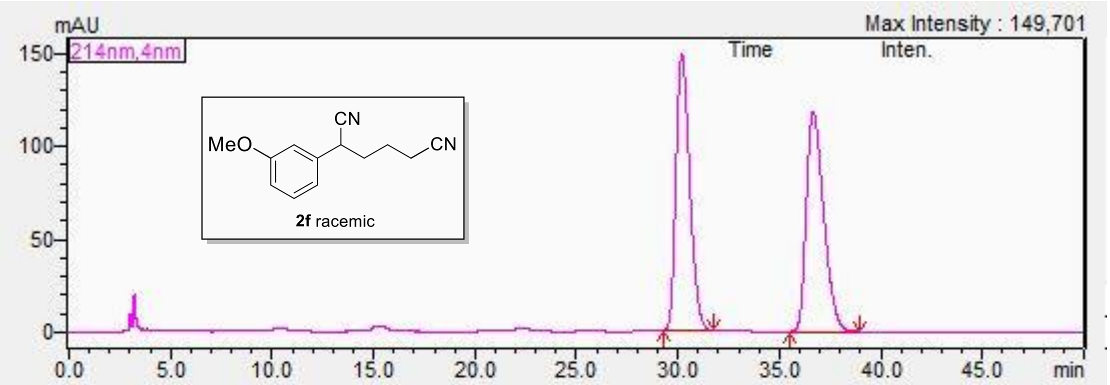

Peak	Ret. time	Area %	Height	Area
1	30.195	49.821	148727	6998292
2	36.668	50.179	118283	7048545
Totol:		100.000	267010	14046838

Supplementary Figure 179. HPLC data of rac-**2f**

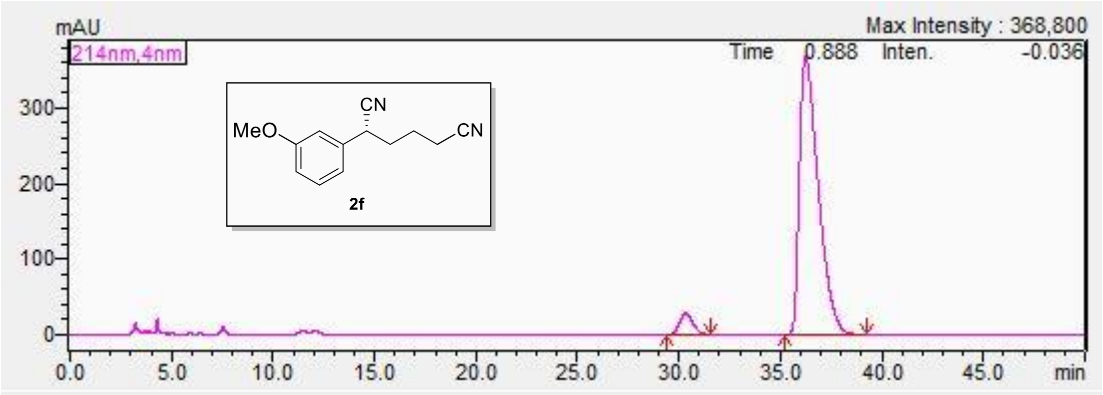

Peak	Ret. time	Area %	Height	Area
1	30.344	5.132	28150	1287689
2	36.248	94.868	369147	23801454
Totol:		100.000	397297	25089144

Supplementary Figure 180. HPLC data of **2f**

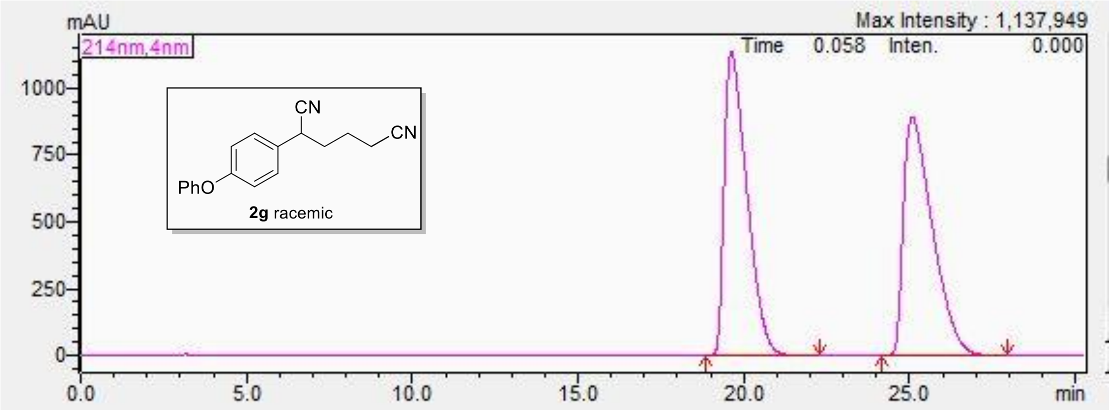

Peak	Ret. time	Area %	Height	Area
1	19.626	50.026	1137173	54153369
2	25.080	49.974	892649	54096018
Totol:		100.000	2029822	108249387

Supplementary Figure 181. HPLC data of rac-**2g**

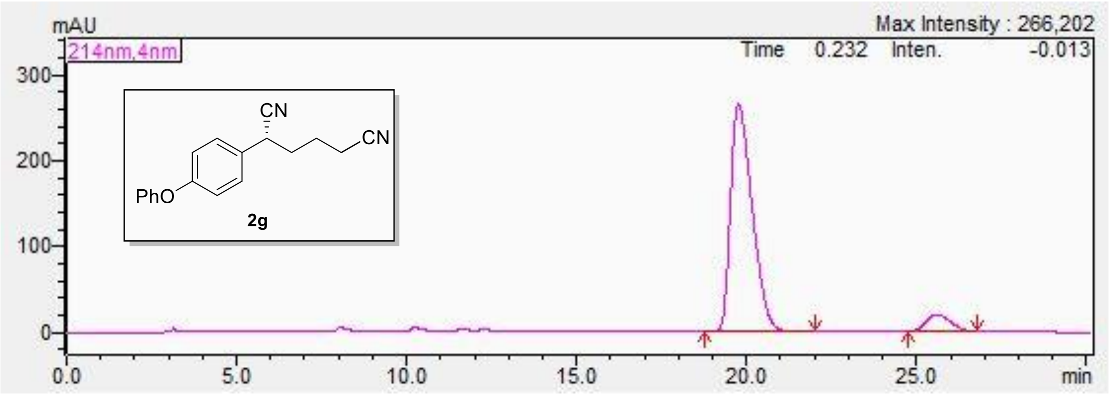

Peak	Ret. time	Area %	Height	Area
1	19.768	92.113	266013	11788503
2	25.611	7.887	19922	1009358
Totol:		100.000	285935	12797861

Supplementary Figure 182. HPLC data of **2g**

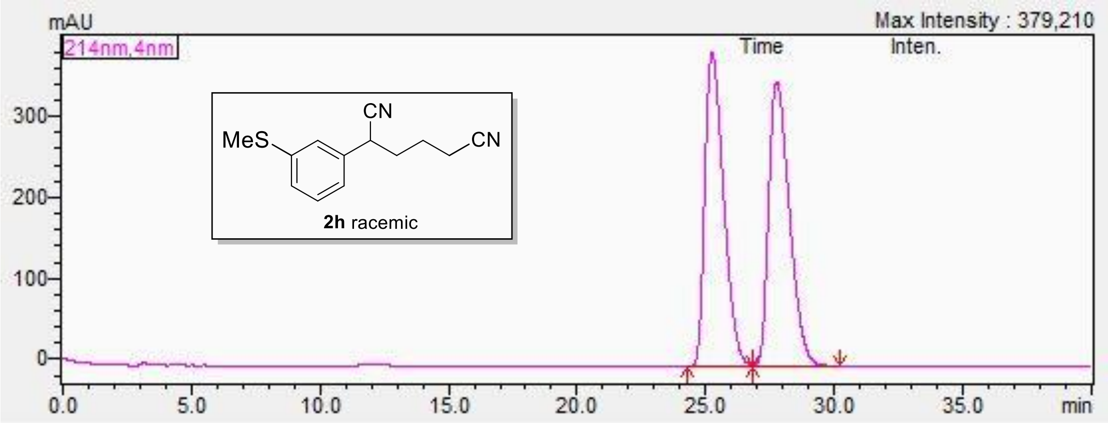

Peak	Ret. time	Area %	Height	Area
1	25.251	49.938	387603	19641132
2	27.766	50.062	351511	19690091
Totol:		100.000	739114	39331223

Supplementary Figure 183. HPLC data of rac-**2h**

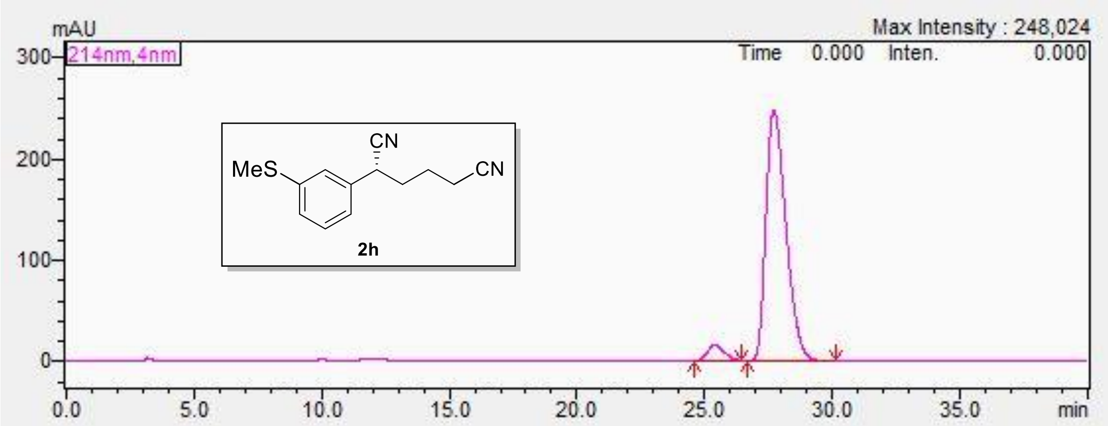

Peak	Ret. time	Area %	Height	Area
1	25.406	4.938	15150	699557
2	27.717	95.062	247380	13467988
Totol:		100.000	262530	14167545

Supplementary Figure 184. HPLC data of **2h**

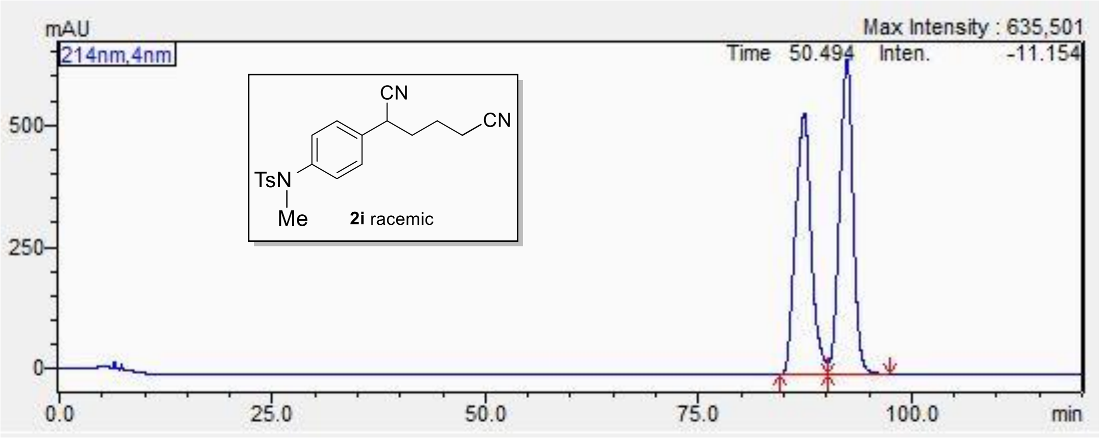

Peak	Ret. time	Area %	Height	Area
1	87.405	49.181	536227	68926239
2	92.439	50.819	645825	71222674
Totol:		100.000	1182052	140148913

Supplementary Figure 185. HPLC data of rac-**2i**

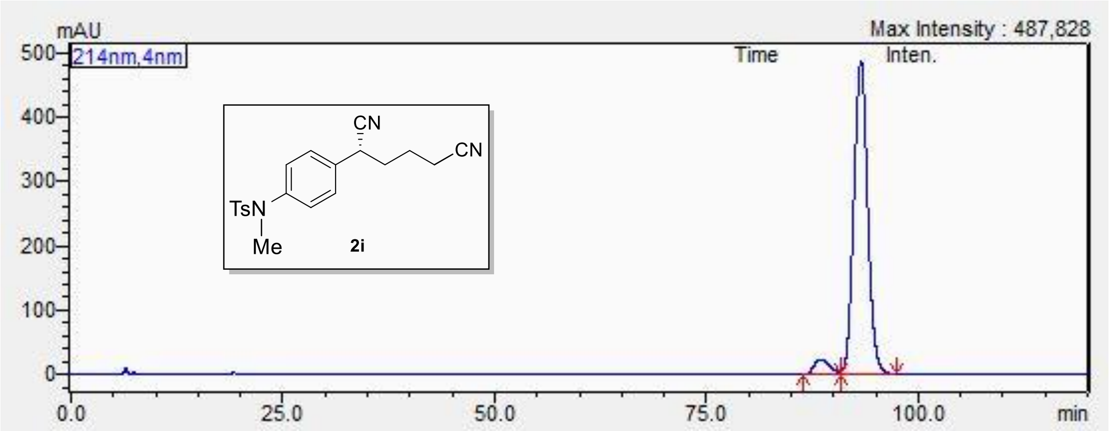

Peak	Ret. time	Area %	Height	Area
1	88.428	5.106	23141	2975965
2	93.250	94.894	487477	55311320
Totol:		100.000	510618	58287285

Supplementary Figure 186. HPLC data of **2i**

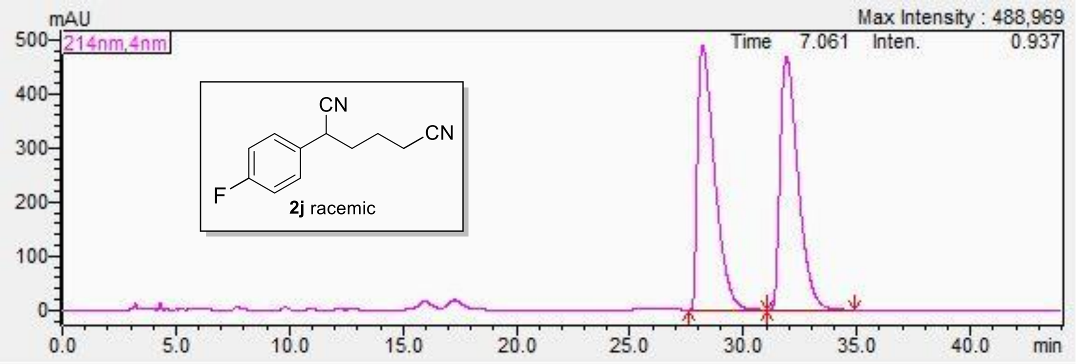

Peak	Ret. time	Area %	Height	Area
1	28.209	49.830	488000	25533343
2	31.903	50.170	468146	25707213
Totol:		100.000	956146	51240556

Supplementary Figure 187. HPLC data of rac-**2j**

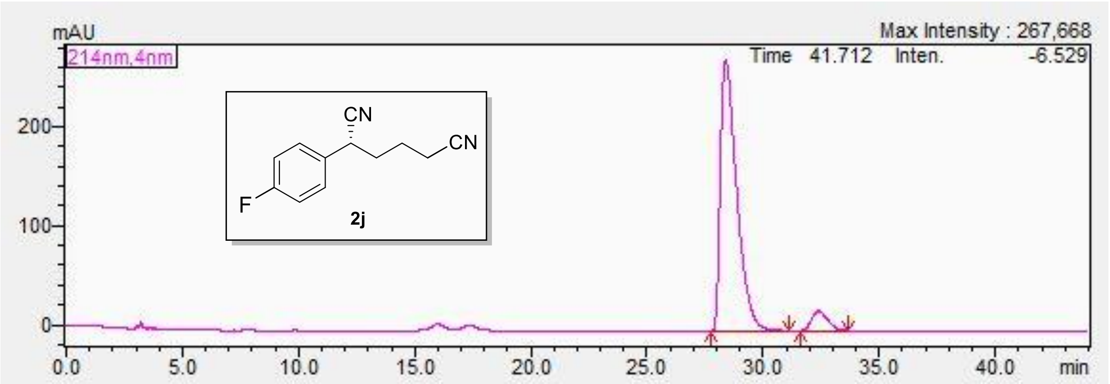

Peak	Ret. time	Area %	Height	Area
1	28.395	93.411	273800	13400075
2	32.390	6.589	19842	945145
Totol:		100.000	293642	14345220

Supplementary Figure 188. HPLC data of **2j**

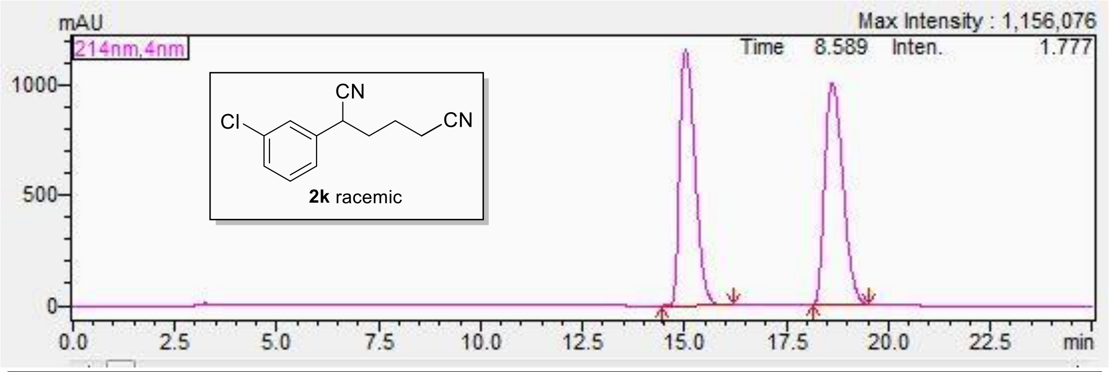

Peak	Ret. time	Area %	Height	Area
1	15.030	49.243	1154683	30072261

2	18.631	50.757	1001967	30996533
Totol:		100.000	2156651	61068795

Supplementary Figure 189. HPLC data of rac-**2k**

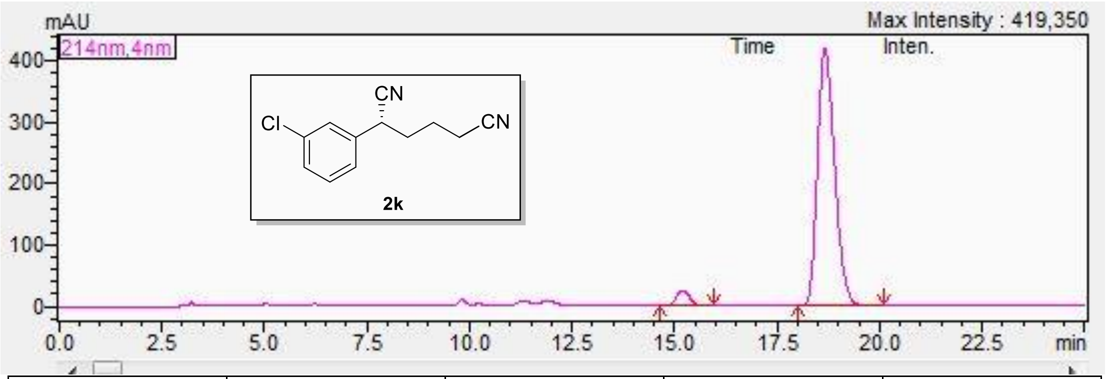

Peak	Ret. time	Area %	Height	Area
1	15.199	4.582	25682	588358
2	18.672	95.418	418529	12253502
Totol:		100.000	444211	12841860

Supplementary Figure 190. HPLC data of **2k**

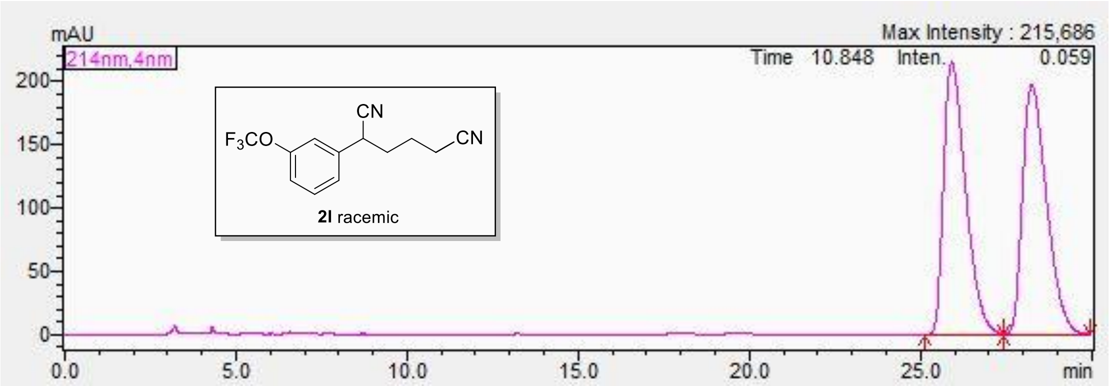

Peak	Ret. time	Area %	Height	Area
1	25.917	50.005	215726	9676116
2	28.255	49.995	197350	9674355
Totol:		100.000	413076	19350471

Supplementary Figure 191. HPLC data of rac-**2l**

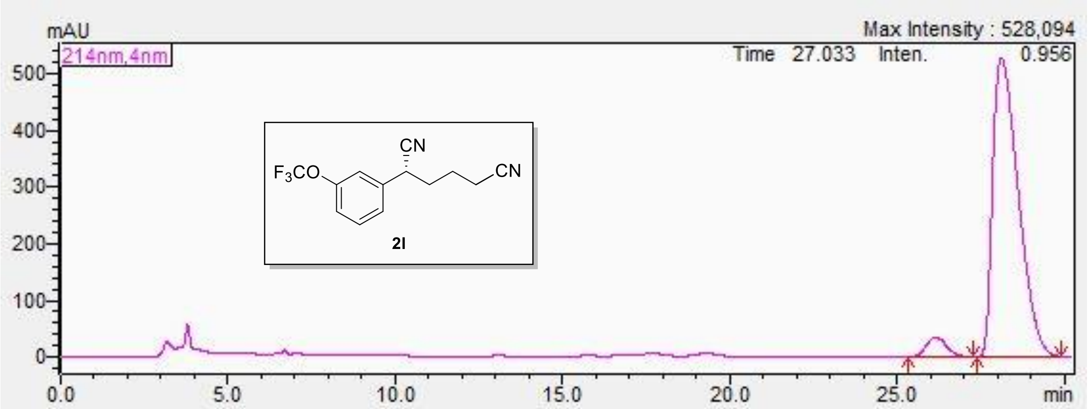

Peak	Ret. time	Area %	Height	Area
1	26.144	4.952	34865	1461850
2	28.107	95.048	527220	28056044
Totol:		100.000	562085	29517894

Supplementary Figure 192. HPLC data of **2l**

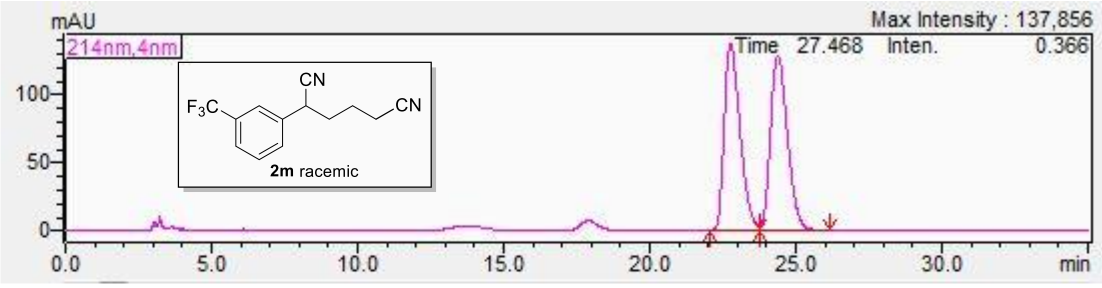

Peak	Ret. time	Area %	Height	Area
1	22.769	49.807	137238	5166377
2	24.388	50.193	128646	5206397
Totol:		100.000	265884	10372774

Supplementary Figure 193. HPLC data of rac-**2m**

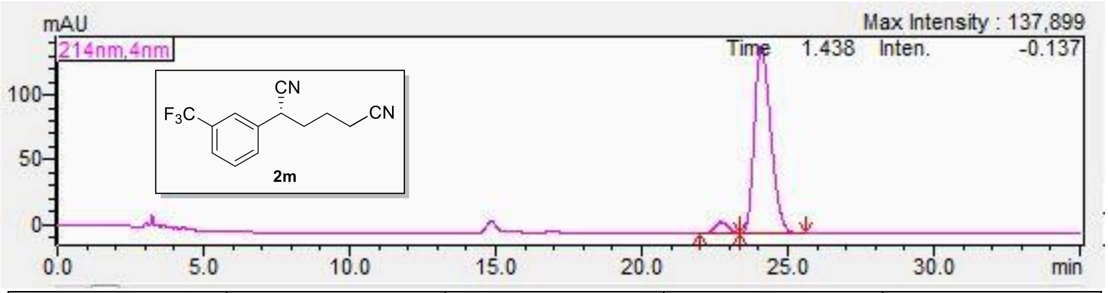

Peak	Ret. time	Area %	Height	Area
1	22.711	5.186	8953	311115
2	24.099	94.814	145075	5687658
Totol:		100.000	154028	5998773

Supplementary Figure 194. HPLC data of **2m**

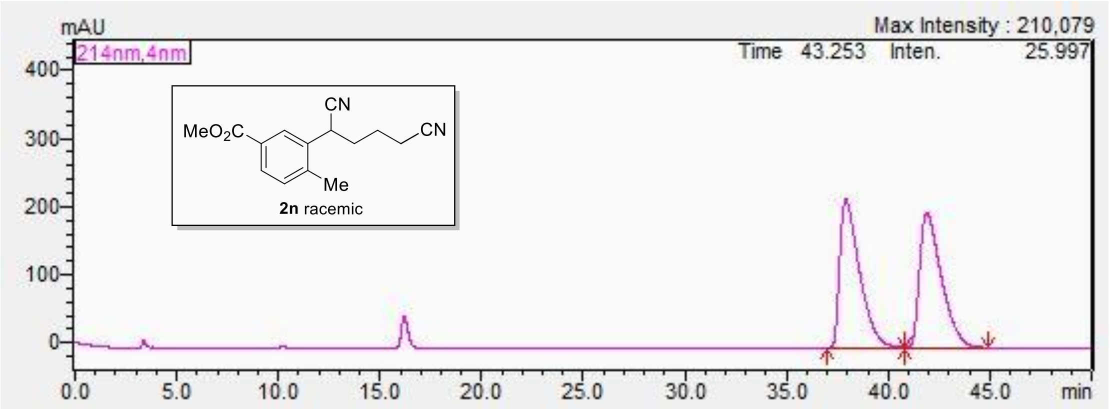

Peak	Ret. time	Area %	Height	Area
1	37.921	49.891	217240	14708704
2	41.901	50.109	198191	14772915
Totol:		100.000	415431	29481619

Supplementary Figure 195. HPLC data of rac-**2n**

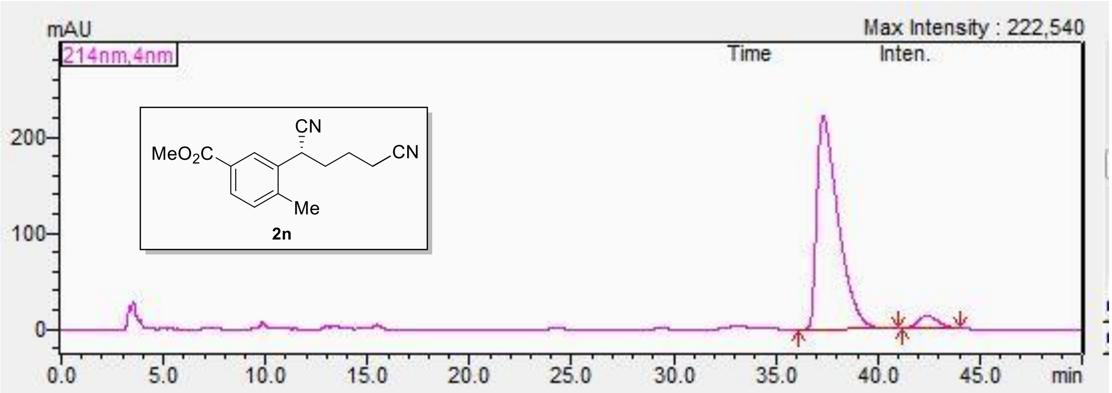

Peak	Ret. time	Area %	Height	Area
1	37.331	94.520	222561	16137746
2	42.414	5.480	13380	935658
Totol:		100.000	235942	17073404

Supplementary Figure 196. HPLC data of **2n**

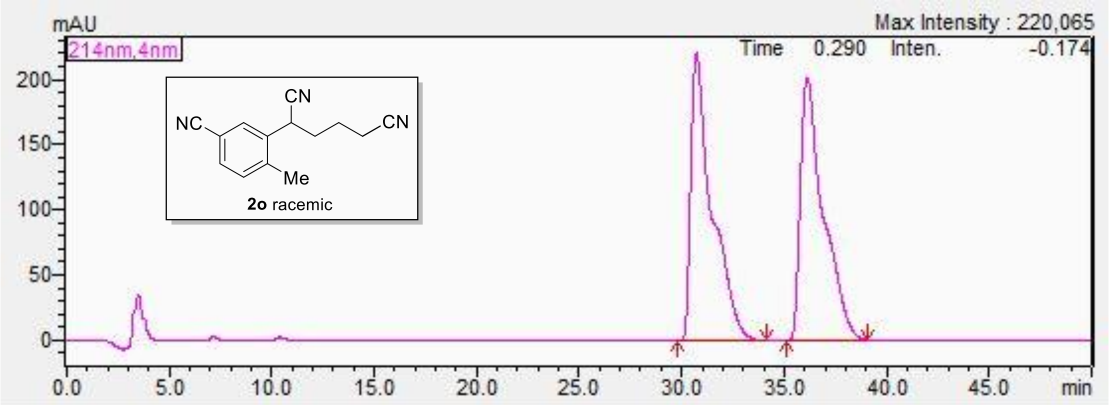

Peak	Ret. time	Area %	Height	Area
1	30.728	50.177	220568	15823659
2	36.130	49.823	202173	15711789
Totol:		100.000	422741	31535448

Supplementary Figure 197. HPLC data of rac-**2o**

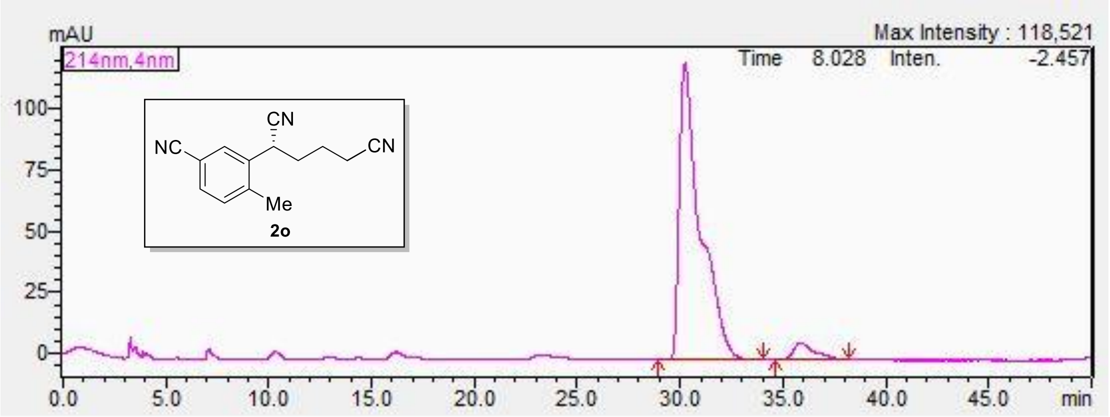

Peak	Ret. time	Area %	Height	Area
1	30.239	94.459	121137	8529419
2	35.870	5.541	6941	500312
Totol:		100.000	128078	9029731

Supplementary Figure 198. HPLC data of **2o**

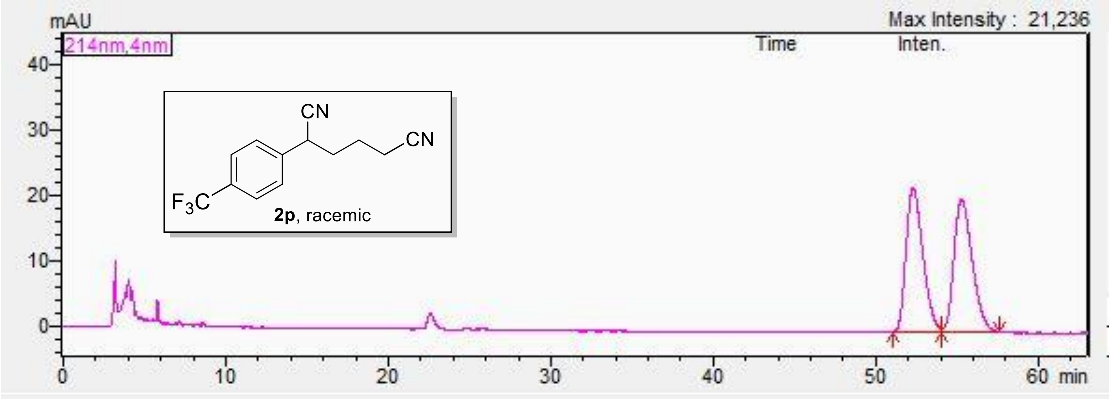

Peak	Ret. time	Area %	Height	Area
1	52.292	49.880	22051	1593710
2	55.272	50.120	20413	1601383
Totol:		100.000	42464	3195093

Supplementary Figure 199. HPLC data of rac-**2p**

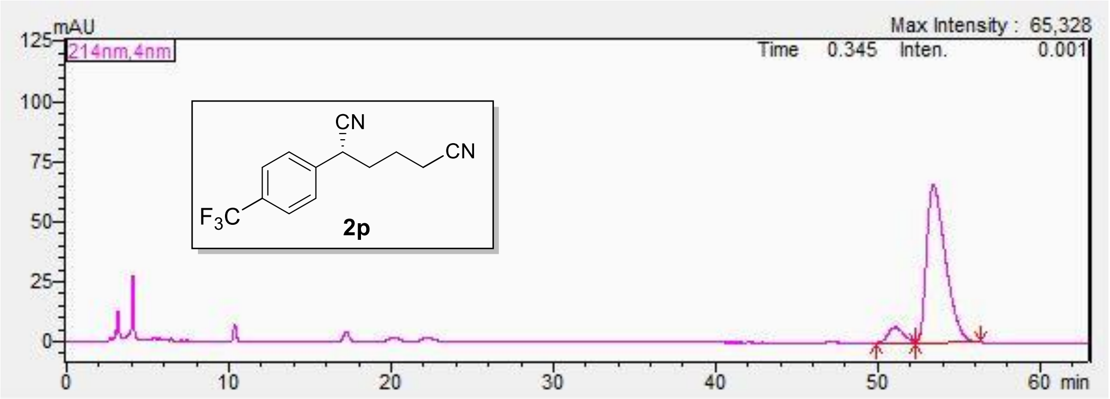

Peak	Ret. time	Area %	Height	Area
1	51.073	7.321	6332	420558
2	53.432	92.679	65675	5324199
Totol:		100.000	72007	5744757

Supplementary Figure 200. HPLC data of **2p**

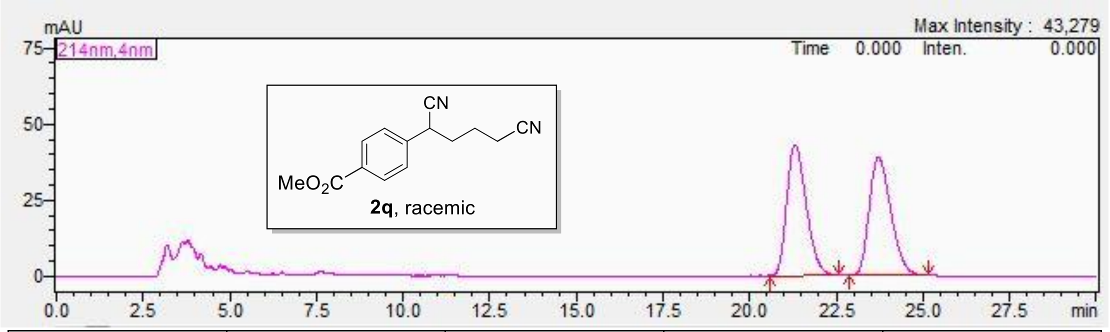

Peak	Ret. time	Area %	Height	Area
1	21.314	49.879	43090	1687552

2	23.719	50.121	38950	1695773
Totol:		100.000	82040	3383326

Supplementary Figure 201. HPLC data of rac-**2q**

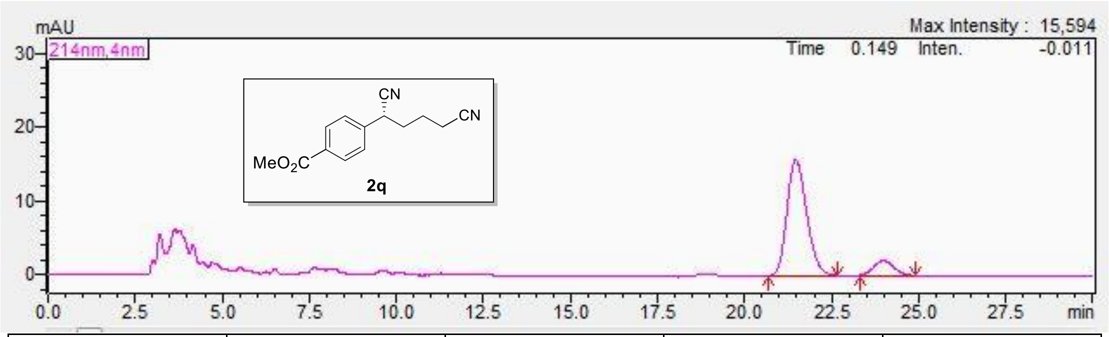

Peak	Ret. time	Area %	Height	Area
1	21.474	88.187	15690	615179
2	23.997	11.813	2002	82407
Totol:		100.000	17692	697585

Supplementary Figure 202. HPLC data of **2q**

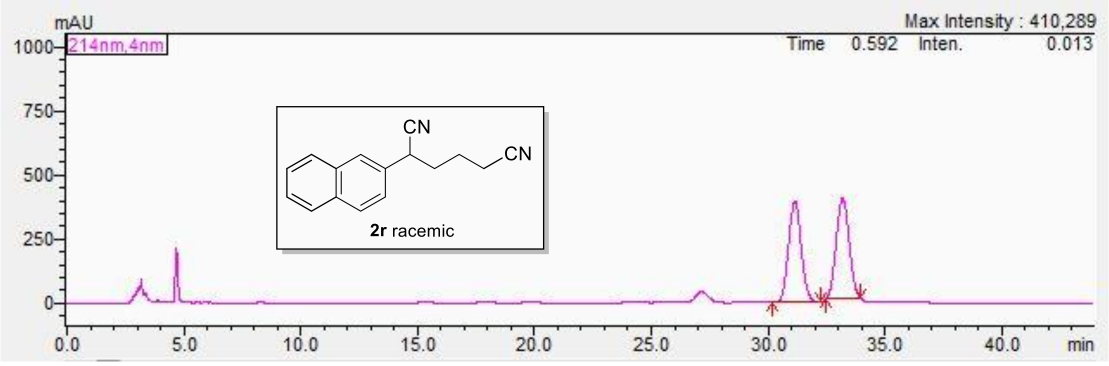

Peak	Ret. time	Area %	Height	Area
1	31.144	49.478	394152	14766900
2	33.190	50.522	392866	15078214
Totol:		100.000	787018	29845114

Supplementary Figure 203. HPLC data of rac-**2r**

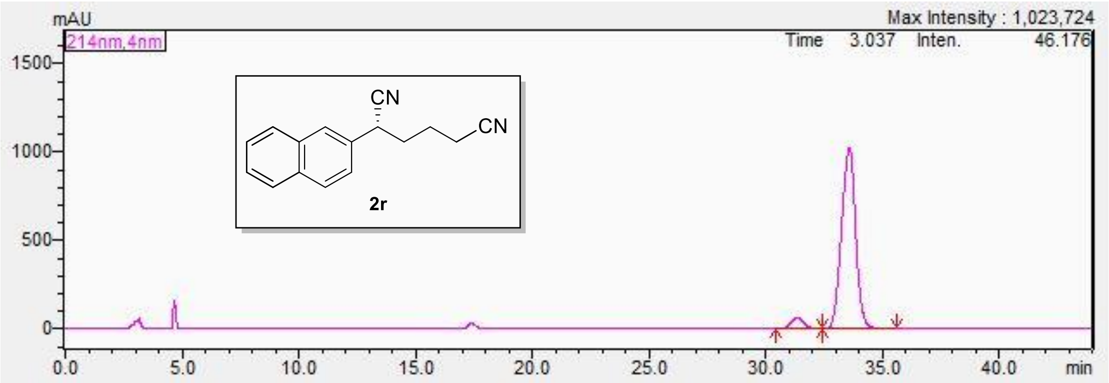

Peak	Ret. time	Area %	Height	Area
1	31.357	5.119	60979	2325608
2	33.606	94.881	1022333	43101992
Totol:		100.000	1083312	45427600

Supplementary Figure 204. HPLC data of **2r**

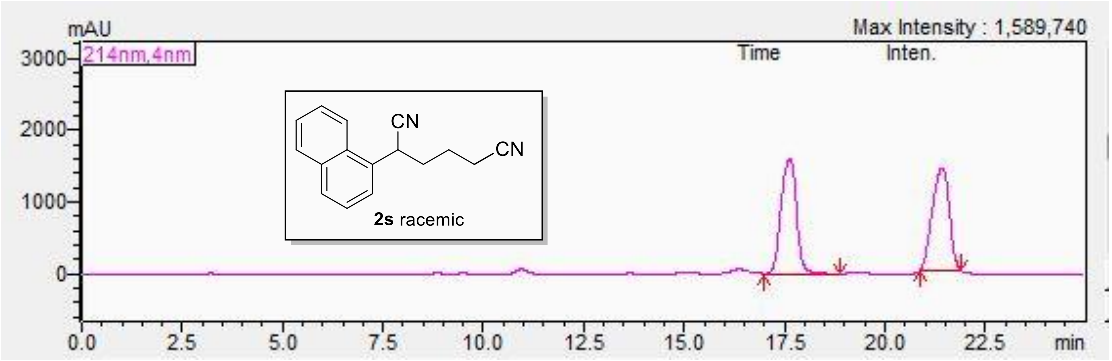

Peak	Ret. time	Area %	Height	Area
1	17.630	49.920	1587922	42303287
2	21.429	50.080	1433941	42439452
Totol:		100.000	3021863	84742739

Supplementary Figure 205. HPLC data of rac-**2s**

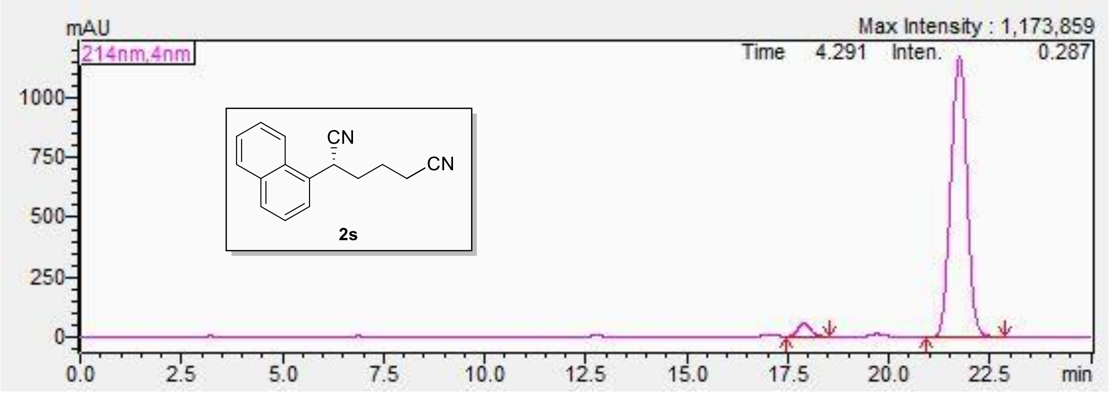

Peak	Ret. time	Area %	Height	Area

1	17.893	3.429	55504	1150657
2	21.748	96.571	1173178	32410824
Totol:		100.000	1228682	33561482

Supplementary Figure 206. HPLC data of **2s**

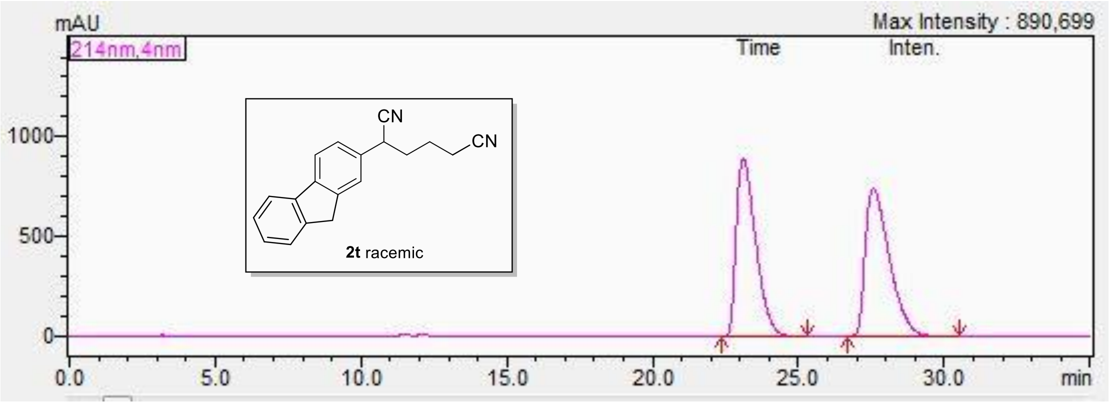

Peak	Ret. time	Area %	Height	Area
1	23.109	49.471	890346	41525070
2	27.571	50.529	740700	42412671
Totol:		100.000	1631047	83937741

Supplementary Figure 207. HPLC data of rac-**2t**

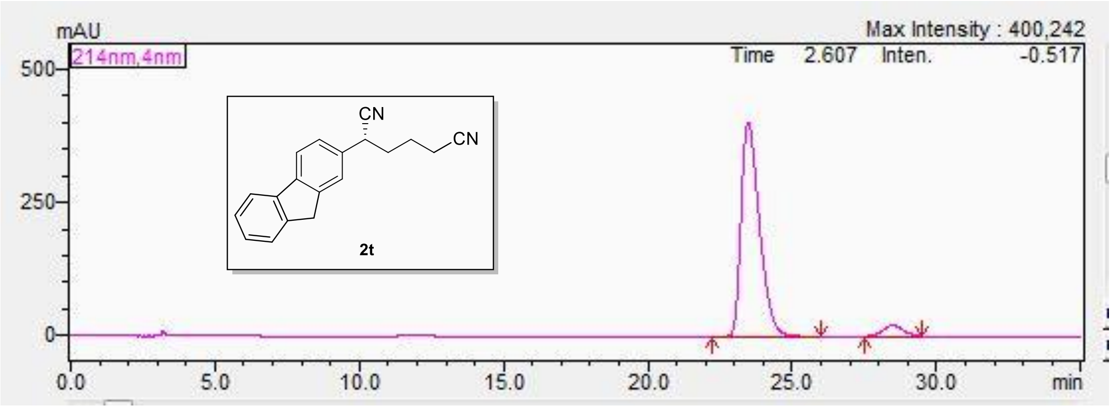

Peak	Ret. time	Area %	Height	Area
1	23.479	94.782	401146	17629646
2	28.475	5.218	19786	970623
Totol:		100.000	420933	18600268

Supplementary Figure 208. HPLC data of **2t**

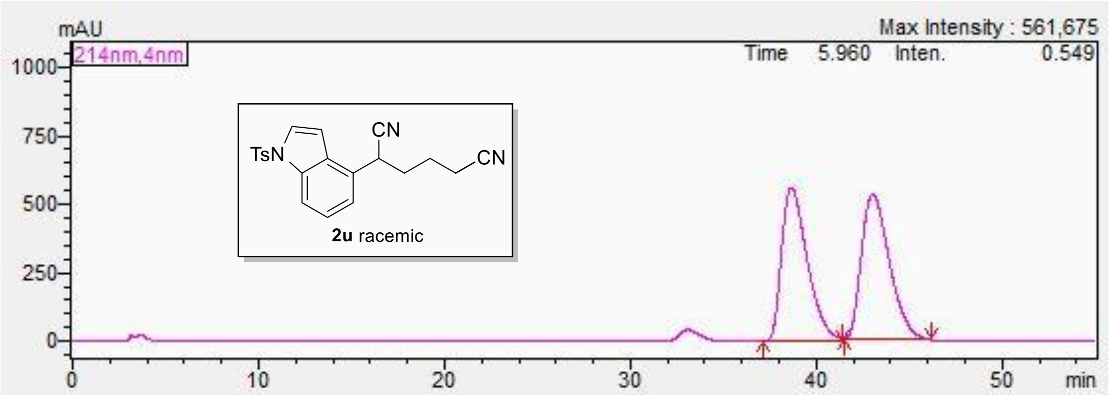

Peak	Ret. time	Area %	Height	Area
1	38.653	49.412	561909	54258269
2	43.032	50.588	529866	55550667
Totol:		100.000	1091775	109808937

Supplementary Figure 209. HPLC data of rac-**2u**

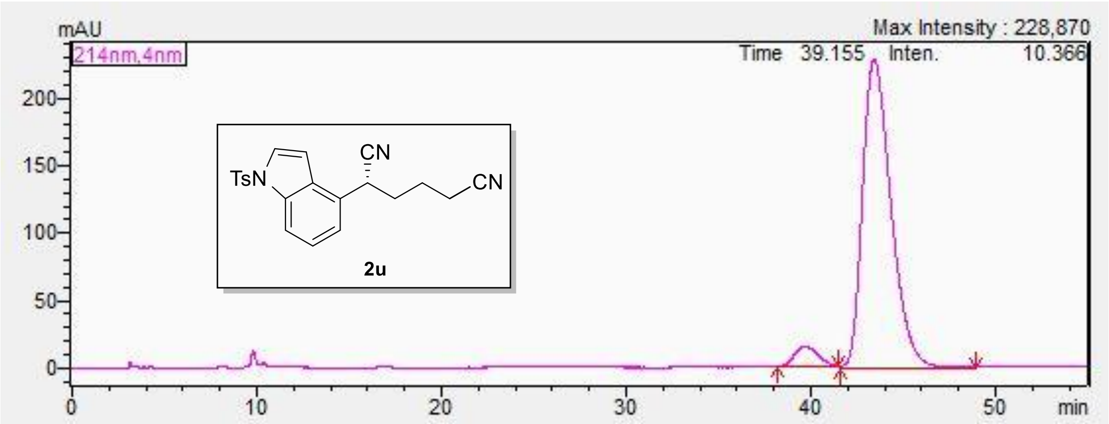

Peak	Ret. time	Area %	Height	Area
1	39.691	5.490	15362	1418277
2	43.442	94.510	228499	24413604
Totol:		100.000	243861	25831880

Supplementary Figure 210. HPLC data of **2u**

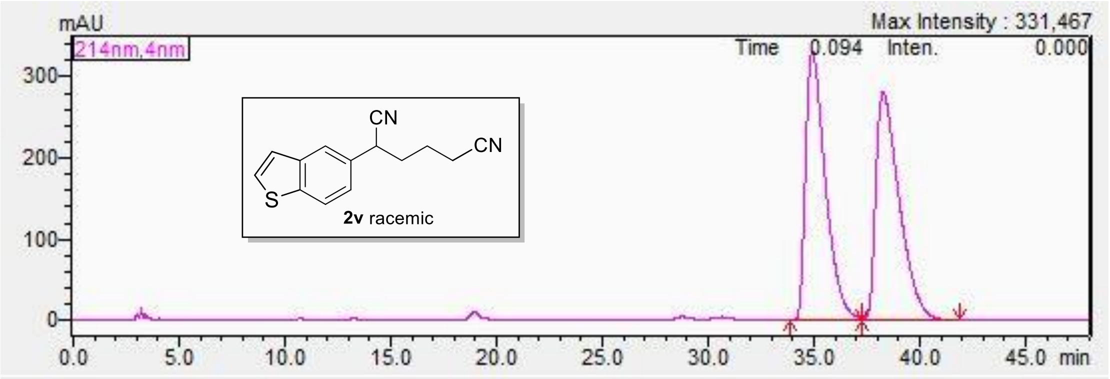

Peak	Ret. time	Area %	Height	Area
1	34.918	49.918	330654	21469335
2	38.271	50.082	280114	21540206
Totol:		100.000	610768	43009541

Supplementary Figure 211. HPLC data of rac-**2v**

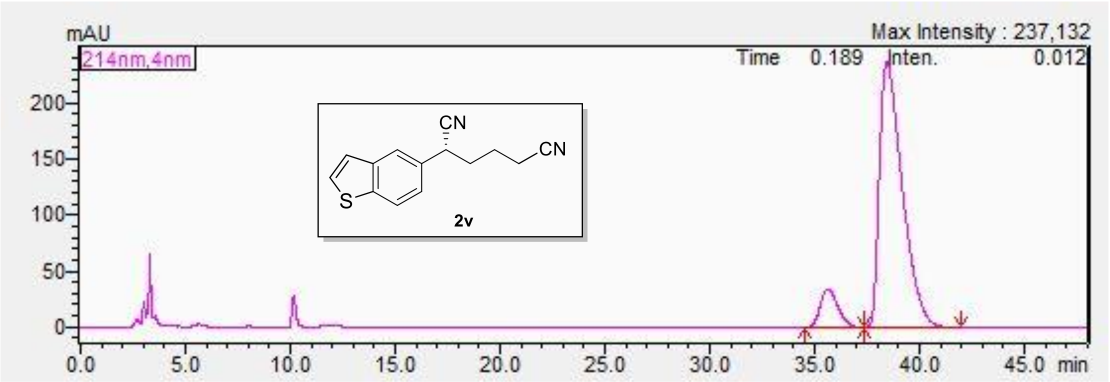

Peak	Ret. time	Area %	Height	Area
1	35.620	10.200	34455	2050545
2	38.441	89.800	237835	18051911
Totol:		100.000	272290	20102456

Supplementary Figure 212. HPLC data of **2v**

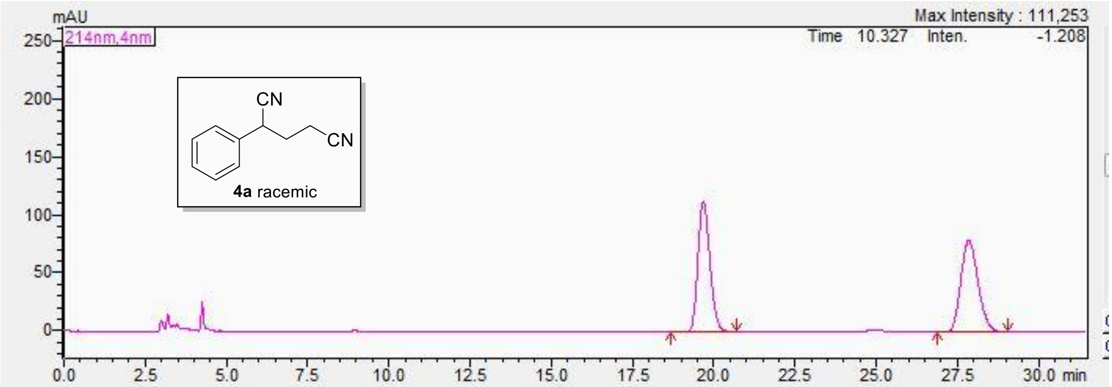

Peak	Ret. time	Area %	Height	Area
1	19.683	49.936	112292	2893054
2	27.849	50.064	79035	2900517
Totol:		100.000	191327	5793570

Supplementary Figure 213. HPLC data of rac-**4a**

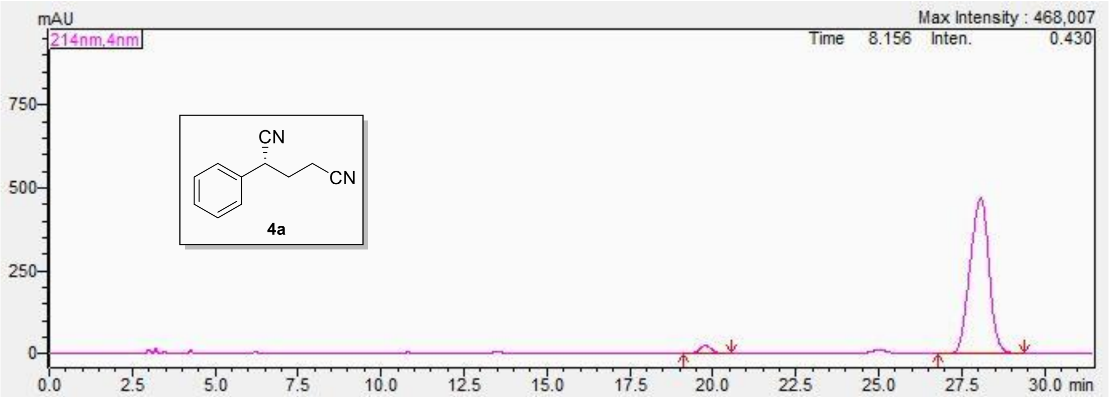

Peak	Ret. time	Area %	Height	Area
1	19.769	3.218	23998	615140
2	28.074	96.782	467582	18502967
Totol:		100.000	491579	19118107

Supplementary Figure 214. HPLC data of **4a**

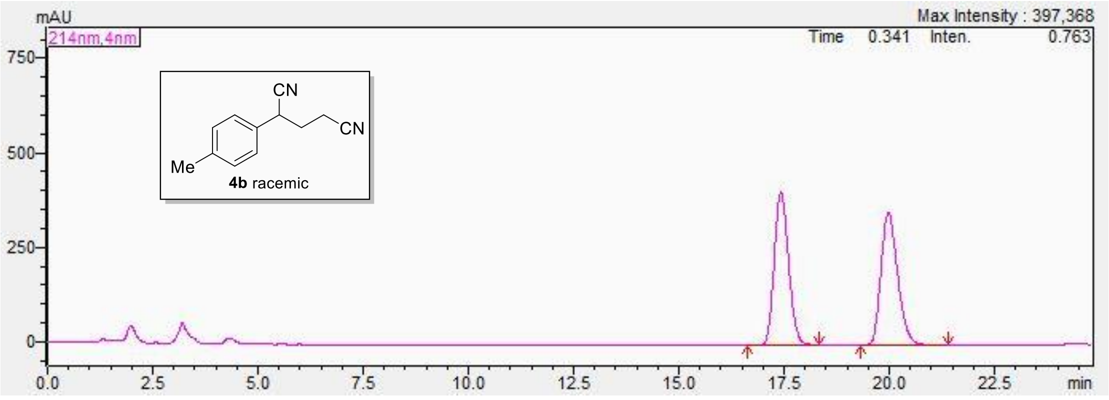

Peak	Ret. time	Area %	Height	Area
1	17.428	49.776	405705	9525417
2	19.982	50.224	348130	9611207
Totol:		100.000	753835	19136624

Supplementary Figure 215. HPLC data of rac-**4b**

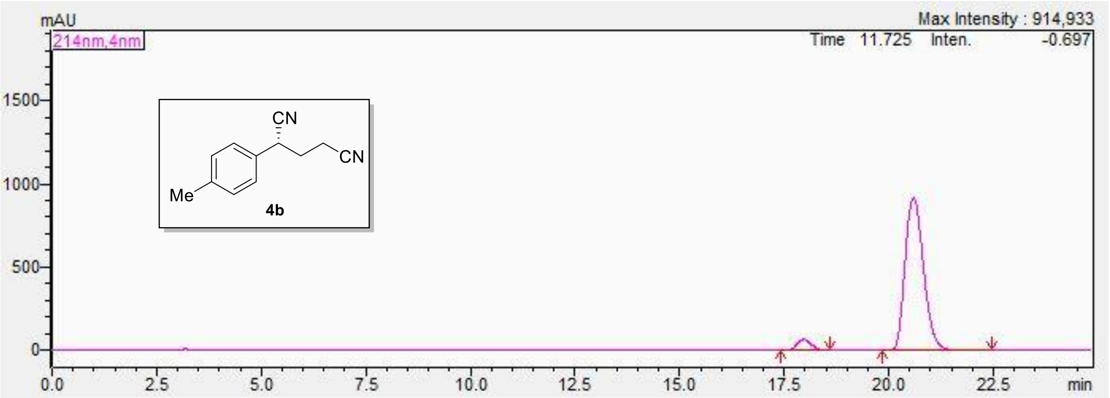

Peak	Ret. time	Area %	Height	Area
1	17.970	5.286	65706	1575165
2	20.592	94.714	915454	28221854
Totol:		100.000	981160	29797019

Supplementary Figure 216. HPLC data of **4b**

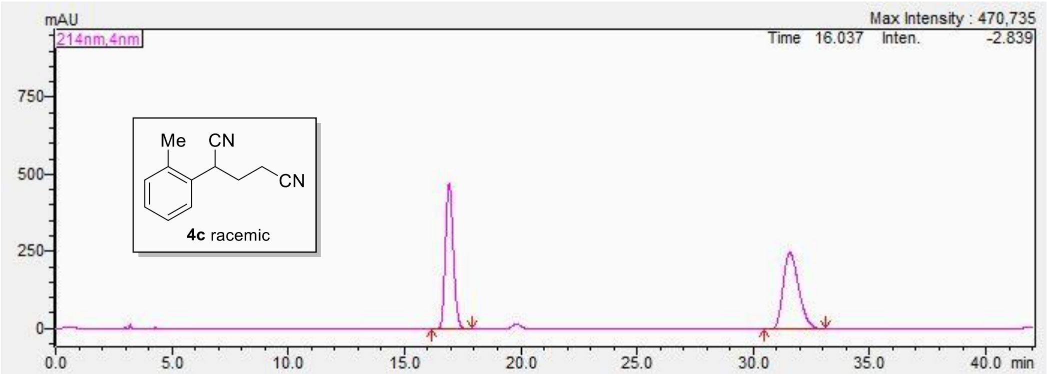

Peak	Ret. time	Area %	Height	Area
1	16.919	49.429	473320	11367944
2	31.567	50.571	248812	11630440
Totol:		100.000	722132	22998384

Supplementary Figure 217. HPLC data of rac-**4c**

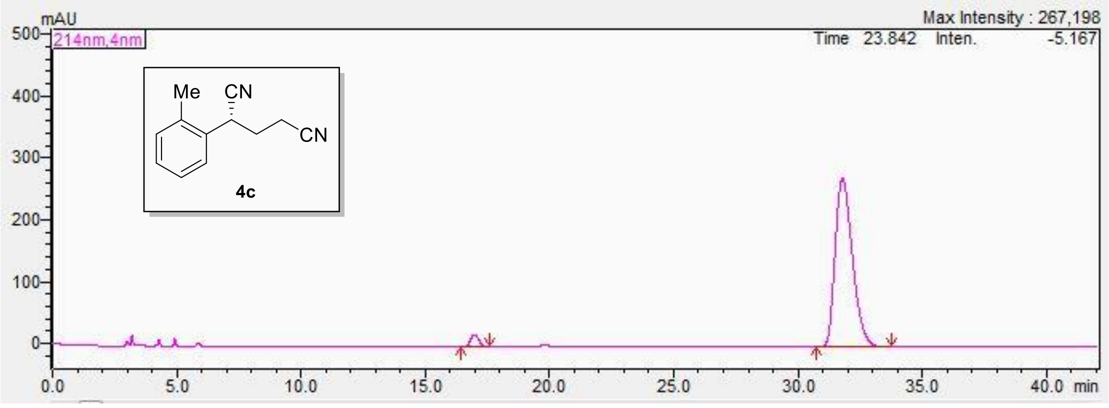

Peak	Ret. time	Area %	Height	Area
1	16.984	3.445	19793	468556
2	31.797	96.555	272589	13133932
Totol:		100.000	292382	13602487

Supplementary Figure 218. HPLC data of **4c**

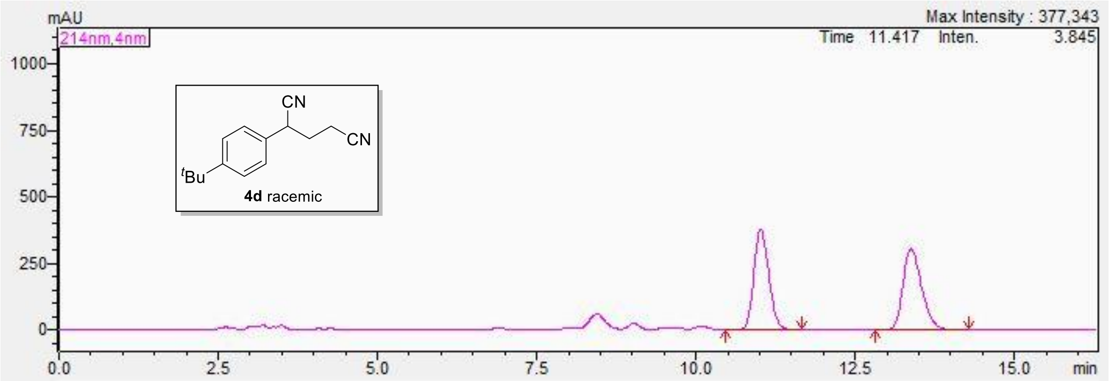

Peak	Ret. time	Area %	Height	Area
1	11.017	49.818	376890	6143830
2	13.377	50.182	303617	6188718
Totol:		100.000	680507	12332548

Supplementary Figure 219. HPLC data of rac-**4d**

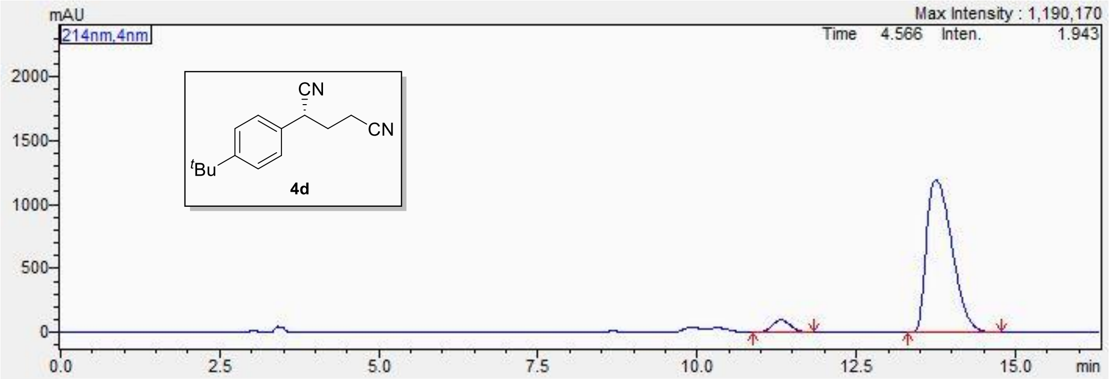

Peak	Ret. time	Area %	Height	Area
1	11.314	5.472	96692	1928361
2	13.752	94.528	1189249	33312362
Totol:		100.000	1285941	35240723

Supplementary Figure 220. HPLC data of **4d**

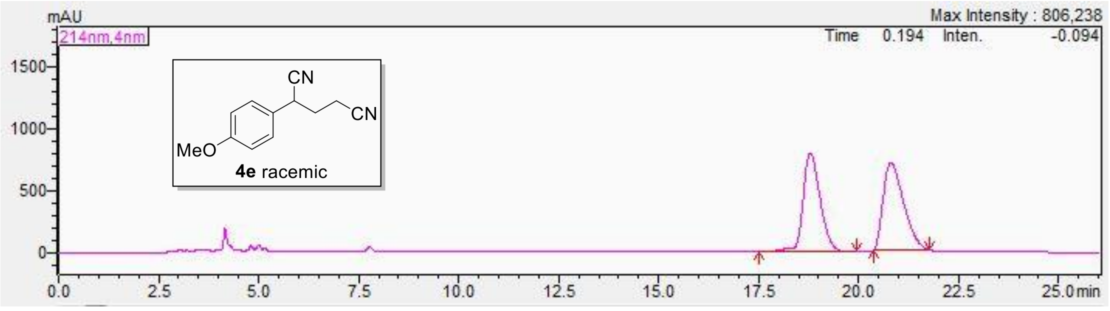

Peak	Ret. time	Area %	Height	Area
1	18.794	49.328	800897	24073404
2	20.810	50.672	702330	24728972
Totol:		100.000	1503226	48802376

Supplementary Figure 221. HPLC data of rac-**4e**

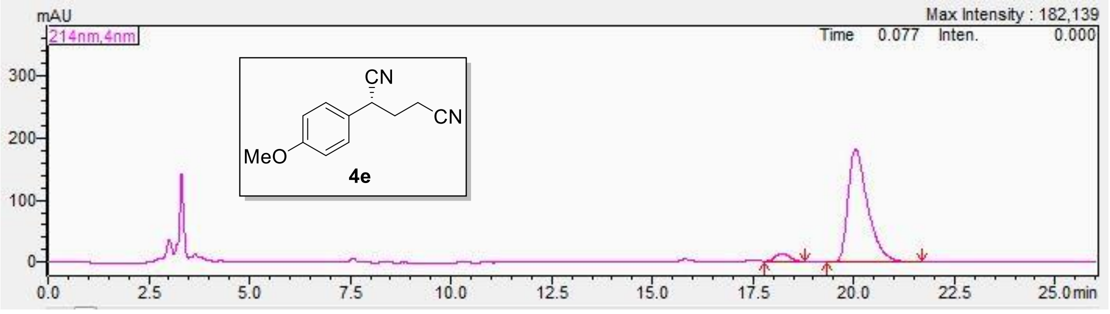

Peak	Ret. time	Area %	Height	Area
1	18.223	5.131	13393	332771
2	20.046	94.869	181767	6152745
Totol:		100.000	195160	6485516

Supplementary Figure 222. HPLC data of **4e**

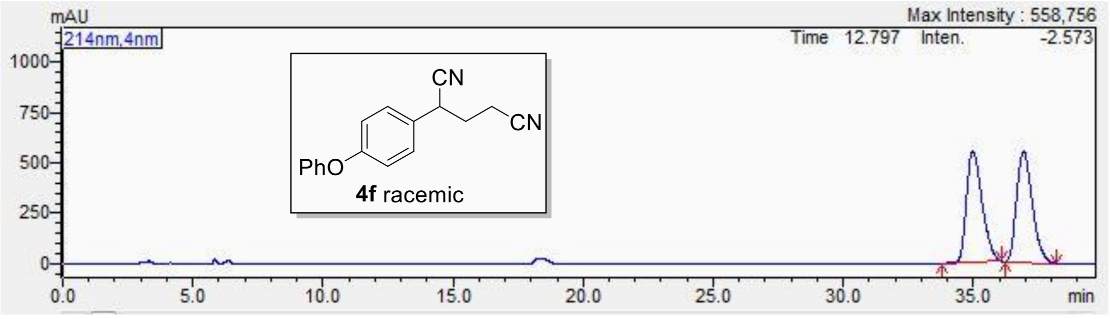

Peak	Ret. time	Area %	Height	Area
1	34.985	50.131	553878	23348401
2	36.931	49.869	552318	23226652
Totol:		100.000	1106197	46575053

Supplementary Figure 223. HPLC data of rac-**4f**

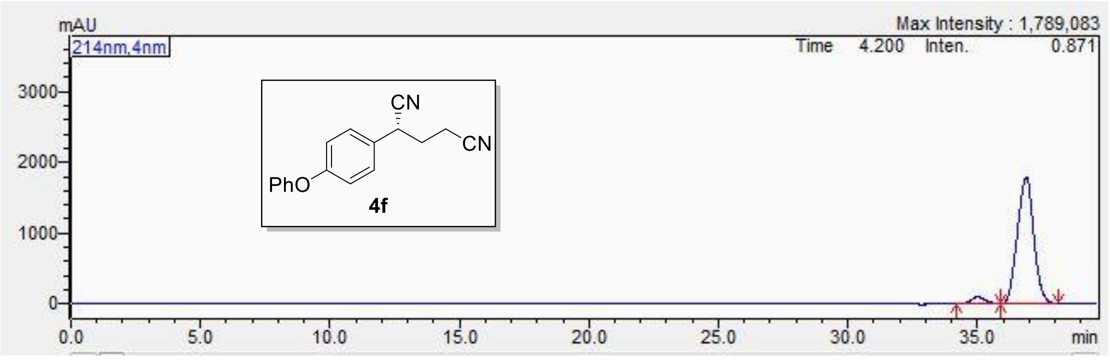

Peak	Ret. time	Area %	Height	Area
1	35.016	4.388	92178	3619634
2	36.894	95.612	1784119	78876356
Totol:		100.000	1876297	82495990

Supplementary Figure 224. HPLC data of **4f**

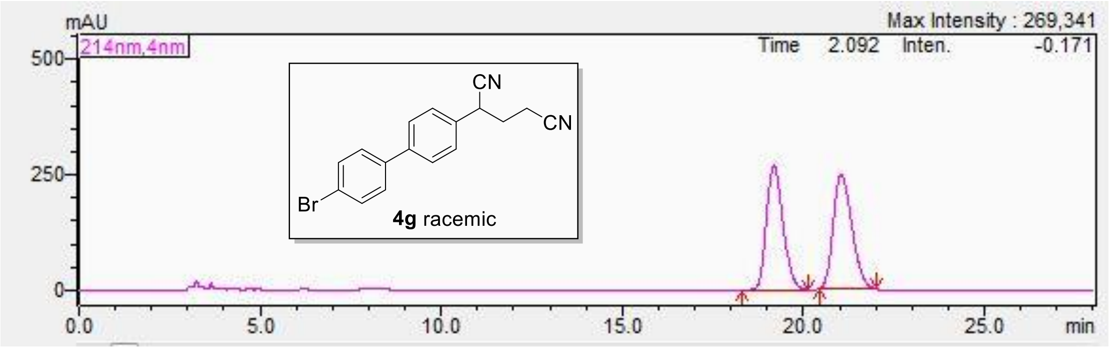

Peak	Ret. time	Area %	Height	Area
1	19.181	49.056	268396	8545400
2	21.045	50.944	248521	8874453
Totol:		100.000	516917	17419853

Supplementary Figure 225. HPLC data of rac-**4g**

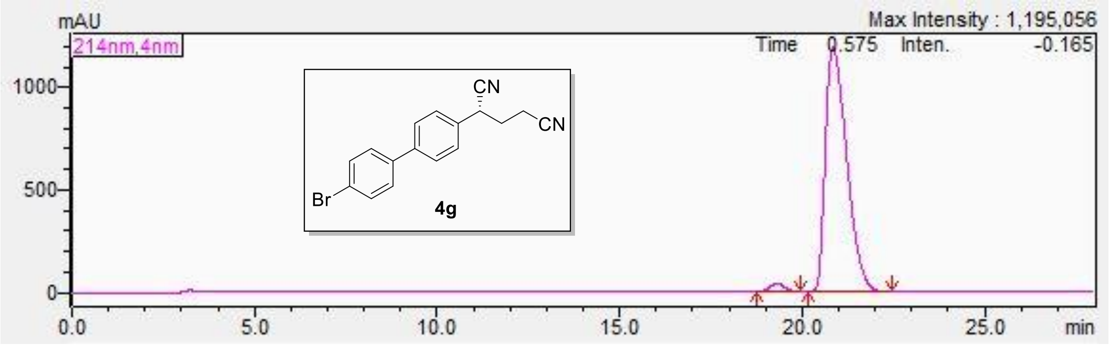

Peak	Ret. time	Area %	Height	Area
1	19.309	2.219	38118	1108224
2	20.850	97.781	1193211	48834361
Totol:		100.000	1231329	49942586

Supplementary Figure 226. HPLC data of **4g**

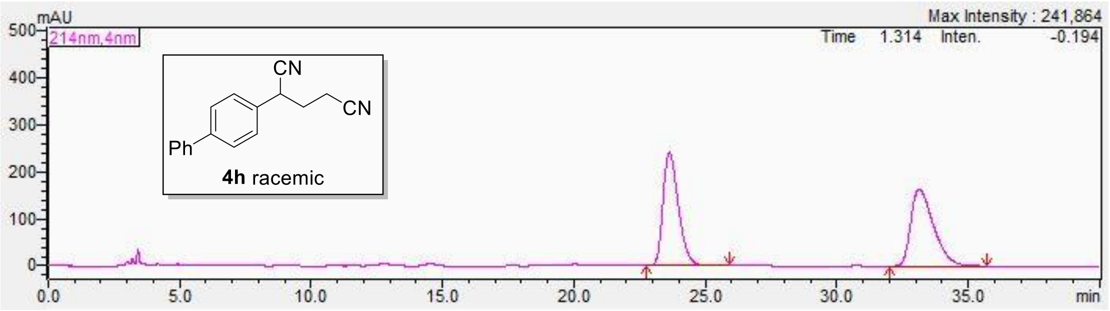

Peak	Ret. time	Area %	Height	Area
1	23.629	49.996	241600	9824775
2	33.134	50.004	164087	9826156
Totol:		100.000	405687	19650931

Supplementary Figure 227. HPLC data of rac-**4h**

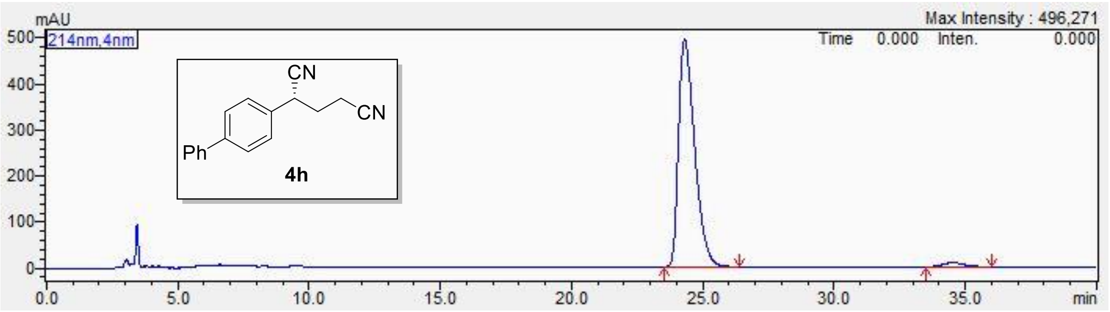

Peak	Ret. time	Area %	Height	Area

1	24.306	97.709	493055	21018489
2	34.523	2.291	8674	492805
Totol:		100.000	501730	21511295

Supplementary Figure 228. HPLC data of **4h**

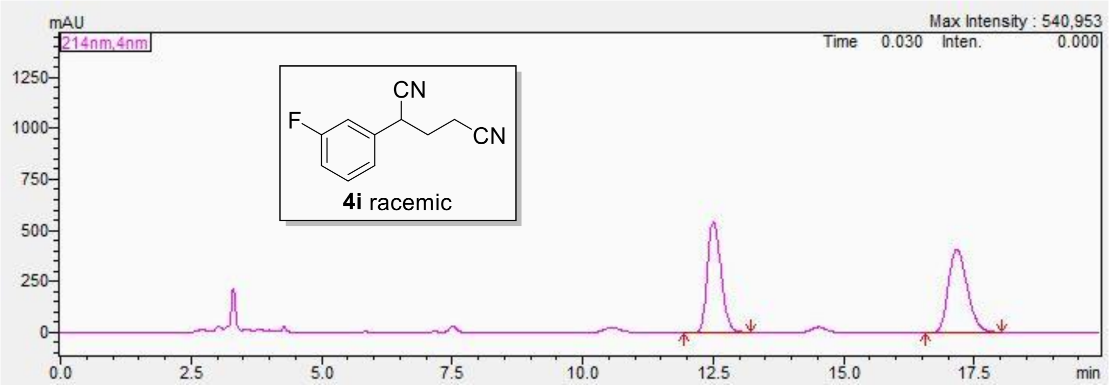

Peak	Ret. time	Area %	Height	Area
1	12.505	49.143	540240	9831993
2	17.163	50.857	405770	10175057
Totol:		100.000	946010	20007050

Supplementary Figure 229. HPLC data of rac-**4i**

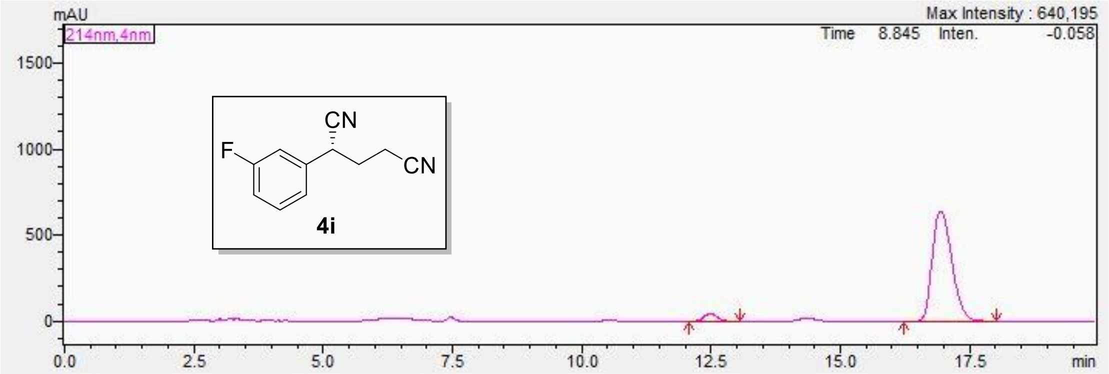

Peak	Ret. time	Area %	Height	Area
1	12.483	4.360	44777	770893
2	16.942	95.640	639849	16909681
Totol:		100.000	684626	17680574

Supplementary Figure 230. HPLC data of **4i**

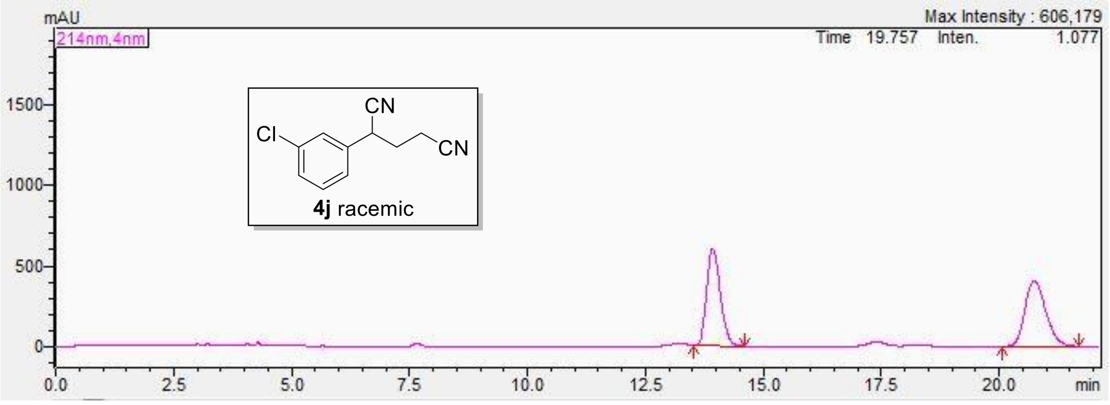

Peak	Ret. time	Area %	Height	Area
1	13.928	49.282	600345	12090757
2	20.743	50.718	407201	12443113
Totol:		100.000	1007546	24533870

Supplementary Figure 231. HPLC data of rac-**4j**

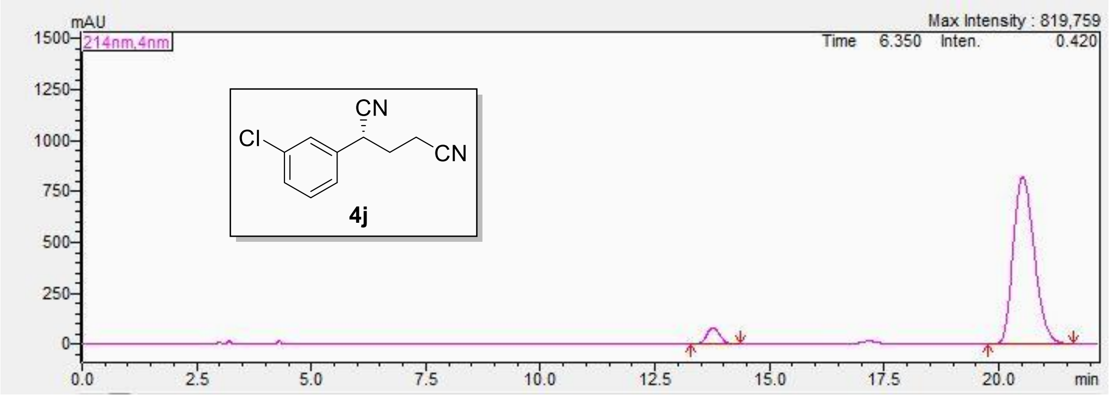

Peak	Ret. time	Area %	Height	Area
1	13.762	5.493	79956	1531043
2	20.523	94.507	819054	26343527
Totol:		100.000	899010	27874570

Supplementary Figure 232. HPLC data of **4j**

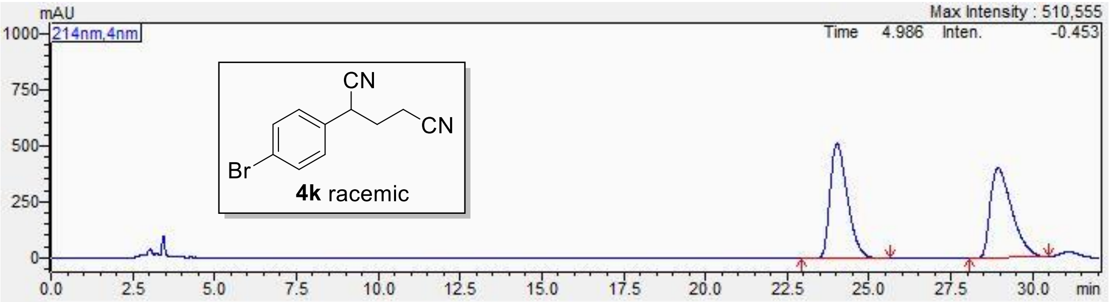

Peak	Ret. time	Area %	Height	Area

1	24.020	50.609	512053	18861078
2	28.941	49.391	401610	18406820
Totol:		100.000	913663	37267898

Supplementary Figure 233. HPLC data of rac-**4k**

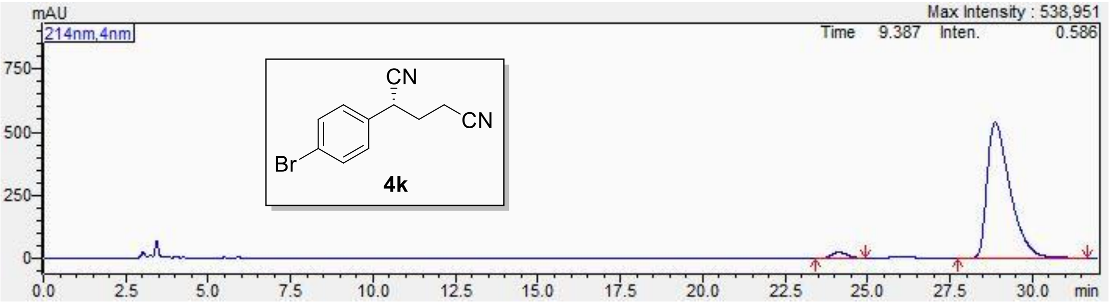

Peak	Ret. time	Area %	Height	Area
1	24.129	3.190	25159	870019
2	28.871	96.810	538977	26404801
Totol:		100.000	564136	27274820

Supplementary Figure 234. HPLC data of **4k**

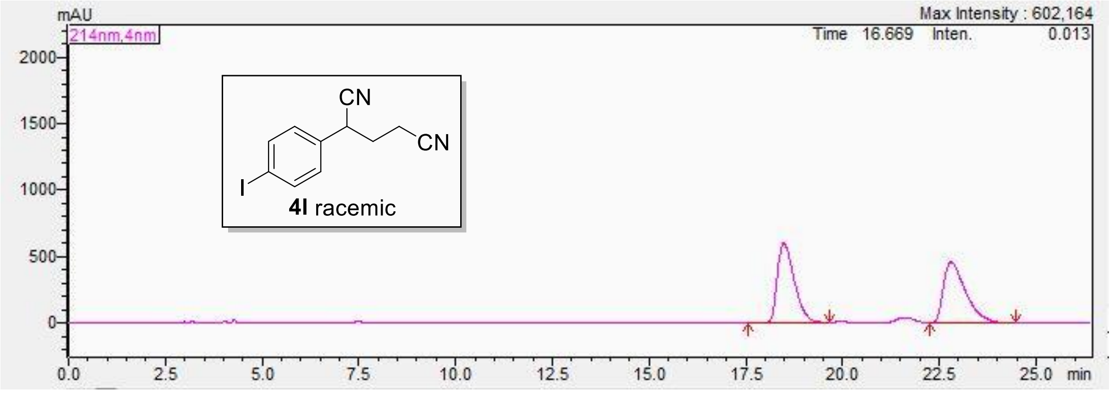

Peak	Ret. time	Area %	Height	Area
1	18.484	49.673	598553	18095779
2	22.806	50.327	457300	18333829
Totol:		100.000	1055853	36429608

Supplementary Figure 235. HPLC data of rac-**4l**

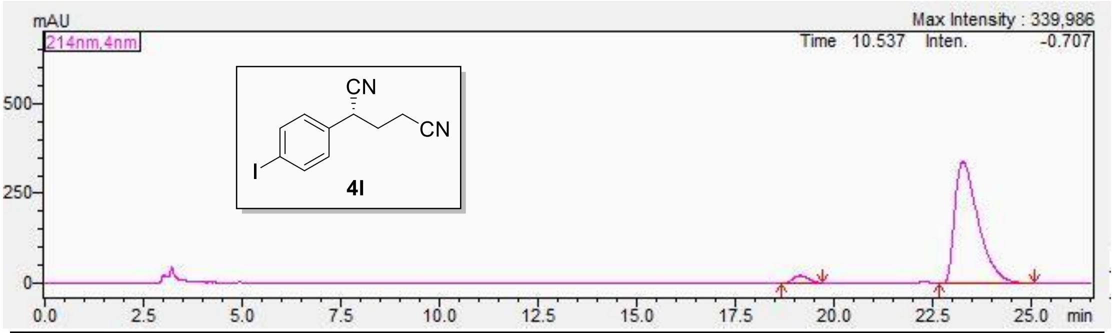

Peak	Ret. time	Area %	Height	Area
1	19.135	4.139	21286	603644
2	23.260	95.861	339858	13982000
Totol:		100.000	361144	14585644

Supplementary Figure 236. HPLC data of **4l**

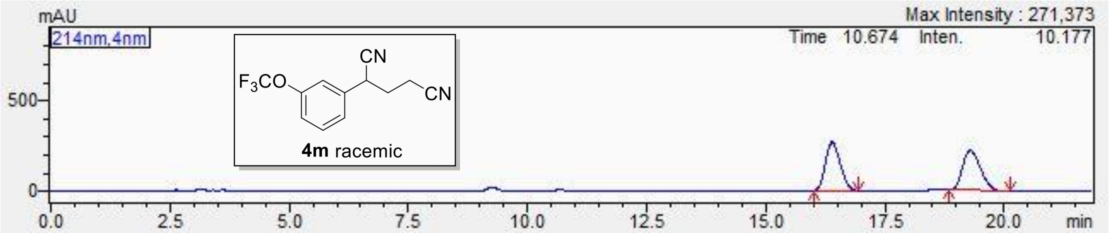

Peak	Ret. time	Area %	Height	Area
1	16.393	50.385	268765	5880478
2	19.292	49.615	221632	5790573
Totol:		100.000	490397	11671050

Supplementary Figure 237. HPLC data of rac-**4m**

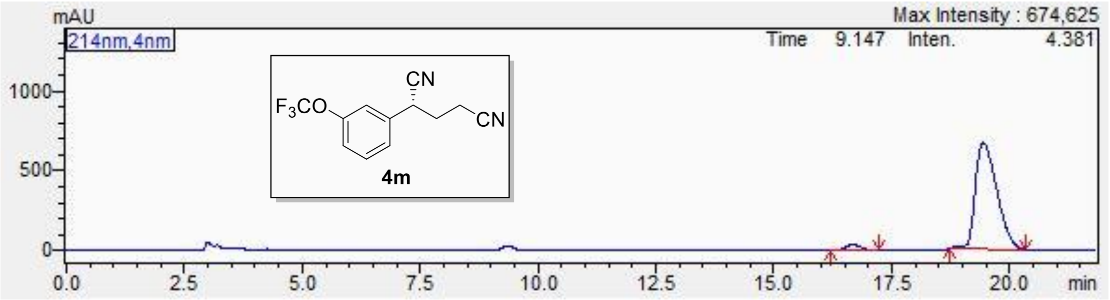

Peak	Ret. time	Area %	Height	Area
1	16.680	3.696	36611	820531
2	19.444	96.304	666264	21378195
Totol:		100.000	702875	22198725

Supplementary Figure 238. HPLC data of **4m**

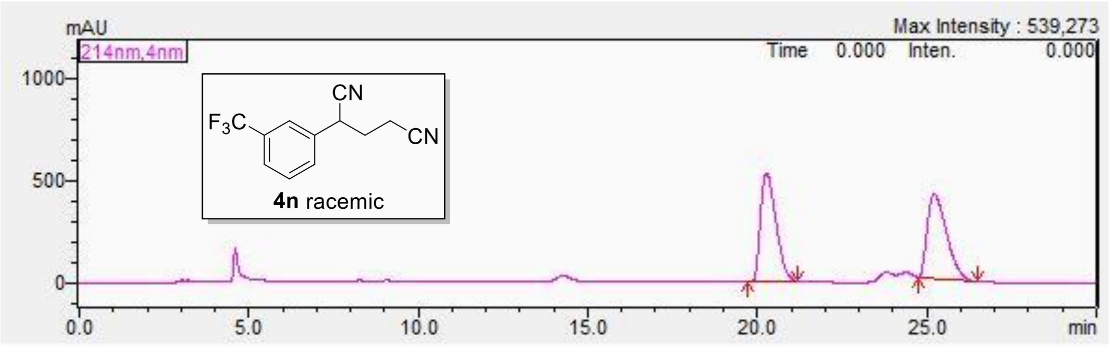

Peak	Ret. time	Area %	Height	Area
1	20.272	50.968	531718	16962366
2	25.226	49.032	414151	16317902
Totol:		100.000	945869	33280268

Supplementary Figure 239. HPLC data of rac-**4n**

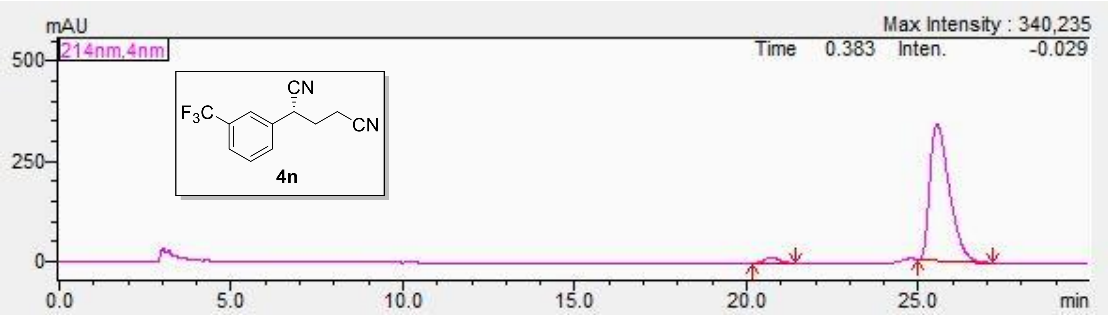

Peak	Ret. time	Area %	Height	Area
1	20.727	2.969	14274	408977
2	25.545	97.031	337800	13365589
Totol:		100.000	352074	13774566

Supplementary Figure 240. HPLC data of **4n**

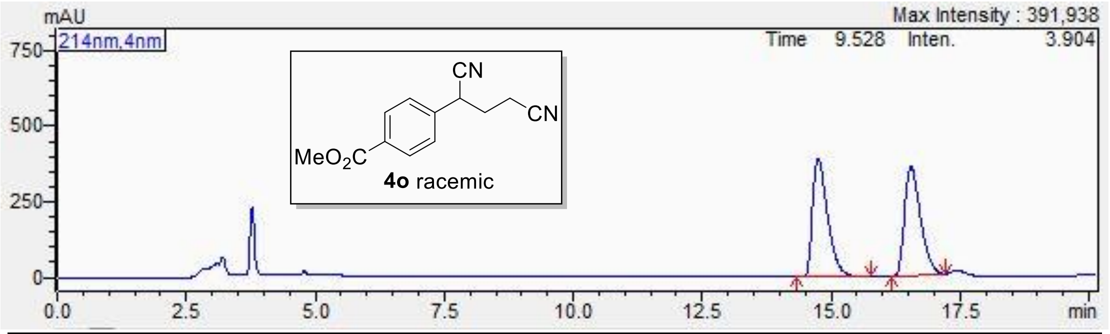

Peak	Ret. time	Area %	Height	Area
1	14.747	50.337	388466	7865032
2	16.547	49.663	358321	7759843
Totol:		100.000	746787	15624875

Supplementary Figure 241. HPLC data of rac-**4o**

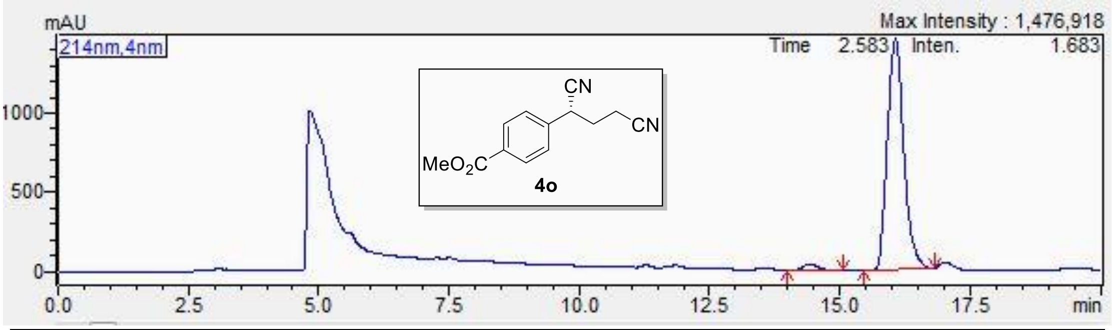

Peak	Ret. time	Area %	Height	Area
1	14.422	2.396	39990	782603
2	16.071	97.604	1464194	31876062
Totol:		100.000	1504185	32658665

Supplementary Figure 242. HPLC data of **4o**

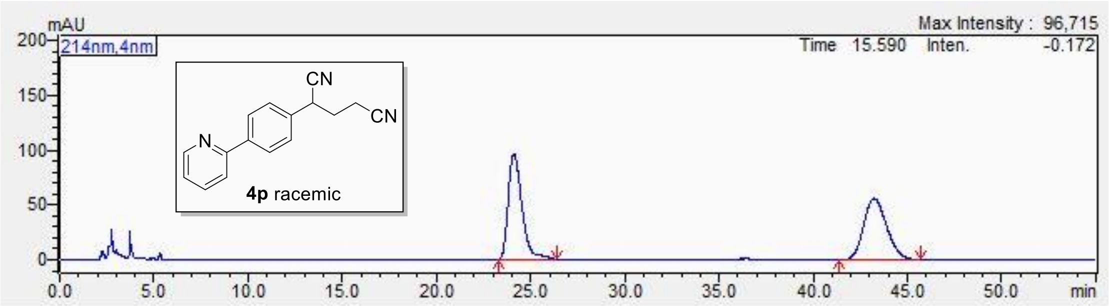

Peak	Ret. time	Area %	Height	Area
1	24.109	50.718	96590	4910369
2	43.228	49.282	55988	4771330
Totol:		100.000	152578	9681699

Supplementary Figure 243. HPLC data of rac-**4p**

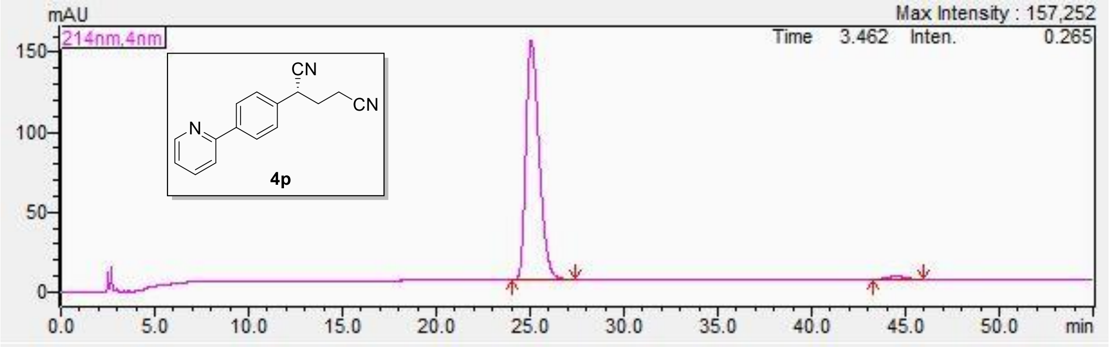

Peak	Ret. time	Area %	Height	Area
1	25.073	97.796	149895	7346504

2	44.488	2.204	2160	165578
Totol:		100.000	152055	7512081

Supplementary Figure 244. HPLC data of **4p**

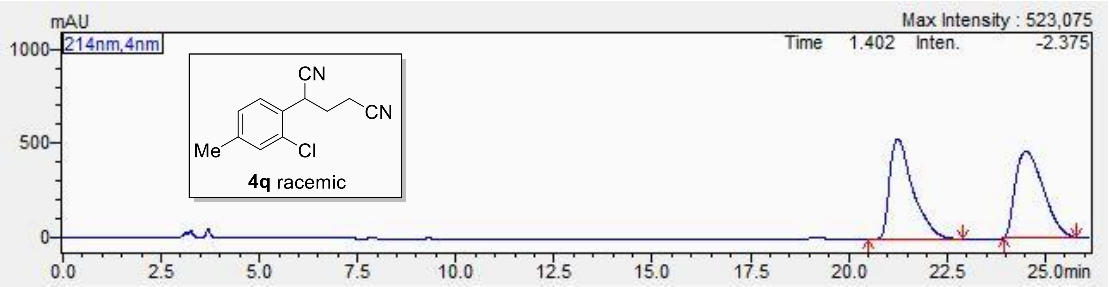

Peak	Ret. time	Area %	Height	Area
1	21.250	49.255	534048	22053017
2	24.510	50.745	458360	22720504
Totol:		100.000	992408	44773521

Supplementary Figure 245. HPLC data of rac-**4q**

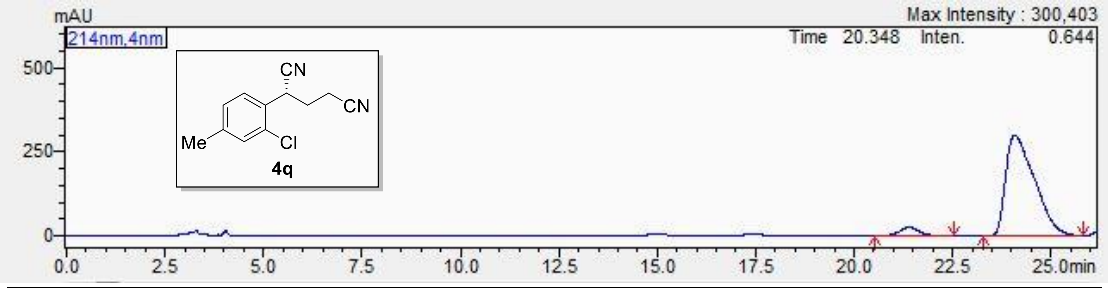

Peak	Ret. time	Area %	Height	Area
1	21.398	5.406	25431	869087
2	24.085	94.594	299843	15207757
Totol:		100.000	325274	16076844

Supplementary Figure 246. HPLC data of **4q**

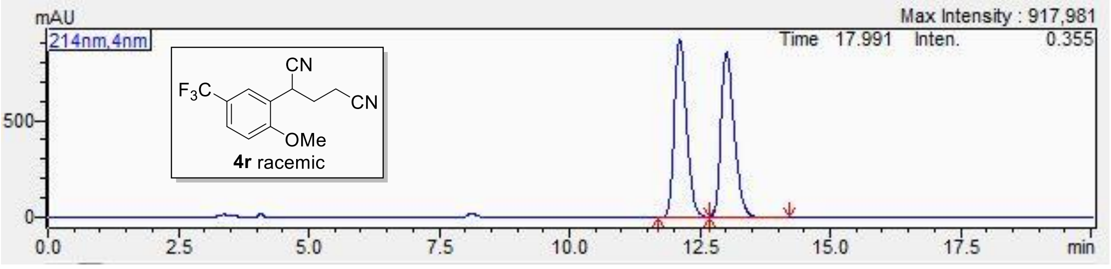

Peak	Ret. time	Area %	Height	Area
1	12.115	49.878	917715	14965355
2	13.012	50.122	849132	15038337
Totol:		100.000	1766846	30003692

Supplementary Figure 247. HPLC data of rac-**4r**

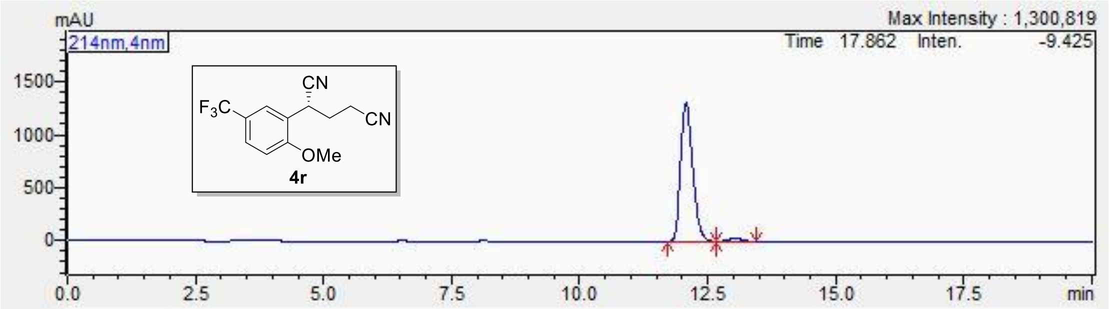

Peak	Ret. time	Area %	Height	Area
1	12.077	97.455	1308217	21516109
2	13.038	2.545	33887	561934
Totol:		100.000	1342104	22078043

Supplementary Figure 248. HPLC data of **4r**

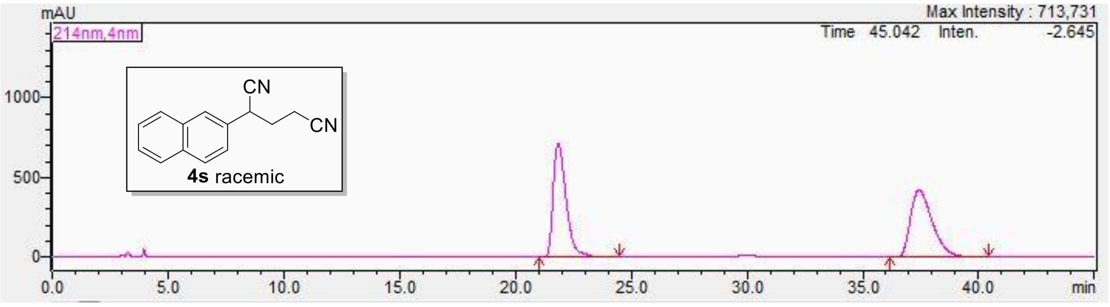

Peak	Ret. time	Area %	Height	Area
1	21.852	50.067	716411	27801563
2	37.433	49.933	421585	27727379
Totol:		100.000	1137996	55528942

Supplementary Figure 249. HPLC data of rac-**4s**

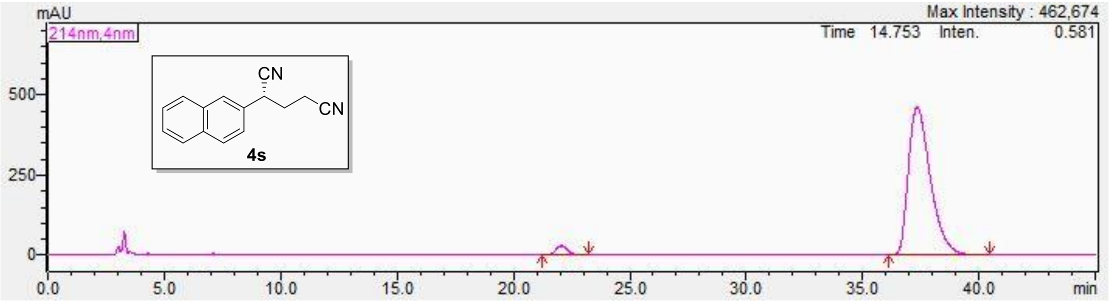

Peak	Ret. time	Area %	Height	Area
1	22.058	3.277	28647	1026689
2	37.341	96.723	461715	30301874

Totol:		100.000	490361	31328563

Supplementary Figure 250. HPLC data of **4s**

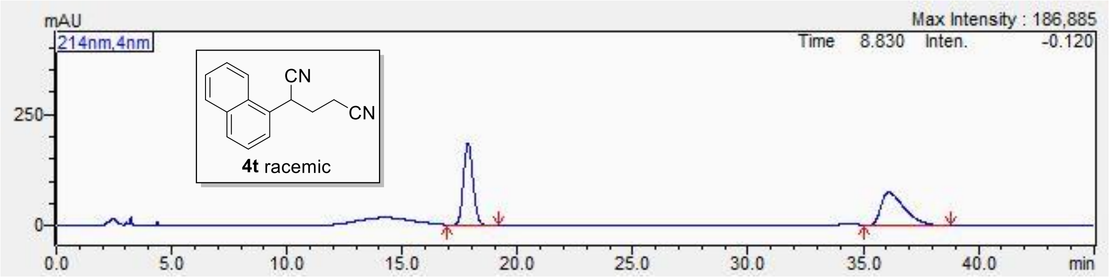

Peak	Ret. time	Area %	Height	Area
1	17.858	50.147	185718	5318123
2	36.123	49.853	74182	5286890
Totol:		100.000	259900	10605013

Supplementary Figure 251. HPLC data of rac-**4t**

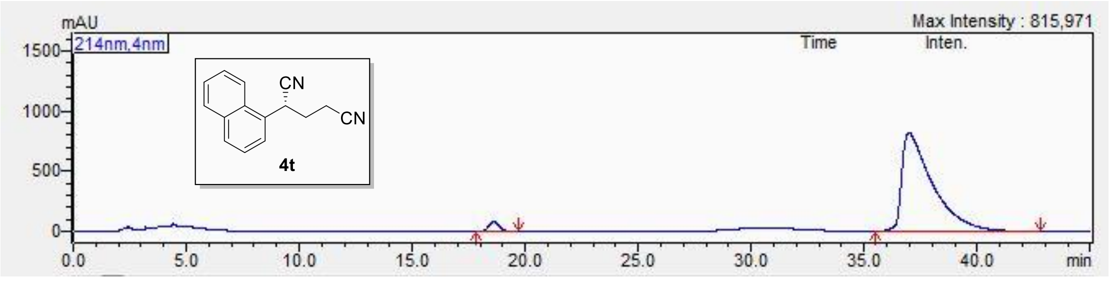

Peak	Ret. time	Area %	Height	Area
1	18.610	3.057	78011	2437679
2	36.986	96.943	816502	77292310
Totol:		100.000	894513	79729990

Supplementary Figure 252. HPLC data of **4t**

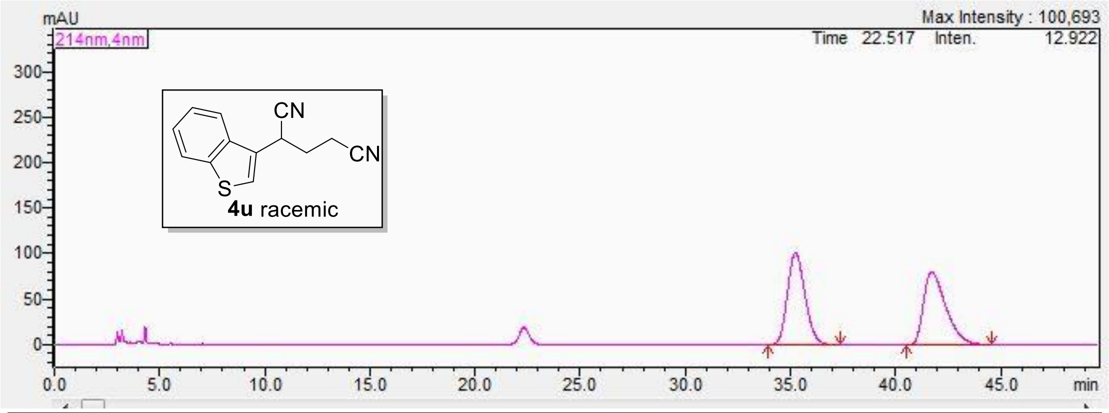

Peak	Ret. time	Area %	Height	Area
1	35.244	49.950	101262	5907657

2	41.727	50.050	79936	5919500
Totol:		100.000	181199	11827157

Supplementary Figure 253. HPLC data of rac-**4u**

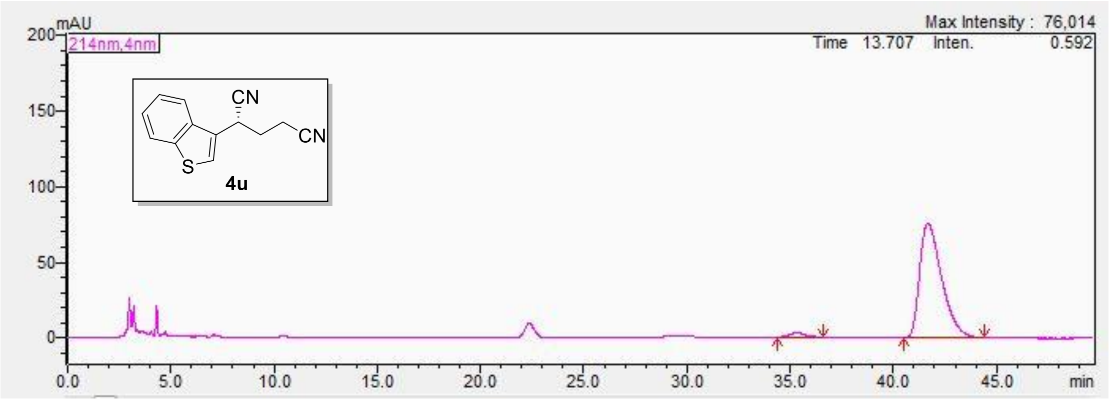

Peak	Ret. time	Area %	Height	Area
1	35.346	2.616	2676	150677
2	41.678	97.384	75912	5609811
Totol:		100.000	78588	5760488

Supplementary Figure 254. HPLC data of **4u**

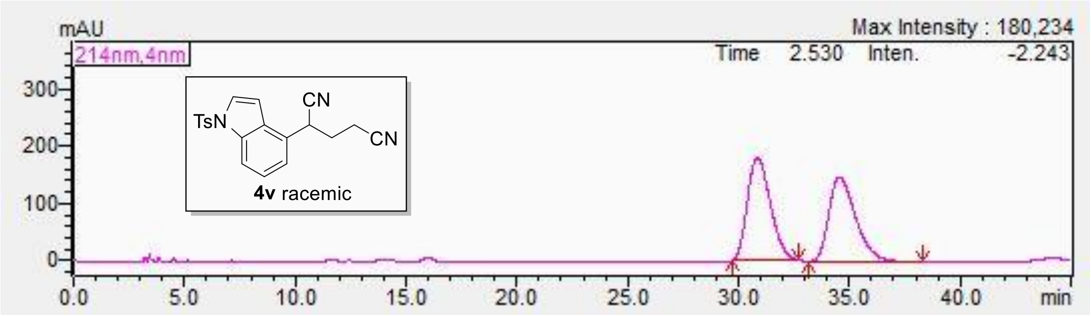

Peak	Ret. time	Area %	Height	Area
1	30.858	50.997	178733	12868867
2	34.569	49.003	147287	12365858
Totol:		100.000	326020	25234724

Supplementary Figure 255. HPLC data of rac-**4v**

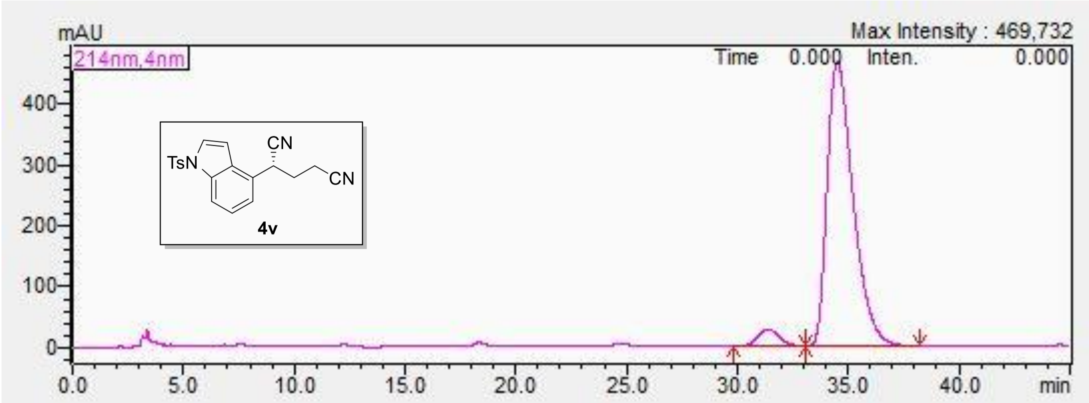

Peak	Ret. time	Area %	Height	Area
1	31.388	4.920	28587	1971149
2	34.516	95.080	467567	38090871
Totol:		100.000	496154	40062019

Supplementary Figure 256. HPLC data of **4v**

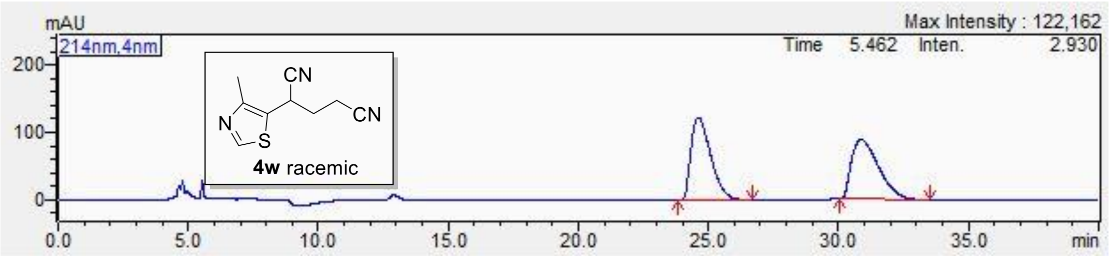

Peak	Ret. time	Area %	Height	Area
1	24.615	50.297	123269	6537809
2	30.879	49.703	88748	6460641
Totol:		100.000	212017	12998450

Supplementary Figure 257. HPLC data of rac-**4w**

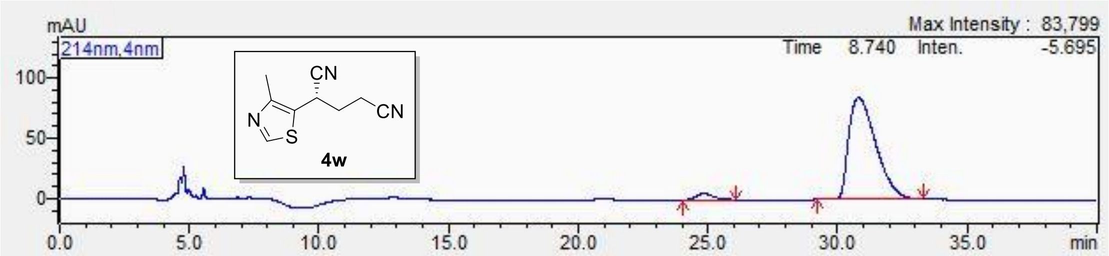

Peak	Ret. time	Area %	Height	Area
1	24.838	3.715	5158	239019
2	30.815	96.285	84569	6194066
Totol:		100.000	89728	6433085

Supplementary Figure 258. HPLC data of **4w**

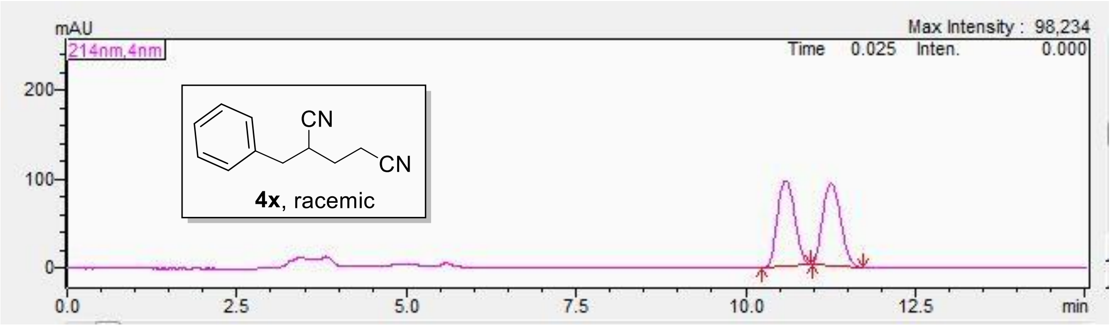

Peak	Ret. time	Area %	Height	Area
1	10.590	50.235	96031	1718263
2	11.259	49.765	92077	1702164
Totol:		100.000	188108	3420428

Supplementary Figure 259. HPLC data of rac-**4x**

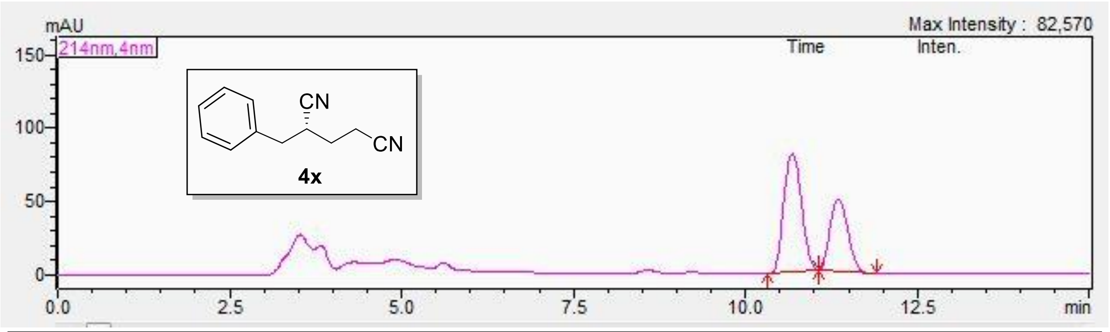

Peak	Ret. time	Area %	Height	Area
1	10.679	61.654	80437	1431250
2	11.350	38.346	48957	890167
Totol:		100.000	129394	2321417

Supplementary Figure 260. HPLC data of **4x**

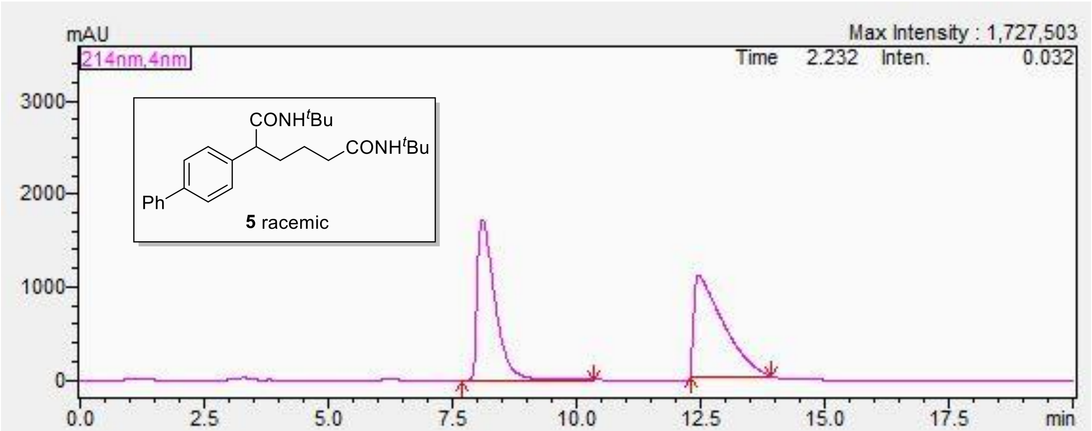

Peak	Ret. time	Area %	Height	Area
1	8.110	49.364	1730362	43389738

2	12.472	50.636	1080572	44508003
Totol:		100.000	2810934	87897742

Supplementary Figure 261. HPLC data of rac-**5**

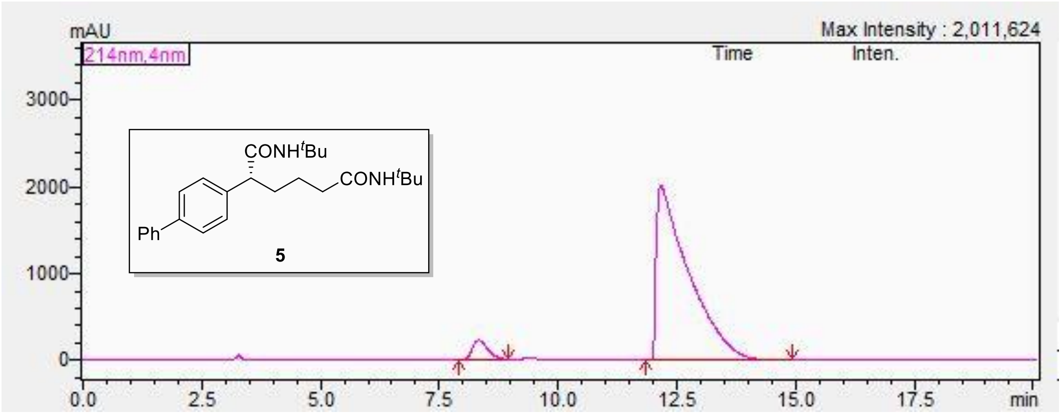

Peak	Ret. time	Area %	Height	Area
1	8.334	5.139	226876	5191848
2	12.164	94.861	2009410	95833648
Totol:		100.000	2236286	101025496

Supplementary Figure 262. HPLC data of **5**

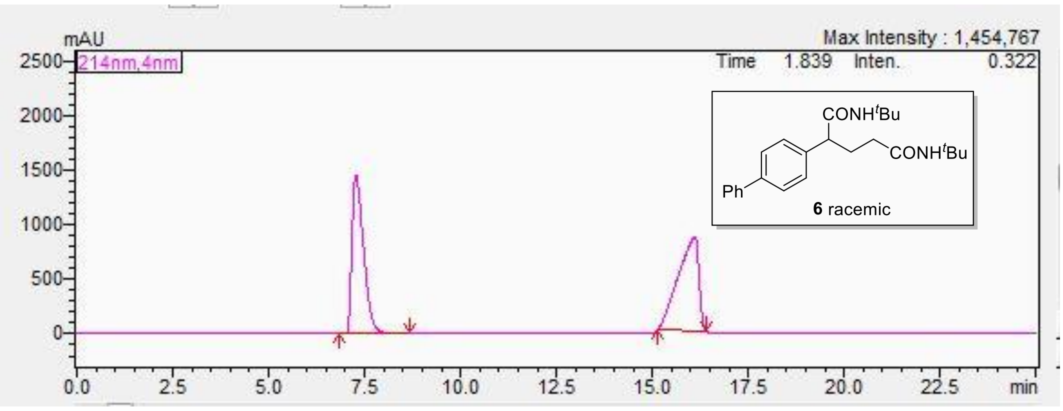

Peak	Ret. time	Area %	Height	Area
1	7.276	49.208	1454214	31065110
2	16.101	50.792	861532	32065715
Totol:		100.000	2315746	63130826

Supplementary Figure 263. HPLC data of rac-**6**

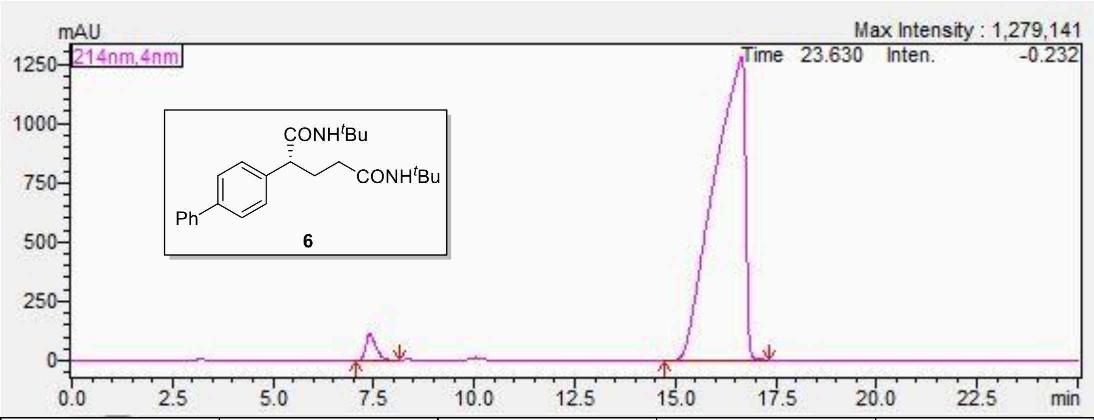

Peak	Ret. time	Area %	Height	Area
1	7.406	2.778	110652	1980064
2	16.650	97.222	1276891	69301930
Totol:		100.000	1387543	71281994

Supplementary Figure 264. HPLC data of **6**

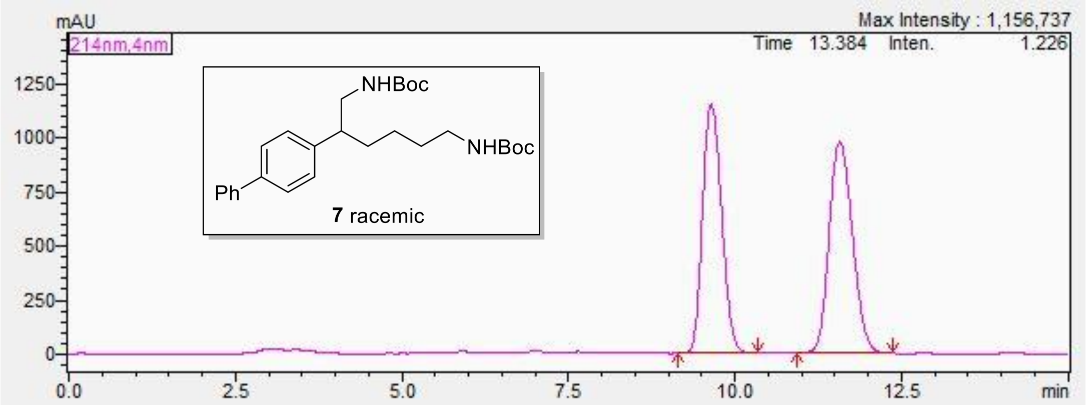

Peak	Ret. time	Area %	Height	Area
1	9.646	49.131	1151032	22990013
2	11.576	50.869	975037	23803264
Totol:		100.000	2126070	46793277

Supplementary Figure 265. HPLC data of rac-**7**

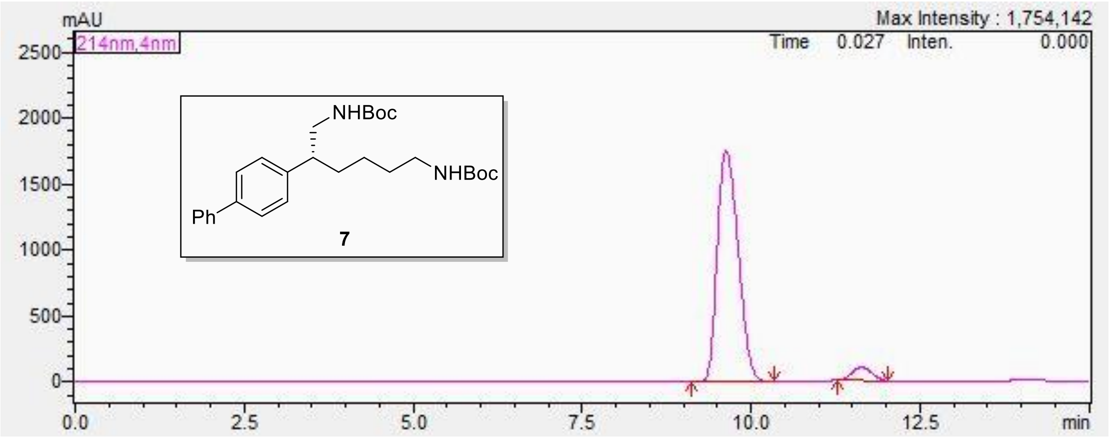

Peak	Ret. time	Area %	Height	Area
1	9.629	94.779	1751637	37675479
2	11.635	5.221	100773	2075260
Totol:		100.000	1852410	39750738

Supplementary Figure 266. HPLC data of **7**

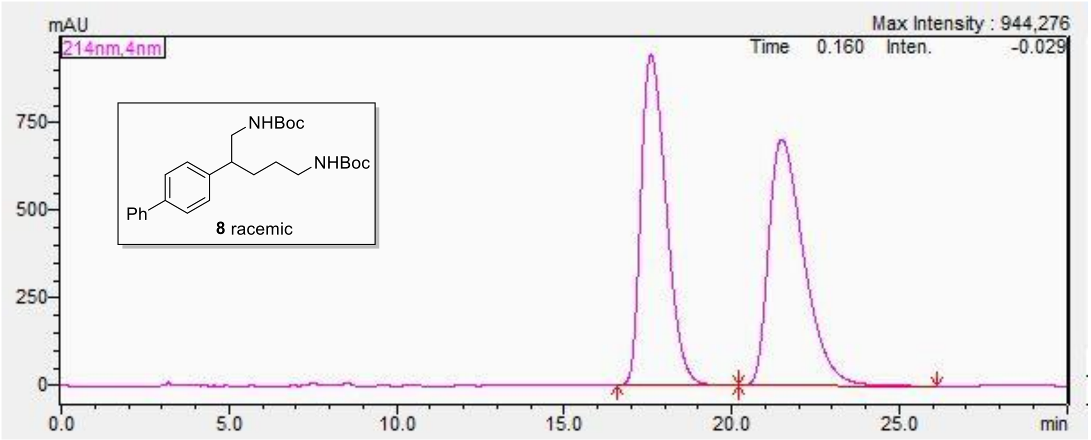

Peak	Ret. time	Area %	Height	Area
1	17.592	49.058	943880	49556085
2	21.489	50.942	703573	51459994
Totol:		100.000	1647453	101016079

Supplementary Figure 267. HPLC data of rac-**8**

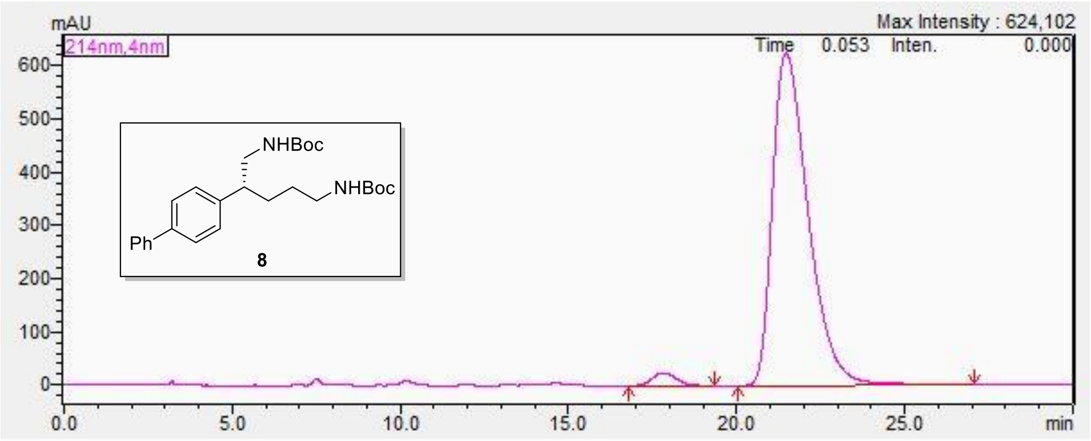

Peak	Ret. time	Area %	Height	Area
1	17.833	2.526	24174	1200380
2	21.480	97.474	625083	46315576
Totol:		100.000	649257	47515956

Supplementary Figure 268. HPLC data of **8**

Supplementary Tables

Supplementary Table 1 Optimization of Reaction Conditions

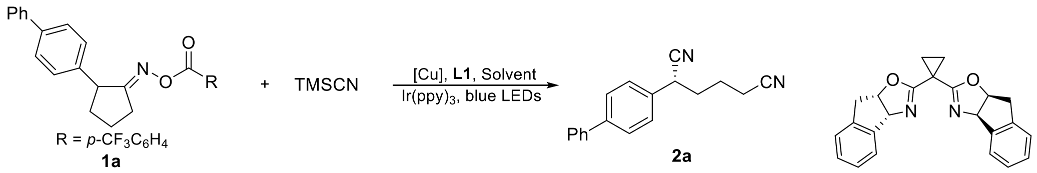

L1

Entry	Cu	$Ir(ppy)_3$	TMSCN	Solvent	Yield %[a]	ee %[b]
1	10 mol %	1 mol %	1.5 eq.	DMF/DCM	38	87
2	5 mol %	1 mol %	1.5 eq.	DMF/DCM	48	87
3	5 mol %	0.5 mol %	1.5 eq.	DMF/DCM	50	89
4	5 mol %	1 mol %	3 eq.	DMF/DCM	55	88
5	5 mol %	1 mol %	1.5 eq.	DCM	49	88
6	5 mol %	0.5 mol %	3 eq.	DCM	53	90
7	3 mol %	0.5 mol %	3 eq.	DCM	62	90
8	2 mol %	0.5 mol %	3 eq.	DCM	72	90
9	1 mol %	0.5 mol %	3 eq.	DCM	-[c]	-

Conditions: x mol % $Cu(CH_3CN)_4PF_6$, 1.2x mol % **L1**, y mol% $Ir(ppy)_3$, 1.5 or 3.0 eq. TMSCN, 1.0 mL solvent was used for 0.1 mmol scale reaction, 24 W blue LEDs, RT, 24 h, 4/6 DMF/DCM.
[a]isolated yield. [b]detected by HPLC. [c]low conversion.

Supplementary Table 2 Optimization of Reaction Conditions

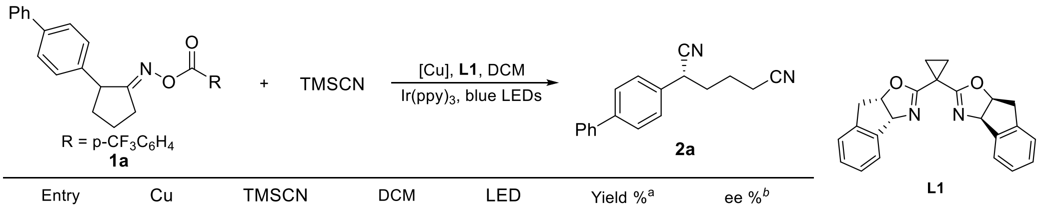

Entry	Cu	TMSCN	DCM	LED	Yield %[a]	ee %[b]
1	2 mol %	3 eq.	1 mL	24 W	69	90
2	2 mol %	2 eq.	1 mL	24 W	68	90
3	2 mol %	2 eq.	0.5 mL	24 W	50	90
4	2 mol %	2 eq.	2 mL	24 W	-[c]	-
5	1.8 mol %	3 eq.	1 mL	24 W	53	90
6	1.5 mol %	3 eq.	1 mL	24 W	62	89
7	2 mol %	3 eq.	1 mL	5 W	71	90.6

Conditions: x mol % $Cu(CH_3CN)_4PF_6$, 1.5x mol % **L1**, 0.5 mol% $Ir(ppy)_3$, y eq. TMSCN was used for 0.1 mmol scale reaction, 24 or 5 W blue LEDs. RT. 36 h.
[a]isolated yield. [b]detected by HPLC. [c]low conversion.

Supplementary Table 3 Screening of Copper Catalyst

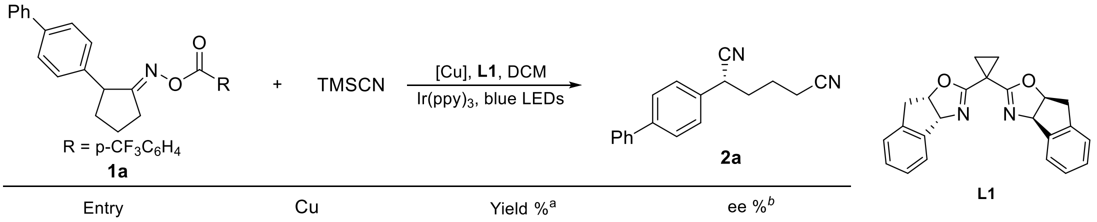

Entry	Cu	Yield %[a]	ee %[b]
1	CuBr	48	90
2	CuCN	42	90.6
3	CuOAc	51	91
4	$Cu(CH_3CN)_4BF_4$	64	91
5	CuCl	43	91
6	CuSCN	49	90.6
7	$Cu(CH_3CN)_4PF_6$	71	91

Conditions: 2 mol % [Cu], 3 mol % **L1**, 0.5 mol% $Ir(ppy)_3$, 3 eq. TMSCN, 1 mL DCM was used for 0.1 mmol scale reaction, 5 W blue LEDs, RT, 36 h.
[a]isolated yield.[b]detected by HPLC.

Supplementary Table 4 Screening of Temperature

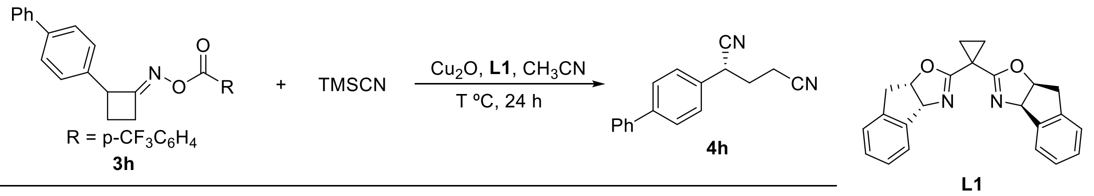

Entry	[Cu]	T/ºC	Yield %[a]	ee %[b]
1	Cu_2O	-10 ºC	47	94
2	Cu_2O	0 ºC	68	94
3	Cu_2O	10 ºC	91	94
4	Cu_2O	25 ºC	98	92.5

Conditions: 1 mol% Cu_2O, 4 mol % **L1**, 1.1 eq. TMSCN, 1.0 mL CH_3CN was used for 0.1 mmol scale reaction, T ºC, 24h.
[a]isolated yield.[b]detected by HPLC.

Supplementary Table 5 Screening of Copper Catalyst

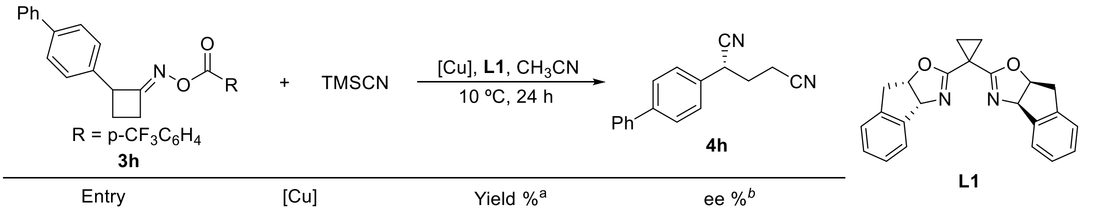

Entry	[Cu]	Yield %[a]	ee %[b]
1	CuCl	88	94
2	CuBr	78	94
3	CuI	91	94
4	CuSCN	97	94
5	CuCN	88	94
6	$Cu(acac)_2$	81	94
7	$Cu(CH_3CN)_4PF_6$	trace	-
8	CuF_2	76	94
9	$Cu(OAc)_2$	74	94

Conditions: 2 mol % [Cu], 4 mol % **L1**, 1.1 eq. TMSCN, 1.0 mL CH_3CN was used for 0.1 mmol scale reaction, 10 ºC, 24h .
[a]isolated yield.[b]detected by HPLC.

Supplementary Table 6 Screening of Solvent

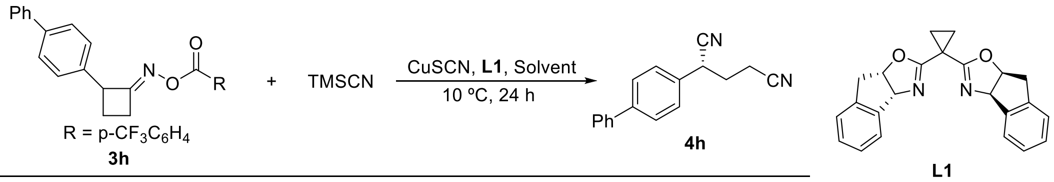

Entry	[Cu]	solvent	Yield %[a]	ee %[b]
1	CuSCN	DCM	72	94
2	CuSCN	Et_2O	56	93
3	CuSCN	DMF	71	93
4	CuSCN	toluene	78	94
5	CuSCN	MeOH	75	89
6	CuSCN	Acetone	70	95
7[c]	CuSCN(3 mol %)	Acetone	93	95

Conditions: 2 mol % CuSCN, 4 mol % **L1**, 1.1 eq. TMSCN, 1.0 mL Solvent was used for 0.1 mmol scale reaction, 10 °C, 24h.
[a]isolated yield.[b]detected by HPLC. [c]3 mol % Cu, 3.6 mol % **L1**, 1.5 eq. TMSCN, 1.0 mL degassed Acetone.

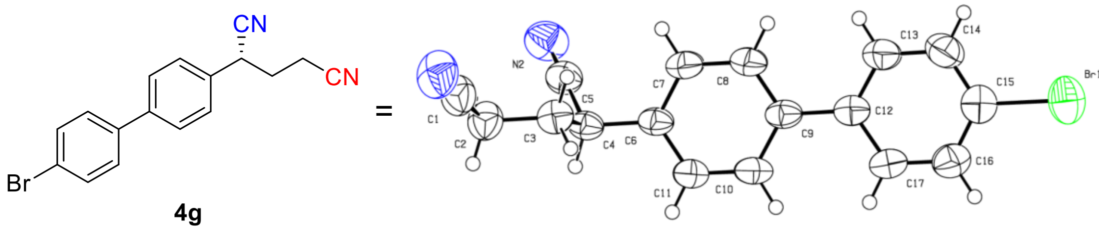

Supplementary Figure 269. X-ray crystallography of **4g**

Supplementary Table 7 Crystal data and structure refinement for **4g**.

Identification code	4g
Empirical formula	$C_{17}H_{13}BrN_2$
Formula weight	325.20
Temperature/K	293(2)
Crystal system	monoclinic
Space group	$P2_1$
a/Å	7.98210(10)
b/Å	5.77100(10)
c/Å	16.0567(3)
α/°	90

β/°	93.240(2)
γ/°	90
Volume/Å^3	738.46(2)
Z	2
ρ_{calc}g/cm^3	1.463
μ/mm^{-1}	3.706
F(000)	328.0
Crystal size/mm^3	0.3 × 0.22 × 0.2
Radiation	CuKα (λ = 1.54184)
2Θ range for data collection/°	11.038 to 147.586
Index ranges	-9 ≤ h ≤ 8, -6 ≤ k ≤ 6, -19 ≤ l ≤ 19
Reflections collected	6082
Independent reflections	2726 [R_{int} = 0.0195, R_{sigma} = 0.0166]
Data/restraints/parameters	2726/1/181
Goodness-of-fit on F^2	1.367
Final R indexes [I>=2σ (I)]	R_1 = 0.0402, wR_2 = 0.1493
Final R indexes [all data]	R_1 = 0.0405, wR_2 = 0.1504
Largest diff. peak/hole / e Å^{-3}	0.31/-0.61
Flack parameter	0.024(15)

Supplementary Table 8 Fractional Atomic Coordinates (×10^4) and Equivalent Isotropic Displacement Parameters (Å^2×10^3) for **4g**. U_{eq} is defined as 1/3 of of the trace of the orthogonalised U_{IJ} tensor.

Atom	*x*	*y*	*z*	U(eq)
Br1	240.9(6)	3265.2(16)	2337.5(3)	81.7(3)
C12	2534(4)	3632(8)	5089(3)	49.0(9)
C5	5078(6)	6564(9)	8880(3)	58.4(10)
N2	5053(6)	8376(11)	9137(3)	80.9(12)
C9	3206(4)	3779(7)	5975(3)	50.0(9)
C4	5164(4)	4181(7)	8549(3)	49.4(8)
C3	7014(5)	3332(11)	8559(3)	56.3(8)
C15	1195(5)	3403(11)	3455(3)	61.0(10)
C6	4420(5)	4085(7)	7650(2)	48.8(9)
C2	7864(5)	3338(15)	9429(3)	67.0(11)
N1	11113(5)	3097(17)	9336(4)	93.6(16)
C14	2200(8)	5243(11)	3707(3)	74.4(13)
C1	9689(6)	3173(14)	9380(3)	71.3(11)
C17	1537(6)	1768(9)	4803(3)	64.0(11)
C13	2845(7)	5337(9)	4515(3)	66.8(12)

C11	3454(7)	2255(10)	7381(4)	71.2(14)
C16	882(6)	1642(10)	3992(4)	67.1(12)
C10	2877(8)	2065(11)	6556(4)	77.5(16)
C8	4164(8)	5629(10)	6251(3)	71.7(14)
C7	4774(8)	5793(10)	7085(4)	73.3(14)

Supplementary Table 9 Anisotropic Displacement Parameters ($Å^2 \times 10^3$) for **4g**. The Anisotropic displacement factor exponent takes the form: $-2\pi^2[h^2a^{*2}U_{11}+2hka^*b^*U_{12}+\ldots]$.

Atom	U_{11}	U_{22}	U_{33}	U_{23}	U_{13}	U_{12}
Br1	81.4(4)	98.9(5)	64.5(4)	-9.1(3)	2.6(2)	20.4(3)
C12	40.4(15)	45(2)	63(2)	0.6(16)	12.6(14)	2.5(13)
C5	55(2)	57(3)	64(2)	3.8(19)	9.0(17)	0.7(17)
N2	99(3)	61(3)	85(3)	-5(3)	16(2)	10(2)
C9	37.7(14)	43(2)	70(2)	4.8(15)	9.5(14)	-0.8(12)
C4	42.0(16)	46(2)	61(2)	7.6(15)	9.8(14)	-0.9(13)
C3	47.3(16)	56(2)	66(2)	-1(2)	8.4(14)	3.0(19)
C15	54.5(18)	68(3)	61(2)	-5(2)	9.5(15)	8(2)
C6	40.2(13)	45(2)	62(2)	7.2(15)	10.1(13)	-3.3(13)
C2	56(2)	78(3)	67(2)	-1(3)	-0.6(17)	-1(2)
N1	54(2)	124(5)	101(3)	4(4)	-6(2)	4(3)
C14	101(4)	63(3)	60(3)	9(2)	11(2)	-7(3)
C1	60(2)	82(3)	70(2)	3(3)	-7.7(18)	3(3)
C17	67(3)	52(3)	73(3)	3.6(19)	4(2)	-16.2(19)
C13	80(3)	50(3)	71(3)	5(2)	6(2)	-10.9(19)
C11	77(3)	59(3)	76(3)	21(2)	-13(2)	-23(2)
C16	63(2)	65(3)	74(3)	-1(2)	8(2)	-10(2)
C10	87(3)	56(3)	88(4)	24(3)	-12(3)	-29(3)
C8	86(3)	66(3)	63(3)	14(2)	6(2)	-31(3)
C7	90(3)	62(3)	67(3)	7(2)	5(2)	-36(3)

Supplementary Table 10 Bond Lengths for **4g**

Atom	Atom	Length/Å	Atom	Atom	Length/Å
Br1	C15	1.911(4)	C15	C14	1.377(8)
C12	C9	1.494(6)	C15	C16	1.364(8)
C12	C17	1.400(6)	C6	C11	1.364(6)
C12	C13	1.381(7)	C6	C7	1.380(6)
C5	N2	1.124(9)	C2	C1	1.466(6)
C5	C4	1.478(7)	N1	C1	1.143(7)
C9	C10	1.395(6)	C14	C13	1.370(8)

C9	C8	1.372(7)	C17	C16	1.378(8)
C4	C3	1.555(5)	C11	C10	1.381(8)
C4	C6	1.530(6)	C8	C7	1.401(8)
C3	C2	1.519(6)			

Supplementary Table 11 Bond Angles for **4g**

**Atom**	**Atom**	**Atom**	**Angle/°**	**Atom**	**Atom**	**Atom**	**Angle/°**
C17	C12	C9	121.5(4)	C11	C6	C4	120.6(4)
C13	C12	C9	121.8(4)	C11	C6	C7	118.5(4)
C13	C12	C17	116.7(4)	C7	C6	C4	120.8(4)
N2	C5	C4	178.4(5)	C1	C2	C3	110.1(4)
C10	C9	C12	121.7(4)	C13	C14	C15	119.3(5)
C8	C9	C12	121.0(4)	N1	C1	C2	178.4(9)
C8	C9	C10	117.3(5)	C16	C17	C12	121.9(5)
C5	C4	C3	110.7(4)	C14	C13	C12	122.2(5)
C5	C4	C6	110.5(4)	C6	C11	C10	121.2(4)
C6	C4	C3	108.5(3)	C15	C16	C17	119.0(5)
C2	C3	C4	112.6(4)	C11	C10	C9	121.3(5)
C14	C15	Br1	120.1(4)	C9	C8	C7	121.2(4)
C16	C15	Br1	119.0(4)	C6	C7	C8	120.5(4)
C16	C15	C14	120.9(4)				

Supplementary Table 12 Hydrogen Atom Coordinates (Å×10^4) and Isotropic Displacement Parameters (Å^2×10^3) for **4g**.

**Atom**	***x***	***y***	***z***	**U(eq)**
H4	4524.68	3144.47	8895.43	59
H3A	7038.37	1772.68	8334.28	68
H3B	7638.71	4325.65	8200.46	68
H2A	7584.47	4752.89	9716.14	80
H2B	7463.42	2037.29	9745.02	80
H14	2438.98	6408.79	3331.45	89
H17	1309.55	580.01	5171.73	77
H13	3514.81	6590.71	4682.02	80
H11	3179.1	1115.15	7758.92	85
H16	236.47	376.94	3812.85	80
H10	2257.31	768.22	6385.18	93
H8	4412.29	6796.1	5878.29	86
H7	5422.63	7061.83	7257.88	88

Supplementary Methods

General Information ^{1}H NMR spectra were recorded at ambient temperature on Bruker-400 (400 MHz) spectrometers and are referenced relative to the residual protons in $CDCl_3$ at δ 7.26 ppm or $(CD_3)_2SO\text{-}d_6$ at δ 2.50 ppm. Data for ^{1}H NMR are reported as follows: chemical shift (ppm), multiplicity (s = singlet, d = doublet, t = triplet, q = quartet, m = multiplet, ap = apparent), integration, and coupling constant (Hz). ^{13}C NMR spectra were recorded at ambient temperature on Bruker-400 (100 MHz) spectrometers and are referenced relative to $CDCl_3$ at δ 77.16 ppm or $(CD_3)_2SO\text{-}d_6$ at δ 39.25 ppm. The ^{13}C NMR spectra were obtained with 1H decoupling. Data for ^{13}C NMR are reported in terms of chemical shift and multiplicity where appropriate. High resolution mass spectra were recorded on P-SIMS-Gly of Bruker Daltonics Inc. using ESI-TOF (electrospray ionization-time of flight). High performance liquid chromatography was performed on shimadzu Series HPLC, using AD-H, OD-H, ID chiral column eluted with a mixture of hexane and isopropyl alcohol. TMSCN was purchased from energy-chemical, $Cu(CH_3CN)_4PF_6$ and CuSCN was purchased from TCI. $Ir(ppy)_3$ was purchased from Laajoo Reagent. DMAc was purchased from J&K Chemical Reagent. And acetone was purchased from Sinopharm Chemical Reagent Co. Ltd.

Genaral procedure (A) for synthesis of the substrates 1[1,2,3].

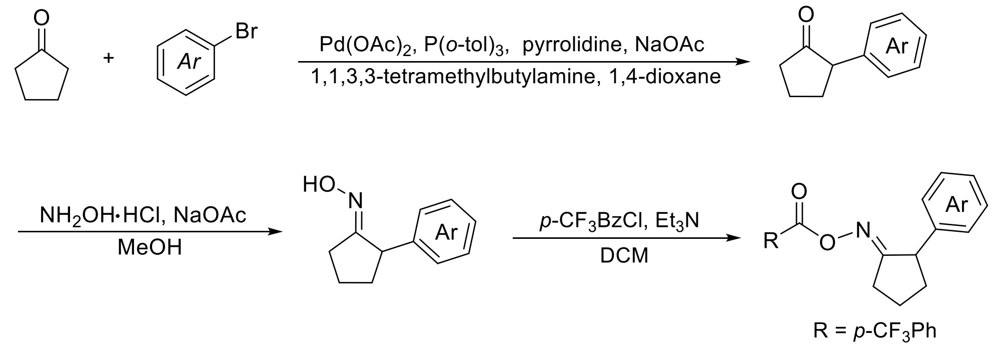

1

The 2-arylcyclopentan-1-ones were synthesized according to the reported procedure[3]. An 100 mL Schlenk flask was charged with $Pd(OAc)_2$ (67.6 mg, 0.3 mmol, 0.05 equiv), P(*o*-tol)$_3$ (182.8 mg, 0.6 mmol, 0.1 equiv), NaOAc (492.4 mg, 6.0 mmol, 1.0 equiv) and aryl bromides (7.8 mmol, 1.3 equiv), and then the flask was evacuated and backfilled with N_2 (repeated for 3 times). 1,4-dioxane (30.0 mL) was added in, followed by cyclopentanone (531 uL, 6.0 mmol, 1.0 equiv), pyrrolidine (148 μL, 1.8 mmol, 0.3 equiv) and 1,1,3,3-tetramethylbutylamine (289 uL, 1.8 mmol, 0.3 equiv). The flask was then heated in an oil bath at 110 °C under stirring for 16 hours, before cooled to room temperature. The mixture was filtered through a small plug of silica gel, eluted with ethyl acetate. The solvent was removed in vacuo and flash column chromatography of the residue gave the arylation product.

The ketones (1.0 equiv, ~5 mmol), hydroxylamine hydrochloride (1.2 equiv) and sodium acetate (1.5 equiv) were added to a round bottom flask containing a stirrer bar. MeOH (0.56 M) was added and the reaction was stirred for 12 h at 75 ºC. Then methanol was removed under vacuum and the resulting mixture was extracted with DCM. The organic layer was washed with water and dried over Na_2SO_4. The solvent was removed under reduced pressure and the crude material was subjected to column chromatography to afford oximes.

To a mixture of oxime (1.0 equiv, ~4 mmol), triethylamine (1.5 equiv) and DCM (0.5 M) in a flask was added 4-(trifluoromethyl)benzoyl chlorides (1.1 equiv) slowly at 0 °C. After 1 h, a saturated solution of aqueous $NaHCO_3$ (10 mL) was added to the above

solution, and the mixture was diluted with DCM. The organic layer was washed with brine (20 mL) and dried over Na_2SO_4. The solvent was removed under vacuum and the residue was subjected to column chromatography on silica gel with EtOAc–petroleum ether as an eluent to give the substrate.

Characterization data for cyclopentanone oxime esters 1

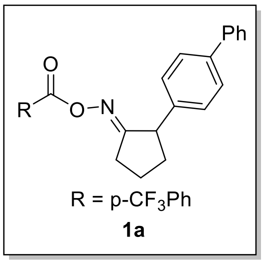

Prepared according to general procedure (A) from cyclopentanone and 4-bromo-1,1'-biphenyl to provide the title compound **1a** as a yellowish solid[1]. ^{1}H NMR ($CDCl_3$, 400 MHz) δ 8.19 (d, *J* = 8.2 Hz, 2H), 7.74 (d, *J* = 8.4 Hz, 2H), 7.64 – 7.54 (m, 4H), 7.48 – 7.38 (m, 4H), 7.34 (t, *J* = 7.4 Hz, 1H), 4.11 (t, *J* = 7.6 Hz, 1H), 3.01 – 2.94 (m, 1H), 2.88 – 2.81 (m, 1H), 2.41 – 2.34 (m, 1H), 2.14 – 2.03 (m, 2H), 1.92 – 1.87 (m, 1H); ^{13}C NMR($CDCl_3$, 101 MHz) δ 178.2, 162.6, 140.8, 139.8,139.1, 134.6 (q, *J* = 32.8 Hz), 132.5, 129.9, 128.7, 128.3, 127.4, 127.2, 127.0, 125.5 (q, *J* = 3.6 Hz), 123.5 (q, *J* = 272.8 Hz), 49.0, 34.7, 30.0, 22.5.

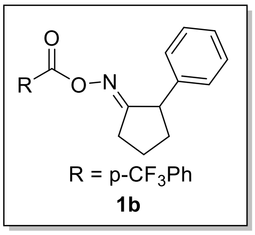

Prepared according to general procedure (A) from cyclopentanone and bromobenzene to provide the title compound **1b** as a yellowish solid[1]. ^{1}H NMR (400 MHz, $CDCl_3$) 8.17 (d, *J* = 8.2 Hz, 2H), 7.73 (d, *J* = 8.3 Hz, 2H), 7.34 (d, *J* = 6.6 Hz, 4H), 7.26 – 7.21 (m, 1H), 4.07 (t, *J* = 7.2 Hz, 1H), 3.01 – 2.74 (m, 2H), 2.38 – 2.29 (m, 1H), 2.13 – 1.94 (m, 2H), 1.93 – 1.79 (m, 1H); ^{13}C NMR (101 MHz, $CDCl_3$) δ 178.2, 162.6, 140.1, 134.6 (q, *J* = 32.7 Hz), 132.5, 129.9, 128.6, 127.8, 126.9, 125.5 (q, *J* = 3.5 Hz), 123.5 (q, *J* = 272.8 Hz), 49.2, 34.7, 30.0, 22.5.

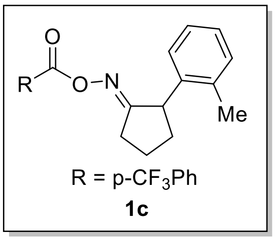

Prepared according to general procedure (A) from cyclopentanone and 1-bromo-2-methylbenzene to provide the title compound **1c** as a yellowish solid[1]. ^{1}H NMR (400 MHz, $CDCl_3$) δ 8.18 (d, *J* = 8.1 Hz, 2H), 7.73 (d, *J* = 8.3 Hz, 2H), 7.21 – 7.08 (m, 4H), 4.22 (t, *J* = 7.3 Hz, 1H), 3.02 – 2.83 (m, 2H), 2.40 (s, 3H), 2.36 – 2.26 (m, 1H), 2.07 – 2.01 (m, 1H), 1.94 – 1.83 (m, 2H); ^{13}C NMR (101 MHz, $CDCl_3$) δ 178.7, 162.6, 139.4, 135.9, 134.6 (q, *J* = 32.8 Hz), 132.5, 130.6,

129.9, 127.1, 126.8, 126.2, 125.5 (q, *J* = 3.6 Hz), 123.5 (q, *J* = 270.4 Hz), 46.5, 34.0, 30.5, 22.5, 19.9.

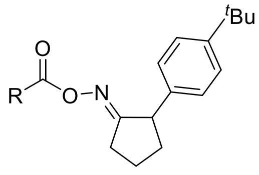

R = p-CF_3Ph
1d

Prepared according to general procedure (A) from cyclopentanone and 1-bromo-4-(*tert*-butyl)benzene to provide the title compound **1d** as a white solid[1]. ^{1}H NMR ($CDCl_3$, 400 MHz) δ 8.18 (d, *J* = 8.1 Hz, 2H), 7.73 (d, *J* = 8.3 Hz, 2H), 7.37 (d, *J* = 8.4 Hz, 2H), 7.27 (d, *J* = 8.0 Hz, 2H), 4.04 (t, *J* = 7.3 Hz, 1H), 2.97 – 2.91 (m, 1H), 2.85 – 2.78 (m, 1H), 2.40 – 2.22 (m, 1H), 2.15 – 1.97 (m, 2H), 1.93 – 1.78 (m, 1H), 1.31 (s, 9H); ^{13}C NMR ($CDCl_3$, 101 MHz) δ 178.4, 162.6, 149.6, 136.9, 134.5 (q, *J* = 32.7 Hz), 132.5, 129.9, 127.4, 125.5 (q, *J* = 3.3 Hz), 123.5 (q, *J* = 272.7 Hz), 48.8, 34.5, 34.3.31.3, 29.9, 22.5.

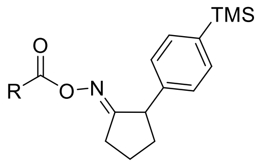

R = p-CF_3Ph
1e

Prepared according to general procedure (A) from cyclopentanone and (4-bromophenyl)trimethylsilane to provide the title compound **1e** as a yellowish oil[2]. ^{1}H NMR (400 MHz, $CDCl_3$) δ 8.17 (d, *J* = 8.1 Hz, 2H), 7.73 (d, *J* = 8.3 Hz, 2H), 7.50 (d, *J* = 8.1 Hz, 2H), 7.31 (d, *J* = 7.9 Hz, 2H), 4.05 (t, *J* = 7.2 Hz, 1H), 2.98 – 2.92 (m, 1H), 2.85 – 2.78 (m, 1H), 2.37 – 2.30 (m, 1H), 2.10 – 2.00 (m, 2H), 1.93 – 1.80 (m, 1H), 0.25 (s, 9H); ^{13}C NMR (101 MHz, $CDCl_3$) δ 178.3, 162.6, 140.6, 138.8, 134.6 (q, *J* = 32.7 Hz), 133.7, 132.5, 129.9, 127.2, 125.5 (q, *J* = 3.6 Hz), 123.5 (q, *J* = 272.9 Hz), 49.3, 34.6, 30.1, 22.6, -1.6.

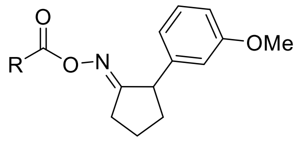

R = p-CF_3Ph
1f

Prepared according to general procedure (A) from cyclopentanone and 1-bromo-3-methoxybenzene to provide the title compound **1f** as a yellowish liquid (37% total yield). ^{1}H NMR ($CDCl_3$, 400 MHz) δ ^{1}H NMR (400 MHz, $CDCl_3$) δ 8.17 (d, *J* = 8.1 Hz, 2H), 7.73 (d, *J* = 8.2 Hz, 2H), 7.26 (t, *J* = 7.9 Hz, 1H), 6.94 – 6.88 (m, 2H), 6.82 – 6.76 (m, 1H), 4.05 (t, *J* = 7.1 Hz, 1H), 3.81 (s, 3H), 3.00 – 2.87 (m, 1H), 2.87 – 2.73 (m, 1H), 2.39 – 2.26 (m, 1H), 2.13 – 1.95 (m, 2H), 1.94 – 1.80 (m, 1H); ^{13}C NMR ($CDCl_3$, 100 MHz) δ 178.2, 162.7, 159.8, 141.8, 134.7 (q, *J* = 32.7 Hz), 130.1, 129.7, 125.6 (q, *J* = 3.7 Hz), 123.7 (q, *J* = 272.8 Hz), 120.3, 114.1, 112.1, 55.3, 49.2, 34.7, 30.1, 22.6. HRMS (ESI) calcd. for $C_{20}H_{18}O_3NF_3$

$[M+Na]^+$ *m/z* 400.1136, found 400.1138.

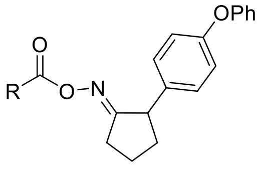

R = p-CF_3Ph
1g

Prepared according to general procedure (A) from cyclopentanone and 1-bromo-4-phenoxybenzene to provide the title compound **1g** as a yellowish liquid (44% total yield). ^{1}H NMR (400 MHz, $CDCl_3$) δ 8.16 (d, *J* = 8.1 Hz, 2H), 7.71 (d, *J* = 8.2 Hz, 2H), 7.37 – 7.25 (m, 4H), 7.11 – 7.04 (m, 1H), 7.03 – 6.93 (m, 4H), 4.02 (t, *J* = 7.6 Hz, 1H), 3.02 – 2.87 (m, 1H), 2.86 – 2.70 (m, 1H), 2.39 – 2.23 (m, 1H), 2.08 – 1.93 (m, 2H), 1.92 – 1.78 (m, 1H); ^{13}C NMR (101 MHz, $CDCl_3$) δ 178.2 , 162.6 , 157.2 , 156.1, 134.9 , 134.6 (q, *J* = 32.7 Hz), 132.6 , 130.0 , 129.7, 129.2, 125.6 (q, *J* = 3.7 Hz), 123.6 (q, *J* = 272.8 Hz), 123.3, 118.9, 48.7, 34.9, 30.0, 22.5. HRMS (ESI) calcd. for $C_{25}H_{20}O_3NF_3$ $[M+Na]^+$ *m/z* 462.1293, found 462.1291.

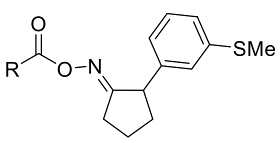

R = p-CF_3Ph
1h

Prepared according to general procedure (A) from cyclopentanone and (3-bromophenyl)(methyl)sulfane to provide the title compound **1h** as a yellowish solid (43% total yield). ^{1}H NMR (400 MHz, $CDCl_3$) δ 8.17 (d, *J* = 8.1 Hz, 2H), 7.72 (d, *J* = 8.3 Hz, 2H), 7.31 – 7.21 (m, 2H), 7.17 – 7.05 (m, 2H), 4.02 (t, *J* = 7.4 Hz, 1H), 3.04 – 2.88 (m, 1H), 2.87 – 2.73 (m, 1H), 2.48 (s, 3H), 2.39 – 2.25 (m, 1H), 2.10 – 1.93 (m, 2H), 1.92 – 1.77 (m, 1H); ^{13}C NMR (101 MHz, $CDCl_3$) δ 177.9, 162.6 , 140.9, 138.8, 134.6 (q, *J* = 32.7 Hz), 132.5, 130.0, 129.1 , 126.3, 125.6 (q, *J* = 3.7 Hz), 125.1 , 124.7, 123.6 (q, *J* = 272.8 Hz), 49.2 , 34.8 , 30.1 , 22.6, 15.9. HRMS (ESI) calcd. for $C_{20}H_{18}O_2NF_3S$ $[M+Na]^+$ *m/z* 416.0908, found 416.0901.

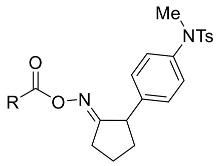

R = p-CF_3Ph
1i

Prepared according to general procedure (A) from cyclopentanone and N-(4-bromophenyl)-N,4-dimethylbenzenesulfonamide to provide the title compound **1i** as a yellowish solid (11% total yield). ^{1}H NMR (400 MHz, $CDCl_3$) δ 8.14 (d, *J* = 8.1 Hz, 2H), 7.70 (d, *J* = 8.3 Hz, 2H), 7.41 (d, *J* = 8.2 Hz, 2H), 7.26 – 7.20 (m, 4H), 7.04 (d, *J* = 8.5 Hz, 2H), 4.01 (t, *J* = 7.6 Hz, 1H), 3.10 (s, 3H), 3.00 – 2.87 (m, 1H), 2.86 – 2.72 (m, 1H), 2.37 (s, 3H), 2.36 – 2.26 (m, 1H), 2.06 – 1.92 (m, 2H), 1.91 – 1.78 (m, 1H); ^{13}C NMR (101 MHz, $CDCl_3$) δ 177.9, 162.6, 143.6, 140.2, 139.3, 134.5 (q, *J* = 32.7 Hz), 133.4, 132.4, 129.9, 129.4, 128.4, 127.8, 126.7, 125.5 (q, *J* = 3.6 Hz), 123.5 (q, *J* = 272.8 Hz), 48.8 ,

38.0 , 34.7 , 30.0 , 22.5 , 21.5. HRMS (ESI) calcd. for $C_{27}H_{25}O_4N_2F_3S$ $[M+Na]^+$ *m/z* 553.1385, found 553.1385.

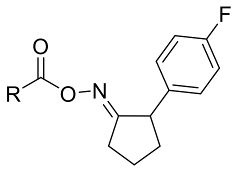

R = p-CF$_3$Ph
1j

Prepared according to general procedure (A) from cyclopentanone and 1-bromo-4-fluorobenzene to provide the title compound **1j** as a yellowish solid[1]. ^{1}H NMR (400 MHz, $CDCl_3$) δ 8.17 (d, *J* = 8.1 Hz, 2H), 7.73 (d, *J* = 8.3 Hz, 2H), 7.29 (dd, *J* = 8.6, 5.3 Hz, 2H), 7.03 (t, *J* = 8.7 Hz, 2H), 4.03 (t, *J* = 7.3 Hz, 1H), 2.98 – 2.92 (m, 1H), 2.83 – 2.76 (m, 1H), 2.37 – 2.31 (m, 1H), 2.05 – 1.97 (m, 2H), 1.91 – 1.83 (m, 1H); ^{13}C NMR (101 MHz, $CDCl_3$) δ 178.0, 162.7 (d, *J* = 20.7 Hz), 160.8, 135.7 (d, *J* = 3.3 Hz), 134.6 (q, *J* = 32.8 Hz), 132.4, 129.9, 129.4 (d, *J* = 8.0 Hz), 125.5 (q, *J* = 3.7 Hz), 123.5 (q, *J* = 272.8 Hz), 115.4 (d, *J* = 21.4 Hz), 48.6, 34.8, 29.9, 22.4.

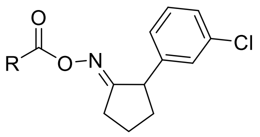

R = p-CF$_3$Ph
1k

Prepared according to general procedure (A) from cyclopentanone and 1-bromo-3-chlorobenzene to provide the title compound **1k** as a yellowish solid (43% total yield). ^{1}H NMR (400 MHz, $CDCl_3$) δ 8.18 (d, *J* = 8.1 Hz, 2H), 7.74 (d, *J* = 8.3 Hz, 2H), 7.37 – 7.17 (m, 4H), 4.03 (t, *J* = 7.5 Hz, 1H), 3.04 – 2.90 (m, 1H), 2.89 – 2.74 (m, 1H), 2.43 – 2.29 (m, 1H), 2.10 – 1.95 (m, 2H), 1.95 – 1.79 (m, 1H); ^{13}C NMR (101 MHz, $CDCl_3$) δ 177.6, 162.7 , 142.3, 134.8 (q, *J* = 32.8 Hz), 134.5, 132.5, 130.1, 130.0 , 128.1, 127.3, 126.4, 125.7 (q, *J* = 3.7 Hz), 123.7 (q, *J* = 272.9 Hz), 49.1, 34.9, 30.2, 22.6. HRMS (ESI) calcd. for $C_{19}H_{15}O_2NF_3Cl$ $[M+Na]^+$ *m/z* 404.0641, found 404.0638.

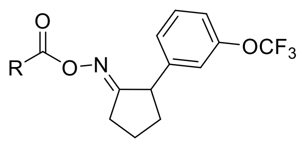

R = p-CF$_3$Ph
1l

Prepared according to general procedure (A) from cyclopentanone and 1-bromo-3-(trifluoromethoxy)benzene to provide the title compound **1l** as a yellowish solid (33% total yield). ^{1}H NMR (400 MHz, $CDCl_3$) δ 8.18 (d, *J* = 8.1 Hz, 2H), 7.74 (d, *J* = 8.2 Hz, 2H), 7.37 (t, *J* = 7.9 Hz, 1H), 7.28 (d, *J* = 7.9 Hz, 1H), 7.17 (s, 1H), 7.15 – 7.08 (m, 1H), 4.07 (t, *J* = 7.2 Hz, 1H), 3.06 – 2.91 (m, 1H), 2.91 – 2.73 (m, 1H), 2.45 – 2.28 (m, 1H), 2.12 – 1.96 (m, 2H), 1.96 – 1.81 (m, 1H); ^{13}C NMR (101 MHz, $CDCl_3$) δ 177.4, 162.7, 149.6, 142.6, 134.9

(q, J = 32.7 Hz), 132.6, 130.12, 130.08, 126.6, 125.7 (q, J = 3.7 Hz), δ 123.7 (q, J = 272.8 Hz), 120.7, 120.6 (q, J = 257.2 Hz),119.4, 49.1, 35.0, 30.2, 22.6. HRMS (ESI) calcd. for $C_{20}H_{15}O_3NF_6$ $[M+Na]^+$ m/z 454.0854, found 454.0856.

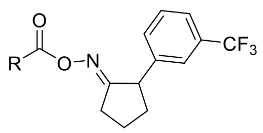

R = p-CF3Ph
1m

Prepared according to general procedure (A) from cyclopentanone and 1-bromo-3-(trifluoromethyl)benzene to provide the title compound **1m** as a yellowish solid (24% total yield). ^1H NMR (400 MHz, $CDCl_3$) δ 8.17 (d, J = 8.6 Hz, 2H), 7.73 (d, J = 8.6 Hz, 2H), 7.63 – 7.41 (m, 4H), 4.10 (t, J = 7.4 Hz, 1H), 3.08 – 2.94 (m, 1H), 2.92 – 2.77 (m, 1H), 2.48 – 2.33 (m, 1H), 2.14 – 1.96 (m, 2H), 1.96 – 1.80 (m, 1H); ^{13}C NMR (101 MHz, $CDCl_3$) δ 177.6, 162.7, 141.2, 134.9 (q, J = 32.8 Hz), 132.5, 131.7, 131.0 (q, J = 32.2 Hz), 130.1, 129.3, 125.7 (q, J = 3.7 Hz), 124.8 (q, J = 3.8 Hz), 124.2 (q, J = 272.3 Hz), 124.0 (q, J = 3.8 Hz), 123.7 (q, J = 272.7 Hz), 49.3, 35.1, 30.2, 22.7. HRMS (ESI) calcd. for $C_{20}H_{15}O_2NF_6$ $[M+Na]^+$ m/z 438.0905, found 438.0904.

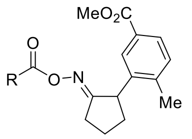

R = p-CF3Ph
1n

Prepared according to general procedure (A) from cyclopentanone and methyl 3-bromo-4-methylbenzoate to provide the title compound **1n** as a white solid (42% total yield). ^1H NMR (400 MHz, $CDCl_3$) δ 8.17 (d, J = 8.1 Hz, 2H), 7.85 – 7.78 (m, 2H), 7.73 (d, J = 8.3 Hz, 2H), 7.30 – 7.21 (m, 1H), 4.22 (t, J = 8.2 Hz, 1H), 3.87 (s, 3H), 3.08 – 2.86 (m, 2H), 2.47 (s, 3H), 2.42 – 2.27 (m, 1H), 2.19 – 2.02 (m, 1H), 2.03 – 1.80 (m, 2H); ^{13}C NMR (101 MHz, $CDCl_3$) δ 178.1, 167.2, 162.7, 142.0, 139.7, 134.8 (q, J = 32.7 Hz), 132.6, 130.9, 130.1, 128.5, 128.3, 128.2, 125.7(q, J = 3.7 Hz), 123.7(q, J = 272.6 Hz), 52.1, 46.5, 34.0, 30.6, 22.8, 20.3. HRMS (ESI) calcd. for $C_{22}H_{20}O_4NF_3$ $[M+Na]^+$ m/z 442.1242, found 442.1240.

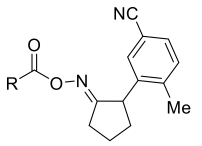

R = p-CF3Ph
1o

Prepared according to general procedure (A) from cyclopentanone and 3-bromo-4-methylbenzonitrile to provide the title compound **1o** as a yellowish solid (5% total yield). ^1H NMR (400 MHz, $CDCl_3$) δ 8.17 (d, J = 8.1 Hz, 2H), 7.74 (d, J = 8.3 Hz, 2H), 7.46 – 7.40 (m, 2H), 7.27 (d, J = 7.4 Hz, 1H), 4.20 (t, J = 7.6 Hz, 1H), 3.09 – 2.83 (m, 2H), 2.46 (s, 3H), 2.42 – 2.29 (m, 1H), 2.14 – 1.98 (m, 1H), 1.98 – 1.80 (m, 2H); ^{13}C NMR (101 MHz, $CDCl_3$) δ 177.4, 162.6, 142.2, 140.9, 134.8 (q, J = 32.8 Hz), 132.4 , 131.5 , 131.0,

130.5 , 130.1, 125.7 (q, J = 3.7 Hz), 123.6 (q, J = 272.9 Hz), 119.2 , 110.2, 46.3, 33.9 , 30.5, 22.7 , 20.4. HRMS (ESI) calcd. for $C_{21}H_{17}O_2N_2F_3$ $[M+Na]^+$ m/z 409.1140, found 409.1129.

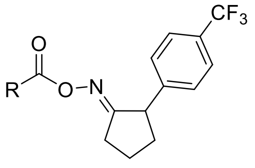

R = p-CF_3Ph
1p

Prepared according to general procedure (A) from cyclopentanone and 1-bromo-4-(trifluoromethyl)benzene to provide the title compound **1p** as a yellowish solid (36% total yield). ^{1}H NMR (400 MHz, Chloroform-d) δ 8.17 (d, J = 8.2 Hz, 2H), 7.73 (d, J = 8.2 Hz, 2H), 7.59 (d, J = 8.2 Hz, 2H), 7.45 (d, J = 8.1 Hz, 2H), 4.09 (t, J = 7.6 Hz, 1H), 3.08 – 2.93 (m, 1H), 2.91 – 2.74 (m, 1H), 2.47 – 2.31 (m, 1H), 2.12 – 1.96 (m, 2H), 1.95 – 1.80 (m, 1H); ^{13}C NMR (101 MHz, Chloroform-d) δ 177.6, 162.7, 144.3, 134.8 (q, J = 32.7 Hz), 132.5, 130.1, 129.3 (q, J = 32.5 Hz), 128.5, 125.7 (q, J = 3.8 Hz), 124.3 (q, J = 270.3 Hz), 123.6 (q, J = 272.8 Hz), 49.4, 35.0, 30.2, 22.7. HRMS (ESI) calcd. for $C_{20}H_{15}O_2NF_6$ $[M+H]^+$ m/z 416.1085, found 416.1072.

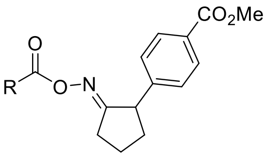

R = p-CF_3Ph
1q

Prepared according to general procedure (A) from cyclopentanone and methyl 4-bromobenzoate to provide the title compound **1q** as a yellowish solid[2]. ^{1}H NMR (400 MHz, $CDCl_3$) δ 8.17 (d, J = 8.1 Hz, 2H), 7.73 (d, J = 8.3 Hz, 2H), 7.25 (d, J = 8.7 Hz, 2H), 6.88 (d, J = 8.7 Hz, 2H), 4.01 (t, J = 7.2 Hz, 1H), 3.79 (s, 3H), 3.00 – 2.87 (m, 1H), 2.84 – 2.73 (m, 1H), 2.36 – 2.23 (m, 1H), 2.07 – 1.96 (m, 2H), 1.90 – 1.80 (m, 1H); ^{13}C NMR (101 MHz, $CDCl_3$) δ 178.4, 162.6,158.4, 134.6 (q, J = 32.7 Hz), 132.5, 131.9, 129.9, 128.8, 125.5 (q, J = 3.7 Hz), 123.5(q, J = 272.8 Hz), 114.0, 55.2, 48.5, 34.6, 29.9, 22.4. HRMS (ESI) calcd. for $C_{21}H_{18}O_4NF_3$ $[M+H]^+$ m/z 406.1266, found 406.1254.

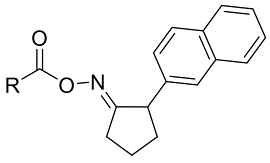

R = p-CF_3Ph
1r

Prepared according to general procedure (A) from cyclopentanone and 2-bromonaphthalene to provide the title compound **1r** as a yellowish solid[1]. ^{1}H NMR (400 MHz, $CDCl_3$) δ 8.18 (d, J = 8.1 Hz, 2H), 7.88 – 7.79 (m, 3H), 7.77 – 7.72 (m, 3H), 7.50 – 7.42 (m, 3H), 4.24 (t, J = 7.7 Hz, 1H),

3.02 – 2.96 (m, 1H), 2.91 – 2.84 (m, 1H), 2.44 – 2.37 (m, 1H), 2.22 – 2.15 (m, 1H), 2.11 – 2.03 (m, 1H), 1.97 –1.86 (m, 1H); ^{13}C NMR (101 MHz, $CDCl_3$) δ 178.1, 162.6, 137.4, 134.6 (q, *J* = 32.9 Hz), 133.4, 132.5, 132.4, 130.0, 128.4, 127.8, 127.6, 126.4, 126.1, 126.2, 125.7, 125.5(q, *J* = 3.7 Hz), 123.5 (q, *J* = 272.8 Hz), 49.4, 34.6, 30.1, 22.6.

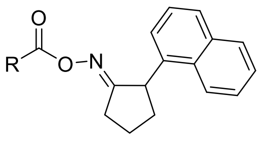

R = p-CF_3Ph
1s

Prepared according to general procedure (A) from cyclopentanone and 1-bromonaphthalene to provide the title compound **1s** as a yellowish solid (20% total yield). ^{1}H NMR (400 MHz, $CDCl_3$) δ 8.22 (d, *J* = 8.1 Hz, 2H), 8.06 (d, *J* = 8.3 Hz, 1H), 7.88 (d, *J* = 7.8 Hz, 1H), 7.81 – 7.71 (m, 3H), 7.60 – 7.48 (m, 2H), 7.44 (t, *J* = 7.7 Hz, 1H), 7.34 (d, *J* = 7.1 Hz, 1H), 4.86 (t, *J* = 7.0 Hz, 1H), 3.03 (t, *J* = 7.5 Hz, 2H), 2.54 – 2.35 (m, 1H), 2.14 – 2.03 (m, 1H), 2.03 – 1.87 (m, 2H); ^{13}C NMR (101 MHz, $CDCl_3$) δ 178.7 , 162.6 , 137.4 , 134.6 (q, *J* = 32.7 Hz), 134.1 , 132.6, 131.3, 130.0 , 129.0 , 127.6 , 126.1 , 125.7 , 125.6 (q, *J* = 3.7 Hz), 125.4, 124.7 , 123.6 (q, *J* = 272.7 Hz), 123.5 , 46.0 , 34.7 , 30.6 , 22.6 . HRMS (ESI) calcd. for $C_{23}H_{18}O_2NF_3$ $[M+Na]^+$ *m/z* 420.1187, found 420.1186.

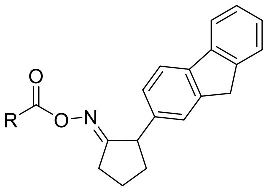

R = p-CF_3Ph
1t

Prepared according to general procedure (A) from cyclopentanone and 2-bromo-9*H*-fluorene to provide the title compound **1t** as a yellowish solid (38% total yield). ^{1}H NMR (400 MHz, $CDCl_3$) δ 8.19 (d, *J* = 8.1 Hz, 2H), 7.75 (dt, *J* = 8.8, 4.8 Hz, 4H), 7.57 – 7.48 (m, 2H), 7.40 – 7.28 (m, 3H), 4.15 (t, *J* = 7.8 Hz, 1H), 3.90 (s, 2H), 3.08 – 2.78 (m, 2H), 2.47 – 2.33 (m, 1H), 2.20 – 2.01 (m, 2H), 1.99 – 1.83 (m, 1H); ^{13}C NMR (101 MHz, $CDCl_3$) δ 178.6, 162.8, 143.8, 143.4, 141.5, 140.7, 138.9 , 134.7 (q, *J* = 32.9 Hz), 132.7, 130.1, 126.8, 126.7, 126.6, 125.7 (q, *J* = 3.7 Hz), 125.1, 124.7, 123.7 (q, *J* = 272.8 Hz), 120.1, 119.9, 49.5 , 37.0, 35.0, 30.2, 22.7. HRMS (ESI) calcd. for $C_{26}H_{20}O_2NF_3$ $[M+Na]^+$ *m/z* 458.1344, found 458.1342.

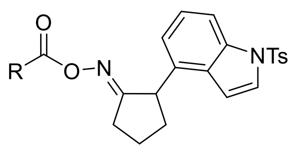

R = p-CF_3Ph
1u

Prepared according to general procedure (A) from cyclopentanone and 4-bromo-1-tosyl-1*H*-indole to provide the title compound **1u** as a yellowish solid (28% total yield). ^{1}H NMR (400 MHz, $CDCl_3$) δ 8.16 (d, *J* = 8.2 Hz, 2H), 7.88 (d, *J* = 8.3 Hz, 1H), 7.75 (d, *J* = 8.5 Hz, 2H), 7.73 (d, *J* = 8.9 Hz, 2H), 7.60 (d, *J* = 3.7 Hz, 1H), 7.32 – 7.19 (m, 3H), 7.06 (d, *J* = 7.5 Hz, 1H), 6.71 (d, *J* = 3.7 Hz, 1H), 4.34 (t, *J* = 7.2 Hz, 1H), 3.06 – 2.84 (m, 2H), 2.42 – 2.28 (m, 4H), 2.09 – 1.85 (m, 3H); ^{13}C NMR (101 MHz, $CDCl_3$) δ 177.9, 162.7, 145.2, 135.3, 135.1, 134.8 (q, *J* = 32.6 Hz), 133.9, 132.6, 130.1, 130.1, 129.7, 127.0, 126.3, 125.7 (q, *J* = 3.6 Hz), 124.9, 123.7 (q, *J* = 272.7 Hz), 121.8, 112.5, 107.2, 47.1, 34.8, 30.5, 22.9, 21.7. HRMS (ESI) calcd. for $C_{28}H_{23}O_4N_2F_3S$ $[M+Na]^+$ *m/z* 563.1228, found 563.1224.

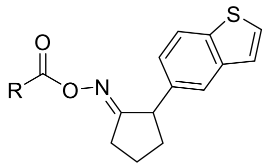

R = p-CF_3Ph
1v

Prepared according to general procedure (A) from cyclopentanone and 5-bromobenzo[b]thiophene to provide the title compound **1v** as a yellowish solid[1]. ^{1}H NMR (400 MHz, $CDCl_3$) δ 8.18 (d, *J* = 8.1 Hz, 2H), 7.85 (d, *J* = 8.4 Hz, 1H), 7.80 – 7.69 (m, 3H), 7.43 (d, *J* = 5.4 Hz, 1H), 7.35 – 7.28 (m, 2H), 4.19 (t, *J* = 7.9 Hz, 1H), 3.02 – 2.94 (m, 1H), 2.91 – 2.78 (m, 1H), 2.43 – 2.35 (m, 1H), 2.20 – 2.02 (m, 2H), 1.95 – 1.83 (m, 1H); ^{13}C NMR (101 MHz, $CDCl_3$) δ 178.3, 162.6, 139.9, 138.4, 136.2, 134.6 (q, *J* = 32.7 Hz), 132.5, 129.9, 126.8, 125.5 (q, *J* = 3.7 Hz), 124.4, 123.8, 123.5 (q, *J* = 272.8 Hz), 122.7, 49.2, 34.9, 30.0, 22.5.

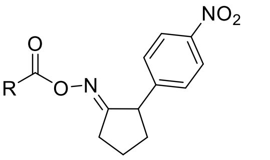

R = p-CF_3Ph
1w

Prepared according to general procedure (A) from cyclopentanone and 1-bromo-4-nitrobenzene to provide the title compound **1w** as a yellow oil (18% total yield). ^{1}H NMR (400 MHz, Chloroform-*d*) δ 8.23 – 8.07 (m, 4H), 7.71 (d, *J* = 8.3 Hz, 2H), 7.48 (d, *J* = 8.7 Hz, 2H), 4.11 (t, *J* = 8.3 Hz, 1H), 3.12 – 2.94 (m, 1H), 2.93 – 2.77 (m, 1H), 2.51 – 2.33 (m, 1H), 2.14 – 1.81 (m, 3H); ^{13}C NMR (101 MHz, Chloroform-*d*) δ 177.1, 162.5, 147.8, 146.9, 134.7 (q, *J* = 32.8 Hz), 132.3, 130.0 , 129.1, 125.6 (q, *J* = 3.7 Hz), 123.8, 123.5 (q, *J* = 272.8 Hz), 49.4, 35.0, 30.2, 22.7. HRMS (ESI) calcd. for $C_{19}H_{15}O_4N_2F_3$ $[M+H]^+$ *m/z* 393.1062, found 393.1057.

The procedure for synthesis of the substrate 1x.

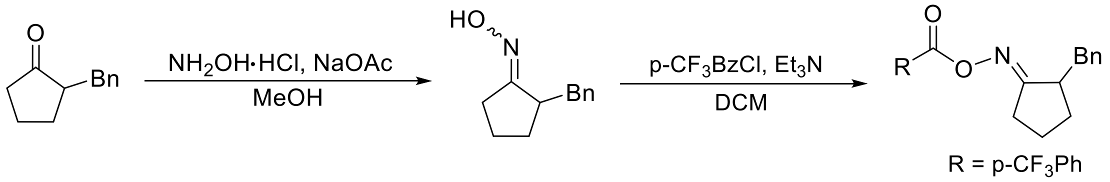

The 2-benzylcyclopentan-1-one were synthesized according to the reported procedure[4]. 2-benzylcyclopentan-1-one (1.0 equiv, 12.6 mmol), hydroxylamine hydrochloride (1.2 equiv) and sodium acetate (1.5 equiv) were added to a round bottom flask containing a stirrer bar. MeOH (0.56 M) was added and the reaction was stirred for 12 h at 75 ºC. Then methanol was removed under vacuum and the resulting mixture was extracted with DCM. The organic layer was washed with water and dried over Na_2SO_4. The solvent was removed under reduced pressure and the crude material was subjected to column chromatography to afford oximes.

To a mixture of oxime (1.0 equiv, 6.7 mmol), triethylamine (1.5 equiv) and DCM (0.5 M) in a flask was added 4-(trifluoromethyl)benzoyl chlorides (1.1 equiv) slowly at 0 °C. After 1 h, a saturated solution of aqueous $NaHCO_3$ (10 mL) was added to the above solution, and the mixture was diluted with DCM. The organic layer was washed with brine (20 mL) and dried over Na_2SO_4. The solvent was removed under vacuum and the residue was subjected to column chromatography on silica gel with EtOAc–petroleum ether as an eluent to give the substrate.

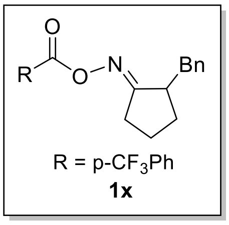

Prepared from 2-benzylcyclopentan-1-one to provide the title compound **1x** as a white solid (77% total yield). ^{1}H NMR (400 MHz, Chloroform-*d*) δ 8.20 (d, *J* = 8.1 Hz, 2H), 7.74 (d, *J* = 8.2 Hz, 2H), 7.38 – 7.18 (m, 5H), 3.39 (dd, *J* = 13.7, 4.2 Hz, 1H), 3.14 – 3.01 (m, 1H), 2.93 – 2.78 (m, 1H), 2.77 – 2.64 (m, 2H), 1.97 – 1.83 (m, 2H), 1.77 – 1.63 (m, 1H), 1.62 – 1.50 (m, 1H); ^{13}C NMR (101 MHz, Chloroform-*d*) δ 178.1, 162.9, 139.8, 134.8 (q, *J* = 32.7 Hz), 132.7, 130.1, 129.2, 128.6, 126.4, 125.7 (q, *J* = 3.7 Hz), 123.7 (q, *J* = 272.8 Hz), 45.8, 38.1, 30.9, 29.9, 22.3. HRMS (ESI) calcd. for $C_{20}H_{18}O_2NF_3$ $[M+H]^+$ *m/z* 362.1368, found 362.1361.

Genaral procedure (B) for synthesis of the substrates 3[5-9].

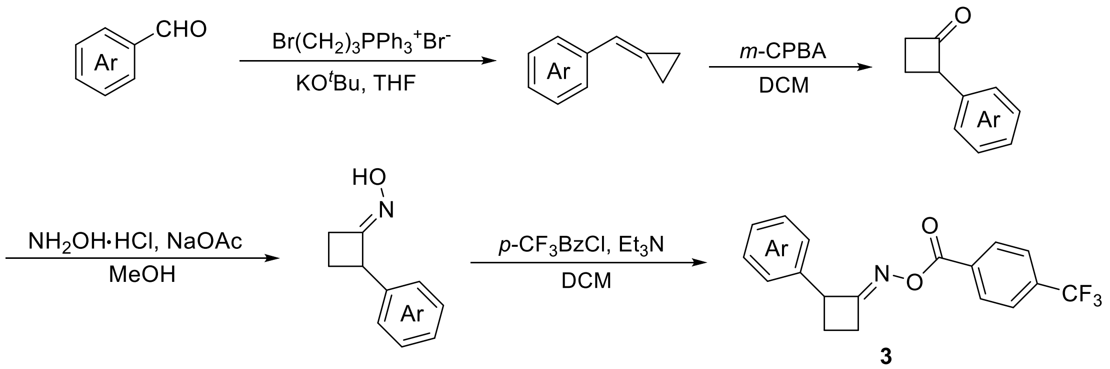

A solution of KO^{t}Bu (3.0 equiv) in THF (1.3 M) was slowly added to a solution of (3-bromopropyl)triphenylphosphonium bromide (1.5 equiv) in dry THF (0.5 M) and stirred at 70 °C for 1 h. Then a THF solution of benzaldehyde (~20 mmol, 2.0 M in THF, 1.0 equiv) was added dropwise and the mixture was refluxed for 6 h. After cooling, the suspension was filtered and the solvent of the filtrate was removed under vacuum, the products were purified by column chromatography on silica gel to afford (phenylmethylene)cyclopropanes.

To the solution of (phenylmethylene)cyclopropanes (~15 mmol, 1.0 equiv) in DCM (0.15 M) was added a solution of *m*-CPBA (1.0 equiv) in DCM (0.38 M) dropwise at 0 °C and stirred at 0 °C (as for synthesis of substrates **3m**, **3n**, **3o**, **3p**, temperature should be elevated to RT) for ~1 h (detected by TLC). Then, the solution was diluted with a saturated solution of aqueous Na_2SO_3 (30 mL) and extracted with DCM (3*20 mL). The organic phase was washed successively with a saturated solution of aqueous $NaHCO_3$ (2*30 mL), and brine (30 mL), then dried over Na_2SO_4 and concentrated in vacuum. The crude material was then purified by column chromatography on silica gel with a mixture of petroleum ether and ethyl acetate to give various cyclobutanones.

To a mixture of hydroxylamine hydrochloride (1.2 equiv), sodium acetate (1.5 equiv), methanol (0.56 M) in a flask was added cyclobutanone (~5 mmol, 1.0 equiv) and the mixture was stirred at 75 °C for 12 h. The reaction mixture was cooled to room

temperature and then methanol was removed under vacuum and the resulting mixture was extracted with DCM. The organic layer was washed with water and dried over Na_2SO_4. The solvent was removed under reduced pressure and the crude material was subjected to column chromatography to afford cyclobutanone oximes.

To a mixture of cyclobutanone oxime (~3 mmol, 1.0 equiv), triethylamine (1.5 equiv) and DCM (0.5 M) in a flask was added 4-(trifluoromethyl)benzoyl chlorides (1.1 equiv) slowly at 0 °C. After 1 h, a saturated solution of aqueous $NaHCO_3$ (10 mL) was added to the above solution, and the mixture was diluted with DCM. The organic layer was washed with brine and dried over Na_2SO_4. The solvent was removed under vacuum and the residue was subjected to column chromatography on silica gel with EtOAc–petroleum ether as an eluent to give cyclobutanone *O*-(4-(trifluoromethyl)benzoyl) oximes.

Characterization data for cyclobutanone oxime esters 3

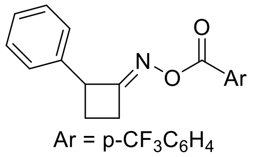

3a

Prepared according to general procedure (B) from benzaldehyde to provide the title compound **3a** as a yellowish solid[5]. ^{1}H NMR (400 MHz, $CDCl_3$) δ 8.17 (d, *J* = 8.1 Hz, 2H), 7.74 (d, *J* = 8.2 Hz, 2H), 7.44 – 7.26 (m, 5H), 4.70 (ddd, *J* = 10.0, 7.1, 2.8 Hz, 1H), 3.31 – 3.07 (m, 2H), 2.65 (dtd, *J* = 11.3, 9.6, 6.1 Hz, 1H), 2.31 (ddt, *J* = 11.2, 9.9, 7.3 Hz, 1H); ^{13}C NMR (101 MHz, $CDCl_3$) δ 171.6, 162.7, 138.5, 134.7 (q, *J* = 33.3 Hz), 132.4, 130.0, 128.7, 127.2, 127.1, 125.5 (q, *J* = 4.0 Hz), 123.5 (q, *J* = 274.7 Hz), 49.7, 29.6, 23.2.

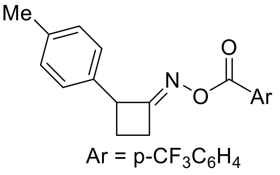

3b

Prepared according to general procedure (B) from 4-methylbenzaldehyde to provide the title compound **3b** as a yellowish solid[5]. ^{1}H NMR (400 MHz, $CDCl_3$) δ 8.21–8.13 (m, 2H), 7.73 (d, *J* = 8.2 Hz, 2H), 7.29 (d, *J* = 7.8 Hz, 2H), 7.17 (d, *J* = 7.9 Hz, 2H), 4.66 (ddd, *J* = 10.0, 6.9, 2.9 Hz, 1H), 3.34– 3.04 (m, 2H), 2.62 (dtd, *J* = 11.3, 9.7, 6.1 Hz, 1H), 2.34 (s, 3H), 2.33–2.22 (m, 1H); ^{13}C NMR (101 MHz, $CDCl_3$) δ 171.8, 162.7, 136.8, 135.5, 134.7 (q, *J* = 32.3 Hz), 132.4, 130.0, 129.3, 127.0, 125.5 (q, *J* = 4.0 Hz), 123.5 (q, *J* = 273.7 Hz), 49.4, 29.5, 23.2, 21.1.

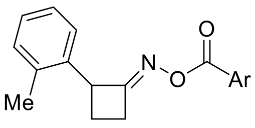

Ar = p-$CF_3C_6H_4$

3c

Prepared according to general procedure (B) from 2-methylbenzaldehyde to provide the title compound **3c** as a white solid[5]. ^{1}H NMR (400 MHz, $CDCl_3$) δ 8.19 (d, *J* = 8.1 Hz, 2H), 7.74 (d, *J* = 8.2 Hz, 2H), 7.60–7.53 (m, 1H), 7.27–7.13 (m, 3H), 4.80 (ddd, *J* = 10.0, 7.1, 3.0 Hz, 1H), 3.33–3.03 (m, 2H), 2.65 (dtd, *J* = 11.2, 9.6, 6.1 Hz, 1H), 2.33 (s, 3H), 2.22–2.05 (m, 1H); ^{13}C NMR (101 MHz, $CDCl_3$) δ 171.6, 162.7, 136.9, 135.5, 134.7 (q, *J* = 33.3 Hz), 132.4, 130.4, 130.0, 127.2, 126.2, 126.1, 125.5 (q, *J* = 4.0 Hz), 123.5 (q, *J* = 243.4 Hz), 47.9, 29.3, 23.4, 19.5.

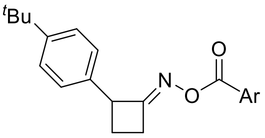

Ar = p-$CF_3C_6H_4$

3d

Prepared according to general procedure (B) from 4-(tert-butyl)benzaldehyde to provide the title compound **3d** as a white solid (5% total yield). ^{1}H NMR (400 MHz, $CDCl_3$) δ 8.17 (d, *J* = 8.1 Hz, 2H), 7.74 (d, *J* = 8.3 Hz, 2H), 7.43 – 7.30 (m, 4H), 4.70 – 4.61 (m, 1H), 3.31 – 3.04 (m, 2H), 2.69 – 2.52 (m, 1H), 2.38 – 2.24 (m, 1H), 1.32 (s, 9H); ^{13}C NMR (101 MHz, $CDCl_3$) δ 172.1, 162.9, 150.2, 135.5, 134.8 (q, *J* = 32.7 Hz), 132.6, 130.1, 127.0, 125.73, 125.67 (q, *J* = 3.7 Hz), 123.7 (q, *J* = 272.7 Hz), 49.5, 34.6, 31.4, 29.7, 23.2. HRMS (ESI) calcd. for $C_{22}H_{22}O_2NF_3$ $[M+Na]^+$ *m/z* 412.1500, found 412.1498.

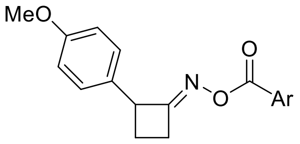

Ar = p-$CF_3C_6H_4$

3e

Prepared according to general procedure (B) from 4-methoxybenzaldehyde to provide the title compound **3e** as a yellowish solid[5]. ^{1}H NMR (400 MHz, $CDCl_3$) δ 7.63–7.40 (m, 4H), 7.22 (d, *J* = 8.7 Hz, 2H), 6.87 (d, *J* = 8.7 Hz, 2H), 4.50 (ddd, *J* = 10.2, 7.4, 2.8 Hz, 1H), 3.77 (s, 3H), 3.28–3.08 (m, 2H), 2.65–2.49 (m, 1H), 2.23–2.08 (m, 1H); ^{13}C NMR (101 MHz, $CDCl_3$) δ 170.5, 162.5, 158.8, 134.3 (q, *J* = 32.3 Hz), 131.9, 131.0, 129.8, 128.4, 125.1 (q, *J* = 3.0 Hz), 123.4 (q, *J* = 273.7 Hz), 114.1, 55.2, 50.8, 29.1, 24.3.

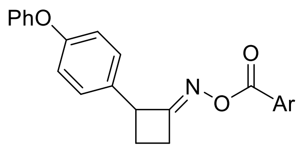

Ar = p-$CF_3C_6H_4$
3f

Prepared according to general procedure (B) from 4-phenoxybenzaldehyde to provide the title compound **3f** as a yellowish solid (3% total yield). ^{1}H NMR (400 MHz, $CDCl_3$) δ 8.17 (d, *J* = 8.0 Hz, 2H), 7.74 (d, *J* = 8.2 Hz, 2H), 7.42 – 7.30 (m, 4H), 7.13 – 7.06 (m, 1H), 7.05 – 6.97 (m, 4H), 4.73 – 4.60 (m, 1H), 3.31 – 3.08 (m, 2H), 2.71 – 2.57 (m, 1H), 2.37 – 2.21 (m, 1H); ^{13}C NMR (101 MHz, $CDCl_3$) δ 171.9, 162.8, 157.3, 156.4, 134.8 (q, *J* = 32.7 Hz), 133.5, 132.5, 130.1, 129.8, 128.7, 125.7 (q, *J* = 3.7 Hz), 123.7 (q, *J* = 272.9 Hz), 123.4, 119.2, 118.9, 49.2, 29.6, 23.5. HRMS (ESI) calcd. for $C_{24}H_{18}O_3NF_3$ $[M+Na]^+$ *m/z* 448.1136, found 448.1138.

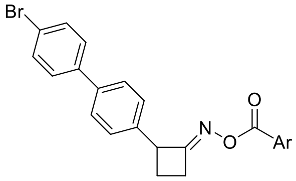

Ar = p-$CF_3C_6H_4$
3g

Prepared according to general procedure (B) from 4'-bromo-[1,1'-biphenyl]-4-carbaldehyde to provide the title compound **3g** as a yellowish solid (3% total yield). ^{1}H NMR (400 MHz, $CDCl_3$) δ 8.18 (d, *J* = 8.1 Hz, 2H), 7.74 (d, *J* = 8.2 Hz, 2H), 7.58 – 7.51 (m, 4H), 7.51 – 7.40 (m, 4H), 4.78 – 4.67 (m, 1H), 3.33 – 3.13 (m, 2H), 2.74 – 2.60 (m, 1H), 2.41 – 2.26 (m, 1H); ^{13}C NMR (101 MHz, $CDCl_3$) δ 171.6, 162.9, 139.7, 139.0, 138.1, 134.8 (q, *J* = 32.6 Hz), 132.4, 132.0, 130.1, 128.8, 127.7, 127.3, 125.7 (q, *J* = 3.6 Hz), 123.7 (q, *J* = 272.7 Hz), 121.7, 49.5, 29.8, 23.3. HRMS (ESI) calcd. for $C_{24}H_{17}O_2NF_3Br$ $[M+Na]^+$ *m/z* 510.0292, found 510.0292.

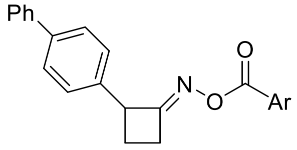

Ar = p-$CF_3C_6H_4$
3h

Prepared according to general procedure (B) from [1,1'-biphenyl]-4-carbaldehyde to provide the title compound **3h** as a yellowish solid (8% total yield). ^{1}H NMR (400 MHz, $CDCl_3$) δ 8.21 – 8.15 (m, 2H), 7.75 (d, *J* = 8.2 Hz, 2H), 7.62 – 7.56 (m, 4H), 7.51 – 7.41 (m, 4H), 7.39 – 7.32 (m, 1H), 4.80 – 4.69 (m, 1H), 3.37 – 3.11 (m, 2H), 2.75 – 2.62 (m, 1H), 2.43 – 2.27 (m, 1H); ^{13}C NMR (101 MHz, $CDCl_3$) δ 171.7, 162.7, 140.7, 140.1, 137.6, 134.6 (q, *J* = 32.8 Hz), 132.4, 130.0, 128.8, 127.6, 127.4, 127.3, 127.0, 125.5, 123.6 (q, *J* = 272.8 Hz), 49.4, 29.6, 23.2. HRMS (ESI) calcd. for $C_{24}H_{18}O_2NF_3$ $[M+Na]^+$ *m/z* 432.1187, found 432.1178.

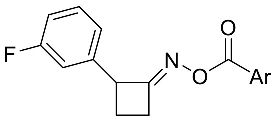

Ar = p-$CF_3C_6H_4$

3i

Prepared according to general procedure (B) from 3-fluorobenzaldehyde to provide the title compound **3i** as a yellowish solid[5]. ^{1}H NMR (400 MHz, $CDCl_3$): δ 7.62–7.51 (m, 4H), 7.33 (td, *J* = 7.9, 6.0 Hz, 1H), 7.10 (d, *J* = 7.7 Hz, 1H), 7.06–6.94 (m, 2H), 4.56 (ddd, *J* = 10.2, 7.2, 2.8 Hz, 1H), 3.34– 3.13 (m, 2H), 2.64 (dtd, *J* = 11.5, 9.7, 6.2 Hz, 1H), 2.21 (ddt, *J* = 11.5, 9.9, 7.6 Hz, 1H); ^{13}C NMR (101 MHz, $CDCl_3$): δ 169.1, 163.1 (d, *J* = 247.4 Hz), 162.4, 141.3 (d, *J* = 7.1 Hz), 134.6 (d, *J* = 33.3 Hz), 131.8, 130.4 (d, *J* = 9.1 Hz), 129.8, 125.3 (q, *J* = 4.0 Hz), 123.5 (q, *J* = 273.7 Hz), 123.0 (d, *J* = 3.0 Hz), 114.5 (d, *J* = 18.2 Hz), 114.3 (d, *J* = 18.2 Hz), 50.9 (d, *J* = 2.0 Hz), 29.4, 24.2.

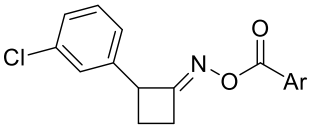

Ar = p-$CF_3C_6H_4$

3j

Prepared according to general procedure (B) from 3-chlorobenzaldehyde to provide the title compound **3j** as a yellowish solid (3% total yield). ^{1}H NMR (400 MHz, $CDCl_3$): δ 8.05 (d, *J* = 7.4 Hz, 2H), 7.61 (d, *J* = 8.1 Hz, 2H), 7.27 (s, 1H), 7.24 – 7.07 (m, 3H), 4.63 – 4.45 (m, 1H), 3.25 – 2.97 (m, 2H), 2.66 – 2.44 (m, 1H), 2.27 – 2.06 (m, 1H); ^{13}C NMR (101 MHz, $CDCl_3$): δ 170.9, 162.7, 140.5, 134.7 (q, *J* = 32.6 Hz), 134.5, 132.3, 130.03, 130.00, 127.4, 127.3, 125.6 (q, *J* = 3.6 Hz), 125.5, 123.6 (q, *J* = 272.8 Hz), 49.1, 29.6, 23.2. HRMS (ESI) calcd. for $C_{18}H_{13}O_2NF_3Cl$ $[M+Na]^+$ *m/z* 390.0485, found 390.0484.

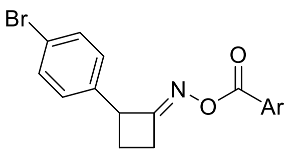

Ar = p-$CF_3C_6H_4$

3k

Prepared according to general procedure (B) from 4-bromobenzaldehyde to provide the title compound **3k** as a yellow solid[5]. ^{1}H NMR (400 MHz, $CDCl_3$) δ 8.16 (d, *J* = 8.1 Hz, 2H), 7.73 (d, *J* = 8.3 Hz, 2H), 7.47 (d, *J* = 8.4 Hz, 2H), 7.29 (d, *J* = 8.2 Hz, 2H), 4.64 (ddd, *J* = 10.1, 7.1, 2.9 Hz, 1H), 3.37–3.06 (m, 2H), 2.64 (dtd, *J* = 11.4, 9.6, 6.1 Hz, 1H), 2.38–2.17 (m, 1H); ^{13}C NMR (101 MHz, $CDCl_3$) δ 170.9, 162.6, 137.4, 134.7 (q, *J* = 33.3 Hz), 132.3, 131.8, 130.0, 128.8, 125.5 (q, *J* = 3.0 Hz), 123.5 (q, *J* = 274.7 Hz), 121.1, 49.0, 29.5, 23.2.

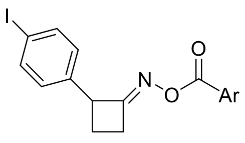

Ar = p-$CF_3C_6H_4$
3l

Prepared according to general procedure (B) from 4-iodobenzaldehyde to provide the title compound **3l** as a yellowish solid[5]. ^{1}H NMR (400 MHz, $CDCl_3$) δ 8.16 (d, J = 8.1 Hz, 2H), 7.73 (d, *J* = 8.2 Hz, 2H), 7.68 (d, *J* = 8.4 Hz, 2H), 7.16 (d, *J* = 8.0 Hz, 2H), 4.63 (ddd, *J* = 10.0, 7.1, 2.8 Hz, 1H), 3.31–3.05 (m, 2H), 2.64 (dtd, *J* = 11.4, 9.6, 6.1 Hz, 1H), 2.25 (ddt, *J* = 11.4, 10.0, 7.4 Hz, 1H); ^{13}C NMR (101 MHz, $CDCl_3$) δ 170.9, 162.6, 138.1, 137.8, 134.8 (q, *J* = 32.3 Hz), 132.3, 130.0, 129.1, 125.6 (q, *J* = 4.0 Hz), 123.5 (q, *J* = 273.7 Hz), 92.6, 49.1, 29.6, 23.1.

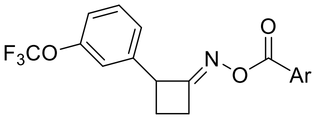

Ar = p-$CF_3C_6H_4$
3m

Prepared according to general procedure (B) from 3-(trifluoromethoxy)benzaldehyde to provide the title compound **3m** as a white solid (4 % total yield). ^{1}H NMR ($CDCl_3$, 400 MHz) δ 8.20 – 8.13 (m, 2H), 7.73 (dd, *J* = 8.8, 0.6 Hz, 2H), 7.40 – 7.37 (m, 2H), 7.25 – 7.22 (m, 1H), 7.17 – 7.09 (m, 1H), 4.75 – 4.65 (m, 1H), 3.32 – 3.09 (m, 2H), 2.75 – 2.59 (m, 1H), 2.36 – 2.20 (m, 1H); ^{13}C NMR ($CDCl_3$, 101 MHz) δ 170.8, 162.8, 149.6 (q, *J* = 1.8 Hz), 140.9, 134.9 (q, *J* = 32.7 Hz), 132.4, 130.2, 130.2, 125.8, 125.7 (q, *J* = 3.8 Hz), 123.7 (q, *J* = 272.8 Hz), 120.6 (q, *J* = 257.2 Hz), 119.9, 119.7, 49.2, 29.7, 23.4. HRMS (ESI) calcd. for $C_{19}H_{13}O_3NF_6$ $[M+Na]^+$ *m/z* 440.0697, found 440.0700.

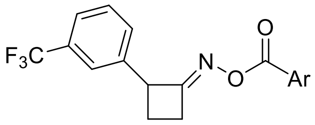

Ar = p-$CF_3C_6H_4$
3n

Prepared according to general procedure (B) from 3-(trifluoromethyl)benzaldehyde to provide the title compound **3n** as a yellowish liquid (4% total yield). ^{1}H NMR (400 MHz, $CDCl_3$) δ 8.16 (d, *J* = 8.7 Hz, 2H), 7.72 (d, *J* = 8.6 Hz, 2H), 7.68 – 7.58 (m, 2H), 7.56 – 7.42 (m, 2H), 4.84 – 4.66 (m, 1H), 3.40 – 3.11 (m, 2H), 2.78 – 2.61 (m, 1H), 2.40 – 2.21 (m, 1H); ^{13}C NMR (101 MHz, $CDCl_3$) δ 170.8, 162.8, 139.6, 134.9 (q, *J* = 32.7 Hz), 132.3, 131.1 (q, *J* = 32.3 Hz), 130.9, 130.1, 129.4, 125.69 (q, *J* = 3.7 Hz), 124.2 (q, *J* = 3.8 Hz), 124.16 (q, *J* = 272.4 Hz), 123.99 (q, *J* = 3.8 Hz), 123.65 (q, *J* = 272.7 Hz), 49.3, 29.7, 23.4. HRMS (ESI) calcd. for $C_{19}H_{13}O_2NF_6$ $[M+Na]^+$ *m/z* 423.0748, found 424.0753.

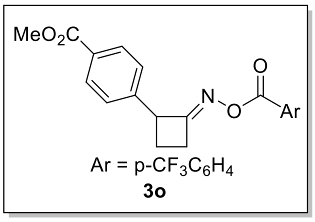

Prepared according to general procedure (B) from methyl 4-formylbenzoate to provide the title compound **3o** as a yellowish solid (2% total yield). ^{1}H NMR (400 MHz, $CDCl_3$) δ 8.14 (d, *J* = 8.2 Hz, 2H), 8.00 (d, *J* = 8.2 Hz, 2H), 7.71 (d, *J* = 8.1 Hz, 2H), 7.47 (d, *J* = 8.2 Hz, 2H), 4.79 – 4.63 (m, 1H), 3.89 (s, 3H), 3.32 – 3.08 (m, 2H), 2.75 – 2.59 (m, 1H), 2.36 – 2.20 (m, 1H); ^{13}C NMR (101 MHz, $CDCl_3$) δ 170.9, 166.8, 162.7, 143.7, 134.8 (q, *J* = 32.8 Hz), 132.3, 130.06, 130.08, 129.1, 127.2, 125.6 (q, *J* = 3.6 Hz), 123.6 (q, *J* = 272.8 Hz), 52.1, 49.5, 29.7, 23.3. HRMS (ESI) calcd. for $C_{20}H_{16}O_4NF_3$ $[M+Na]^+$ *m/z* 414.0929, found 414.0927.

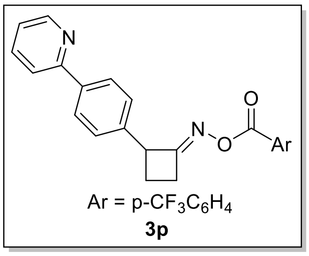

Prepared according to general procedure (B) from 4-(pyridin-2-yl)benzaldehyde to provide the title compound **3p** as a yellowish solid (14% total yield). ^{1}H NMR (400 MHz, $CDCl_3$) δ 8.67 (d, *J* = 4.6 Hz, 1H), 8.16 (d, *J* = 8.1 Hz, 2H), 7.98 (d, *J* = 8.3 Hz, 2H), 7.76 – 7.66 (m, 4H), 7.50 (d, *J* = 8.2 Hz, 2H), 7.23 – 7.17 (m, 1H), 4.79 – 4.67 (m, 1H), 3.32 – 3.09 (m, 2H), 2.73 – 2.57 (m, 1H), 2.38 – 2.24 (m, 1H). ^{13}C NMR (101 MHz, $CDCl_3$) δ 171.5, 162.8, 157.0, 149.7, 139.4, 138.3, 136.9, 134.7 (q, *J* = 32.7 Hz), 132.4, 130.1, 127.6, 127.3, 125.6 (q, *J* = 3.7 Hz), 123.6 (q, *J* = 272.7 Hz), 122.2, 120.5, 49.4, 29.7, 23.3. HRMS (ESI) calcd. for $C_{23}H_{17}O_2N_2F_3$ $[M+H]^+$ *m/z* 411.1320, found 411.1320.

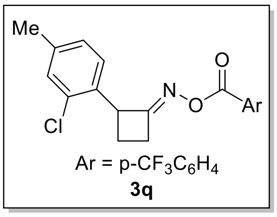

Prepared according to general procedure (B) from 2-chloro-4-methylbenzaldehyde to provide the title compound **3q** as a white solid (5% total yield). ^{1}H NMR (400 MHz, $CDCl_3$) δ 8.18 (d, *J* = 8.7 Hz, 2H), 7.72 (d, *J* = 8.6 Hz, 2H), 7.54 (d, *J* = 7.9 Hz, 1H), 7.19 (s, 1H), 7.05 (d, *J* = 7.9 Hz, 1H), 4.95 – 4.83 (m, 1H), 3.29 – 3.05 (m, 2H), 2.78 – 2.64 (m, 1H), 2.30 (s, 3H), 2.17 – 2.03 (m, 1H); ^{13}C NMR (101 MHz, $CDCl_3$) δ 171.0, 162.7, 138.8, 134.8 (q, *J* = 32.7 Hz), 133.3, 132.9, 132.4, 130.2, 130.1, 128.2, 127.9, 125.6 (q, *J* = 3.7 Hz), 123.6 (q, *J* = 272.8 Hz), 47.7, 29.4, 24.1, 20.8. HRMS (ESI) calcd. for $C_{19}H_{15}O_2NF_3Cl$ $[M+Na]^+$ *m/z* 404.0641, found 404.0634.

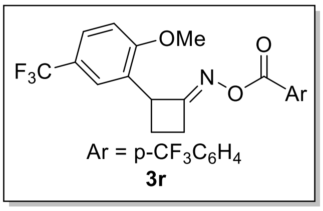

Prepared according to general procedure (B) from 2-methoxy-5-(trifluoromethyl)benzaldehyde to provide the title compound **3r** as a yellowish solid (15% total yield). ^{1}H NMR (400 MHz, $CDCl_3$) δ 8.18 (d, *J* = 8.4 Hz, 2H), 7.73 (d, *J* = 8.2 Hz, 2H), 7.66 (s, 1H), 7.52 (d, *J* = 8.6 Hz, 1H), 6.94 (d, *J* = 8.6 Hz, 1H), 4.84 – 4.72 (m, 1H), 3.90 (s, 3H), 3.35 – 3.20 (m, 1H), 3.20 – 3.06 (m, 1H), 2.67 – 2.53 (m, 1H), 2.28 – 2.14 (m, 1H); ^{13}C NMR (101 MHz, $CDCl_3$) δ 171.6, 162.8, 159.6, 134.8 (q, *J* = 32.7 Hz), 132.6, 130.2, 128.0, 126.2 (q, *J* = 3.8 Hz), 125.9 – 125.6 (m), 124.5 (q, *J* = 271.3 Hz), 123.7 (q, *J* = 272.8 Hz), 122.9 (q, *J* = 32.7 Hz), 110.7, 55.9, 45.9, 29.7, 23.2. HRMS (ESI) calcd. for $C_{20}H_{15}O_3NF_6$ $[M+Na]^+$ *m/z* 454.0854, found 454.0854.

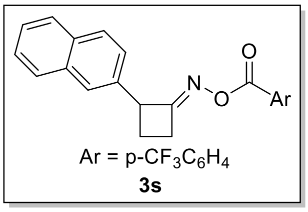

Prepared according to general procedure (B) from 2-naphthaldehyde to provide the title compound **3s** as a yellowish solid[5]. ^{1}H NMR (400 MHz, $CDCl_3$) δ 8.19 (d, *J* = 8.1 Hz, 2H), 7.84 (ddd, *J* = 12.3, 8.4, 3.6 Hz, 4H), 7.74 (d, *J* = 8.2 Hz, 2H), 7.59–7.38 (m, 3H), 4.86 (ddd, *J* = 10.0, 6.8, 2.9 Hz, 1H), 3.41–3.13 (m, 2H), 2.71 (dtd, *J* = 11.3, 9.7, 6.1 Hz, 1H), 2.40 (ddt, *J* = 11.2, 9.9, 7.1 Hz, 1H); ^{13}C NMR (101 MHz, $CDCl_3$) δ 171.4, 162.7, 135.8, 134.7 (q, *J* = 32.3 Hz), 133.3, 132.6, 132.4, 130.0, 128.6, 127.9, 127.6, 126.3, 125.9, 125.6 (q, *J* = 3.0 Hz), 125.3, 123.5 (d, *J* = 273.7 Hz), 49.8, 29.6, 23.2.

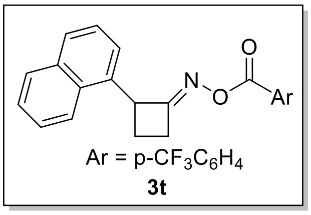

Prepared according to general procedure (B) from 1-naphthaldehyde to provide the title compound **3t** as a yellowish solid (6% total yield). ^{1}H NMR (400 MHz, $CDCl_3$) δ 8.22 (d, *J* = 8.1 Hz, 2H), 7.91 – 7.82 (m, 3H), 7.81 – 7.73 (m, 3H), 7.58 – 7.43 (m, 2H), 5.29 (t, *J* = 8.1 Hz, 1H), 3.30 – 3.21 (m, 2H), 2.91 – 2.79 (m, 1H), 2.26 – 2.12 (m, 1H); ^{13}C NMR (101 MHz, $CDCl_3$) δ 171.3, 162.8, 134.8 (q, *J* = 32.7 Hz), 134.3, 134.1, 132.4, 130.7, 130.1, 129.1, 128.0, 126.3, 125.9, 125.7 (q, *J* = 3.3 Hz), 125.6, 123.7, 123.7 (q, *J* = 272.8 Hz), 123.4, 47.7, 29.6, 24.5. HRMS (ESI) calcd. for $C_{22}H_{16}O_2NF_3$ $[M+Na]^+$ *m/z* 406.1031, found 406.1028.

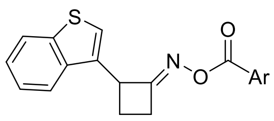

Ar = p-$CF_3C_6H_4$
3u

Prepared according to general procedure (B) from benzo[b]thiophene-3-carbaldehyde to provide the title compound **3u** as a yellowish solid (2% total yield). ^{1}H NMR (400 MHz, $CDCl_3$) δ 8.20 (d, J = 8.0 Hz, 2H), 7.91 – 7.85 (m, 1H), 7.78 – 7.68 (m, 3H), 7.65 (d, J = 1.3 Hz, 1H), 7.44 – 7.35 (m, 2H), 5.00 – 4.88 (m, 1H), 3.39 – 3.17 (m, 2H), 2.85 – 2.68 (m, 1H), 2.36 – 2.20 (m, 1H); ^{13}C NMR (101 MHz, $CDCl_3$) δ 170.8, 162.9, 141.0, 137.6, 134.9 (q, J = 32.8 Hz), 132.7, 130.7, 130.2, 125.7 (q, J = 3.7 Hz), 124.7, 124.3 , 123.7 (q, J = 272.8 Hz), 123.2, 122.9, 121.8, 44.7, 30.0, 22.8. HRMS (ESI) calcd. for $C_{20}H_{14}O_2NSF_3$ $[M+H]^+$ m/z 390.0776, found 390.0780.

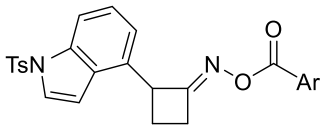

Ar = p-$CF_3C_6H_4$
3v

Prepared according to general procedure (B) from 1-tosyl-1*H*-indole-4-carbaldehyde to provide the title compound **3v** as a yellowish solid (8% total yield). ^{1}H NMR (400 MHz, $CDCl_3$) δ 8.17 (d, J = 8.1 Hz, 2H), 7.91 (d, J = 8.3 Hz, 1H), 7.80 – 7.71 (m, 4H), 7.61 (d, J = 3.7 Hz, 1H), 7.40 (dt, J = 7.5, 0.8 Hz, 1H), 7.29 (t, 1H), 7.25 – 7.21 (m, 2H), 6.69 (dd, J = 3.7, 0.8 Hz, 1H), 4.96 – 4.87 (m, 1H), 3.35 – 3.13 (m, 2H), 2.75 – 2.62 (m, 1H), 2.35 (s, 3H), 2.40 – 2.25 (m, 1H); ^{13}C NMR (101 MHz, $CDCl_3$) δ 171.2, 162.7, 145.2, 135.2, 135.0, 134.7 (q, J = 32.5 Hz),132.4, 131.3, 130.1, 130.0, 129.0, 126.9, 126.3, 125.6 (q, J = 3.7 Hz), 124.8, 123.6 (q, J = 272.8 Hz), 121.2, 112.7, 107.0, 29.8, 23.4, 21.6. HRMS (ESI) calcd. for $C_{27}H_{21}O_4N_2SF_3$ $[M+Na]^+$ m/z 549.1072, found 549.1074.

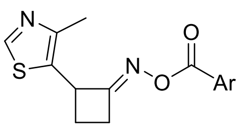

Ar = p-$CF_3C_6H_4$
3w

Prepared according to general procedure (B) from 4-methylthiazole-5-carbaldehyde to provide the title compound **3w** as a yellowish solid (9% total yield). ^{1}H NMR (400 MHz, $CDCl_3$) δ 8.60 (s, 1H), 8.12 (d, J = 8.1 Hz, 2H), 7.68 (d, J = 8.2 Hz, 2H), 4.87 – 4.76 (m, 1H), 3.35 – 3.22 (m, 1H), 3.20 – 3.07 (m, 1H), 2.77 – 2.64 (m, 1H), 2.41 (s, 3H), 2.20 – 2.09 (m, 1H); ^{13}C NMR (101 MHz, $CDCl_3$) δ 170.8, 162.5, 150.3, 149.8, 134.8 (q, J = 32.7 Hz), 132.1, 130.1, 128.9, 125.6 (q, J = 3.7 Hz), 123.5 (q, J = 272.8 Hz), 42.2, 29.8, 25.6, 15.4. HRMS (ESI) calcd. for $C_{16}H_{13}O_2N_2SF_3$ $[M+H]^+$ m/z 355.0728, found 355.0726.

General Procedure (C) for the asymmetric ring-opening cyanation of cyclopentanone oxime esters 1:

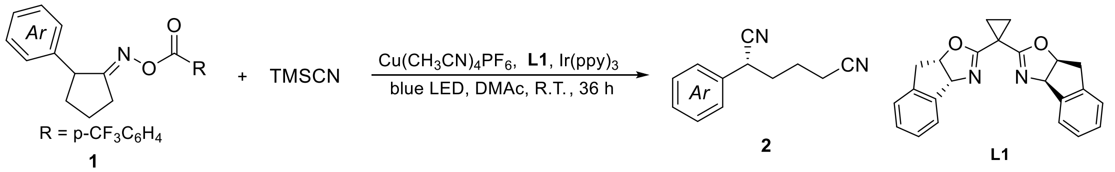

To a 25 mL Schlenk tube, $Cu(CH_3CN)_4PF_6$ (3.7 mg, 0.01 mmol), chiral bisoxazoline ligand **L1** (5.3 mg, 0.015 mmol), $Ir(ppy)_3$ (1.6 mg, 0.0025 mmol) were added in super dry DMAc (5 mL) under N_2 atmosphere. The tube was sealed with a Teflon-lined cap, then the mixture was stirred at room temperature for 0.5 h. The **catalyst solution I** was prepared firstly and used in the next step.

To a 25 mL Schlenk tube containing substrate **1**(0.1 mmol, 1.0 equiv), **catalyst solution I**(1 mL) and TMSCN (0.15 mmol, 1.5 equiv) were sequentially added under N_2 atmosphere. The tube was sealed with a Teflon-lined cap, and the mixture was stirred at a distance of ~5 cm from a 5 W blue LEDs at room temperature for 36 h .The reaction mixture was diluted with EA(10 mL). The organic layer was washed with brine (3×5 mL) and dried over anhydrous Na_2SO_4. After filtration and concentration, the residue was purified by silica gel chromatography with petroleum ether and ethyl acetate (PE/EA = 9:1~3:1) to afford the product **2**.

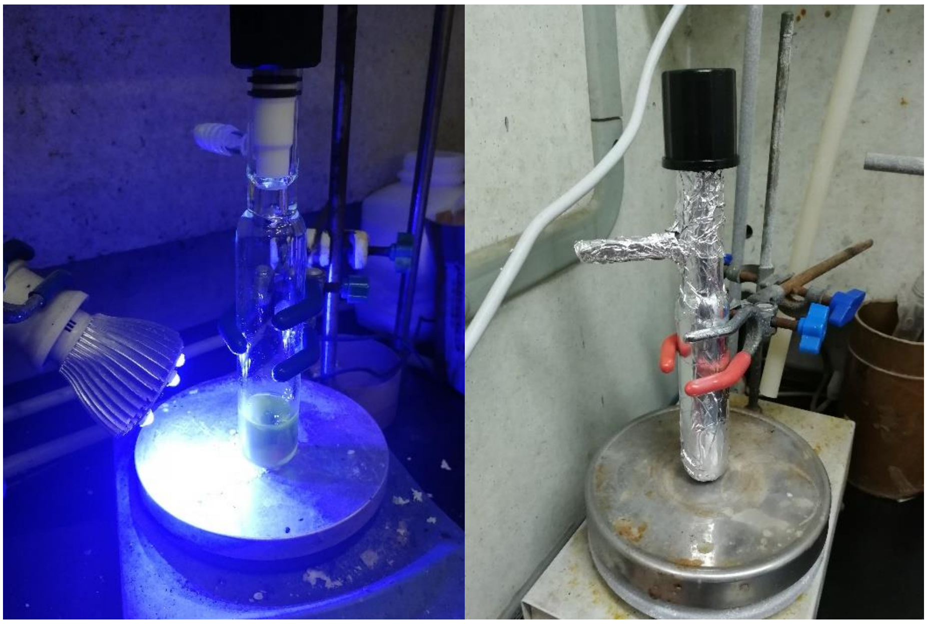

Supplementary Figure 270. photoreactions' set-up and the reaction performed in dark

Characterization data for 2-arylhexanedinitriles

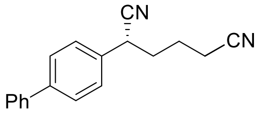

2a

Prepared according to general procedure (C) using **1a** to provide the title compound **2a** as a yellowish solid (78% yield, 92% ee; 1mmol scale: 68% yield, 90% ee). ^{1}H NMR (400 MHz, $CDCl_3$) δ 7.68 – 7.55 (m, 4H), 7.51 – 7.44 (m, 2H), 7.44 – 7.34 (m, 3H), 3.92 (t, *J* = 7.2 Hz, 1H), 2.52 – 2.33 (m, 2H), 2.19 – 2.04 (m, 2H), 2.01 – 1.77 (m, 2H); ^{13}C NMR (101 MHz, $CDCl_3$) δ 141.7, 140.2, 133.7, 129.0, 128.1, 127.9, 127.7, 127.2, 120.1, 118.8, 36.6, 34.5, 22.9, 17.0. HRMS (ESI) calcd. for $C_{18}H_{16}N_2$ $[M+Na]^+$ *m/z* 283.1211, found 283.1206.

HPLC (OD-H, 0.46*25 cm, 5 μm, hexane/isopropanol = 8/2, flow 1.0 mL/min, detection at 214 nm) retention time = 21.23 min (major) and 27.83 min (minor). $[\alpha]_D^{20.0}$ = 3.77 (c 1.0, $CHCl_3$).

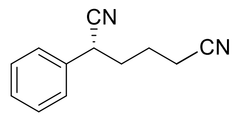

2b

Prepared according to general procedure (C) using **1b** to provide the title compound **2b** as a yellow oil (64% yield, 91% ee). ^{1}H NMR (400 MHz, $CDCl_3$) δ 7.46 – 7.31 (m, 5H), 3.87 (t, *J* = 7.2 Hz, 1H), 2.51 – 2.31 (m, 2H), 2.18 – 1.99 (m, 2H), 1.94 – 1.73 (m, 2H); ^{13}C NMR (101

MHz, $CDCl_3$) δ 134.8, 129.5, 128.6, 127.3, 120.1, 118.8, 36.9, 34.6, 22.9, 16.9. HRMS (ESI) calcd. for $C_{12}H_{12}N_2$ $[M+Na]^+$ *m/z* 207.0898, found 207.0889.

HPLC (OD-H, 0.46*25 cm, 5 μm, hexane/isopropanol = 8/2, flow 1.0 mL/min, detection at 214 nm) retention time = 18.83 min (minor) and 21.38 min (major). $[\alpha]_D^{20.0}$ = 37.0 (c 0.39, $CHCl_3$).

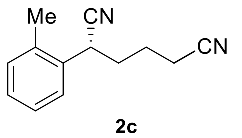

2c

Prepared according to general procedure (C) using **1c** to provide the title compound **2c** as a yellow oil (81% yield, 90% ee). ^{1}H NMR (400 MHz, $CDCl_3$) δ 7.46 – 7.39 (m, 1H), 7.31 – 7.18 (m, 3H), 4.07 – 3.97 (m, 1H), 2.47 – 2.39 (m, 2H), 2.36 (s, 3H), 2.09 – 1.77 (m, 4H); ^{13}C NMR (101 MHz, $CDCl_3$) δ 135.0, 133.2, 131.4, 128.6, 127.5, 127.2, 120.4, 118.8, 33.7, 33.1, 23.1, 19.3, 16.9. HRMS (ESI) calcd. for $C_{13}H_{14}N_2$ $[M+Na]^+$ *m/z* 221.1055, found 221.1050.

HPLC (OD-H, 0.46*25 cm, 5 μm, hexane/isopropanol = 8/2, flow 1.0 mL/min, detection at 214 nm) retention time = 20.60 min (minor) and 29.09 min (major). $[\alpha]_D^{20.0}$ = 49.0 (c 0.99, $CHCl_3$).

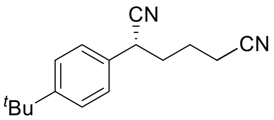

2d

Prepared according to general procedure (C) using **1d** to provide the title compound **2d** as a yellowish oil (91% yield, 88% ee). ^{1}H NMR (400 MHz, $CDCl_3$) δ 7.46 – 7.37 (m, 2H), 7.30 – 7.23 (m, 2H), 3.84 (t, *J* = 7.1 Hz, 1H), 2.46 – 2.37 (m, 2H), 2.13 – 2.01 (m, 2H), 1.94 – 1.73 (m, 2H), 1.32 (s, 9H); ^{13}C NMR (101 MHz, $CDCl_3$) δ 151.7, 131.7, 127.0, 126.4, 120.3, 118.8, 36.4, 34.7, 34.5, 31.4, 22.9, 16.9. HRMS (ESI) calcd. for $C_{16}H_{20}N_2$ $[M+Na]^+$ *m/z* 263.1524, found 263.1522.

HPLC (OD-H, 0.46*25 cm, 5 μm, hexane/isopropanol = 9/1, flow 1.0 mL/min, detection at 214 nm) retention time = 16.39 min (minor) and 17.83 min (major). $[\alpha]_D^{20.0}$ = 9.95 (c 1.24, $CHCl_3$).

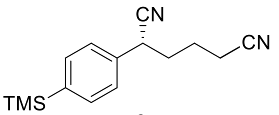

2e

Prepared according to general procedure (C) using **1e** to provide the title compound **2e** as a yellow oil (70% yield, 90% ee). ^{1}H NMR (400 MHz, $CDCl_3$) δ 7.55 (d, *J* = 8.1 Hz, 2H), 7.32 (d, *J* = 7.9 Hz, 2H), 3.86 (t, *J* = 7.1 Hz, 1H), 2.51 – 2.32 (m, 2H), 2.14 – 2.00 (m, 2H), 1.95 – 1.73 (m, 2H), 0.28 (s, 9H); ^{13}C NMR (101 MHz, $CDCl_3$) δ 141.3, 135.1,

134.4, 126.6, 120.0, 118.8, 36.8, 34.5, 22.9, 16.9, -1.1. HRMS (ESI) calcd. for $C_{15}H_{20}N_2Si$ [M+Na]$^+$ *m/z* 279.1293, found 279.1292.

HPLC (AS-H, 0.46*25 cm, 5 μm, hexane/isopropanol = 19/1, flow 1.0 mL/min, detection at 214 nm) retention time = 18.20 min (major) and 22.01 min (minor). $[\alpha]_D^{20.0}$ = 7.66 (c 1.34, $CHCl_3$).

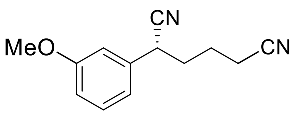

2f

Prepared according to general procedure (C) using **1f** to provide the title compound **2f** as a yellow oil (52% yield, 90% ee). ^{1}H NMR (400 MHz, $CDCl_3$) δ 7.32 (t, *J* = 7.9 Hz, 1H), 6.94 – 6.85 (m, 3H), 3.84 (t, *J* = 7.1 Hz, 1H), 3.83 (s, 3H), 2.50 – 2.33 (m, 2H), 2.16 – 2.01 (m, 2H), 1.93 – 1.75 (m, 2H); ^{13}C NMR (101 MHz, $CDCl_3$) δ 160.3, 136.2, 130.5, 120.0, 119.5, 118.8, 113.9, 113.2, 55.5, 36.8, 34.4, 22.8, 16.9. HRMS (ESI) calcd. for $C_{13}H_{14}N_2O$ [M+Na]$^+$ *m/z* 237.1004, found 237.0998.

HPLC (OD-H, 0.46*25 cm, 5 μm, hexane/isopropanol = 8/2, flow 1.0 mL/min, detection at 214 nm) retention time = 30.34 min (minor) and 36.25 min (major). $[\alpha]_D^{20.0}$ = 18.0 (c 0.43, $CHCl_3$).

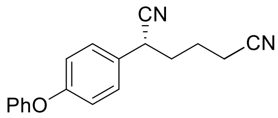

2g

Prepared according to general procedure (C) using **1g** to provide the title compound **2g** as a yellow oil (63% yield, 84% ee). ^{1}H NMR (400 MHz, $CDCl_3$) δ 7.41 – 7.34 (m, 2H), 7.31 – 7.25 (m, 3H), 7.18 – 7.12 (m, 1H), 7.07 – 6.99 (m, 4H), 3.85 (t, *J* = 7.2 Hz, 1H), 2.50 – 2.34 (m, 2H), 2.14 – 2.01 (m, 2H), 1.97 – 1.75 (m, 2H); ^{13}C NMR (101 MHz, $CDCl_3$) δ 157.8, 156.6, 130.0, 129.2, 128.7, 124.0, 120.1, 119.5, 119.3, 118.8, 36.2, 34.6, 22.9, 16.9. HRMS (ESI) calcd. for $C_{18}H_{16}N_2O$ [M+Na]$^+$ *m/z* 299.1160, found 299.1158.

HPLC (OD-H, 0.46*25 cm, 5 μm, hexane/isopropanol = 8/2, flow 1.0 mL/min, detection at 214 nm) retention time = 19.77 min (major) and 25.61 min (minor). $[\alpha]_D^{20.0}$ = -3.41 (c 0.99, $CHCl_3$).

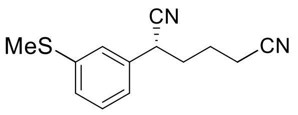

2h

Prepared according to general procedure (C) using **1h** to provide the title compound **2h** as a yellow oil (81% yield, 90% ee). ^{1}H NMR (400 MHz, $CDCl_3$) δ 7.36 – 7.26 (m, 1H), 7.26 – 7.16 (m, 2H), 7.09 (dt, *J* = 7.7, 1.4 Hz, 1H), 3.83 (t, *J* = 7.2 Hz, 1H), 2.50 (s, 3H), 2.47 – 2.36 (m, 2H), 2.14 – 2.00 (m, 2H), 1.95 – 1.73 (m, 2H); ^{13}C NMR (101 MHz,

$CDCl_3$) δ 140.4, 135.5, 129.8, 126.3, 125.0, 123.8, 119.8, 118.7, 36.8, 34.4, 22.9, 16.9, 15.7. HRMS (ESI) calcd. for $C_{13}H_{14}N_2S$ $[M+Na]^+$ *m/z* 253.0775, found 253.0775.

HPLC (OD-H, 0.46*25 cm, 5 μm, hexane/isopropanol = 8/2, flow 1.0 mL/min, detection at 214 nm) retention time = 25.41 min (minor) and 27.72 min (major). $[\alpha]_D^{20.0}$ = 22.7 (c 1.04, $CHCl_3$).

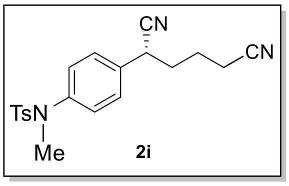

Prepared according to general procedure (C) using **1i** to provide the title compound **2i** as a yellowish oil (79% yield, 90% ee). ^{1}H NMR (400 MHz, $CDCl_3$) δ 7.42 (d, *J* = 8.3 Hz, 2H), 7.31 – 7.23 (m, 4H), 7.14 (d, *J* = 8.5 Hz, 2H), 3.87 (t, *J* = 7.2 Hz, 1H), 3.15 (s, 3H), 2.49 – 2.36 (m, 5H), 2.12 – 1.98 (m, 2H), 1.94 – 1.74 (m, 2H); ^{13}C NMR (101 MHz, $CDCl_3$) δ 144.0, 142.0, 133.6, 133.3, 129.6, 127.9, 127.8, 127.3, 119.8, 118.8, 38.0, 36.4, 34.4, 22.9, 21.7, 16.9. HRMS (ESI) calcd. for $C_{20}H_{21}N_3O_2S$ $[M+Na]^+$ *m/z* 390.1252, found 390.1253.

HPLC (AD-H, 0.46*25 cm, 5 μm, hexane/isopropanol = 85/15, flow 0.5 mL/min, detection at 214 nm) retention time = 88.43 min (minor) and 93.25 min (major). $[\alpha]_D^{20.0}$ = 4.15 (c 1.37, $CHCl_3$).

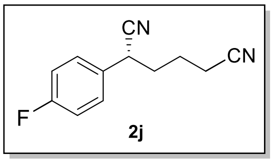

Prepared according to general procedure (C), with modifications of 1.5 mol% $Cu(CH_3CN)_4PF_6$ and 2.25 mol% **L1**, using **1j** to provide the title compound **2j** as a yellow oil (71% yield, 87% ee). ^{1}H NMR (400 MHz, $CDCl_3$) δ 7.36 – 7.29 (m, 2H), 7.15 – 7.06 (m, 2H), 3.86 (t, *J* = 7.2 Hz, 1H), 2.50 – 2.34 (m, 2H), 2.12 – 1.99 (m, 2H), 1.97 – 1.73 (m, 2H); ^{13}C NMR (101 MHz, $CDCl_3$) δ 162.7 (d, *J* = 248.3 Hz), 130.6 (d, *J* = 3.4 Hz), 129.0 (d, *J* = 8.4 Hz), 119.9 , 118.7 , 116.5 (d, *J* = 21.9 Hz), 36.2 , 34.6 , 22.8, 16.9. HRMS (ESI) calcd. for $C_{12}H_{11}N_2F$ $[M+Na]^+$ *m/z* 225.0804, found 225.0806.

HPLC (OD-H, 0.46*25 cm, 5 μm, hexane/isopropanol = 9/1, flow 1.0 mL/min, detection at 214 nm) retention time = 28.40 min (major) and 32.39 min (minor). $[\alpha]_D^{20.0}$ = 18.5 (c 1.49, $CHCl_3$).

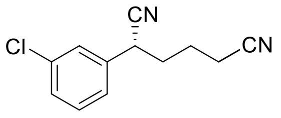

2k

Prepared according to general procedure (C) using **1k** to provide the title compound **2k** as a yellow oil (64% yield, 91% ee). ^{1}H NMR (400 MHz, $CDCl_3$) δ 7.40 – 7.31 (m, 3H), 7.26 – 7.20 (m, 1H), 3.86 (t, *J* = 7.2 Hz, 1H), 2.53 – 2.34 (m, 2H), 2.14 – 1.99 (m, 2H), 1.95 – 1.73 (m, 2H); ^{13}C NMR (101 MHz, $CDCl_3$) δ 136.7, 135.4, 130.8, 129.0, 127.5, 125.5, 119.4, 118.7, 36.6, 34.4, 22.8, 16.9. HRMS (ESI) calcd. for $C_{12}H_{11}N_2Cl$ $[M+Na]^+$ *m/z* 241.0508, found 241.0503.

HPLC (OD-H, 0.46*25 cm, 5 μm, hexane/isopropanol = 8/2, flow 1.0 mL/min, detection at 214 nm) retention time = 15.20 min (minor) and 18.67 min (major). $[\alpha]_D^{20.0}$ = 22.0 (c 0.81, $CHCl_3$).

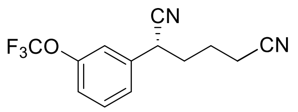

2l

Prepared according to general procedure (C), with modifications of 1.5 mol% $Cu(CH_3CN)_4PF_6$ and 2.25 mol% **L1**, using **1l** to provide the title compound **2l** as a yellow oil (79% yield, 90% ee). ^{1}H NMR (400 MHz, $CDCl_3$) δ 7.46 (t, *J* = 8.0 Hz, 1H), 7.31 (d, *J* = 7.8 Hz, 1H), 7.26 – 7.18 (m, 2H), 3.91 (t, *J* = 7.2 Hz, 1H), 2.55 – 2.36 (m, 2H), 2.17 – 2.01 (m, 2H), 1.98 – 1.76 (m, 2H); ^{13}C NMR (101 MHz, $CDCl_3$) δ 149.9, 137.0, 131.0, 125.7, 121.1, 120.5 (q, *J* = 258.0 Hz), 120.0, 119.3, 118.6, 36.6, 34.4, 22.8, 16.9. HRMS (ESI) calcd. for $C_{13}H_{11}N_2OF_3$ $[M+Na]^+$ *m/z* 291.0721, found 291.0720.

HPLC (OD-H, 0.46*25 cm, 5 μm, hexane/isopropanol = 92/8, flow 1.0 mL/min, detection at 214 nm) retention time = 26.14 min (minor) and 28.11 min (major). $[\alpha]_D^{20.0}$ = 18.8 (c 0.66, $CHCl_3$).

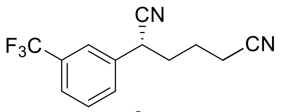

2m

Prepared according to general procedure (C) using **1m** to provide the title compound **2m** as a yellow oil (72% yield, 90% ee). ^{1}H NMR (400 MHz, $CDCl_3$) δ 7.70 – 7.52 (m, 4H), 3.95 (t, *J* = 7.3 Hz, 1H), 2.59 – 2.36 (m, 2H), 2.16 – 2.03 (m, 2H), 1.99 – 1.77 (m, 2H); ^{13}C NMR (101 MHz, $CDCl_3$) δ 135.9 , 132.0 (q, *J* = 32.8 Hz), 130.7, 130.2, 125.7 (q, *J* = 3.7 Hz), 124.1 (q, *J* = 3.7 Hz), 123.7 (q, *J* = 272.5 Hz), 119.3, 118.6, 36.8, 34.5, 22.9, 16.9. HRMS (ESI) calcd. for $C_{13}H_{11}N_2F_3$ $[M+Na]^+$ *m/z* 275.0772, found 275.0765.

HPLC (OD-H, 0.46*25 cm, 5 μm, hexane/isopropanol = 9/1, flow 1.0 mL/min, detection at 214 nm) retention time = 22.71 min (minor) and 24.10 min (major). $[\alpha]_D^{20.0}$ = 19.9 (c 1.12, $CHCl_3$).

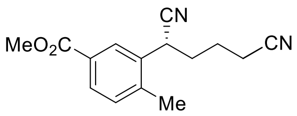

2n

Prepared according to general procedure (C) using **1n** to provide the title compound **2n** as a yellowish oil (79% yield, 89% ee). ^1H NMR (400 MHz, $CDCl_3$) δ 8.08 (d, *J* = 1.6 Hz, 1H), 7.92 (dd, *J* = 7.9, 1.7 Hz, 1H), 7.30 (d, *J* = 8.0 Hz, 1H), 4.04 (dd, *J* = 8.8, 5.6 Hz, 1H), 3.93 (s, 3H), 2.55 – 2.38 (m, 5H), 2.17 – 1.80 (m, 4H); ^{13}C NMR (101 MHz, $CDCl_3$) δ 166.4, 140.5, 133.6, 131.6, 129.8, 129.3, 128.6, 119.8, 118.7, 52.4, 33.7, 32.9, 23.2, 19.6, 16.9. HRMS (ESI) calcd. for $C_{15}H_{16}N_2O_2$ $[M+Na]^+$ *m/z* 279.1109, found 279.1107.

HPLC (ID, 0.46*25 cm, 5 μm, hexane/isopropanol = 8/2, flow 1.0 mL/min, detection at 214 nm) retention time = 37.33 min (major) and 42.41 min (minor). $[\alpha]_D^{20.0}$ = 45.7 (c 0.94, $CHCl_3$).

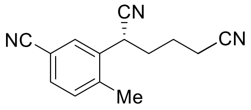

2o

Prepared according to general procedure (C) using **1o** to provide the title compound **2o** as a yellow solid (85% yield, 89% ee). ^1H NMR (400 MHz, $CDCl_3$) δ 7.72 (d, *J* = 1.5 Hz, 1H), 7.56 (dd, *J* = 7.9, 1.6 Hz, 1H), 7.35 (d, *J* = 7.9 Hz, 1H), 4.04 (dd, *J* = 8.3, 6.0 Hz, 1H), 2.58 – 2.39 (m, 5H), 2.13 – 1.82 (m, 4H); ^{13}C NMR (101 MHz, $CDCl_3$) δ 140.9, 134.9, 132.4, 132.2, 131.1, 119.2, 118.5, 118.2, 111.4, 33.7, 32.8, 23.1, 19.7, 16.9. HRMS (ESI) calcd. for $C_{14}H_{13}N_3$ $[M+Na]^+$ *m/z* 246.1007, found 246.1003.

HPLC (ID, 0.46*25 cm, 5 μm, hexane/isopropanol = 8/2, flow 1.0 mL/min, detection at 214 nm) retention time = 30.24 min (major) and 35.87 min (minor). $[\alpha]_D^{20.0}$ = 40.7 (c 0.91, $CHCl_3$).

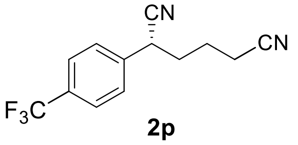

2p

Prepared according to general procedure (C) using **1p** to provide the title compound **2p** as a yellow oil (50% yield, 85% ee). ^1H NMR (400 MHz, Chloroform-*d*) δ 7.69 (d, *J* = 8.1 Hz, 2H), 7.49 (d, *J* = 8.1 Hz, 2H), 3.96 (t, *J* = 7.2 Hz, 1H), 2.55 – 2.36 (m, 2H), 2.18 – 2.01 (m, 2H), 1.98 – 1.76 (m, 2H); ^{13}C NMR (101 MHz, Chloroform-*d*) δ 138.8, 131.1 (q, *J* = 32.8 Hz), 127.8, 126.5 (q, *J* = 3.7 Hz), 123.8 (q, *J* = 270.7 Hz), 119.3, 118.6,

36.8, 34.4, 22.8, 16.9. HRMS (ESI) calcd. for $C_{13}H_{11}N_2F_3$ $[M+H]^+$ *m/z* 253.0953, found 253.0946.

HPLC (OD-H, 0.46*25 cm, 5 μm, hexane/isopropanol = 95/5, flow 1.0 mL/min, detection at 214 nm) retention time = 51.07 min (minor) and 53.43 min (major). $[\alpha]_D^{20.0}$ = 10.1 (c 0.73, $CHCl_3$).

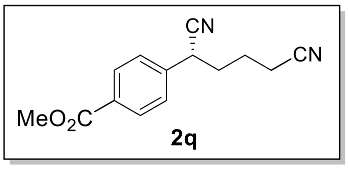

Prepared according to general procedure (C) using **1q** to provide the title compound **2q** as a yellowish oil (74% yield, 76% ee). ^1H NMR (400 MHz, Chloroform-*d*) δ 8.08 (d, *J* = 8.4 Hz, 2H), 7.43 (d, *J* = 8.3 Hz, 2H), 4.03 – 3.87 (m, 4H), 2.52 – 2.34 (m, 2H), 2.17 – 2.02 (m, 2H), 1.96 – 1.74 (m, 2H); ^{13}C NMR (101 MHz, $CDCl_3$) δ 166.4, 139.6, 130.7, 130.6, 127.4, 119.4, 118.6, 52.5, 36.9, 34.3, 22.8, 16.9. HRMS (ESI) calcd. for $C_{14}H_{14}N_2O_2$ $[M+H]^+$ *m/z* 243.1134, found 243.1124.

HPLC (OD-H, 0.46*25 cm, 5 μm, hexane/isopropanol = 8/2, flow 1.0 mL/min, detection at 214 nm) retention time = 21.47 min (major) and 24.00 min (minor). $[\alpha]_D^{20.0}$ = 7.2 (c 1.17, $CHCl_3$).

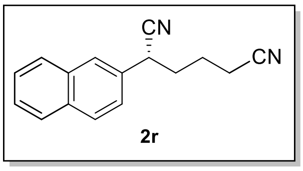

Prepared according to general procedure (C) using **1r** to provide the title compound **2r** as a yellowish solid (79% yield, 90% ee). ^1H NMR (400 MHz, $CDCl_3$) δ 7.95 – 7.81 (m, 4H), 7.60 – 7.50 (m, 2H), 7.40 (dd, *J* = 8.5, 1.9 Hz, 1H), 4.04 (t, *J* = 7.1 Hz, 1H), 2.55 – 2.29 (m, 2H), 2.23 – 2.08 (m, 2H), 1.97 – 1.74 (m, 2H); ^{13}C NMR (101 MHz, $CDCl_3$) δ 133.4, 133.1, 132.0, 129.6, 128.0, 127.9, 127.1, 126.9, 126.5, 124.5, 120.1, 118.8, 37.0, 34.4, 22.9, 17.0. HRMS (ESI) calcd. for $C_{16}H_{14}N_2$ $[M+Na]^+$ *m/z* 257.1055, found 257.1054.

HPLC (AD-H, 0.46*25 cm, 5 μm, hexane/isopropanol = 19/1, flow 1.0 mL/min, detection at 214 nm) retention time = 31.36 min (minor) and 33.61 min (major). $[\alpha]_D^{20.0}$ = 11.7 (c 1.08, $CHCl_3$).

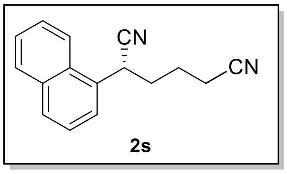

Prepared according to general procedure (C) using **1s** to provide the title compound **2s** as a yellow oil (89% yield, 93% ee). ^1H NMR (400 MHz, $CDCl_3$) δ 7.98 – 7.83 (m, 3H), 7.69 (d, *J* = 6.8 Hz, 1H), 7.65 – 7.47 (m, 3H), 4.64 (dd, *J* = 8.3, 5.5 Hz, 1H), 2.51 – 2.32 (m,

2H), 2.30 – 2.12 (m, 2H), 2.01 – 1.87 (m, 2H); ^{13}C NMR (101 MHz, $CDCl_3$) δ 134.2, 130.4, 129.9, 129.6, 129.5, 127.4, 126.5, 125.8, 125.6, 121.9, 120.4, 118.8, 33.9, 33.3, 23.2, 16.9. HRMS (ESI) calcd. for $C_{16}H_{14}N_2$ $[M+Na]^+$ *m/z* 257.1055, found 257.1050. HPLC (AD-H, 0.46*25 cm, 5 μm, hexane/isopropanol = 9/1, flow 1.0 mL/min, detection at 214 nm) retention time = 17.89 min (minor) and 21.75 min (major). $[\alpha]_D^{20.0}$ = 98.3 (c 1.01, $CHCl_3$).

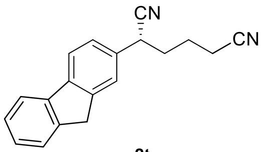

2t

Prepared according to general procedure (C) using **1t** to provide the title compound **2t** as a yellowish solid (88% yield, 90% ee). ^{1}H NMR (400 MHz, $CDCl_3$) δ 7.85 – 7.76 (m, 2H), 7.61 – 7.50 (m, 2H), 7.40 (t, *J* = 7.1 Hz, 1H), 7.37 – 7.29 (m, 2H), 4.00 – 3.87 (m, 3H), 2.49 – 2.34 (m, 2H), 2.17 – 2.06 (m, 2H), 1.97 – 1.77 (m, 2H); ^{13}C NMR (101 MHz, $CDCl_3$) δ 144.5, 143.4, 142.2, 140.8, 133.0, 127.4, 127.1, 126.0, 125.3, 124.0, 120.6, 120.3, 120.2, 118.8, 36.98, 36.96, 34.8, 22.9, 16.9. HRMS (ESI) calcd. for $C_{19}H_{16}N_2$ $[M+Na]^+$ *m/z* 295.1211, found 295.1205.

HPLC (OD-H, 0.46*25 cm, 5 μm, hexane/isopropanol = 8/2, flow 1.0 mL/min, detection at 214 nm) retention time = 23.48 min (major) and 28.48 min (minor). $[\alpha]_D^{20.0}$ = 11.4 (c 1.17, $CHCl_3$).

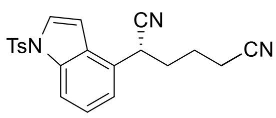

2u

Prepared according to general procedure (C) using **1u** to provide the title compound **2u** as a yellowish oil (84% yield, 89% ee). ^{1}H NMR (400 MHz, $CDCl_3$) δ 7.99 (d, *J* = 8.3 Hz, 1H), 7.78 (d, *J* = 8.4 Hz, 2H), 7.67 (d, *J* = 3.8 Hz, 1H), 7.37 – 7.28 (m, 1H), 7.29 – 7.21 (m, 3H), 6.75 (dd, *J* = 3.8, 0.9 Hz, 1H), 4.11 (t, *J* = 7.4 Hz, 1H), 2.47 – 2.29 (m, 5H), 2.20 – 2.04 (m, 2H), 1.94 – 1.70 (m, 2H); ^{13}C NMR (101 MHz, $CDCl_3$) δ 145.5, 135.2, 135.1, 130.2, 128.4, 127.4, 127.2, 127.0, 125.1, 122.2, 119.7, 118.7, 113.9, 105.6, 34.7, 33.3, 23.0, 21.7, 16.8. HRMS (ESI) calcd. for $C_{21}H_{19}N_3O_2S$ $[M+Na]^+$ *m/z* 400.1096, found 400.1096.

HPLC (OD-H, 0.46*25 cm, 5 μm, hexane/isopropanol = 8/2, flow 1.0 mL/min, detection at 214 nm) retention time = 39.69 min (minor) and 43.44 min (major). $[\alpha]_D^{20.0}$ = 9.13 (c 1.14, $CHCl_3$).

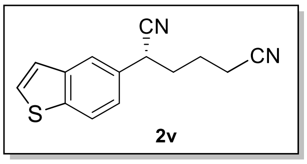

Prepared according to general procedure (C) using **1v** to provide the title compound **2v** as a yellow solid (67% yield, 80% ee). ^{1}H NMR (400 MHz, $CDCl_3$) δ 7.91 (d, *J* = 8.3 Hz, 1H), 7.82 (d, *J* = 1.5 Hz, 1H), 7.54 (d, *J* = 5.4 Hz, 1H), 7.36 (d, *J* = 5.4 Hz, 1H), 7.29 (dd, *J* = 8.4, 1.7 Hz, 1H), 4.01 (t, *J* = 7.1 Hz, 1H), 2.51 – 2.34 (m, 2H), 2.21 – 2.08 (m, 2H), 1.98 – 1.77 (m, 2H); ^{13}C NMR (101 MHz, $CDCl_3$) δ 140.2, 139.9, 130.9, 128.3, 123.8, 123.6, 123.1, 122.4, 120.2, 118.8, 36.9, 34.8, 22.8, 16.9. HRMS (ESI) calcd. for $C_{14}H_{12}N_2S$ $[M+Na]^+$ *m/z* 263.0619, found 263.0618.

HPLC (OD-H, 0.46*25 cm, 5 μm, hexane/isopropanol = 8/2, flow 1.0 mL/min, detection at 214 nm) retention time = 35.62 min (minor) and 38.44 min (major). $[\alpha]_D^{20.0}$ = 15.8 (c 0.96, $CHCl_3$).

General procedure (D) for the asymmetric ring-opening cyanation of cyclobutanone oxime esters 3:

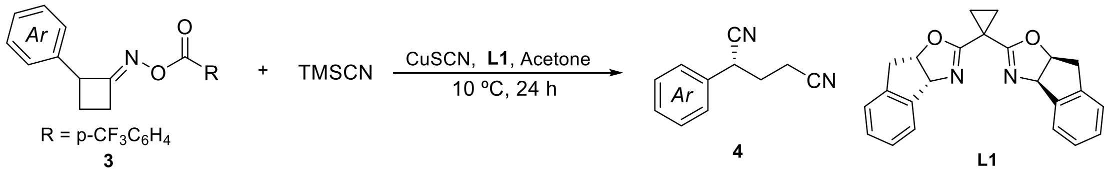

To a 5 mL Schlenk tube, CuSCN (1.8 mg, 0.015 mmol), chiral bisoxazoline ligand **L1** (6.4 mg, 0.018 mmol) were added in degassed Acetone (1 mL) under N_2 atmosphere. The tube was sealed with a Teflon-lined cap, then the mixture was stirred at room temperature for 0.5 h. The **catalyst solution II** was prepared firstly and used in the next step.

To a 25 mL Schlenk tube containing substrate **3**(0.1 mmol, 1.0 equiv), 0.8 mL Acetone, **catalyst solution II**(200 uL) and TMSCN (0.15 mmol, 1.5 equiv) were sequentially added under N_2 atmosphere. The tube was sealed with a Teflon-lined cap, and the mixture was stirred at 10 ºC for 24 h . Then solvent was removed under vacuum

and the residue was purified by silica gel chromatography with petroleum ether and ethyl acetate (PE/EA = 10:1~5:1) to afford the product **4**.

Characterization data for 2-arylpentanedinitriles

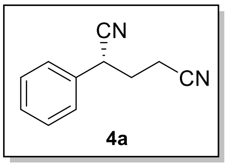

Prepared according to general procedure (D) using **3a** to provide the title compound **4a** as a colorless oil (73% yield, 94% ee). ^{1}H NMR (400 MHz, $CDCl_3$) δ 7.48 – 7.32 (m, 5H), 3.99 (t, *J* = 7.5 Hz, 1H), 2.64 – 2.49 (m, 1H), 2.49 – 2.16 (m, 3H); ^{13}C NMR (101 MHz, $CDCl_3$) δ 133.5, 129.7, 129.1, 127.4, 119.3, 117.9, 36.2, 31.5, 15.1. HRMS (ESI) calcd. for $C_{11}H_{10}N_2$ $[M+H]^+$ *m/z* 171.0922, found 171.0926.

HPLC (OD-H, 0.46*25 cm, 5 μm, hexane/isopropanol = 86/14, flow 1.0 mL/min, detection at 214 nm) retention time = 19.77 min (minor) and 28.07 min (major). $[\alpha]_D^{20.0}$ = -18.7 (c 0.52, $CHCl_3$).

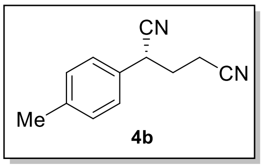

Prepared according to general procedure (D) using **3b** to provide the title compound **4b** as a yellowish oil (64% yield, 89% ee). ^{1}H NMR (400 MHz, $CDCl_3$) δ 7.23 (s, 4H), 3.94 (t, *J* = 7.6 Hz, 1H), 2.59 – 2.47 (m, 1H), 2.46 – 2.33 (m, 1H), 2.37 (s, 3H), 2.33 – 2.15 (m, 2H); ^{13}C NMR (101 MHz, $CDCl_3$) δ 139.1, 130.5, 130.3, 127.3, 119.5, 117.9, 35.8, 31.5, 21.2, 15.1. HRMS (ESI) calcd. for $C_{12}H_{12}N_2$ $[M+Na]^+$ *m/z* 207.0898, found 207.0891.

HPLC (OD-H, 0.46*25 cm, 5 μm, hexane/isopropanol = 86/14, flow 1.0 mL/min, detection at 214 nm) retention time = 17.97 min (minor) and 20.59 min (major). $[\alpha]_D^{20.0}$ = -20.0 (c 0.36, $CHCl_3$).

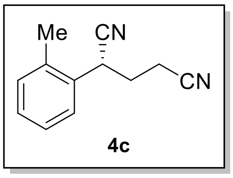

Prepared according to general procedure (D) using **3c** to provide the title compound **4c** as a yellowish oil (95% yield, 93% ee). ^{1}H NMR (400 MHz, $CDCl_3$) δ 7.43 – 7.37 (m, 1H), 7.32 – 7.20 (m, 3H), 4.16 (dd, *J* = 8.8, 6.2 Hz, 1H), 2.67 – 2.47 (m, 2H), 2.39 (s, 3H), 2.29 – 2.14 (m, 2H); ^{13}C NMR (101 MHz, $CDCl_3$) δ 135.3, 131.9, 131.6, 129.1, 127.5, 127.3, 119.5, 118.0, 33.1, 30.2, 19.2, 15.4. HRMS (ESI) calcd. for $C_{12}H_{12}N_2$ $[M+Na]^+$ *m/z* 207.0898, found 207.0893.

HPLC (OD-H, 0.46*25 cm, 5 μm, hexane/isopropanol = 8/2, flow 1.0 mL/min,

detection at 214 nm) retention time = 16.98 min (minor) and 31.80 min (major). $[\alpha]_D^{20.0}$ = 14.3 (c 0.66, $CHCl_3$).

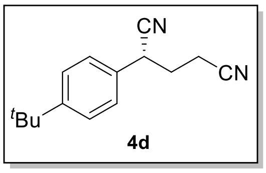

Prepared according to general procedure (D) using **3d** to provide the title compound **4d** as a yellowish oil (89% yield, 89% ee). ^{1}H NMR (400 MHz, $CDCl_3$) δ 7.44 (d, *J* = 8.4 Hz, 2H), 7.27 (d, *J* = 8.2 Hz, 2H), 3.95 (t, *J* = 7.4 Hz, 1H), 2.60 – 2.49 (m, 1H), 2.47 – 2.36 (m, 1H), 2.35 – 2.17 (m, 2H), 1.32 (s, 9H); ^{13}C NMR (101 MHz, $CDCl_3$) δ 152.3, 130.4, 127.1, 126.6, 119.5, 118.0, 35.7, 34.8, 31.4, 31.3, 15.1. HRMS (ESI) calcd. for $C_{15}H_{18}N_2$ $[M+Na]^+$ *m/z* 249.1368, found 249.1359.

HPLC (OD-H, 0.46*25 cm, 5 μm, hexane/isopropanol = 86/14, flow 1.0 mL/min, detection at 214 nm) retention time = 11.31 min (minor) and 13.75 min (major). $[\alpha]_D^{20.0}$ = -25.9 (c 0.93, $CHCl_3$).

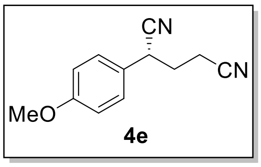

Prepared according to general procedure (D) using **3e** to provide the title compound **4e** as a yellowish oil (62% yield, 90% ee). ^{1}H NMR (400 MHz, $CDCl_3$) δ 7.26 (d, *J* = 8.6 Hz, 2H), 6.94 (d, *J* = 8.7 Hz, 2H), 3.93 (t, *J* = 7.5 Hz, 1H), 3.82 (s, 3H), 2.61 – 2.47 (m, 1H), 2.47 – 2.34 (m, 1H), 2.35 – 2.13 (m, 2H); ^{13}C NMR (101 MHz, $CDCl_3$) δ 160.1, 128.6, 125.3, 119.6, 118.0, 115.0, 55.5, 35.4, 31.5, 15.0. HRMS (ESI) calcd. for $C_{12}H_{12}N_2O$ $[M+Na]^+$ *m/z* 223.0847, found 223.0853.

HPLC (OD-H, 0.46*25 cm, 5 μm, hexane/isopropanol = 8/2, flow 1.0 mL/min, detection at 214 nm) retention time = 18.22 min (minor) and 20.05 min (major). $[\alpha]_D^{20.0}$ = -31.0 (c 0.56, $CHCl_3$).

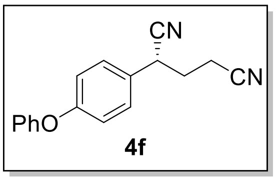

Prepared according to general procedure (D) using **3f** to provide the title compound **4f** as a yellowish oil (82% yield, 91% ee). ^{1}H NMR (400 MHz, $CDCl_3$) δ 7.41 – 7.34 (m, 2H), 7.33 – 7.27 (m, 2H), 7.20 – 7.13 (m, 1H), 7.07 – 7.00 (m, 4H), 3.97 (t, *J* = 7.5 Hz, 1H), 2.62 – 2.51 (m, 1H), 2.50 – 2.39 (m, 1H), 2.37 – 2.15 (m, 2H); ^{13}C NMR (101 MHz, $CDCl_3$) δ 158.3, 156.4, 130.1, 128.9, 127.8, 124.2, 119.6, 119.4, 117.9, 35.5, 31.5, 15.1. HRMS (ESI) calcd. for $C_{17}H_{14}N_2O$ $[M+Na]^+$ *m/z* 285.1004, found 285.0998.

HPLC (AD-H, 0.46*25 cm, 5 μm, hexane/isopropanol = 97/3, flow 1.0 mL/min,

detection at 214 nm) retention time = 35.02 min (minor) and 36.89 min (major). $[\alpha]_D^{20.0}$ = -40.7 (c 0.97, $CHCl_3$).

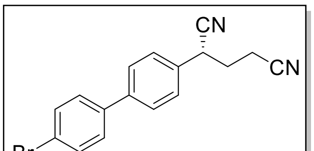

4g

Prepared according to general procedure (D) using **3g** to provide the title compound **4g** as a yellowish solid (71% yield, 96% ee). ^{1}H NMR (400 MHz, $CDCl_3$) δ 7.64 – 7.56 (m, 4H), 7.47 – 7.40 (m, 4H), 4.04 (t, J = 8.8, 1H), 2.65 – 2.54 (m, 1H), 2.52 – 2.41 (m, 1H), 2.40 – 2.21 (m, 2H); ^{13}C NMR (101 MHz, $CDCl_3$) δ 141.0, 138.9, 132.8, 132.2, 128.8, 128.2, 128.0, 122.4, 119.2, 117.8, 35.9, 31.4, 15.2. HRMS (ESI) calcd. for $C_{17}H_{13}N_2Br$ $[M+Na]^+$ *m/z* 347.0160, found 347.0157.

HPLC (OD-H, 0.46*25 cm, 5 μm, hexane/isopropanol = 8/2, flow 1.0 mL/min, detection at 214 nm) retention time = 19.31 min (minor) and 20.85 min (major). $[\alpha]_D^{20.0}$ = -38.5 (c 1.0, $CHCl_3$).

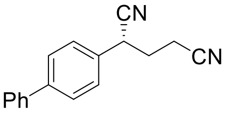

4h

Prepared according to general procedure (D) using **3h** to provide the title compound **4h** as a yellowish solid (93% yield, 95% ee; 1 mmol scale: 87% yield, 95% ee). ^{1}H NMR (400 MHz, $CDCl_3$) δ 7.69 – 7.63 (m, 2H), 7.62 – 7.56 (m, 2H), 7.52 – 7.35 (m, 5H), 4.04 (t, J = 7.5 Hz,1H), 2.66 – 2.53 (m, 1H), 2.53 – 2.41 (m, 1H), 2.41 – 2.21 (m, 2H); ^{13}C NMR (101 MHz, $CDCl_3$) δ 142.1, 140.0, 132.4, 129.1, 128.3, 128.0, 127.8, 127.2, 119.3, 117.9, 35.9, 31.4, 15.2. HRMS (ESI) calcd. for $C_{17}H_{14}N_2$ $[M+Na]^+$ *m/z* 269.1055, found 269.1052.

HPLC (OD-H, 0.46*25 cm, 5 μm, hexane/isopropanol = 8/2, flow 1.0 mL/min, detection at 214 nm) retention time = 24.31 min (major) and 34.52 min (minor). $[\alpha]_D^{20.0}$ = -40.7 (c 1.0, $CHCl_3$).

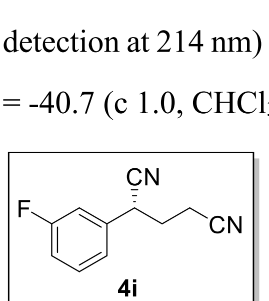

4i

Prepared according to general procedure (D) using **3i** to provide the title compound **4i** as a yellowish oil (86% yield, 91% ee). ^{1}H NMR (400 MHz, $CDCl_3$) δ 7.47 – 7.38 (m, 1H), 7.20 – 7.06 (m, 3H), 4.00 (dd, J = 8.1, 6.9 Hz, 1H), 2.67 – 2.52 (m, 1H), 2.52 – 2.40 (m, 1H), 2.39 – 2.18 (m, 2H); ^{13}C NMR (101 MHz, $CDCl_3$) δ ^{13}C NMR (101 MHz, $CDCl_3$) δ 163.3 (d, J = 249.1 Hz), 135.8 (d, J = 7.4 Hz), 131.5 (d, J = 8.4 Hz), 123.2 (d, J = 3.1 Hz), 118.7, 117.7, 116.3 (d, J = 21.0 Hz), 114.7 (d, J = 23.0 Hz), 35.92, 35.9, 31.4, 15.2. HRMS (ESI) calcd. for $C_{11}H_9N_2F$ $[M+H]^+$ *m/z* 189.0828, found 189.0839.

HPLC (OD-H, 0.46*25 cm, 5 μm, hexane/isopropanol = 8/2, flow 1.0 mL/min, detection at 214 nm) retention time = 12.48 min (minor) and 16.94 min (major). $[\alpha]_D^{20.0}$ = -16.2 (c 0.64, $CHCl_3$).

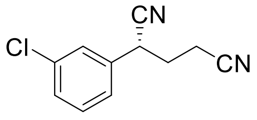

4j

Prepared according to general procedure (D) using **3j** to provide the title compound **4j** as a yellowish oil (77% yield, 89% ee). ^{1}H NMR (400 MHz, $CDCl_3$) δ 7.42 – 7.34 (m, 3H), 7.29 – 7.23 (m, 1H), 3.98 (dd, *J* = 8.2, 6.8 Hz, 1H), 2.67 – 2.52 (m, 1H), 2.52 – 2.39 (m, 1H), 2.38 – 2.17 (m, 2H); ^{13}C NMR (101 MHz, $CDCl_3$) δ 135.7, 135.5, 131.0, 129.5, 127.6, 125.6, 118.7, 117.6, 35.87, 31.37, 15.20. HRMS (ESI) calcd. for $C_{11}H_9N_2Cl$ $[M+Na]^+$ *m/z* 227.0352, found 227.0353.

HPLC (OD-H, 0.46*25 cm, 5 μm, hexane/isopropanol = 8/2, flow 1.0 mL/min, detection at 214 nm) retention time = 13.76 min (minor) and 20.52 min (major). $[\alpha]_D^{20.0}$ = -15.5 (c 0.67, $CHCl_3$).

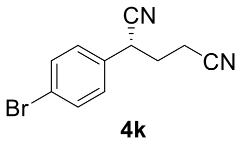

4k

Prepared according to general procedure (D) using **3k** to provide the title compound **4k** as a yellowish oil (89% yield, 94% ee). ^{1}H NMR (400 MHz, $CDCl_3$) δ 7.68 – 7.46 (m, 2H), 7.35 – 7.13 (m, 2H), 3.97 (t, *J* = 8.8 Hz, 1H), 2.65 – 2.52 (m, 1H), 2.50 – 2.37 (m, 1H), 2.35 – 2.15 (m, 2H); ^{13}C NMR (101 MHz, $CDCl_3$) δ 132.9, 132.6, 129.1, 123.3, 118.8, 117.7, 35.7, 31.3, 15.2. HRMS (ESI) calcd. for $C_{11}H_9N_2Br$ $[M+Na]^+$ *m/z* 270.9847, found 270.9865.

HPLC (OD-H, 0.46*25 cm, 5 μm, hexane/isopropanol = 86/14, flow 1.0 mL/min, detection at 214 nm) retention time = 24.13 min (minor) and 28.87 min (major). $[\alpha]_D^{20.0}$ = -29.2 (c 0.97, $CHCl_3$).

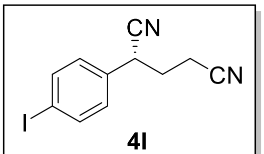

4l

Prepared according to general procedure (D) using **3l** to provide the title compound **4l** as a yellowish oil (83% yield, 92% ee). ^{1}H NMR (400 MHz, $CDCl_3$) δ 7.77 (d, *J* = 8.4 Hz, 2H), 7.11 (d, *J* = 8.4 Hz, 2H), 3.95 (t, *J* = 7.6 Hz, 1H), 2.65 – 2.51 (m, 1H), 2.50 – 2.37 (m, 1H), 2.36 – 2.16 (m, 2H); ^{13}C NMR (101 MHz, $CDCl_3$) δ 138.9, 133.2, 129.2, 118.7, 117.7, 94.9, 35.9, 31.4, 15.2. HRMS (ESI) calcd. for $C_{11}H_9N_2I$ $[M+H]^+$ *m/z* 296.9889, found 296.9890.

HPLC (OD-H, 0.46*25 cm, 5 μm, hexane/isopropanol = 8/2, flow 1.0 mL/min,

detection at 214 nm) retention time = 19.14 min (minor) and 23.26 min (major). $[\alpha]_D^{20.0}$ = -27.5 (c 1.0, $CHCl_3$).

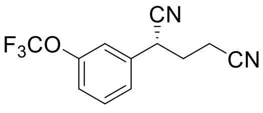
4m

Prepared according to general procedure (D), with modifications of 5 mol% CuSCN and 6 mol% **L1**, using **3m** to provide the title compound **4m** as a yellow oil (91% yield, 93% ee). ^1H NMR (400 MHz, $CDCl_3$) δ 7.49 (t, J = 8.0 Hz, 1H), 7.32 (d, J = 7.9 Hz, 1H), 7.29 – 7.24 (m, 1H), 7.23 (s, 1H), 4.03 (dd, J = 8.2, 6.9 Hz, 1H), 2.66 – 2.54 (m, 1H), 2.53 – 2.42 (m, 1H), 2.38 – 2.19 (m, 2H); ^{13}C NMR (101 MHz, $CDCl_3$) δ 150.1, 135.8, 131.3, 125.8, 121.5, 120.5 (q, J = 258.3 Hz), 120.1, 118.5, 117.6, 36.0, 31.4, 15.2. HRMS (ESI) calcd. for $C_{12}H_9N_2OF_3$ $[M+Na]^+$ *m/z* 277.0565, found 277.0555.

HPLC (OD-H, 0.46*25 cm, 5 μm, hexane/isopropanol = 9/1, flow 1.0 mL/min, detection at 214 nm) retention time = 16.68 min (minor) and 19.44 min (major). $[\alpha]_D^{20.0}$ = -8.67 (c 1.24, $CHCl_3$).

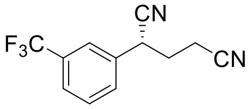
4n

Prepared according to general procedure (D) using **3n** to provide the title compound **4n** as a yellowish oil (75% yield, 94% ee). ^1H NMR (400 MHz, $CDCl_3$) δ 7.78 – 7.52 (m, 4H), 4.08 (dd, J = 8.6, 6.6 Hz, 1H), 2.69 – 2.57 (m, 1H), 2.56 – 2.45 (m, 1H), 2.40 – 2.21 (m, 2H); ^{13}C NMR (101 MHz, $CDCl_3$) δ 134.8 , 132.3 (q, J = 32.8 Hz), 130.8 , 130.4 , 126.1 (q, J = 3.7 Hz), 124.2 (q, J = 3.8 Hz), 123.6 (q, J = 272.6 Hz), 118.5, 117.5, 36.1, 31.4, 15.3. HRMS (ESI) calcd. for $C_{12}H_9N_2F_3$ $[M+H]^+$ *m/z* 239.0796, found 239.0806.

HPLC (OD-H, 0.46*25 cm, 5 μm, hexane/isopropanol = 9/1, flow 1.0 mL/min, detection at 214 nm) retention time = 20.73 min (minor) and 25.55 min (major). $[\alpha]_D^{20.0}$ = -6.31 (c 1.18, $CHCl_3$).

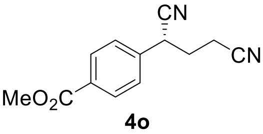
4o

Prepared according to general procedure (D) using **3o** to provide the title compound **4o** as a yellowish oil (89% yield, 95% ee, ee of the crude product. 28% ee of **4o** was obtained after purification by silica gel chromatography). ^1H NMR (400 MHz, $CDCl_3$) δ 8.10 (d, J = 8.4 Hz, 2H), 7.45 (d, J = 8.3 Hz, 2H), 4.06 (dd, J = 8.2, 6.8 Hz, 1H), 3.94 (s, 3H), 2.64 – 2.55 (m, 1H), 2.51 – 2.40 (m, 1H), 2.38 – 2.19 (m, 2H); ^{13}C NMR (101 MHz, $CDCl_3$) δ 166.2, 138.3, 131.1, 130.9, 127.5, 118.6, 117.6, 52.5, 36.2, 31.3, 15.2. HRMS (ESI)

calcd. for $C_{13}H_{12}N_2O_2$ $[M+H]^+$ *m/z* 229.0977, found 229.0971.

HPLC (AD-H, 0.46*25 cm, 5 μm, hexane/isopropanol = 85/15, flow 1.0 mL/min, detection at 214 nm) retention time = 14.42 min (minor) and 16.07 min (major).

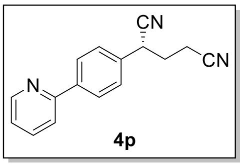

Prepared according to general procedure (D) using **3p** to provide the title compound **4p** as a white solid (92% yield, 91% ee). ^{1}H NMR (400 MHz, $CDCl_3$) δ 8.75 – 8.67 (m, 1H), 8.06 (d, *J* = 8.2 Hz, 2H), 7.83 – 7.71 (m, 2H), 7.47 (d, *J* = 8.4 Hz, 2H), 7.30 – 7.24 (m, 1H), 4.06 (t, *J* = 7.4 Hz, 1H), 2.65 – 2.52 (m, 1H), 2.50 – 2.22 (m, 3H); ^{13}C NMR (101 MHz, $CDCl_3$) δ 156.3, 150.0, 140.4, 137.1, 134.1, 128.2, 127.9, 122.8, 120.7, 119.2, 117.8, 36.0, 31.5, 15.1. HRMS (ESI) calcd. for $C_{16}H_{13}N_3$ $[M+H]^+$ *m/z* 248.1188, found 248.1187.

HPLC (OD-H, 0.46*25 cm, 5 μm, hexane/isopropanol = 7/3, flow 1.2 mL/min, detection at 214 nm) retention time = 24.10 min (major) and 43.66 min (minor). $[\alpha]_D^{20.0}$ = -46.4 (c 0.95, $CHCl_3$).

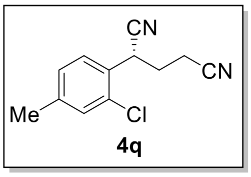

Prepared according to general procedure (D) using **3q** to provide the title compound **4q** as a yellowish oil (87% yield, 89% ee). ^{1}H NMR (400 MHz, $CDCl_3$) δ 7.41 (d, *J* = 7.9 Hz, 1H), 7.26 (s, 1H), 7.19 – 7.13 (m, 1H), 4.41 (dd, *J* = 8.6, 5.8 Hz, 1H), 2.66 – 2.47 (m, 2H), 2.36 (s, 3H), 2.35 – 2.16 (m, 2H); ^{13}C NMR (101 MHz, $CDCl_3$) δ 141.1, 132.6, 131.0, 128.9, 128.8, 128.3, 118.8, 117.8, 33.6, 29.8, 21.0, 15.2. HRMS (ESI) calcd. for $C_{12}H_{11}N_2Cl$ $[M+Na]^+$ *m/z* 241.0508, found 241.0506.

HPLC (AS-H, 0.46*25 cm, 5 μm, hexane/isopropanol = 9/1, flow 1.0 mL/min, detection at 214 nm) retention time = 21.40 min (minor) and 24.09 min (major). $[\alpha]_D^{20.0}$ = 27.3 (c 0.94, $CHCl_3$).

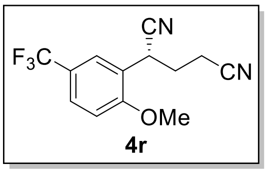

Prepared according to general procedure (D) using **3r** to provide the title compound **4r** as a yellowish oil (96% yield, 95% ee). ^{1}H NMR (400 MHz, $CDCl_3$) δ 7.74 – 7.56 (m, 2H), 7.02 (d, *J* = 9.2 Hz, 1H), 4.37 (dd, *J* = 7.9, 6.7 Hz, 1H), 3.96 (s, 3H), 2.66 – 2.44 (m, 2H), 2.37 – 2.18 (m, 2H); ^{13}C NMR (101 MHz, $CDCl_3$) δ 158.7, δ 128.0 (q, *J* = 3.8 Hz), 125.7 (q, *J* = 3.6 Hz) , 123.9 (q, *J* = 269.9 Hz), 123.8 (q, *J* = 33.3 Hz), 122.8, 118.8, 117.9, 111.3, 56.2, 31.0, 29.3, 15.4. HRMS (ESI) calcd. for $C_{13}H_{11}N_2OF_3$

[M+Na]+ *m/z* 291.0721, found 291.0719.

HPLC (ID, 0.46*25 cm, 5 μm, hexane/isopropanol = 9/1, flow 1.0 mL/min, detection at 214 nm) retention time = 12.08 min (major) and 13.04 min (minor). $[\alpha]_D^{20.0}$ = 17.2 (c 0.94, $CHCl_3$).

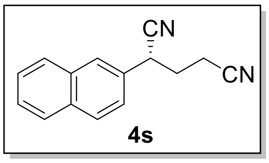

Prepared according to general procedure (D) using **3s** to provide the title compound **4s** as a yellowish oil (94% yield, 93% ee). ^1H NMR (400 MHz, $CDCl_3$) δ 7.93 (d, *J* = 8.5 Hz, 1H), 7.87 (dd, *J* = 6.2, 3.8 Hz, 3H), 7.60 – 7.52 (m, 2H), 7.41 (dd, *J* = 8.5, 1.9 Hz, 1H), 4.17 (t, *J* = 7.2 Hz, 1H), 2.65 – 2.53 (m, 1H), 2.50 – 2.28 (m, 3H); ^{13}C NMR (101 MHz, $CDCl_3$) δ 133.4, 133.2, 130.7, 129.9, 128.1, 128.0, 127.3, 127.2, 126.9, 124.3, 119.3, 117.9, 36.3, 31.4, 15.2. HRMS (ESI) calcd. for $C_{15}H_{12}N_2$ [M+Na]+ *m/z* 243.0898, found 243.0892.

HPLC (OD-H, 0.46*25 cm, 5 μm, hexane/isopropanol = 77/23, flow 1.0 mL/min, detection at 214 nm) retention time = 22.06 min (minor) and 37.34 min (major). $[\alpha]_D^{20.0}$ = -51.2 (c 0.79, $CHCl_3$).

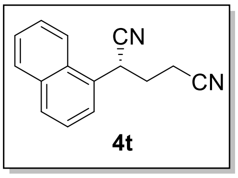

Prepared according to general procedure (D) using **3t** to provide the title compound **4t** as a yellowish oil (83% yield, 94% ee). ^1H NMR (400 MHz, $CDCl_3$) δ 7.98 – 7.88 (m, 3H), 7.70 (dd, *J* = 7.2, 1.1 Hz, 1H), 7.64 (ddd, *J* = 8.6, 6.9, 1.4 Hz, 1H), 7.58 (ddd, *J* = 7.9, 6.9, 1.2 Hz, 1H), 7.52 (dd, *J* = 8.2, 7.2 Hz, 1H), 4.77 (dd, *J* = 8.7, 5.5 Hz, 1H), 2.76 – 2.62 (m, 1H), 2.60 – 2.32 (m, 3H); ^{13}C NMR (101 MHz, $CDCl_3$) δ 134.3, 130.0, 129.8, 129.7, 129.2, 127.7, 126.7, 126.0, 125.6, 121.7, 119.5, 118.0, 33.4, 30.5, 15.5. HRMS (ESI) calcd. for $C_{15}H_{12}N_2$ [M+H]+ *m/z* 221.1079, found 221.1075.

HPLC (OD-H, 0.46*25 cm, 5 μm, hexane/isopropanol = 7/3, flow 1.0 mL/min, detection at 214 nm) retention time = 18.61 min (minor) and 36.99 min (major). $[\alpha]_D^{20.0}$ = 31.4 (c 0.94, $CHCl_3$).

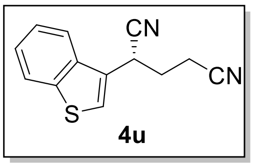

Prepared according to general procedure (D) using **3u** to provide the title compound **4u** as a yellowish oil (50% yield, 95% ee). ^1H NMR (400 MHz, $CDCl_3$) δ 7.95 – 7.90 (m, 1H), 7.82 – 7.77 (m, 1H), 7.56 (s, 1H), 7.51 – 7.42 (m, 2H), 4.45 – 4.38 (m, 1H), 2.70 – 2.60 (m, 1H), 2.55 – 2.39 (m, 3H); ^{13}C NMR (101 MHz, $CDCl_3$) δ 141.1, 135.9, 127.3, 125.8, 125.5, 125.2,

123.7, 120.9, 118.6, 117.9, 30.6, 29.3, 15.3. HRMS (ESI) calcd. for $C_{13}H_{10}N_2S$ $[M+Na]^+$ *m/z* 249.0462, found 249.0455.

HPLC (OD-H, 0.46*25 cm, 5 μm, hexane/isopropanol = 77/23, flow 1.0 mL/min, detection at 214 nm) retention time = 35.35 min (minor) and 41.68 min (major). $[\alpha]_D^{20.0}$ = 37.3 (c 0.22, $CHCl_3$).

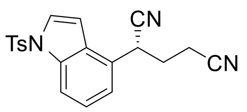

4v

Prepared according to general procedure (D), with modifications of 5 mol% CuSCN and 6 mol% **L1**, using **3v** to provide the title compound **4v** as a yellowish oil (73% yield, 90% ee). ^{1}H NMR (400 MHz, $CDCl_3$) δ 8.03 (d, *J* = 8.3 Hz, 1H), 7.79 (d, *J* = 8.4 Hz, 2H), 7.70 (d, *J* = 3.8 Hz, 1H), 7.35 (t, , *J* = 8.0 Hz 1H), 7.30 – 7.22 (m, 3H), 6.78 (d, *J* = 3.8 Hz, 1H), 4.25 (t, *J* = 7.3 Hz, 1H), 2.63 – 2.48 (m, 1H), 2.37 (s, 3H), 2.46 – 2.21 (m, 3H); ^{13}C NMR (101 MHz, $CDCl_3$) δ 145.6, 135.3, 135.0, 130.2, 128.4, 127.7, 127.0, 125.8, 125.2, 122.3, 119.0, 117.9, 114.4, 105.4, 34.0, 30.4, 21.7, 15.3. HRMS (ESI) calcd. for $C_{20}H_{17}N_3O_2S$ $[M+Na]^+$ *m/z* 386.0939, found 386.0934.

HPLC (OD-H, 0.46*25 cm, 5 μm, hexane/isopropanol = 77/23, flow 1.0 mL/min, detection at 214 nm) retention time = 31.39 min (minor) and 34.52 min (major). $[\alpha]_D^{20.0}$ = -22.6 (c 1.23, $CHCl_3$).

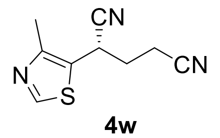

4w

Prepared according to general procedure (D) using **3w** to provide the title compound **4w** as a yellowish oil (57% yield, 93% ee). ^{1}H NMR (400 MHz, $CDCl_3$) δ 8.76 (s, 1H), 4.29 (t, *J* = 7.8 Hz, 1H), 2.67 – 2.57 (m, 1H), 2.56 – 2.46 (m, 1H), 2.51 (s, 3H), 2.46 – 2.36 (m, 1H), 2.31 – 2.19 (m, 1H); ^{13}C NMR (101 MHz, $CDCl_3$) δ 152.1, 152.0, 123.6, 117.8, 117.4, 31.3, 28.2, 15.5, 15.3. HRMS (ESI) calcd. for $C_9H_9N_3S$ $[M+H]^+$ *m/z* 192.0595, found 192.0592.

HPLC (AS-H, 0.46*25 cm, 5 μm, hexane/isopropanol = 8/2, flow 1.0 mL/min, detection at 214 nm) retention time = 24.84 min (minor) and 30.82 min (major). $[\alpha]_D^{20.0}$ = -34.0 (c 0.44, $CHCl_3$).

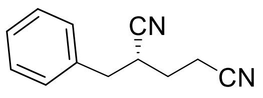

4x

Prepared according to general procedure (D) using **3x** (prepared according to the reported literature[5]) to provide the title compound **4x** as a yellowish oil (82% yield, 23% ee). ^{1}H NMR (400 MHz, Chloroform-*d*) δ 7.40 – 7.29 (m, 3H), 7.26 – 7.22 (m, 2H), 3.09 – 2.87 (m, 3H), 2.71 – 2.49 (m, 2H), 2.08 – 1.88 (m, 2H); ^{13}C NMR (101 MHz, $CDCl_3$) δ 135.7, 129.12, 129.09, 127.9, 120.0, 118.0, 38.0, 32.8, 27.7, 15.5. HRMS (ESI) calcd. for $C_{12}H_{12}N_2$ $[M+H]^+$ *m/z* 185.1079, found 185.1070.

HPLC (ID, 0.46*25 cm, 5 µm, hexane/isopropanol = 8/2, flow 1.0 mL/min, detection at 214 nm) retention time = 10.68 min (major) and 11.35 min (minor). $[\alpha]_D^{20.0}$ = 5.1 (c 1.06, $CHCl_3$).

The ring-opening reactions of several limited substrates:

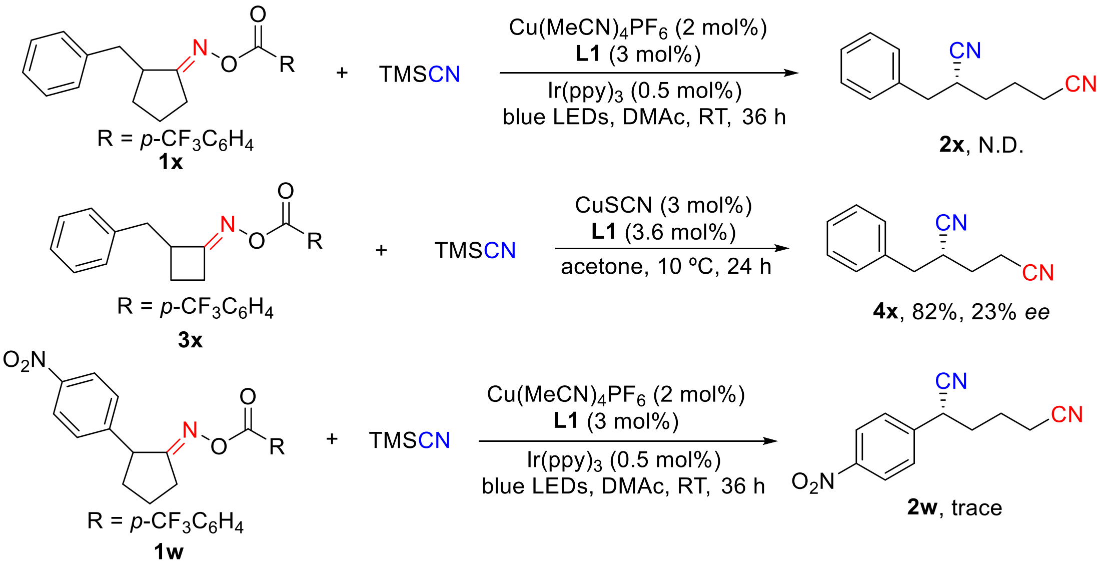

Supplementary Figure 271. The ring-opening reactions of several limited substrates

Mechanism Details

(1) Procedure of the radical trapping experiment with TEMPO:

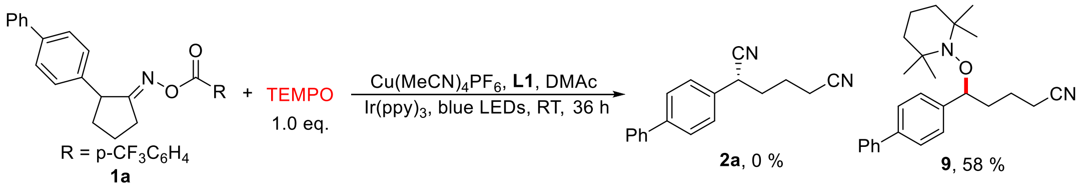

To a 25 mL Schlenk tube, $Cu(CH_3CN)_4PF_6$ (3.7 mg, 0.01 mmol), chiral bisoxazoline ligand **L1** (5.3 mg, 0.015 mmol), $Ir(ppy)_3$ (1.6 mg, 0.0025 mmol) were added in super dry DMAc (5 mL) under N_2 atmosphere. The tube was sealed with a Teflon-lined cap, then the mixture was stirred at room temperature for 0.5 h. The **catalyst solution I** was prepared firstly and used in the next step.

To a 25 mL Schlenk tube containing substrate **1a** (0.1 mmol, 1.0 equiv) and TEMPO (0.1 mmol, 1.0 equiv), **catalyst solution I**(1 mL) and TMSCN (0.15 mmol, 1.5 equiv) were sequentially added under N_2 atmosphere. The tube was sealed with a Teflon-lined cap, and the mixture was stirred at a distance of ~5 cm from a 5 W blue LEDs at room temperature for 36 h .The reaction mixture was diluted with EA(10 mL). The organic layer was washed with brine (3×5 mL) and dried over anhydrous Na_2SO_4. After filtration and concentration, the residue was purified by silica gel chromatography to afford product **9** as a yellowish oil (58 %).

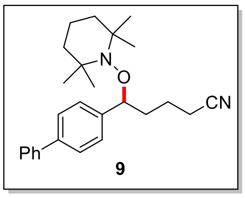

9

^{1}H NMR (400 MHz, $CDCl_3$) δ 7.65 – 7.59 (m, 2H), 7.59 – 7.55 (m, 2H), 7.48 – 7.41 (m, 2H), 7.38 – 7.31 (m, 3H), 4.71 (dd, *J* = 9.1, 4.0 Hz, 1H), 2.32 – 2.15 (m, 3H), 2.11 – 1.94 (m, 1H), 1.60 – 0.54 (m, 20H); ^{13}C NMR (101 MHz, $CDCl_3$) δ 141.8, 140.9, 140.2, 128.9, 128.0, 127.4, 127.1, 126.9, 119.7, 86.1, 59.9, 40.5, 35.0, 34.6, 34.3, 21.5, 20.5, 17.4, 17.3. HRMS (ESI) calcd. for $C_{26}H_{34}N_2O$ $[M+H]^+$ *m/z* 391.2749, found 391.2743.

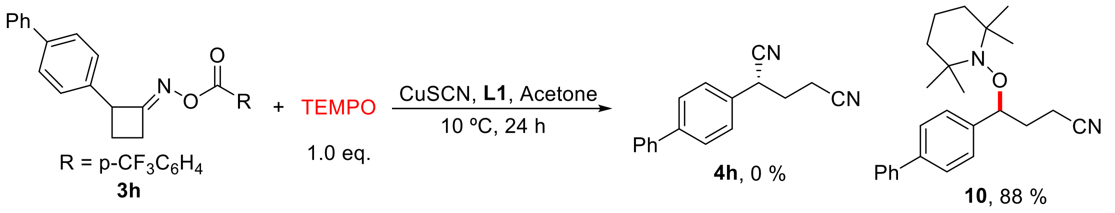

To a 5 mL Schlenk tube, CuSCN (1.8 mg, 0.015 mmol), chiral bisoxazoline ligand **L1** (6.4 mg, 0.018 mmol) were added in degassed Acetone (1 mL) under N_2 atmosphere. The tube was sealed with a Teflon-lined cap, then the mixture was stirred at room temperature for 0.5 h. The **catalyst solution II** was prepared firstly and used in the next step.

To a 25 mL Schlenk tube containing substrate **3h** (0.1 mmol, 1.0 equiv) and TEMPO (0.1 mmol, 1.0 equiv), 0.8 mL Acetone, **catalyst solution II** (200 uL), TMSCN (0.15 mmol, 1.5 equiv) were sequentially added under N_2 atmosphere. The tube was sealed with a Teflon-lined cap, and the mixture was stirred at 10 °C for 24 h. Then Solvent was removed under vacuum and the crude product was purified by flash chromatography on silica gel directly to give the product **10** as a white solid.

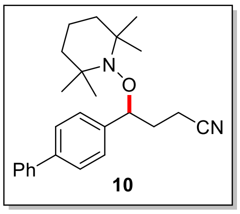

^{1}H NMR (400 MHz, $CDCl_3$) δ 7.66 – 7.55 (m, 4H), 7.49 – 7.41 (m, 2H), 7.40 – 7.31 (m, 3H), 4.85 (dd, J = 8.8, 3.9 Hz, 1H), 2.54 – 2.41 (m, 1H), 2.34 – 2.13 (m, 2H), 2.08 – 1.94 (m, 1H), 1.73 – 0.58 (m, 18H); ^{13}C NMR (101 MHz, $CDCl_3$) δ 140.7, 140.7, 140.3, 128.9, 127.8, 127.5, 127.2, 127.1, 119.7, 84.9, 60.1, 40.5, 34.6, 34.2, 31.6, 20.5, 17.2, 13.4. HRMS (ESI) calcd. for $C_{25}H_{32}N_2O$ $[M+H]^+$ *m/z* 377.2593, found 377.2594.

(2) Procedure of the radical trapping experiment with O_2:

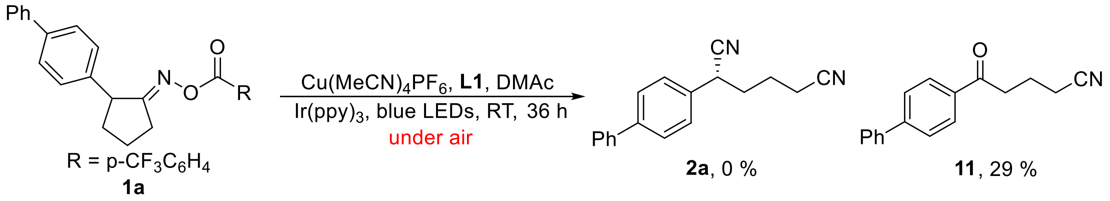

To a 25 mL screw-cap sealed tube containing substrate **1a** (0.1 mmol, 1.0 equiv), **catalyst solution I**(1 mL) and TMSCN (0.15 mmol, 1.5 equiv) were sequentially added under air atmosphere. The tube was sealed with a Teflon screw-cap, and the mixture

was stirred at a distance of ~5 cm from a 5 W blue LEDs at room temperature for 36 h .The reaction mixture was diluted with EA(10 mL). The organic layer was washed with brine (3×5 mL) and dried over anhydrous Na_2SO_4. After filtration and concentration, the residue was purified by silica gel chromatography to afford product **11** as a white solid.

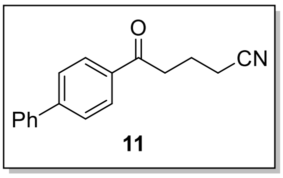

^{1}H NMR (400 MHz, $CDCl_3$) δ 8.08 – 8.02 (m, 2H), 7.74 – 7.68 (m, 2H), 7.66 – 7.61 (m, 2H), 7.52 – 7.45 (m, 2H), 7.45 – 7.38 (m, 1H), 3.22 (t, *J* = 6.8 Hz, 2H), 2.55 (t, *J* = 7.0 Hz, 2H), 2.15 (p, *J* = 6.9 Hz, 2H); ^{13}C NMR (101 MHz, $CDCl_3$) δ 197.9, 146.3, 139.9, 135.3, 129.1, 128.7, 128.5, 127.5, 127.4, 119.5, 36.5, 19.9, 16.8. HRMS (ESI) calcd. for $C_{17}H_{15}NO$ $[M+Na]^+$ *m/z* 272.1051, found 272.1049.

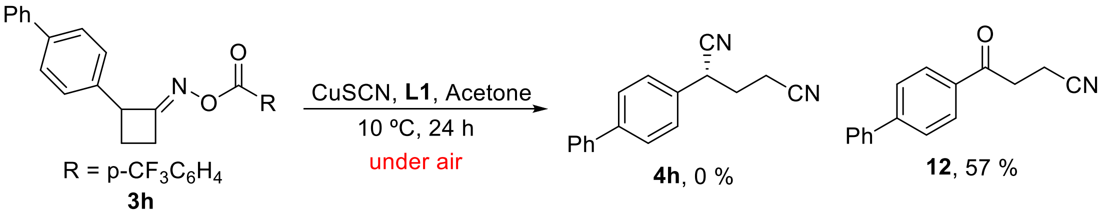

To a 25 mL screw-cap sealed tube containing substrate **3h**(0.1 mmol, 1.0 equiv), 0.8 mL Acetone, **catalyst solution II** (200 uL) and TMSCN (0.15 mmol, 1.5 equiv) were sequentially added under air atmosphere. The tube was sealed with a Teflon screw-cap, and the mixture was stirred at 10 ºC for 24 h .Then Solvent was removed under vacuum and the crude product was purified by flash chromatography on silica gel directly to give the product **12** as a white solid.

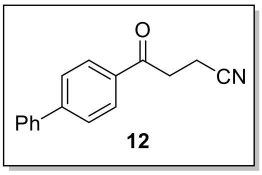

^{1}H NMR (400 MHz, $CDCl_3$) δ 8.08 – 8.00 (m, 2H), 7.76 – 7.69 (m, 2H), 7.68 – 7.61 (m, 2H), 7.53 – 7.45 (m, 2H), 7.46 – 7.40 (m, 1H), 3.42 (t, *J* = 7.2 Hz, 2H), 2.81 (t, *J* = 7.2 Hz, 2H); ^{13}C NMR (101 MHz, $CDCl_3$) δ 195.0, 146.7, 139.7, 134.4, 129.2, 128.8, 128.6, 127.6, 127.4, 119.4, 34.4, 12.0. HRMS (ESI) calcd. for $C_{16}H_{13}NO$ $[M+H]^+$ *m/z* 236.1075, found 236.1070.

Further transformations of the products

(1) Procedure for the synthesis of diamides 5 and 6.[10]

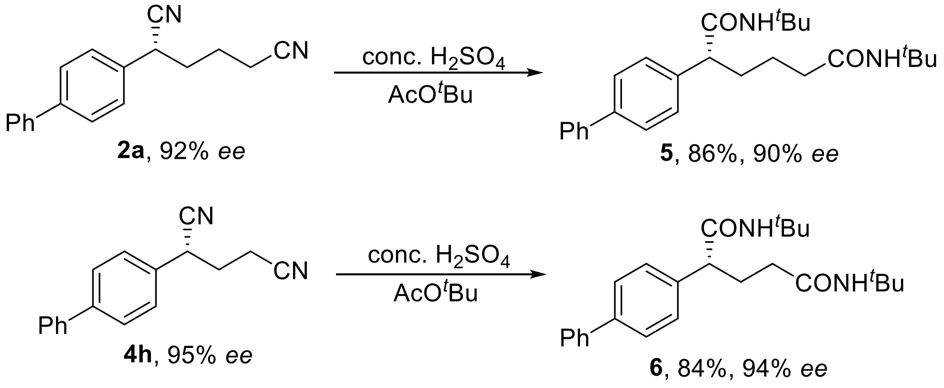

To a stirred solution of **2a** or **4h** (1.0 equiv, 0.1 mmol) in *tert*-butyl acetate(0.4 mL) in a 25 mL screw-cap sealed tube was added slowly conc. H_2SO_4 (10 uL). The tube was sealed with a Teflon screw-cap, and the mixture was stirred at 42 ºC for 2 h. The reaction mixture was diluted with EA(10 mL). The organic layer was washed with sat. aq. $NaHCO_3$ (2×5 mL) and dried over anhydrous Na_2SO_4. After filtration and concentration, the residue was purified by silica gel chromatography to afford product **5** or **6** as a white solid.

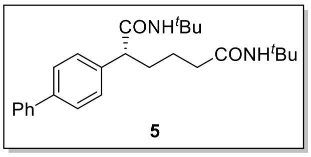

^{1}H NMR (400 MHz, $CDCl_3$) δ 7.61 – 7.50 (m, 4H), 7.43 (t, *J* = 7.6 Hz, 2H), 7.38 – 7.29 (m, 3H), 5.48 (s, 1H), 5.45 (s, 1H), 3.28 (t, *J* = 7.4 Hz, 1H), 2.21 – 2.02 (m, 3H), 1.81 – 1.65 (m, 1H), 1.66 – 1.54 (m, 2H), 1.33 (s, 9H), 1.30 (s, 9H); ^{13}C NMR (101 MHz, $CDCl_3$) δ 172.7, 172.2, 140.8, 140.0, 139.7, 128.9, 128.2, 127.5, 127.3, 127.1, 53.6, 51.4, 51.2, 37.4, 33.3, 28.9, 28.8, 24.0. HRMS (ESI) calcd. for $C_{26}H_{36}O_2N_2$ $[M+H]^+$ *m/z* 409.2855, found 409.2849.

HPLC (AD-H, 0.46*25 cm, 5 μm, hexane/isopropanol = 97/3, flow 1.0 mL/min, detection at 214 nm) retention time = 8.33 min (minor) and 12.16 min (major). $[\alpha]_D^{20.0}$ = -11.6 (c 1.0, $CHCl_3$).

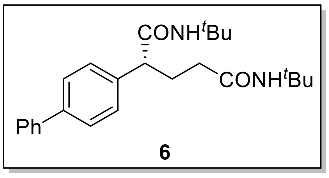

^{1}H NMR (400 MHz, $CDCl_3$) δ 7.64 – 7.51 (m, 4H), 7.48 – 7.40 (m, 2H), 7.39 – 7.30 (m, 3H), 5.56 (s, 1H), 5.41 (s, 1H), 3.44 (dd, J = 8.0, 6.7 Hz, 1H), 2.45 – 2.27 (m, 1H), 2.25 – 1.96 (m, 3H), 1.34 (s, 9H), 1.30 (s, 9H); ^{13}C NMR (101 MHz, $CDCl_3$) δ 172.5, 172.0, 140.7, 140.1, 139.1, 128.9, 128.4, 127.5, 127.4, 127.1, 52.2, 51.4, 51.2, 35.2, 29.9, 28.9, 28.8. HRMS (ESI) calcd. for $C_{25}H_{34}O_2N_2$ $[M+H]^+$ *m/z* 395.2699, found 395.2692.

HPLC (AD-H, 0.46*25 cm, 5 μm, hexane/isopropanol = 97/3, flow 1.0 mL/min, detection at 214 nm) retention time = 7.41 min (minor) and 16.65 min (major). $[\alpha]_D^{20.0}$ = -16.5 (c 1.0, $CHCl_3$).

(2) Procedure for the synthesis of diamines 7 and 8.[11]

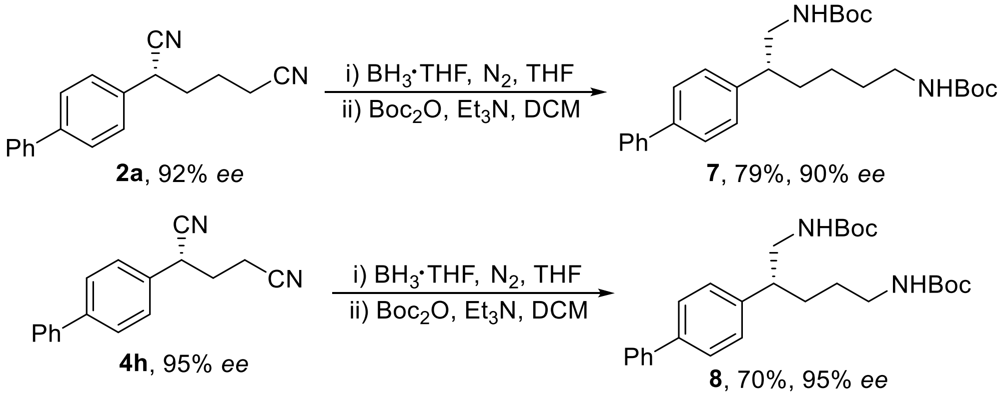

To the solution of **2a** or **4h** (0.1 mmol) in THF (0.4 mL) was added BH_3·THF (0.6 mmol, 1M in THF) at room temperature under N_2 atmosphere, then the mixture was refluxed for 3 h. The reaction was quenched by the dropwise addition of MeOH. Then solvent was removed under vacuum. And 6 M aqueous HCl (~3 mL)was added followed by refluxing another 2 h. After cooling to room temperature, the solution was made basic with 6 M aqueous NaOH. Then it was extracted with DCM (5×10 mL), dried over Na_2SO_4, filtered. After DCM was removed under vacuum, 2 mL DCM, 2.4 eq. Et_3N (0.24 mmol) and 2.4 eq. Boc_2O (0.24 mmol)were added. The mixture was stirred for 3 h at room temperature, and purified by column chromatography directly to afford the product **7** or **8** as a colorless oil.

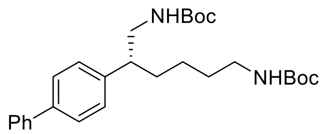

7

^{1}H NMR (400 MHz, $CDCl_3$) δ 7.67 – 7.49 (m, 4H), 7.44 (t, *J* = 7.6 Hz, 2H), 7.34 (t, *J* = 7.3 Hz, 1H), 7.22 (d, *J* = 8.0 Hz, 2H), 4.52 (s, 1H), 4.46 (s, 1H), 3.63 – 3.44 (m, 1H), 3.26 – 3.11 (m, 1H), 3.10 – 2.96 (m, 2H), 2.78 (s, 1H), 1.74 – 1.53 (m, 3H), 1.52 – 1.35 (m, 19H), 1.33 – 1.14 (m, 2H); ^{13}C NMR (101 MHz, $CDCl_3$) δ 156.1, 141.9, 140.9, 139.7, 128.9, 128.3, 127.5, 127.3, 127.1, 79.3, 79.1, 46.3, 45.9, 40.4, 33.3, 30.2, 28.52, 28.49, 24.6. HRMS (ESI) calcd. for $C_{28}H_{40}O_4N_2$ $[M+Na]^+$ *m/z* 491.2886, found 491.2885.

HPLC (AD-H, 0.46*25 cm, 5 μm, hexane/isopropanol = 9/1, flow 1.0 mL/min, detection at 214 nm) retention time = 9.63 min (major) and 11.64 min (minor). $[\alpha]_D^{20.0}$ = 32.3 (c 1.0, $CHCl_3$).

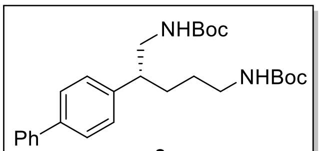

8

^{1}H NMR (400 MHz, Chloroform-*d*) δ 7.57 (dd, *J* = 14.5, 7.8 Hz, 4H), 7.44 (t, *J* = 7.6 Hz, 2H), 7.34 (t, *J* = 7.3 Hz, 1H), 7.23 (d, *J* = 8.0 Hz, 2H), 4.49 (s, 1H), 3.62 – 3.42 (m, 1H), 3.30 – 3.13 (m, 1H), 3.15 – 2.96 (m, 2H), 2.80 (s, 1H), 1.77 – 1.67 (m, 1H), 1.66 – 1.52 (m, 2H), 1.52 – 1.31 (m, 19H); ^{13}C NMR (101 MHz, $CDCl_3$) δ 156.1, 141.6, 140.9, 139.8, 128.9, 128.3, 127.5, 127.3, 127.1, 79.3, 79.2, 46.3, 45.6, 40.5, 30.6, 28.5, 28.5, 28.0. HRMS (ESI) calcd. for $C_{27}H_{38}O_4N_2$ $[M+Na]^+$ *m/z* 477.2729, found 477.2723.

HPLC (OD-H, 0.46*25 cm, 5 μm, hexane/isopropanol = 19/1, flow 1.0 mL/min, detection at 214 nm) retention time = 17.83 min (minor) and 21.48 min (major). $[\alpha]_D^{20.0}$ = 32.6 (c 1.0, $CHCl_3$).

Supplementary References

1. Zhao, B., Chen, C., Lv, J., Li, Z., Yuan, Y., Shi, Z. Photoinduced fragmentation-rearrangement sequence of cycloketoxime esters. *Org. Chem. Front.* **5**, 2719-2722 (2018).
2. Zhao, B., Tan, H., Chen, C., Jiao, N., Shi, Z. Photoinduced C−C bond cleavage and oxidation of cycloketoxime esters. *Chin. J. Chem.* **36**, 995-999 (2018).

3. Xu, Y., Su, T.-S., Huang, Z.-X., Dong, G. Practical direct α-arylation of cyclopentanones by palladium/enamine cooperative catalysis. *Angew. Chem. Int. Ed*. **55**, 2559-2563 (2016).
4. Wang, S., Chen, G., Kayser, M. M., Iwaki, H., Lau, P. C.K., Hasegawa, Y. Baeyer-Villiger oxidations catalyzed by engineered microorganisms: Enantioselective synthesis of δ-valerolactones with functionalized chains. *Can. J. Chem.* **80**, 613-621 (2002).
5. Ai, W., Liu, Y., Wang, Q., Lu, Z., Liu, Q. Cu-catalyzed redox-neutral ring cleavage of cycloketone *O*-acyl oximes: chemodivergent access to distal oxygenated nitriles. *Org. Lett.* **20**, 409-412 (2018).
6. Wang, Y., Muratore, M. E., Rong, Z., Echavarren, A. M. Formal (4+1) cycloaddition of methylenecyclopropanes with 7-aryl-1,3,5-cycloheptatrienes by triple gold(I) catalysis. *Angew. Chem. Int. Ed.* **53**, 14022-14026 (2014).
7. Yu, L., Ren, L., Yi, R., Wu, Y., Chen, T., Guo, R. Iron salt, a cheap, highly efficient and environment-friendly metal catalyst for Se−Se bond cleavage and the further reaction with methylenecyclopropanes under mild conditions. *J. Organomet. Chem.* **696**, 2228-2233 (2011).
8. Cho, H., Iwama, Y., Sugimoto, K., Mori, S., Tokuyama, H. Regioselective synthesis of heterocycles containing nitrogen neighboring an aromatic ring by reductive ring expansion using diisobutylaluminum hydride and studies on the reaction mechanism. *J. Org. Chem.* **75**, 627-636 (2010).
9. Nishimura, T., Uemura, S. Palladium(0)-catalyzed ring cleavage of cyclobutanone oximes leading to nitriles via *β*-carbon elimination. *J. Am. Chem. Soc.* **122**, 12049-12050 (2000).
10. Reddy, K. L. An efficient method for the conversion of aromatic and aliphatic nitriles to the corresponding *N-tert*-butyl amides: a modified Ritter reaction. *Tetrahedron Lett*. **44**, 1453-1455 (2003).
11. Guo, Q., Wang, M., Peng, Q., Huo, Q., Liu, Q., Wang, R., Xu, Z. Dual-functional chiral Cu-catalyst-induced photoredox asymmetric cyanofluoroalkylation of

alkenes. *ACS Catal*. **9**, 4470-4476 (2019).
